# Supplementary material for: Extended embryo retention and viviparity in the first amniotes
Source: Nat Ecol Evol. 2023 Jun 12;7(7):1131–40. doi: 10.1038/s41559-023-02074-0 (PMC10333127; doi:10.1038/s41559-023-02074-0)
Supplement: Supplementary file 1 — Supplementary Results, Tables 1–45 and References. [file 41559_2023_2074_MOESM1_ESM.pdf]

nature ecology & evolution

Article https://doi.org/10.1038/s41559-023-02074-0

Extended embryo retention and viviparity in the first amniotes

In the format provided by the authors and unedited

SUPPLEMENTARY INFORMATION FOR

Extended embryo retention and viviparity in the first amniotes

Baoyu Jiang[1*], Yiming He[1], Armin Elsler[2], Shengyu Wang[1], Joseph N. Keating[2], Junyi Song[1], Stuart L. Kearns[2] & Michael J. Benton[2]

Supplementary Results	1
Anatomical description of the specimen	1
Evidence that the choristodere embryo is an archosauromorph	3
References cited in the Supplementary Text	5
Supplementary Tables 1–45	7
References cited in Supplementary Tables	168

Supplementary Results

Anatomical description of the specimen

Two categories of mineralization of the skeleton are recognized, both of which are lighter and denser than the embedding sediments in the CT scan slices. Type 1 are well-ossified bones that are well-organized porous or compact with distinct surfaces. Type 2 are poorly mineralized bones that are disorderly porous with irregular and blurry surfaces[1]. The former includes the dermal bones and well ossified chondral elements, while the latter represents mineralized or partly ossified cartilages, such as the quadrate, dorsal neural arch, ilium and pubis (Fig. 3D–G). Even though many taphonomic processes could result in loss of skeletal elements, the absence of skeletal structures in embryos is most likely because mineralization of the structure had not yet begun when the embryos died[2]. Hence the absence of some bones is attributed to non-ossification, except those that were lost during the collecting process, as noted in the following description.

The skull is very large (31.7 mm long), around 46.58% of its snout-vent length (68.05

[1] State Key Laboratory for Mineral Deposits Research, School of Earth Sciences and Engineering and Frontiers Science Center for Critical Earth Material Cycling, Nanjing University, Nanjing 210023, China

[2] School of Earth Sciences, University of Bristol, Bristol BS8 1RJ, UK. *e-mail: byjiang@nju.edu.cn

mm) (Fig. 3A, B). The chondrocranial and splanchnocranial bones are poorly mineralized. Only the supraoccipital and otic capsule are present in the former, and stapes, quadrate and articular in the latter (Fig. 3D). The dermatocranial ossification is approximately complete. The paired prefrontals, frontals and parietals meet one another along the midline, and the frontal-prefrontal and frontal-parietal sutures are well defined (Fig. 3C). The frontals and the parietals form a uniformly curved cranial vault. The squamosal ramus of the parietal extends about half-way along the posterior border of the upper temporal opening but does not contact the squamosal probably because of damage during preservation or collection. The upper and lower temporal openings are both anteroposteriorly elongate and positioned largely above one another. Their posterior edges are formed by the squamosal articulating with the parietal and postorbital mediodorsally, and the quadratojugal and quadrate lateroventrally, respectively. Their anterior edges are separated from the orbits by the jugals, postorbitals, and postfrontals which constitute the posterior and lateral edges of the orbits. The orbit is also relatively large (8.1 mm long by 5.3 mm wide) and much wider than the interorbital bar (1.5 mm wide). The anterior edge of the orbit consists of the lacrimal articulating with prefrontal and jugal. The jugal forms the ventral edge of the orbit and extends between the maxilla and lacrimal (Extended Data Fig. 1A). The lacrimal duct extends anteriorly into the olfactory cavity.

The snout is long (13.8 mm, 43.5% of the skull length). It is broad and flat in front of the orbits, and gradually tapers to about the midpoint along the snout. It comprises a pair of maxillae which articulate with paired premaxillae anteriorly forming a confluent external naris, and behind that a single midline nasal. The maxilla has a strongly inrolled dorsal portion and a narrow tooth-bearing medial shelf ventrally. 70 marginal teeth are present in the upper tooth row with 64 on the maxillae and 6 enlarged ones on the premaxillae. All the teeth are simple cones, without distinct ridges or crests. The ventral part of the skull is poorly preserved largely due to the damage caused by the split of the specimen along its ventral surface during collection. Fragments of pterygoids are present between and posterior to the orbits with ectopterygoids and palatines on both lateral sides. The palatine is less ossified than the pterygoid and ectopterygoids. Palatal teeth are locally preserved on the palatine. No vomer is recognizable. Both mandibles are partly preserved, including dentary, surangular and articular (Extended Data Fig. 1).

Most of the vertebral column is preserved except portions around the sacral region which were lost during collection. The centra are well ossified and articulate with each other in the cervical and dorsal regions (Fig. 3E) but have open ends and large notochodal canals in the caudal region (Fig. 3F; Extended Data Fig. 2). Only the bases of neural arches are ossified and separated from the centra in the cervical and dorsal regions (Fig. 3F). In contrast, the neural arches are well developed in the caudal region where they suture and gradually fuse with the centra (Fig. 3H). Neural spines are only weakly developed in the distal caudal region. Transverse processes are present in the dorsal vertebrae and poorly ossified. Ribs are present on most vertebrae except in the sacral and distal caudal regions. They are well ossified in the dorsal regions, but poorly ossified in proximal caudal and cervical regions.

Only the left forelimb, right hindlimb and their corresponding girdles are preserved. The pectoral girdle consists of a curved clavicle and a rectangular scapular blade, both of which are well ossified (Fig. 3E). The forelimb is folded, including the humerus, radius, ulna, metacarpals 1-5, and fragments belonging to phalanges 2-5. The pelvic girdle comprises a well ossified ischium and the poorly mineralized ilium and pubis that form an acetabulum (Fig. 3F). The hind limb is stretched along the tail and noticeably longer than the forelimb. It consists of the femur, tibia, fibula, metatarsals 1–5 and phalanges 1–5. No carpal or tarsal elements are ossified. The femur shows a higher degree of ossification than the remaining limb bones. The proximal end is rounded and the distal end is rectangular (Fig. 3F). Both ends of the remaining limb bones are rectangular or concave, leaving open epiphyseal areas (Extended Data Fig. 2).

Evidence that the choristodere embryo is an archosauromorph

The phylogenetic position of choristoderes has been debated. They are diapsids, but whether a basal clade of archosauromorphs[3], a sister group of archosauromorphs, or basal to (archosauromorphs + lepidosauromorphs)[4,5] has been uncertain. Here we confirm the archosauromorph affinity based on several observations in which our embryo specimen resembles archosaurs but differs from lepidosaurs at a similar ontogenetic stage. First, the parietals of the embryo contact each other medially and the frontals anteriorly form complete sutures (Fig. 3C). This is consistent with the skull of known archosaurs at similar ontogenetic

stages, such as crocodilians[6], dinosaurs[7], and basal clades of extant birds[8], but differs from that of known lepidosaurs[9]and basal diapsids[10] at similar ontogenetic stages, in which a fontanelle exists between the parietals and/or between parietals and frontals. Second, sutures between neural arches and vertebral centra are closed in distal caudal vertebrae (Fig. 3H) but still open in proximal caudal and presacral vertebrae of the embryo (Fig. 3G). This is in accord with the pattern of crocodilians at late ontogenetic stages[11,12], but contrasts with lepidosaurs at late ontogenetic stages, in which the sutures remain open in the distal caudal vertebrae before hatching[9,13] and they are often closed throughout the presacral vertebrae in many neonatal lepidosaurs[13]. Third, the pelvic girdle comprises a well ossified ischium and the poorly mineralized ilium and pubis that form an acetabulum (Fig. 3F). The excellent ossification of the ischium indicates that it is the first ossified element in the pelvic girdle, as in crocodilians but contrary to that in lepidosaurs, in which the ischium is the last element to start ossification[9,13]. Finally, the dermatocranial bones of the embryo are mainly composed of trabecular bone (Fig. 3C), leaving the margins and sutures ill-defined, similar to these in developing crocodiles in contrast to the more compact, almost avascular lamellar bone in developing lepidosaurs[13].

These observations show that the skeletal development of the embryo conforms to that of modern archosaurs but deviates from that of lepidosaurs and basal diapsids, supporting the phylogenetic position of choristoderes as archosauromorphs.

References cited in the Supplementary Text

1. Montero, R., Gans, C. & Luisa Lions, M. Embryonic development of the skeleton of *Amphisbaena darwini* heterozonata (Squamata: Amphisbaenidae). *J. Morph.* **239**, 1–25 (1999).
2. Unwin, D. M. & Deeming, D. C. Prenatal development in pterosaurs and its implications for their postnatal locomotory ability. *Proc. R. Soc. B* **286**, 20190409, doi:10.1098/rspb.2019.0409 (2019).
3. Scheyer, T. M. *et al.* A new, exceptionally preserved juvenile specimen of *Eusaurosphargis dalsassoi* (Diapsida) and implications for Mesozoic marine diapsid phylogeny. *Sci Rep* **7**, 4406, doi: 10.1038/s41598-017-04514-x (2017).
4. Matsumoto, R., Dong, L., Wang, Y. & Evans, S. E. The first record of a nearly complete choristodere (Reptilia: Diapsida) from the Upper Jurassic of Hebei Province, People's Republic of China. *J. Syst. Palaeontol.* **17**, 1031–1048, doi: 10.1080/14772019.2018.1494220 (2019).
5. Zhang, W. & Gao, K.-Q. Early Cretaceous evolution of choristoderes in western Liaoning based on geographic and stratigraphic evidence. *J. Palaeogeogr.* **16**, 205–216 (2014).
6. Rieppel, O. Studies on skeleton formation in reptiles. V. Patterns of ossification in the skeleton of *Alligator mississippiensis* DAUDIN (Reptilia, Crocodylia). *Zool. J. Linn. Soc.* **109**, 301–325, doi: 10.1006/zjls.1993.1040 (1993).
7. Reisz, R. R., Scott, D., Sues, H. D., Evans, D. C. & Raath, M. A. Embryos of an early Jurassic prosauropod dinosaur and their evolutionary significance. *Science* **309**, 761–764, doi: 10.1126/science.1114942 (2005).
8. Maxwell, E. E. Comparative ossification and development of the skull in palaeognathous birds (Aves: Palaeognathae). *Zool. J. Linn. Soc.* **156**, 184–200, doi: 10.1111/j.1096-3642.2009.00480.x (2009).
9. Maisano, J. A survey of state of ossification in neonatal squamates. *Herpetol. Monogr.* **15**, 135–157, doi: 10.2307/1467041 (2001).
10. Sander, P. M. & Greenwood, P. H. The pachypleurosaurids (Reptilia: Nothosauria) from the Middle Triassic of Monte San Giorgio (Switzerland) with the description of a new species. *Phil. Trans. R. Soc. Lond. B* **325**, 561–666, doi: 10.1098/rstb.1989.0103 (1989).

11. Ikejiri, T. Histology-based morphology of the neurocentral synchondrosis in *Alligator mississippiensis* (Archosauria, Crocodylia). *Anat. Rec.* **295**, 18–31, doi: 10.1002/ar.21495 (2012).

12. Brochu, C. Closure of neurocentral sutures during crocodilian ontogeny: implications for maturity assessment in fossil archosaurs. *J. Vertebr. Paleontol.* **16**, 49–62, doi:10.1080/02724634.1996.10011283 (1996).

13. Rieppel, O. Studies on skeleton formation in reptiles. Patterns of ossification in the skeleton of *Lacerta agilis exigua* Eichwald (Reptilia, Squamata). *J. Herpetol.* **28**, 145-153 (1994).

14. Pagel, M. Detecting correlated evolution on phylogenies: a general method for the comparative analysis of discrete characters. *Proc. R. Soc. Lond. B* **255**, 37–45 (1994).

Table S1. Trait data and references used in our ancestral state estimation analysis. '*' indicates extinct species; '-' indicates inapplicable and '?' indicates unknown state.

Clade	Species	Reproduction mode	Egg shell mineralisation	EER	Reference
**Mammalia**	*Ornithorhynchus anatinus*	oviparous	membrane-shelled	absent	Hughes (1993)[1]
**Mammalia**	*Kryptobaatar dashzevegi**	viviparous	-	present	Kielan-Jaworowska (1979)[2]
**Mammalia**	*Macropus rufus*	viviparous	-	present	Laurin (2005)[3]
**Mammalia**	*Elephas asiaticus*	viviparous	-	present	Laurin (2005)[3]
**Mammalia**	*Oreodon culbertsoni**	viviparous	-	present	O'Harra (1930)[4]
**Mammalia**	*Maiacetus inuus**	viviparous	-	present	Gingerich et al. (2009)[5]; Thewissen & McLellan (2009)[6]
**Mammalia**	*Homo sapiens*	viviparous	-	present	Laurin (2005)[3]
**Mesosauridae**	*Mesosaurus tenuidens**	?	?	present	Blackburn & Sidor (2015)[7]
**Sauropterygia**	*Neusticosaurus peyeri**	viviparous	-	present	Blackburn & Sidor (2015)[7]; Sander (1989)[8]
**Sauropterygia**	*Lariosaurus* sp.*	viviparous	-	present	Blackburn & Sidor (2015)[7]; Renesto et al. (2003)[9]
**Sauropterygia**	*Keichosaurus hui**	viviparous	-	present	Blackburn & Sidor (2015)[7]; Cheng et al. (2004)[10]
**Sauropterygia**	*Dolichorhynchops osborni**	viviparous	-	present	Blackburn & Sidor (2015)[7]; Rothschild & Martin (1993)[11]
**Sauropterygia**	*Polycotylus latipinnus**	viviparous	-	present	Blackburn & Sidor (2015)[7]; O'Keefe and Chiappe (2011)[12]
**Ichthyopterygia**	*Chaohusaurus*	viviparous	-	present	Blackburn &

	*geishanensis**				Sidor (2015)[7]; Motani et al. (2014)[13]
**Ichthyopterygia**	*Mixosaurus* sp.*	viviparous	-	present	Blackburn & Sidor (2015)[7]; Brinkman (1996)[14]
**Ichthyopterygia**	*Besanosaurus leptorhynchus**	viviparous	-	present	Blackburn & Sidor (2015)[7]; Dal Sasso & Pinna (1996)[15]
**Ichthyopterygia**	*Shonisaurus popularis**	viviparous	-	present	Blackburn & Sidor (2015)[7]; Camp (1980)[16] [Lomax & Massare 2012][17]
**Ichthyopterygia**	*Qianichthyosaurus zhoui**	viviparous	-	present	Blackburn & Sidor (2015)[7]; Wang et al. (2008)[18]
**Ichthyopterygia**	*Temnodontosaurus* Sp.*	viviparous	-	present	Blackburn & Sidor (2015)[7]; Böttcher (1990)[19] [Motani 2005][20]
**Ichthyopterygia**	*Leptonectes tenuirostris**	viviparous	-	present	Blackburn & Sidor (2015)[7]; Lomax & Massare (2012)[21]
**Ichthyopterygia**	*Ichthyosaurus communis**	viviparous	-	present	Blackburn & Sidor (2015)[7]; Deeming et al. (1993)[22]
**Ichthyopterygia**	*Stenopterygius quadriscissus**	viviparous	-	present	Blackburn & Sidor (2015)[7]; McGowan (1979)[23]; Maxwell (2012)[24]
**Ichthyopterygia**	*Stenopterygius triscissus**	viviparous	-	present	Blackburn & Sidor (2015)[7]; Maxwell (2012)[24]
**Ichthyopterygia**	*Maiaspondylus lindoei**	viviparous	-	present	Blackburn & Sidor (2015)[7]; Maxwell & Caldwell (2003,

					2006)[25,26]
**Ichthyopterygia**	*Platypterygius australis**	viviparous	-	present	Blackburn & Sidor (2015)[7]; Kear et al. (2003)[27]; Kear & Zammit (2014)[28]
**Ichthyopterygia**	*Platypterygius longmani**	viviparous	-	present	Blackburn & Sidor (2015)[7]; Kear et al. (2003)[27]
**Rhynchocephalia**	*Sphenodon punctatus*	oviparous	parchment	absent	Packard et al. (1982)[29]; Moffat (1985)[30]
**Squamata**	*Yabeinosaurus tenuis**	viviparous	-	present	Blackburn & Sidor (2015)[7]; Wang & Evans (2011)[31]
**Squamata**	*Diplodactylus vittatus*	oviparous	parchment	present	Shine (1983)[32]; Deeming (1988)[33]; Kluge (1967)[34]
**Squamata**	*Anepischetosia maccoyi*	oviparous	parchment	present	Lucas & Frost (1893)[35]
**Squamata**	*Podarcis siculus*	oviparous	parchment	present	Shine (1983)[32]; Schleich & Kästle (1988)[36]
**Squamata**	*Elaphe guttata*	oviparous	parchment	present	Shine (1983)[32]; Schleich & Kästle (1988)[36]
**Squamata**	*Carsosaurus marchesetti**	viviparous	-	present	Blackburn & Sidor (2015)[7]; Caldwell & Lee (2001)[37]
**Squamata**	*Plioplatecarpus primaevus**	viviparous	-	present	Blackburn & Sidor (2015)[7]; Bell et al. (1996)[38]
**Squamata**	Phu Phok Embryo*	oviparous	rigid	?	Fernandez et al. (2015)[39]
**Squamata**	*Diploglossus delasagra*	oviparous	parchment	present	Shine (1983)[32]; Barbour & Ramsden (1919)[40]
**Squamata**	*Furcifer lateralis*	oviparous	parchment	absent	Shine (1983)[32]; Gray (1845)[41]

**Squamata**	*Sceloporus clarkii*	oviparous	parchment	present	Shine (1983)[32]; Packard & Demarco (1991)[42]
**Squamata**	*Sceloporus scalaris*	oviparous	parchment	present	Shine (1983)[32]; Packard & Demarco (1991)[42]
**Testudines**	*Pelusios sinuatus*	oviparous	parchment	absent	Kusuda et al. (2013)[43]; Laurin (2005)[3]
**Testudines**	*Acanthochelys radiolata*	oviparous	rigid	absent	Kusuda et al. (2013)[43]; Laurin (2005)[3]
**Testudines**	*Pelodiscus sinesis*	oviparous	rigid	absent	Kusuda et al. (2013)[43]; Laurin (2005)[3]
**Testudines**	*Adocus* sp.*	oviparous	rigid	?	Zelenitsky et al. (2008)[44]
**Testudines**	*Chelonoidis carbonaria*	oviparous	rigid	absent	Kusuda et al. (2013)[43]; Laurin (2005)[3]
**Testudines**	*Kinosternon baurii*	oviparous	rigid	absent	Kusuda et al. (2013)[43]; Laurin (2005)[3]
**Testudines**	*Caretta caretta*	oviparous	parchment	absent	Kusuda et al. (2013)[43]; Laurin (2005)[3]
**Testudines**	*Desmatochelys padillai**	oviparous	rigid	?	Kusuda et al. (2013)[43]
**Protorosauria**	*Dinocephalosaurus orientalis**	viviparous	-	present	Liu et al. (2016)[45]
**Choristodera**	*Philydrosaurus proseilus**	viviparous	-	present	Blackburn & Sidor (2015)[7]; Lü et al. (2014)[46]
**Choristodera**	*Ikechosaurus* sp.*	oviparous	parchment	?	Present study
**Choristodera**	*Monjurosuchus splendens**	viviparous	-	present	Blackburn & Sidor (2015)[7]; Wang et al. (2005)[47]
**Crocodylia**	*Alligator mississippiensis*	oviparous	rigid	absent	Packard & Demarco (1991)[42]; Laurin (2005)[3]
**Crocodylia**	*Crocodylus niloticus*	oviparous	rigid	absent	Packard & Demarco

					(1991)[42]; Laurin (2005)[3]
**Pterosauria**	*Pterodaustro guinazui**	oviparous	rigid	?	Chiappe et al. (2004)[48]; Norell et al. (2020)[49]
**Pterosauria**	*Hamipterus tianshanensis**	oviparous	parchment	?	Wang et al. (2014)[50]; Wang et al. (2017)[51]; Norell et al. (2020)[49]
**Pterosauria**	Pterodactyloid*	oviparous	membrane-shelled	?	Ji et al. (2004)[52]; Norell et al. (2020)[49]
**Pterosauria**	Ornithocheirid*	oviparous	membrane-shelled	?	Wang & Zhou (2004)[53]; Norell et al. (2020)[49]
**Dinosauria**	*Protoceratops andrewsi**	oviparous	membrane-shelled	?	Norell et al. (2020)[49]
**Dinosauria**	*Maiasaura peeblesorum**	oviparous	rigid	?	Norell et al. (2020)[49]
**Dinosauria**	*Telmatosaurus transsylvanicus**	oviparous	rigid	?	Norell et al. (2020)[49]
**Dinosauria**	*Massospondylus carinatus**	oviparous	weakly mineralised	?	Owen (1854)[54]; Yates & Barrett (2010)[55]; Norell et al. (2020)[49]
**Dinosauria**	*Mussaurus patagonicus**	oviparous	membrane-shelled	?	Cerda et al. (2014)[56]; Norell et al. (2020)[49]
**Dinosauria**	Saltasaurid*	oviparous	rigid	?	Norell et al. (2020)[49]
**Dinosauria**	Titanosaurian*	oviparous	rigid	?	Norell et al. (2020)[49]
**Dinosauria**	*Sinosauropteryx**	oviparous	rigid	?	Norell et al. (2020)[49]
**Dinosauria**	*Oviraptor philoceratops**	oviparous	rigid	?	Norell et al. (2020)[49]
**Dinosauria**	*Heyuannia huangi**	oviparous	rigid	?	Norell et al. (2020)[49]
**Dinosauria**	*Deinonychus antirrhopus**	oviparous	rigid	?	Norell et al. (2020)[49]
**Aves**	*Enantiornithine**	oviparous	rigid	?	Norell et al. (2020)[49]; Wiemann et al.

					(2018)[57]
**Aves**	*Gobipipus reshetovi**	oviparous	rigid	?	Norell et al. (2020)[49]; Wiemann et al. (2018)[57]
**Aves**	*Struthio camelus*	oviparous	rigid	absent	Norell et al. (2020)[49]; Wiemann et al. (2018)[57]; Laurin (2005)[3]
**Aves**	*Casuarius casuarius*	oviparous	rigid	absent	Norell et al. (2020)[49]; Wiemann et al. (2018)[57]; Laurin (2005)[3]
**Aves**	*Gallus gallus*	oviparous	rigid	absent	Norell et al. (2020)[49]; Wiemann et al. (2018)[57]; Laurin (2005)[3]
**Aves**	*Anas platyrhynchos*	oviparous	rigid	absent	Norell et al. (2020)[49]; Wiemann et al. (2018)[57]; Laurin (2005)[3]
**Aves**	*Phoenicopterus ruber*	oviparous	rigid	absent	Norell et al. (2020)[49]; Wiemann et al. (2018)[57]; Laurin (2005)[3]
**Aves**	*Columba palumbis*	oviparous	rigid	absent	Norell et al. (2020)[49]; Wiemann et al. (2018)[57]; Laurin (2005)[3]
**Aves**	*Strix nebulosa*	oviparous	rigid	absent	Norell et al. (2020)[49]; Wiemann et al. (2018)[57]; Laurin (2005)[3]
**Aves**	*Passer domesticus*	oviparous	rigid	absent	Norell et al. (2020)[49]; Wiemann et al. (2018)[57]; Laurin

					(2005)[3]
**Dinosauria**	Microtroodontid*	oviparous	rigid	?	Norell et al. (2020)[49]
**Dinosauria**	Troodontid*	oviparous	rigid	?	Norell et al. (2020)[49]

Table S2. References used to construct the phylogeny used in our ancestral state estimation analysis. The base phylogeny reflects the phylogenomic scaffold for relationships of modern tetrapod clades, from Simões et al. (2018, fig. 2)[58].

**Clade**	**References**
Choristodera	Matsumoto et al., 2019[59]; Ezcurra, 2016[60]
Ichthyopterygia	Moon, 2019[61]
Pterosauria	Andres et al., 2014[62]
Mammalia	Zhou et al., 2013[63]
Sauropodomorpha	Otero & Pol, 2013[64]
Theropoda	Cau et al., 2017[65]
Ornithischia	Han et al., 2018[66]
Testudines	Pereira et al., 2017[67]
Squamata	Simões et al., 2018[58]; Pyron et al., 2013[68]
Alternative topological re-arrangements for extinct marine reptiles	Motani et al., 2015[78]; Scheyer et al., 2017[79]; Schoch & Sues, 2018[80]

Table S3a. The ancestral states reconstructions using the equal (equal) dating method, with mean maximum likelihood reconstructions of proportions of the four observable character states of the amalgamated character of reproduction mode and eggshell mineralisation (viviparity: membrane-shelled egg: parchment egg: rigid egg; based on six hidden character states in the SMMs) and the two states of EER (absence: presence) for several key nodes across 100 trees. Results for the amalgamated character are shown for component equal rates (CER), component symmetrical (CSYM), component all-rates-different (CARD), and equal rates (ER) evolutionary models. (ind) models assume that reproduction mode and eggshell mineralisation evolve independently, (sw) models assume a switch-on dependency. EER results are shown for equal rates (EER ER) and all-rates-different (EER ARD) evolutionary models. The green column represents the best-fitting model for most of the trees, grey columns represent models which are the best-fitting model for a smaller sample of trees (see also Table S22) or whose AIC difference relative to the best-fitting model is smaller than 2.

Character	Reproduction mode + egg shell mineralisation					
Model	equal-CER (ind)	equal-CER (sw)	equal-CSYM (ind)	equal-CSYM (sw)	equal-CARD (ind)	equal-CARD (sw)
Log-likelihood range over 100 trees (mean)	-104.426 – -101.800 (-103.220)	-105.135 – -102.185 (-103.759)	-103.598 – -100.070 (-101.928)	-103.677 – -100.195 (-102.054)	-99.064 – -89.170 (-90.013)	-90.811 – -87.742 (-89.340)
AIC range over 100 trees (mean)	207.599 – 212.852 (210.441)	208.370 – 214.269 (211.519)	208.140 – 215.197 (211.857)	208.390 – 215.353 (212.108)	194.339 – 214.128 (196.026)	191.483 – 197.623 (194.680)
Amniota	0.98:0.018:0.001:0.001	0.966:0.032:0.001:0.001	0.966:0.031:0.003:0.001	0.961:0.034:0.004:0.001	1:0:0:0	1:0:0:0
Mammalia	0.952:0.023:0.011:0.014	0.933:0.028:0.021:0.018	0.941:0.024:0.023:0.012	0.939:0.025:0.025:0.011	1:0:0:0	1:0:0:0
Reptilia	0.981:0.018:0:0	0.968:0.032:0:0	0.967:0.032:0.001:0	0.961:0.036:0.002:0	1:0:0:0	1:0:0:0
Diapsida s.l.	0.979:0.015:0.002:0.	0.965:0.028:0.003:0.	0.966:0.024:0.008:0.	0.961:0.027:0.01:0.0	1:0:0:0	1:0:0:0

	003	004	002	02		
Diapsida s.s.	0.327:0.076:0.212:0.385	0.239:0.215:0.241:0.305	0.323:0.318:0.178:0.181	0.264:0.42:0.205:0.111	1:0:0:0	1:0:0:0
Lepidosauria	0.153:0.059:0.466:0.322	0.135:0.155:0.438:0.272	0.151:0.335:0.301:0.213	0.137:0.416:0.329:0.118	1:0:0:0	1:0:0:0
Archelosauria	0.325:0.077:0.195:0.403	0.237:0.214:0.231:0.317	0.321:0.329:0.166:0.184	0.262:0.432:0.191:0.115	1:0:0:0	1:0:0:0
Archosauromorpha	0.349:0.081:0.184:0.386	0.255:0.218:0.22:0.307	0.345:0.326:0.162:0.167	0.284:0.424:0.185:0.107	1:0:0:0	1:0:0:0
Archosauria	0.047:0.154:0.234:0.565	0.025:0.278:0.35:0.347	0.046:0.505:0.241:0.207	0.027:0.605:0.265:0.103	0.011:0.961:0.013:0.014	0.007:0.666:0.324:0.003
Dinosauria	0:0.218:0.209:0.572	0:0.347:0.312:0.341	0:0.577:0.24:0.182	0:0.673:0.235:0.093	0.008:0.967:0.011:0.014	0.007:0.74:0.251:0.002
Saurischia	0:0.237:0.204:0.559	0:0.368:0.301:0.331	0:0.614:0.225:0.161	0:0.712:0.205:0.083	0.005:0.972:0.01:0.013	0.003:0.795:0.199:0.003
Theropoda	0:0.034:0.03:0.936	0:0.063:0.054:0.883	0:0.074:0.051:0.874	0:0.089:0.044:0.867	0.061:0.116:0.006:0.816	0.053:0.073:0.049:0.824

Table S3b. Continuation of Table S3a.

Character	Reproduction mode + egg shell mineralisation		EER	
Model	equal-ER (ind)	equal-ER (sw)	equal-EER ER	equal-EER ARD
Log-likelihood range over 100 trees (mean)	-104.646 – -102.291 (-103.566)	-105.495 – -103.045 (-104.391)	-28.981 – -27.703 (-28.298)	-24.673 – -23.551 (-24.101)
AIC range over 100 trees (mean)	206.582 – 211.291 (209.133)	208.089 – 212.990 (210.782)	57.407 – 59.963 (58.597)	51.102 – 53.347 (52.202)

Amniota	0.983:0.015:0.001:0.001	0.966:0.031:0.002:0.001	0.001:0.999	0:1
Mammalia	0.945:0.024:0.012:0.018	0.908:0.033:0.031:0.028	0.036:0.964	0:1
Reptilia	0.984:0.015:0:0	0.968:0.031:0:0.001	0:1	0:1
Diapsida s.l.	0.982:0.012:0.002:0.004	0.964:0.026:0.004:0.006	0:1	0:1
Diapsida s.s.	0.389:0.038:0.192:0.38	0.252:0.108:0.267:0.374	0.01:0.99	0:1
Lepidosauria	0.21:0.023:0.544:0.223	0.154:0.049:0.591:0.206	0.009:0.991	0:1
Archelosauria	0.387:0.037:0.166:0.41	0.249:0.103:0.243:0.404	0.018:0.982	0:1
Archosauromorpha	0.41:0.039:0.156:0.395	0.269:0.104:0.231:0.396	0.017:0.983	0:1
Archosauria	0.066:0.085:0.207:0.642	0.03:0.14:0.36:0.47	0.863:0.137	0.998:0.002
Dinosauria	0.001:0.134:0.186:0.679	0:0.199:0.319:0.482	0.999:0.001	0.999:0.001
Saurischia	0:0.147:0.182:0.671	0:0.213:0.31:0.477	1:0	0.999:0.001
Theropoda	0.001:0.015:0.018:0.965	0.001:0.024:0.033:0.942	1:0	0.985:0.015

Table S4a. Same as Table S3 but based on 100 trees time-scaled using the minimum branch length (mbl) dating method.

Character	Reproduction mode + egg shell mineralisation					
Model	mbl-CER (ind)	mbl-CER (sw)	mbl-CSYM (ind)	mbl-CSYM (sw)	mbl-CARD (ind)	mbl-CARD (sw)
Log-likelihood range over 100 trees (mean)	-106.267 – -100.825 (-103.303)	-106.699 – -101.120 (-103.694)	-103.929 – -98.748 (-101.293)	-104.259 – -99.096 (-101.629)	-96.629 – -92.205 (-94.395)	-96.833 – -92.075 (-94.325)
AIC range over 100 trees (mean)	205.650 – 216.533 (210.605)	206.240 – 217.397 (211.387)	205.496 – 215.858 (210.586)	206.192 – 216.517 (211.259)	200.411 – 209.259 (204.790)	200.149 – 209.666 (204.649)
Amniota	0.98:0.019:0.001:0.001	0.979:0.019:0.001:0.001	0.98:0.016:0.004:0.001	0.981:0.015:0.004:0	0.995:0.004:0.001:0	0.995:0.004:0:0
Mammalia	0.993:0.003:0.002:0.002	0.993:0.002:0.002:0.002	0.993:0.003:0.003:0.002	0.993:0.002:0.002:0.002	0.998:0.001:0.001:0.00	0.997:0.001:0.001:0.001
Reptilia	0.981:0.019:0:0	0.98:0.019:0:0	0.982:0.017:0.002:0	0.982:0.016:0.002:0	0.987:0.012:0.001:0	0.985:0.014:0.001:0
Diapsida s.l.	1:0:0:0	0.999:0:0:0	1:0:0:0	0.999:0:0:0	1:0:0:0	1:0:0:0
Diapsida s.s.	0.986:0.008:0.003:0.003	0.98:0.013:0.004:0.003	0.986:0.006:0.006:0.002	0.978:0.011:0.01:0.002	0.99:0.004:0.004:0.002	0.97:0.014:0.013:0.004
Lepidosauria	0.05:0.095:0.1:0.755	0.023:0.142:0.116:0.718	0.05:0.07:0.091:0.789	0.019:0.102:0.132:0.746	0.601:0.077:0.032:0.289	0.232:0.157:0.076:0.534
Archelosauria	0.986:0.008:0.003:0.003	0.98:0.013:0.004:0.003	0.986:0.006:0.006:0.002	0.977:0.011:0.01:0.002	0.99:0.004:0.004:0.001	0.985:0.007:0.007:0.002
Archosauromorpha	0.986:0.008:0.003:0.003	0.981:0.013:0.004:0.003	0.986:0.006:0.006:0.002	0.978:0.01:0.009:0.002	0.995:0.002:0.002:0.001	0.994:0.002:0.003:0.001
Archosauria	0.13:0.709:0.1:0.062	0.059:0.709:0.147:0.085	0.13:0.551:0.29:0.029	0.107:0.512:0.35:0.031	0.013:0.71:0.257:0.021	0.012:0.736:0.233:0.019
Dinosauria	0.012:0.846:0.094:0.	0.005:0.794:0.139:0.	0.012:0.702:0.266:0.	0.009:0.651:0.32:0.0	0.008:0.8:0.181:0.01	0.008:0.82:0.162:0.0

	049	062	019	19	1	1
Saurischia	0.003:0.875:0.085:0.038	0.001:0.828:0.123:0.048	0.003:0.759:0.225:0.013	0.001:0.718:0.267:0.013	0.005:0.848:0.14:0.007	0.003:0.866:0.125:0.006
Theropoda	0:0.002:0.002:0.995	0:0.003:0.003:0.994	0:0.001:0.001:0.998	0:0.001:0.002:0.998	0.001:0.013:0.001:0.985	0.001:0.014:0.002:0.983

Table S4b. Continuation of Table S4a.

Character	Reproduction mode + egg shell mineralisation		EER	
Model	mbl-ER (ind)	mbl-ER (sw)	mbl-EER ER	mbl-EER ARD
Log-likelihood range over 100 trees (mean)	-108.446 – -103.209 (-105.602)	-109.491 – -104.117 (-106.600)	-33.660 – -31.170 (-32.087)	-31.841 – -29.431 (-30.152)
AIC range over 100 trees (mean)	208.418 – 218.893 (213.203)	210.234 – 220.982 (215.200)	64.341 – 69.320 (66.174)	62.862 – 67.683 (64.304)
Amniota	0.922:0.074:0.002:0.002	0.923:0.072:0.002:0.002	0.002:0.998	0:1
Mammalia	0.987:0.006:0.003:0.003	0.987:0.006:0.003:0.003	0.007:0.993	0.001:0.999
Reptilia	0.926:0.073:0:0.001	0.927:0.071:0.001:0.001	0:1	0:1
Diapsida s.l.	0.999:0.001:0:0	0.999:0.001:0:0	0:1	0:1
Diapsida s.s.	0.991:0.006:0.001:0.002	0.982:0.013:0.002:0.002	0.002:0.998	0:1
Lepidosauria	0.175:0.047:0.119:0.659	0.087:0.086:0.192:0.635	0.009:0.991	0.001:0.999
Archelosauria	0.991:0.006:0.001:0.002	0.982:0.014:0.002:0.002	0.002:0.998	0.004:0.996

Archosauromorpha	0.992:0.006:0.001:0.001	0.983:0.013:0.002:0.002	0.002:0.998	0.001:0.999
Archosauria	0.168:0.691:0.096:0.045	0.065:0.695:0.155:0.086	0.864:0.136	0.997:0.003
Dinosauria	0.047:0.819:0.096:0.037	0.017:0.764:0.154:0.065	0.986:0.014	0.998:0.002
Saurischia	0.019:0.855:0.093:0.032	0.005:0.79:0.149:0.056	0.996:0.004	0.999:0.001
Theropoda	0:0.001:0:0.999	0:0.001:0.001:0.999	1:0	1:0

Table S5a. Same as Table S3 but based on 100 trees time-scaled using the fossilised birth-death (FBD) tip-dating method with the root age constrained.

Character	Reproduction mode + egg shell mineralisation					
Model	FBD-CER (ind)	FBD-CER (sw)	FBD-CSYM (ind)	FBD-CSYM (sw)	FBD-CARD (ind)	FBD-CARD (sw)
Log-likelihood range over 100 trees (mean)	-110.599 – -100.810 (-105.158)	-111.605 – -101.545 (-105.967)	-109.792 – -99.176 (-103.472)	-110.057 – -99.580 (-103.732)	-100.801 – -91.033 (-95.193)	-99.743 – -89.640 (-93.810)
AIC range over 100 trees (mean)	205.620 – 225.198 (214.316)	207.090 – 227.210 (215.934)	206.352 – 227.584 (214.944)	207.161 – 228.114 (215.464)	198.065 – 217.601 (206.386)	195.280 – 215.486 (203.619)
Amniota	0.959:0.027:0.006:0.008	0.931:0.047:0.014:0.009	0.956:0.02:0.017:0.007	0.942:0.028:0.025:0.005	1:0:0:0	1:0:0:0
Mammalia	0.929:0.036:0.017:0.018	0.901:0.04:0.035:0.024	0.927:0.029:0.027:0.017	0.921:0.032:0.033:0.015	0.99:0.005:0.003:0.002	0.994:0.002:0.002:0.001
Reptilia	0.966:0.027:0.003:0.004	0.939:0.049:0.007:0.005	0.962:0.021:0.014:0.003	0.948:0.03:0.019:0.003	1:0:0:0	1:0:0:0
Diapsida s.l.	0.965:0.024:0.005:0.006	0.937:0.045:0.01:0.009	0.962:0.018:0.015:0.005	0.947:0.027:0.022:0.004	1:0:0:0	1:0:0:0
Diapsida s.s.	0.68:0.081:0.097:0.142	0.563:0.201:0.128:0.107	0.678:0.097:0.111:0.114	0.618:0.152:0.17:0.059	0.983:0.009:0.006:0.003	0.99:0.007:0.003:0.001
Lepidosauria	0.237:0.098:0.431:0.234	0.24:0.157:0.402:0.2	0.237:0.216:0.249:0.298	0.231:0.267:0.329:0.173	0.851:0.043:0.062:0.045	0.917:0.029:0.037:0.017
Archelosauria	0.65:0.089:0.095:0.165	0.522:0.205:0.147:0.126	0.649:0.104:0.121:0.127	0.582:0.159:0.191:0.068	0.986:0.008:0.005:0.002	0.988:0.006:0.005:0.001
Archosauromorpha	0.667:0.092:0.089:0.152	0.536:0.202:0.145:0.117	0.665:0.105:0.122:0.108	0.602:0.155:0.185:0.058	0.997:0.001:0.001:0	0.999:0.001:0.001:0

Archosauria	0.156:0.306:0.187:0.351	0.089:0.332:0.399:0.179	0.156:0.304:0.331:0.209	0.112:0.34:0.477:0.071	0.026:0.642:0.275:0.056	0.022:0.549:0.405:0.023
Dinosauria	0.003:0.435:0.177:0.385	0.001:0.445:0.358:0.196	0.003:0.423:0.373:0.201	0.002:0.456:0.473:0.07	0.011:0.748:0.2:0.04	0.012:0.639:0.328:0.021
Saurischia	0:0.457:0.172:0.371	0:0.473:0.336:0.191	0:0.471:0.351:0.178	0:0.502:0.434:0.064	0.005:0.812:0.156:0.027	0.003:0.704:0.277:0.016
Theropoda	0:0.081:0.034:0.885	0:0.105:0.078:0.817	0:0.074:0.094:0.832	0:0.089:0.112:0.8	0.033:0.102:0.041:0.823	0.031:0.072:0.057:0.84

Table S5b. Continuation of Table S5a.

Character	Reproduction mode + egg shell mineralisation		EER	
Model	FBD-ER (ind)	FBD-ER (sw)	FBD-EER ER	FBD-EER ARD
Log-likelihood range over 100 trees (mean)	-111.322 – -101.592 (-105.702)	-112.308 – -102.568 (-106.847)	-33.794 – -26.468 (-29.584)	-29.876 – -22.339 (-26.085)
AIC range over 100 trees (mean)	205.184 – 224.644 (213.404)	207.137 – 226.615 (215.694)	54.936 – 69.588 (61.167)	48.678 – 63.752 (56.171)
Amniota	0.96:0.024:0.007:0.009	0.915:0.051:0.023:0.011	0.009:0.991	0:1
Mammalia	0.916:0.046:0.018:0.02	0.856:0.059:0.055:0.03	0.06:0.94	0.004:0.996
Reptilia	0.971:0.022:0.003:0.004	0.933:0.051:0.009:0.006	0.001:0.999	0:1
Diapsida s.l.	0.97:0.018:0.005:0.006	0.932:0.045:0.013:0.011	0:1	0:1
Diapsida s.s.	0.728:0.047:0.094:0	0.606:0.135:0.142:0	0.021:0.979	0:1

	.131	.117		
Lepidosauria	0.299:0.042:0.528:0.131	0.318:0.067:0.511:0.104	0.025:0.975	0.002:0.998
Archelosauria	0.695:0.051:0.091:0.163	0.554:0.133:0.164:0.149	0.068:0.932	0.004:0.996
Archosauromorpha	0.709:0.053:0.085:0.154	0.567:0.132:0.157:0.145	0.069:0.931	0:1
Archosauria	0.192:0.199:0.184:0.425	0.107:0.224:0.42:0.248	0.771:0.229	0.992:0.008
Dinosauria	0.006:0.313:0.175:0.506	0.003:0.33:0.378:0.289	0.995:0.005	0.997:0.003
Saurischia	0.001:0.332:0.171:0.496	0:0.351:0.361:0.288	1:0	0.999:0.001
Theropoda	0:0.04:0.02:0.94	0.001:0.051:0.053:0.896	1:0	0.99:0.01

Table S6a. Same as Table S3 but based on 100 trees time-scaled using the fossilised birth-death (FBD) tip-dating method with the root age and the node age of major extant clades constrained.

Character	Reproduction mode + egg shell mineralisation					
Model	FBD_c-CER (ind)	FBD_c-CER (sw)	FBD_c-CSYM (ind)	FBD_c-CSYM (sw)	FBD_c-CARD (ind)	FBD_c-CARD (sw)
Log-likelihood range over 100 trees (mean)	-110.394 – -98.848 (-104.458)	-110.788 – -99.208 (-105.094)	-108.300 – -97.776 (-102.966)	-108.509 – -98.307 (-103.205)	-100.650 – -89.847 (-94.742)	-100.859 – -88.314 (-93.476)
AIC range over 100 trees (mean)	201.695 – 224.789 (212.916)	202.416 – 225.576 (214.187)	203.552 – 224.599 (213.932)	204.614 – 225.018 (214.410)	195.695 – 217.301 (205.484)	192.629 – 217.718 (202.951)
Amniota	0.953:0.031:0.007:0.009	0.927:0.049:0.014:0.01	0.947:0.025:0.021:0.008	0.933:0.033:0.029:0.006	1:0:0:0	1:0:0:0
Mammalia	0.907:0.045:0.023:0.025	0.874:0.048:0.046:0.032	0.904:0.037:0.035:0.024	0.895:0.041:0.042:0.022	0.988:0.005:0.004:0.003	0.994:0.002:0.002:0.001
Reptilia	0.961:0.031:0.003:0.005	0.936:0.052:0.007:0.006	0.955:0.026:0.016:0.004	0.939:0.035:0.022:0.003	1:0:0:0	1:0:0:0
Diapsida s.l.	0.958:0.027:0.006:0.009	0.932:0.046:0.011:0.01	0.953:0.023:0.018:0.006	0.937:0.032:0.025:0.005	1:0:0:0	1:0:0:0
Diapsida s.s.	0.48:0.106:0.167:0.247	0.373:0.233:0.207:0.187	0.477:0.139:0.185:0.199	0.409:0.231:0.259:0.101	0.992:0.005:0.002:0.001	0.99:0.008:0.002:0
Lepidosauria	0.248:0.112:0.366:0.274	0.23:0.185:0.377:0.208	0.246:0.2:0.268:0.286	0.225:0.277:0.341:0.157	0.893:0.031:0.048:0.028	0.937:0.023:0.027:0.013
Archelosauria	0.459:0.11:0.157:0.275	0.346:0.228:0.217:0.209	0.456:0.142:0.191:0.211	0.384:0.231:0.275:0.109	0.992:0.005:0.002:0.001	0.991:0.006:0.003:0
Archosauromorpha	0.466:0.116:0.152:0.267	0.35:0.228:0.219:0.203	0.463:0.146:0.195:0.196	0.392:0.23:0.279:0.099	0.998:0.001:0.001:0	0.998:0.001:0.001:0

Archosauria	0.162:0.212:0.217:0.409	0.089:0.276:0.392:0.243	0.161:0.242:0.327:0.27	0.109:0.32:0.464:0.107	0.035:0.653:0.253:0.059	0.029:0.52:0.425:0.026
Dinosauria	0.003:0.344:0.196:0.457	0.001:0.391:0.341:0.267	0.003:0.352:0.385:0.26	0.001:0.424:0.473:0.101	0.016:0.753:0.191:0.04	0.018:0.605:0.356:0.021
Saurischia	0:0.372:0.188:0.439	0:0.422:0.317:0.26	0:0.397:0.374:0.229	0:0.466:0.442:0.092	0.007:0.82:0.143:0.03	0.004:0.675:0.303:0.018
Theropoda	0:0.058:0.031:0.91	0:0.081:0.061:0.858	0:0.059:0.089:0.852	0:0.074:0.108:0.818	0.038:0.103:0.037:0.823	0.034:0.07:0.06:0.835

Table S6b. Continuation of Table S6a.

Character	Reproduction mode + egg shell mineralisation		EER	
Model	FBD_c-ER (ind)	FBD_c-ER (sw)	FBD_c-EER ER	FBD_c-EER ARD
Log-likelihood range over 100 trees (mean)	-111.014 – -99.394 (-104.933)	-111.676 – -100.005 (-105.848)	-32.556 – -26.540 (-29.120)	-29.577 – -22.778 (-25.432)
AIC range over 100 trees (mean)	200.787 – 224.029 (211.865)	202.010 – 225.352 (213.695)	55.080 – 67.112 (60.240)	49.556 – 63.153 (54.863)
Amniota	0.955:0.026:0.008:0.01	0.916:0.05:0.022:0.012	0.011:0.989	0:1
Mammalia	0.893:0.054:0.025:0.028	0.826:0.065:0.07:0.039	0.076:0.924	0.002:0.998
Reptilia	0.967:0.025:0.004:0.005	0.934:0.051:0.008:0.007	0.001:0.999	0:1
Diapsida s.l.	0.965:0.02:0.006:0.009	0.93:0.044:0.014:0.012	0.001:0.999	0:1
Diapsida s.s.	0.55:0.06:0.165:0.2	0.428:0.148:0.231:0	0.044:0.956	0:1

	25	.193		
Lepidosauria	0.32:0.057:0.42:0.203	0.294:0.097:0.454:0.156	0.05:0.95	0.001:0.999
Archelosauria	0.524:0.061:0.151:0.264	0.391:0.14:0.239:0.23	0.096:0.904	0.003:0.997
Archosauromorpha	0.529:0.064:0.145:0.262	0.393:0.14:0.237:0.23	0.1:0.9	0:1
Archosauria	0.208:0.132:0.216:0.444	0.112:0.178:0.42:0.29	0.676:0.324	0.99:0.01
Dinosauria	0.005:0.251:0.198:0.546	0.002:0.29:0.366:0.342	0.994:0.006	0.996:0.004
Saurischia	0:0.273:0.191:0.535	0:0.314:0.345:0.34	1:0	0.998:0.002
Theropoda	0.001:0.031:0.021:0.948	0.001:0.042:0.043:0.915	1:0	0.989:0.011

Table S7a. Same as Table S3 but excluding *Mesosaurus tenuidens* from the analyses (equal dating method).

Character	Reproduction mode + egg shell mineralisation					
Model	equal-CER (ind)	equal-CER (sw)	equal-CSYM (ind)	equal-CSYM (sw)	equal-CARD (ind)	equal-CARD (sw)
Log-likelihood range over 100 trees (mean)	-103.473 – -100.318 (-101.747)	-104.145 – -100.734 (-102.272)	-102.352 – -98.716 (-100.550)	-102.447 – -98.776 (-100.701)	-90.767 – -87.916 (-88.561)	-89.402 – -86.341 (-87.946)
AIC range over 100 trees (mean)	204.636 – 210.946 (207.494)	205.467 – 212.289 (208.544)	205.432 – 212.703 (209.100)	205.552 – 212.894 (209.402)	191.832 – 197.534 (193.122)	188.682 – 194.804 (191.891)
Amniota	0.912:0.016:0.027:0.044	0.874:0.027:0.046:0.053	0.912:0.029:0.033:0.026	0.905:0.034:0.037:0.023	1:0:0:0	1:0:0:0
Mammalia	0.901:0.033:0.028:0.038	0.858:0.043:0.049:0.05	0.901:0.035:0.037:0.02	0.894:0.039:0.042:0.025	1:0:0:0	1:0:0:0
Reptilia/Diapsida s.l.	0.916:0.013:0.027:0.044	0.879:0.024:0.044:0.054	0.916:0.028:0.031:0.025	0.909:0.033:0.035:0.023	1:0:0:0	1:0:0:0
Diapsida s.s.	0.32:0.06:0.22:0.399	0.226:0.161:0.27:0.343	0.32:0.28:0.202:0.198	0.275:0.368:0.235:0.123	1:0:0:0	1:0:0:0
Lepidosauria	0.149:0.048:0.484:0.319	0.127:0.111:0.481:0.28	0.149:0.302:0.317:0.232	0.147:0.371:0.35:0.132	1:0:0:0	1:0:0:0
Archelosauria	0.319:0.061:0.201:0.419	0.225:0.16:0.257:0.359	0.319:0.289:0.192:0.201	0.273:0.378:0.223:0.127	1:0:0:0	1:0:0:0
Archosauromorpha	0.344:0.064:0.19:0.401	0.245:0.163:0.244:0.349	0.344:0.286:0.187:0.182	0.297:0.37:0.216:0.118	1:0:0:0	1:0:0:0
Archosauria	0.046:0.129:0.238:0.586	0.024:0.216:0.366:0.394	0.046:0.445:0.285:0.224	0.03:0.529:0.329:0.112	0.012:0.969:0.008:0.011	0.008:0.647:0.342:0.003
Dinosauria	0:0.19:0.213:0.596	0:0.283:0.326:0.391	0:0.511:0.292:0.196	0:0.591:0.31:0.099	0.008:0.974:0.006:0.012	0.008:0.716:0.274:0.002

Saurischia	0:0.208:0.208:0.584	0:0.303:0.315:0.382	0:0.547:0.279:0.174	0:0.627:0.283:0.089	0.005:0.978:0.005:0.011	0.003:0.77:0.224:0.003
Theropoda	0:0.029:0.029:0.942	0:0.049:0.049:0.902	0:0.066:0.059:0.875	0:0.077:0.057:0.866	0.061:0.112:0.003:0.824	0.052:0.071:0.05:0.827

Table S7b. Continuation of Table S7a.

Character	Reproduction mode + egg shell mineralisation		EER	
Model	equal-ER (ind)	equal-ER (sw)	equal-EER ER	equal-EER ARD
Log-likelihood range over 100 trees (mean)	-103.634 – -100.790 (-102.058)	-104.435 – -101.488 (-102.791)	-28.974 – -27.614 (-28.270)	-24.593 – -23.539 (-24.043)
AIC range over 100 trees (mean)	203.581 – 209.268 (206.116)	204.976 – 210.869 (207.582)	57.228 – 59.948 (58.540)	51.078 – 53.185 (52.087)
Amniota	0.912:0.014:0.027:0.048	0.844:0.027:0.056:0.072	0.01:0.99	0:1
Mammalia	0.895:0.034:0.028:0.043	0.818:0.051:0.063:0.068	0.043:0.957	0:1
Reptilia/Diapsida s.l.	0.917:0.01:0.026:0.047	0.853:0.022:0.053:0.072	0.002:0.998	0:1
Diapsida s.s.	0.375:0.032:0.199:0.393	0.226:0.084:0.286:0.404	0.01:0.99	0:1
Lepidosauria	0.201:0.02:0.554:0.225	0.135:0.039:0.616:0.21	0.01:0.99	0:1
Archelosauria	0.374:0.031:0.172:0.424	0.224:0.08:0.259:0.437	0.018:0.982	0:1
Archosauromorpha	0.399:0.033:0.161:0.407	0.245:0.081:0.245:0.428	0.018:0.982	0:1
Archosauria	0.064:0.074:0.209:0	0.027:0.115:0.356:0	0.862:0.138	0.998:0.002

	.653	.503		
Dinosauria	0.001:0.122:0.188:0.69	0:0.171:0.314:0.515	0.999:0.001	0.999:0.001
Saurischia	0:0.133:0.184:0.682	0:0.184:0.306:0.51	1:0	0.999:0.001
Theropoda	0.001:0.014:0.018:0.967	0.001:0.02:0.031:0.948	1:0	0.985:0.015

Table S8a. Same as Table S4 but excluding *Mesosaurus tenuidens* from the analyses (mbl dating method).

Model	Reproduction mode + egg shell mineralisation					
Character	mbl-CER (ind)	mbl-CER (sw)	mbl-CSYM (ind)	mbl-CSYM (sw)	mbl-CARD (ind)	mbl-CARD (sw)
Log-likelihood range over 100 trees (mean)	-105.055 – -99.614 (-101.908)	-105.558 – -99.920 (-102.300)	-102.789 – -97.712 (-99.905)	-103.151 – -98.058 (-100.254)	-96.048 – -91.098 (-93.038)	-96.129 – -90.851 (-92.977)
AIC range over 100 trees (mean)	203.229 – 214.110 (207.816)	203.841 – 215.116 (208.600)	203.425 – 213.578 (207.811)	204.115 – 214.303 (208.509)	198.195 – 208.095 (202.075)	197.702 – 208.258 (201.953)
Amniota	0.948:0.019:0.016:0.016	0.952:0.02:0.014:0.014	0.948:0.019:0.019:0.014	0.952:0.018:0.018:0.012	0.982:0.006:0.007:0.005	0.987:0.005:0.005:0.002
Mammalia	0.992:0.003:0.003:0.003	0.993:0.003:0.002:0.002	0.992:0.003:0.003:0.002	0.993:0.003:0.003:0.002	0.997:0.001:0.001:0.001	0.997:0.001:0.001:0.001
Reptilia/Diapsida s.l.	0.999:0:0:0	0.999:0.001:0:0	0.999:0:0:0	0.999:0.001:0.001:0	1:0:0:0	1:0:0:0
Diapsida s.s.	0.986:0.008:0.003:0.003	0.981:0.012:0.004:0.003	0.986:0.006:0.006:0.003	0.978:0.01:0.009:0.002	0.989:0.004:0.005:0.002	0.97:0.013:0.013:0.004
Lepidosauria	0.052:0.095:0.1:0.753	0.025:0.141:0.116:0.718	0.052:0.071:0.089:0.788	0.02:0.104:0.129:0.747	0.573:0.085:0.034:0.308	0.228:0.162:0.076:0.534
Archelosauria	0.986:0.008:0.003:0.003	0.98:0.012:0.004:0.003	0.986:0.006:0.006:0.002	0.977:0.011:0.01:0.002	0.989:0.004:0.005:0.002	0.985:0.007:0.007:0.002

Archosauromorpha	0.986:0.008:0.003:0.003	0.981:0.012:0.004:0.003	0.986:0.006:0.005:0.002	0.979:0.01:0.009:0.002	0.995:0.002:0.002:0.001	0.994:0.002:0.003:0.001
Archosauria	0.126:0.707:0.104:0.062	0.057:0.702:0.154:0.087	0.126:0.562:0.282:0.03	0.101:0.523:0.342:0.034	0.013:0.707:0.257:0.023	0.012:0.73:0.238:0.021
Dinosauria	0.012:0.841:0.099:0.049	0.005:0.785:0.146:0.064	0.012:0.712:0.256:0.02	0.009:0.66:0.31:0.021	0.008:0.797:0.182:0.013	0.008:0.815:0.166:0.011
Saurischia	0.003:0.87:0.089:0.038	0.001:0.82:0.13:0.049	0.003:0.768:0.215:0.014	0.001:0.726:0.258:0.015	0.005:0.847:0.141:0.007	0.003:0.861:0.129:0.006
Theropoda	0:0.002:0.002:0.995	0:0.003:0.003:0.994	0:0.001:0.001:0.998	0:0.001:0.001:0.998	0.001:0.013:0.001:0.985	0.001:0.015:0.002:0.982

Table S8b. Continuation of Table S8a.

Character	Reproduction mode + egg shell mineralisation		EER	
Model	mbl-ER (ind)	mbl-ER (sw)	mbl-EER ER	mbl-EER ARD
Log-likelihood range over 100 trees (mean)	-107.274 – -101.905 (-104.122)	-108.377 – -102.750 (-105.089)	-33.636 – -31.077 (-32.011)	-31.930 – -29.451 (-30.171)
AIC range over 100 trees (mean)	205.809 – 216.548 (210.243)	207.499 – 218.754 (212.178)	64.154 – 69.272 (66.023)	62.902 – 67.859 (64.342)
Amniota	0.846:0.07:0.041:0.043	0.841:0.068:0.043:0.047	0.059:0.941	0.001:0.999
Mammalia	0.986:0.007:0.004:0.004	0.986:0.007:0.004:0.004	0.008:0.992	0.001:0.999
Reptilia/Diapsida s.l.	0.999:0.001:0:0	0.998:0.001:0:0	0:1	0:1
Diapsida s.s.	0.991:0.006:0.002:0.002	0.983:0.012:0.003:0.002	0.002:0.998	0:1
Lepidosauria	0.182:0.047:0.111:0	0.1:0.08:0.186:0.63	0.011:0.989	0.001:0.999

	.659	4		
Archelosauria	0.991:0.006:0.002:0.002	0.983:0.012:0.003:0.002	0.002:0.998	0.005:0.995
Archosauromorpha	0.992:0.005:0.001:0.002	0.984:0.011:0.003:0.002	0.002:0.998	0.001:0.999
Archosauria	0.163:0.68:0.11:0.048	0.066:0.65:0.185:0.098	0.868:0.132	0.997:0.003
Dinosauria	0.047:0.803:0.111:0.04	0.017:0.722:0.186:0.075	0.986:0.014	0.998:0.002
Saurischia	0.019:0.84:0.107:0.034	0.005:0.751:0.18:0.064	0.996:0.004	0.999:0.001
Theropoda	0:0.001:0:0.999	0:0.001:0.001:0.998	1:0	1:0

Table S9a. Same as Table S5 but excluding *Mesosaurus tenuidens* from the analyses (FBD tip-dating method with root age constrained).

Character	Reproduction mode + egg shell mineralisation					
Model	FBD-CER (ind)	FBD-CER (sw)	FBD-CSYM (ind)	FBD-CSYM (sw)	FBD-CARD (ind)	FBD-CARD (sw)
Log-likelihood range over 100 trees (mean)	-108.187 – -96.898 (-103.124)	-108.875 – -97.763 (-103.866)	-106.888 – -96.045 (-101.554)	-107.159 – -96.358 (-101.846)	-101.006 – -87.558 (-93.326)	-99.709 – -86.673 (-92.002)
AIC range over 100 trees (mean)	197.796 – 220.374 (210.248)	199.527 – 221.750 (211.732)	200.090 – 221.775 (211.108)	200.716 – 222.318 (211.692)	191.116 – 218.013 (202.653)	189.346 – 215.418 (200.004)
Amniota	0.893:0.03:0.034:0.043	0.835:0.05:0.064:0.051	0.893:0.031:0.037:0.039	0.87:0.043:0.053:0.034	0.999:0:0:0	0.99:0.002:0.003:0.005
Mammalia	0.906:0.045:0.024:0.026	0.864:0.055:0.046:0.035	0.906:0.038:0.033:0.024	0.893:0.042:0.042:0.023	0.986:0.006:0.005:0.004	0.983:0.006:0.006:0.005
Reptilia/Diapsid	0.902:0.025:0.032:0.	0.846:0.044:0.058:0.	0.902:0.028:0.034:0.	0.879:0.039:0.049:0.	1:0:0:0	0.99:0.002:0.002:0.0

a s.l.	042	051	037	033		06
Diapsida s.s.	0.626:0.083:0.125:0.167	0.495:0.193:0.171:0.142	0.626:0.096:0.137:0.14	0.565:0.159:0.192:0.083	0.982:0.009:0.006:0.002	0.977:0.011:0.005:0.007
Lepidosauria	0.21:0.093:0.48:0.217	0.21:0.14:0.46:0.19	0.211:0.198:0.287:0.305	0.212:0.25:0.342:0.196	0.832:0.048:0.076:0.044	0.898:0.035:0.043:0.024
Archelosauria	0.598:0.092:0.118:0.192	0.456:0.197:0.185:0.162	0.598:0.103:0.147:0.151	0.532:0.163:0.215:0.089	0.984:0.009:0.005:0.002	0.976:0.01:0.007:0.007
Archosauromorpha	0.619:0.097:0.11:0.174	0.476:0.197:0.178:0.149	0.619:0.105:0.148:0.127	0.557:0.159:0.21:0.073	0.997:0.002:0.001:0	0.987:0.004:0.003:0.006
Archosauria	0.16:0.291:0.203:0.347	0.087:0.311:0.401:0.201	0.16:0.279:0.346:0.215	0.117:0.31:0.494:0.079	0.025:0.694:0.235:0.046	0.023:0.56:0.386:0.032
Dinosauria	0.002:0.424:0.195:0.378	0.001:0.422:0.366:0.211	0.002:0.393:0.399:0.206	0.001:0.419:0.507:0.072	0.012:0.786:0.171:0.031	0.014:0.667:0.299:0.02
Saurischia	0:0.451:0.188:0.36	0:0.453:0.343:0.203	0:0.439:0.378:0.182	0:0.471:0.465:0.065	0.005:0.845:0.128:0.022	0.003:0.739:0.243:0.015
Theropoda	0:0.067:0.028:0.904	0:0.082:0.06:0.858	0:0.067:0.077:0.856	0:0.07:0.108:0.823	0.029:0.092:0.039:0.84	0.027:0.07:0.051:0.852

Table S9b. Continuation of Table S9a.

Character	Reproduction mode + egg shell mineralisation		EER	
Model	FBD-ER (ind)	FBD-ER (sw)	FBD-EER ER	FBD-EER ARD
Log-likelihood range over 100 trees (mean)	-108.810 – -97.386 (-103.626)	-109.678 – -98.395 (-104.627)	-33.581 – -25.819 (-29.547)	-31.091 – -21.978 (-26.023)
AIC range over 100 trees (mean)	196.773 – 219.619 (209.252)	198.791 – 221.355 (211.254)	53.639 – 69.162 (61.094)	47.956 – 66.181 (56.046)
Amniota	0.891:0.024:0.039:0.046	0.8:0.05:0.089:0.062	0.023:0.977	0:1

Mammalia	0.897:0.053:0.024:0.026	0.824:0.074:0.062:0.04	0.064:0.936	0.001:0.999
Reptilia/Diapsida s.l.	0.906:0.017:0.034:0.042	0.825:0.039:0.075:0.061	0.007:0.993	0:1
Diapsida s.s.	0.67:0.047:0.126:0.157	0.52:0.128:0.197:0.156	0.025:0.975	0:1
Lepidosauria	0.264:0.039:0.571:0.125	0.27:0.058:0.569:0.103	0.025:0.975	0.001:0.999
Archelosauria	0.639:0.052:0.116:0.194	0.475:0.126:0.208:0.191	0.075:0.925	0.001:0.999
Archosauromorpha	0.658:0.055:0.106:0.181	0.494:0.127:0.195:0.184	0.076:0.924	0:1
Archosauria	0.191:0.189:0.201:0.419	0.101:0.208:0.423:0.268	0.752:0.248	0.992:0.008
Dinosauria	0.005:0.307:0.196:0.493	0.002:0.313:0.388:0.298	0.995:0.005	0.997:0.003
Saurischia	0.001:0.329:0.19:0.48	0:0.337:0.369:0.294	1:0	0.999:0.001
Theropoda	0:0.03:0.017:0.953	0:0.038:0.042:0.92	1:0	0.991:0.009

Table S10a. Same as Table S6 but excluding *Mesosaurus tenuidens* from the analyses (FBD tip-dating method with root age and node age of major extant clades constrained).

Character	Reproduction mode + egg shell mineralisation					
Model	FBD_c-CER (ind)	FBD_c-CER (sw)	FBD_c-CSYM (ind)	FBD_c-CSYM (sw)	FBD_c-CARD (ind)	FBD_c-CARD (sw)
Log-likelihood range over 100 trees (mean)	-108.64 – -97.197 (-102.628)	-109.333 – -97.526 (-103.144)	-107.434 – -96.083 (-101.055)	-107.607 – -96.398 (-101.282)	-100.597 – -88.468 (-93.464)	-100.573 – -87.301 (-91.989)
AIC range over	198.393 – 221.295	199.053 – 222.667	200.166 – 222.868	200.796 – 223.215	192.936 – 217.194	190.603 – 217.146

100 trees (mean)	(209.256)	(210.287)	(210.110)	(210.564)	(202.928)	(199.978)
Amniota	0.862:0.033:0.047:0.057	0.812:0.049:0.077:0.061	0.862:0.037:0.051:0.049	0.841:0.05:0.066:0.043	0.999:0:0:0	1:0:0:0
Mammalia	0.862:0.06:0.037:0.04	0.812:0.071:0.065:0.052	0.862:0.053:0.05:0.035	0.845:0.059:0.06:0.036	0.984:0.006:0.005:0.004	0.992:0.003:0.003:0.002
Reptilia/Diapsida s.l.	0.869:0.027:0.046:0.058	0.821:0.042:0.075:0.062	0.869:0.033:0.05:0.049	0.847:0.046:0.065:0.042	1:0:0:0	1:0:0:0
Diapsida s.s.	0.402:0.101:0.207:0.29	0.305:0.202:0.268:0.226	0.402:0.136:0.226:0.236	0.345:0.221:0.299:0.135	0.989:0.006:0.004:0.001	0.995:0.002:0.003:0
Lepidosauria	0.212:0.108:0.388:0.292	0.193:0.164:0.417:0.226	0.212:0.186:0.287:0.315	0.195:0.259:0.357:0.189	0.86:0.043:0.059:0.038	0.936:0.025:0.027:0.011
Archelosauria	0.382:0.106:0.197:0.315	0.281:0.2:0.275:0.244	0.382:0.14:0.233:0.244	0.322:0.223:0.315:0.14	0.987:0.007:0.004:0.001	0.988:0.006:0.006:0.001
Archosauromorpha	0.391:0.113:0.191:0.305	0.287:0.202:0.275:0.236	0.391:0.145:0.242:0.222	0.331:0.224:0.321:0.124	0.997:0.002:0.001:0	0.998:0.001:0.001:0
Archosauria	0.125:0.206:0.249:0.42	0.07:0.248:0.415:0.267	0.125:0.232:0.372:0.271	0.086:0.298:0.493:0.123	0.031:0.618:0.285:0.066	0.027:0.515:0.432:0.026
Dinosauria	0.002:0.317:0.232:0.449	0.001:0.345:0.373:0.282	0.002:0.324:0.434:0.24	0.001:0.386:0.503:0.109	0.016:0.713:0.225:0.046	0.018:0.594:0.366:0.022
Saurischia	0:0.345:0.225:0.43	0:0.374:0.352:0.273	0:0.368:0.427:0.205	0:0.432:0.472:0.095	0.008:0.779:0.179:0.034	0.005:0.669:0.307:0.018
Theropoda	0:0.047:0.031:0.921	0:0.06:0.055:0.885	0:0.051:0.081:0.868	0:0.062:0.09:0.849	0.032:0.094:0.039:0.835	0.03:0.066:0.051:0.854

Table S10b. Continuation of Table S10a.

Character	Reproduction mode + egg shell	EER

	mineralisation			
Model	FBD_c-ER (ind)	FBD_c-ER (sw)	FBD_c-EER ER	FBD_c-EER ARD
Log-likelihood range over 100 trees (mean)	-108.938 – -97.714 (-103.128)	-109.930 – -98.231 (-103.867)	-33.407 – -25.835 (-29.115)	-30.425 – -22.190 (-25.457)
AIC range over 100 trees (mean)	197.429 – 219.877 (208.257)	198.463 – 221.860 (209.734)	53.670 – 68.813 (60.230)	48.380 – 64.850 (54.915)
Amniota	0.861:0.027:0.052:0.06	0.778:0.048:0.102:0.072	0.031:0.969	0:1
Mammalia	0.853:0.068:0.038:0.041	0.764:0.09:0.088:0.059	0.084:0.916	0.002:0.998
Reptilia/Diapsida s.l.	0.873:0.019:0.049:0.058	0.798:0.035:0.094:0.072	0.014:0.986	0:1
Diapsida s.s.	0.466:0.054:0.21:0.27	0.344:0.119:0.301:0.236	0.061:0.939	0:1
Lepidosauria	0.279:0.051:0.451:0.219	0.245:0.079:0.504:0.172	0.064:0.936	0.002:0.998
Archelosauria	0.442:0.055:0.196:0.308	0.312:0.113:0.306:0.269	0.113:0.887	0.002:0.998
Archosauromorpha	0.449:0.059:0.188:0.304	0.318:0.115:0.3:0.268	0.117:0.883	0:1
Archosauria	0.166:0.123:0.252:0.459	0.088:0.151:0.444:0.318	0.701:0.299	0.991:0.009
Dinosauria	0.005:0.219:0.238:0.538	0.002:0.241:0.4:0.357	0.993:0.007	0.995:0.005
Saurischia	0.001:0.241:0.231:0.527	0:0.263:0.382:0.354	0.999:0.001	0.998:0.002
Theropoda	0.001:0.023:0.021:0	0.001:0.029:0.04:0.	1:0	0.99:0.01

	.956	931		

Table S11a. Same as Table S3 but excluding extinct marine reptiles from the analyses (equal dating method).

Character	Reproduction mode + egg shell mineralisation					
Model	equal-CER (ind)	equal-CER (sw)	equal-CSYM (ind)	equal-CSYM (sw)	equal-CARD (ind)	equal-CARD (sw)
Log-likelihood range over 100 trees (mean)	-83.460 – -80.440 (-82.081)	-83.736 – -80.564 (-82.275)	-81.917 – -78.214 (-80.294)	-82.004 – -78.207 (-80.323)	-67.448 – -66.112 (-66.817)	-68.485 – -64.976 (-66.704)
AIC range over 100 trees (mean)	164.880 – 170.919 (168.162)	165.128 – 171.472 (168.551)	164.428 – 171.833 (168.588)	164.415 – 172.008 (168.646)	148.225 – 150.897 (149.634)	145.953 – 152.971 (149.408)
Amniota	0.443:0.539:0.008:0.01	0.344:0.637:0.009:0.0	0.301:0.6:0.09:0.009	0.29:0.582:0.12:0.008	1:0:0:0	1:0:0:0
Mammalia	0.551:0.334:0.056:0.059	0.513:0.354:0.067:0.066	0.442:0.25:0.226:0.082	0.45:0.243:0.231:0.076	1:0:0:0	1:0:0:0
Reptilia	0.441:0.546:0.005:0.008	0.339:0.647:0.007:0.008	0.297:0.628:0.07:0.005	0.286:0.614:0.095:0.004	1:0:0:0	1:0:0:0
Diapsida s.l./s.s.	0.123:0.341:0.192:0.345	0.068:0.42:0.227:0.284	0.081:0.637:0.171:0.111	0.06:0.62:0.235:0.085	1:0:0:0	1:0:0:0
Lepidosauria	0.059:0.226:0.415:0.299	0.039:0.281:0.425:0.255	0.038:0.545:0.302:0.115	0.03:0.532:0.35:0.088	1:0:0:0	1:0:0:0
Archelosauria	0.123:0.336:0.179:0.362	0.068:0.414:0.219:0.299	0.082:0.644:0.156:0.118	0.06:0.629:0.219:0.092	1:0:0:0	1:0:0:0
Archosauromorpha	0.154:0.331:0.17:0.344	0.09:0.412:0.209:0.289	0.114:0.626:0.151:0.108	0.088:0.613:0.213:0.086	1:0:0:0	1:0:0:0
Archosauria	0.021:0.392:0.181:0.406	0.009:0.456:0.239:0.297	0.015:0.714:0.158:0.113	0.008:0.689:0.224:0.079	0.023:0.965:0.001:0.011	0.02:0.764:0.214:0.002
Dinosauria	0:0.443:0.16:0.397	0:0.505:0.209:0.285	0:0.754:0.144:0.102	0:0.728:0.201:0.071	0.016:0.972:0.001:0.011	0.023:0.812:0.163:0.002

Saurischia	0:0.459:0.156:0.385	0:0.521:0.203:0.276	0:0.776:0.133:0.091	0:0.753:0.183:0.064	0.011:0.977:0.002:0.01	0.006:0.865:0.127:0.002
Theropoda	0:0.073:0.035:0.892	0:0.088:0.046:0.865	0:0.097:0.027:0.875	0:0.095:0.035:0.87	0.105:0.11:0.002:0.783	0.095:0.079:0.032:0.793

Table S11b. Continuation of Table S11a.

Character	Reproduction mode + egg shell mineralisation		EER	
Model	equal-ER (ind)	equal-ER (sw)	equal-EER ER	equal-EER ARD
Log-likelihood range over 100 trees (mean)	-83.666 – -81.120 (-82.508)	-84.050 – -81.450 (-82.857)	-28.453 – -27.191 (-27.753)	-23.422 – -22.343 (-22.821)
AIC range over 100 trees (mean)	164.240 – 169.331 (167.015)	164.900 – 170.100 (167.714)	56.383 – 58.906 (57.505)	48.686 – 50.844 (49.643)
Amniota	0.588:0.396:0.006:0.009	0.423:0.559:0.008:0.01	0.002:0.998	0:1
Mammalia	0.668:0.273:0.027:0.032	0.582:0.342:0.038:0.038	0.044:0.956	0:1
Reptilia	0.587:0.4:0.005:0.008	0.419:0.565:0.007:0.009	0:1	0:1
Diapsida s.l./s.s.	0.204:0.164:0.217:0.415	0.091:0.229:0.287:0.393	0.016:0.984	0:1
Lepidosauria	0.115:0.073:0.577:0.234	0.058:0.097:0.64:0.205	0.014:0.986	0:1
Archelosauria	0.204:0.156:0.19:0.45	0.091:0.218:0.262:0.429	0.024:0.976	0:1
Archosauromorpha	0.235:0.154:0.179:0.432	0.114:0.217:0.248:0.421	0.024:0.976	0:1

Archosauria	0.04:0.193:0.195:0.572	0.013:0.246:0.278:0.463	0.845:0.155	0.996:0.004
Dinosauria	0:0.238:0.172:0.589	0:0.294:0.241:0.465	0.999:0.001	0.998:0.002
Saurischia	0:0.25:0.169:0.581	0:0.305:0.235:0.46	1:0	0.998:0.002
Theropoda	0.001:0.026:0.019:0.953	0.001:0.033:0.027:0.939	0.999:0.001	0.977:0.023

Table S12a. Same as Table S4 but excluding extinct marine reptiles from the analyses (mbl dating method).

Model	Reproduction mode + egg shell mineralisation					
Character	mbl-CER (ind)	mbl-CER (sw)	mbl-CSYM (ind)	mbl-CSYM (sw)	mbl-CARD (ind)	mbl-CARD (sw)
Log-likelihood range over 100 trees (mean)	-85.916 – -80.510 (-82.951)	-86.312 – -80.789 (-83.303)	-83.555 – -78.424 (-80.928)	-83.837 – -78.732 (-81.210)	-74.209 – -69.820 (-72.003)	-74.703 – -70.036 (-72.250)
AIC range over 100 trees (mean)	165.021 – 175.832 (169.903)	165.579 – 176.624 (170.607)	164.847 – 175.109 (169.856)	165.463 – 175.675 (170.420)	155.639 – 164.419 (160.006)	156.072 – 165.406 (160.499)
Amniota	0.894:0.1:0.003:0.003	0.876:0.116:0.004:0.004	0.897:0.083:0.018:0.002	0.865:0.109:0.024:0.002	0.986:0.012:0.001:0	0.983:0.015:0.002:0
Mammalia	0.99:0.003:0.003:0.003	0.991:0.003:0.003:0.003	0.99:0.003:0.003:0.003	0.991:0.003:0.003:0.003	0.998:0.001:0.001:0.001	0.997:0.001:0.001:0.001
Reptilia	0.895:0.102:0.001:0.001	0.877:0.12:0.002:0.002	0.898:0.091:0.01:0.001	0.866:0.12:0.014:0.001	0.976:0.023:0.001:0	0.97:0.029:0.001:0
Diapsida s.l./s.s.	0.876:0.081:0.022:0.021	0.841:0.098:0.033:0.028	0.878:0.055:0.052:0.016	0.817:0.084:0.08:0.02	0.995:0.002:0.002:0.001	0.986:0.007:0.006:0.002
Lepidosauria	0.061:0.094:0.098:0.747	0.027:0.136:0.116:0.722	0.061:0.069:0.09:0.78	0.021:0.099:0.128:0.752	0.841:0.031:0.013:0.115	0.597:0.084:0.04:0.279
Archelosauria	0.878:0.08:0.021:0.0	0.843:0.097:0.033:0.	0.88:0.054:0.051:0.0	0.82:0.083:0.079:0.0	0.993:0.003:0.003:0.	0.991:0.004:0.004:0.

	21	027	15	19	001	001
Archosauromorpha	0.886:0.076:0.02:0.019	0.852:0.092:0.03:0.025	0.888:0.051:0.047:0.013	0.831:0.078:0.073:0.017	0.996:0.002:0.002:0	0.995:0.002:0.002:0.001
Archosauria	0.123:0.718:0.098:0.061	0.054:0.718:0.145:0.083	0.124:0.552:0.296:0.028	0.094:0.527:0.349:0.03	0.03:0.697:0.253:0.02	0.039:0.704:0.237:0.02
Dinosauria	0.015:0.845:0.092:0.047	0.006:0.798:0.135:0.061	0.015:0.696:0.27:0.019	0.01:0.656:0.316:0.018	0.019:0.791:0.179:0.011	0.022:0.799:0.168:0.011
Saurischia	0.004:0.876:0.083:0.037	0.001:0.831:0.12:0.047	0.004:0.755:0.228:0.013	0.002:0.721:0.265:0.013	0.012:0.843:0.139:0.006	0.01:0.853:0.13:0.006
Theropoda	0:0.002:0.002:0.995	0:0.003:0.003:0.994	0:0.001:0.001:0.998	0:0.001:0.002:0.998	0.002:0.013:0.001:0.984	0.002:0.015:0.002:0.982

Table S12b. Continuation of Table S12a.

Character	Reproduction mode + egg shell mineralisation		EER	
Model	mbl-ER (ind)	mbl-ER (sw)	mbl-EER ER	mbl-EER ARD
Log-likelihood range over 100 trees (mean)	-87.234 – -82.023 (-84.400)	-88.159 – -82.822 (-85.272)	-32.912 – -30.436 (-31.354)	-30.694 – -28.294 (-29.046)
AIC range over 100 trees (mean)	166.047 – 176.467 (170.801)	167.644 – 178.318 (172.543)	62.872 – 67.824 (64.708)	60.589 – 65.388 (62.092)
Amniota	0.851:0.141:0.004:0.004	0.834:0.158:0.004:0.004	0.003:0.997	0:1
Mammalia	0.985:0.007:0.004:0.004	0.985:0.007:0.004:0.004	0.009:0.991	0.001:0.999
Reptilia	0.856:0.142:0.001:0.001	0.838:0.158:0.002:0.002	0.001:0.999	0:1
Diapsida s.l./s.s.	0.928:0.054:0.009:0	0.891:0.078:0.016:0	0.01:0.99	0:1

	.01	.015		
Lepidosauria	0.222:0.052:0.085:0.641	0.113:0.088:0.154:0.644	0.028:0.972	0.001:0.999
Archelosauria	0.933:0.05:0.008:0.009	0.897:0.074:0.015:0.014	0.01:0.99	0.004:0.996
Archosauromorpha	0.941:0.044:0.007:0.007	0.909:0.065:0.013:0.012	0.01:0.99	0.001:0.999
Archosauria	0.17:0.699:0.088:0.043	0.076:0.69:0.146:0.089	0.853:0.147	0.995:0.005
Dinosauria	0.059:0.819:0.087:0.034	0.025:0.766:0.143:0.067	0.977:0.023	0.997:0.003
Saurischia	0.027:0.86:0.084:0.029	0.009:0.799:0.136:0.056	0.993:0.007	0.998:0.002
Theropoda	0:0.001:0.001:0.998	0:0.001:0.001:0.998	1:0	1:0

Table S13a. Same as Table S5 but excluding extinct marine reptiles from the analyses (FBD tip-dating method with root age constrained).

Character	Reproduction mode + egg shell mineralisation					
Model	FBD-CER (ind)	FBD-CER (sw)	FBD-CSYM (ind)	FBD-CSYM (sw)	FBD-CARD (ind)	FBD-CARD (sw)
Log-likelihood range over 100 trees (mean)	-88.802 – -78.783 (-83.304)	-89.203 – -78.947 (-83.670)	-87.124 – -77.289 (-81.436)	-87.208 – -77.265 (-81.490)	-80.058 – -68.058 (-71.718)	-84.373 – -66.843 (-70.715)
AIC range over 100 trees (mean)	161.567 – 181.604 (170.609)	161.894 – 182.406 (171.340)	162.578 – 182.248 (170.873)	162.529 – 182.417 (170.980)	152.116 – 176.117 (159.435)	149.687 – 184.746 (157.431)
Amniota	0.449:0.41:0.063:0.078	0.335:0.514:0.08:0.071	0.428:0.274:0.232:0.066	0.363:0.318:0.269:0.05	0.999:0:0:0	0.989:0.008:0.002:0.001
Mammalia	0.733:0.151:0.057:0.	0.714:0.154:0.068:0.	0.726:0.111:0.106:0.	0.711:0.118:0.114:0.	0.996:0.002:0.001:0.	0.986:0.006:0.004:0.

	059	064	057	057	001	004
Reptilia	0.433:0.452:0.049:0.066	0.305:0.568:0.068:0.059	0.411:0.327:0.209:0.053	0.339:0.376:0.247:0.038	0.999:0.001:0:0	0.984:0.013:0.002:0.001
Diapsida s.l./s.s.	0.269:0.389:0.139:0.203	0.148:0.48:0.202:0.169	0.257:0.302:0.287:0.153	0.188:0.358:0.357:0.097	0.998:0.001:0.001:0	0.979:0.017:0.003:0.001
Lepidosauria	0.095:0.218:0.427:0.261	0.064:0.251:0.446:0.238	0.09:0.311:0.324:0.275	0.069:0.349:0.373:0.209	0.968:0.009:0.012:0.011	0.955:0.012:0.026:0.007
Archelosauria	0.267:0.37:0.133:0.231	0.143:0.447:0.215:0.195	0.255:0.291:0.285:0.169	0.184:0.343:0.363:0.109	0.997:0.002:0.001:0	0.981:0.013:0.004:0.001
Archosauromorpha	0.318:0.347:0.127:0.209	0.182:0.425:0.212:0.18	0.307:0.278:0.272:0.143	0.244:0.325:0.34:0.091	0.999:0:0:0	0.988:0.009:0.003:0.001
Archosauria	0.075:0.477:0.162:0.286	0.03:0.491:0.282:0.197	0.072:0.391:0.367:0.17	0.044:0.418:0.452:0.085	0.055:0.66:0.23:0.055	0.065:0.544:0.37:0.021
Dinosauria	0.001:0.563:0.145:0.291	0:0.564:0.239:0.197	0.001:0.483:0.359:0.157	0.001:0.503:0.417:0.08	0.026:0.761:0.172:0.04	0.041:0.639:0.302:0.019
Saurischia	0:0.578:0.142:0.28	0:0.581:0.228:0.191	0:0.532:0.328:0.14	0:0.549:0.378:0.073	0.013:0.819:0.14:0.028	0.017:0.707:0.261:0.015
Theropoda	0:0.112:0.035:0.853	0:0.123:0.056:0.821	0:0.089:0.085:0.825	0:0.097:0.096:0.807	0.07:0.099:0.039:0.792	0.072:0.068:0.049:0.811

Table S13b. Continuation of Table S13a.

Character	Reproduction mode + egg shell mineralisation		EER	
Model	FBD-ER (ind)	FBD-ER (sw)	FBD-EER ER	FBD-EER ARD
Log-likelihood range over 100 trees (mean)	-89.528 – -79.225 (-83.838)	-90.172 – -79.483 (-84.354)	-32.739 – -25.669 (-28.651)	-27.912 – -20.847 (-24.371)
AIC range over 100	160.449 – 181.057	160.965 – 182.345	53.339 – 67.478	45.693 – 59.825

trees (mean)	(169.676)	(170.708)	(59.302)	(52.742)
Amniota	0.559:0.319:0.054:0.069	0.387:0.467:0.08:0.065	0.028:0.972	0:1
Mammalia	0.787:0.139:0.036:0.038	0.747:0.156:0.054:0.043	0.079:0.921	0.004:0.996
Reptilia	0.549:0.34:0.048:0.063	0.364:0.502:0.073:0.06	0.016:0.984	0:1
Diapsida s.l./s.s.	0.375:0.265:0.15:0.209	0.202:0.371:0.237:0.19	0.052:0.948	0:1
Lepidosauria	0.166:0.118:0.555:0.162	0.119:0.143:0.608:0.13	0.046:0.954	0.002:0.998
Archelosauria	0.368:0.244:0.136:0.251	0.192:0.335:0.236:0.237	0.098:0.902	0.004:0.996
Archosauromorpha	0.413:0.229:0.126:0.232	0.231:0.319:0.223:0.228	0.098:0.902	0:1
Archosauria	0.117:0.339:0.166:0.378	0.045:0.376:0.298:0.281	0.754:0.246	0.987:0.013
Dinosauria	0.004:0.43:0.15:0.416	0.001:0.449:0.253:0.296	0.992:0.008	0.994:0.006
Saurischia	0:0.445:0.147:0.408	0:0.463:0.244:0.293	0.999:0.001	0.998:0.002
Theropoda	0.001:0.059:0.021:0.919	0.001:0.067:0.037:0.896	0.999:0.001	0.982:0.018

Table S14a. Same as Table S6 but excluding extinct marine reptiles from the analyses (FBD tip-dating method with root age and node age of major extant clades constrained).

Character	Reproduction mode + egg shell mineralisation					
Model	FBD_c-CER (ind)	FBD_c-CER (sw)	FBD_c-CSYM (ind)	FBD_c-CSYM (sw)	FBD_c-CARD (ind)	FBD_c-CARD (sw)

Log-likelihood range over 100 trees (mean)	-88.007 – -76.898 (-82.338)	-88.205 – -77.000 (-82.621)	-87.680 – -76.114 (-80.660)	-85.773 – -76.088 (-80.641)	-81.859 – -66.250 (-71.427)	-77.362 – -65.231 (-70.351)
AIC range over 100 trees (mean)	157.796 – 180.014 (168.677)	158.000 – 180.410 (169.243)	160.229 – 183.360 (169.319)	160.176 – 179.546 (169.283)	148.501 – 179.719 (158.854)	146.463 – 170.725 (156.702)
Amniota	0.363:0.477:0.071:0.089	0.287:0.546:0.087:0.08	0.335:0.327:0.262:0.075	0.283:0.366:0.297:0.053	0.995:0.005:0:0	0.999:0.001:0:0
Mammalia	0.595:0.227:0.087:0.09	0.583:0.227:0.097:0.093	0.579:0.173:0.159:0.089	0.564:0.182:0.169:0.086	0.99:0.005:0.002:0.003	0.996:0.002:0.001:0.001
Reptilia	0.343:0.526:0.056:0.076	0.255:0.604:0.074:0.066	0.314:0.389:0.237:0.06	0.256:0.434:0.272:0.038	0.994:0.006:0:0	0.99:0.009:0:0
Diapsida s.l./s.s.	0.137:0.419:0.177:0.267	0.074:0.464:0.245:0.217	0.125:0.345:0.321:0.209	0.083:0.394:0.411:0.112	0.994:0.005:0:0	0.986:0.014:0:0
Lepidosauria	0.074:0.29:0.36:0.276	0.049:0.311:0.407:0.233	0.067:0.329:0.361:0.243	0.049:0.368:0.412:0.172	0.97:0.008:0.014:0.008	0.961:0.011:0.022:0.005
Archelosauria	0.134:0.401:0.166:0.299	0.07:0.437:0.247:0.245	0.123:0.333:0.318:0.226	0.08:0.378:0.42:0.122	0.994:0.006:0:0	0.99:0.009:0.001:0
Archosauromorpha	0.151:0.393:0.165:0.291	0.08:0.426:0.254:0.24	0.14:0.328:0.317:0.214	0.098:0.37:0.421:0.112	0.995:0.005:0:0	0.997:0.003:0:0
Archosauria	0.053:0.436:0.178:0.333	0.02:0.444:0.285:0.251	0.048:0.366:0.353:0.232	0.026:0.398:0.469:0.107	0.076:0.647:0.222:0.055	0.071:0.526:0.372:0.032
Dinosauria	0.001:0.512:0.149:0.338	0:0.516:0.227:0.257	0.001:0.435:0.345:0.22	0:0.463:0.437:0.099	0.04:0.75:0.172:0.038	0.041:0.618:0.317:0.024
Saurischia	0:0.53:0.145:0.325	0:0.536:0.214:0.249	0:0.478:0.321:0.201	0:0.505:0.405:0.091	0.022:0.818:0.131:0.029	0.01:0.699:0.27:0.021
Theropoda	0:0.092:0.032:0.876	0:0.101:0.046:0.854	0:0.076:0.078:0.846	0:0.081:0.099:0.82	0.079:0.099:0.039:0.	0.068:0.072:0.053:0.

					783	807

Table S14b. Continuation of Table S14a.

Character	Reproduction mode + egg shell mineralisation		EER	
Model	FBD_c-ER (ind)	FBD_c-ER (sw)	FBD_c-EER ER	FBD_c-EER ARD
Log-likelihood range over 100 trees (mean)	-88.603 – -77.484 (-82.877)	-88.873 – -77.659 (-83.268)	-31.793 – -25.440 (-28.198)	-27.729 – -21.014 (-23.734)
AIC range over 100 trees (mean)	156.969 – 179.205 (167.753)	157.319 – 179.746 (168.536)	52.879 – 65.585 (58.396)	46.028 – 59.459 (51.468)
Amniota	0.483:0.374:0.064:0.079	0.348:0.492:0.088:0.072	0.038:0.962	0:1
Mammalia	0.677:0.208:0.055:0.059	0.634:0.227:0.075:0.064	0.104:0.896	0.002:0.998
Reptilia	0.468:0.4:0.058:0.074	0.319:0.531:0.083:0.067	0.025:0.975	0:1
Diapsida s.l./s.s.	0.231:0.28:0.206:0.283	0.116:0.343:0.299:0.241	0.093:0.907	0:1
Lepidosauria	0.144:0.168:0.466:0.222	0.089:0.191:0.543:0.177	0.092:0.908	0.001:0.999
Archelosauria	0.223:0.261:0.187:0.33	0.108:0.314:0.29:0.288	0.143:0.857	0.002:0.998
Archosauromorpha	0.24:0.255:0.18:0.325	0.119:0.307:0.286:0.288	0.148:0.852	0:1
Archosauria	0.099:0.294:0.197:0.41	0.034:0.326:0.319:0.321	0.662:0.338	0.982:0.018
Dinosauria	0.003:0.379:0.164:0	0.001:0.403:0.251:0	0.991:0.009	0.992:0.008

	.454	.345		
Saurischia	0:0.396:0.159:0.444	0:0.42:0.239:0.341	0.999:0.001	0.997:0.003
Theropoda	0.001:0.048:0.021:0.931	0.001:0.055:0.031:0.913	0.999:0.001	0.981:0.019

Table S15a. Same as Table S3 but adding extinct marine reptiles as sister taxon to Archelosauria (equal dating method).

Character	Reproduction mode + egg shell mineralisation					
Model	equal-CER (ind)	equal-CER (sw)	equal-CSYM (ind)	equal-CSYM (sw)	equal-CARD (ind)	equal-CARD (sw)
Log-likelihood range over 100 trees (mean)	-104.464 – -101.735 (-103.274)	-106.121 – -102.566 (-104.503)	-103.937 – -100.307 (-102.467)	-104.443 – -100.673 (-102.759)	-90.250 – -88.812 (-89.490)	-90.693 – -87.849 (-89.519)
AIC range over 100 trees (mean)	207.470 – 212.927 (210.549)	209.132 – 216.242 (213.006)	208.614 – 215.873 (212.933)	209.346 – 216.885 (213.517)	193.625 – 196.499 (194.979)	191.698 – 197.386 (195.039)
Amniota	0.974:0.024:0.001:0.001	0.93:0.066:0.002:0.002	0.934:0.06:0.006:0.001	0.932:0.056:0.012:0.001	1:0:0:0	1:0:0:0
Mammalia	0.945:0.027:0.013:0.015	0.899:0.046:0.034:0.021	0.915:0.035:0.034:0.016	0.911:0.036:0.041:0.012	1:0:0:0	1:0:0:0
Reptilia	0.975:0.024:0:0.001	0.931:0.067:0.001:0.001	0.934:0.062:0.003:0	0.932:0.06:0.008:0	1:0:0:0	1:0:0:0
Diapsida s.l./s.s.	0.97:0.019:0.005:0.006	0.924:0.059:0.008:0.009	0.931:0.052:0.013:0.004	0.929:0.046:0.023:0.003	1:0:0:0	1:0:0:0
Lepidosauria	0.311:0.03:0.546:0.113	0.37:0.095:0.361:0.174	0.298:0.264:0.295:0.143	0.375:0.246:0.308:0.071	1:0:0:0	1:0:0:0
Marine reptiles + Archelosauria	0.979:0.013:0.003:0.006	0.934:0.048:0.007:0.01	0.95:0.034:0.011:0.005	0.945:0.031:0.02:0.004	1:0:0:0	1:0:0:0
Archelosauria	0.892:0.015:0.014:0.078	0.803:0.067:0.06:0.07	0.867:0.071:0.024:0.038	0.836:0.079:0.061:0.023	1:0:0:0	1:0:0:0
Archosauromorpha	0.896:0.015:0.013:0.075	0.808:0.068:0.056:0.068	0.872:0.07:0.024:0.035	0.842:0.077:0.06:0.021	1:0:0:0	1:0:0:0
Archosauria	0.124:0.101:0.107:0.668	0.085:0.185:0.47:0.26	0.121:0.458:0.175:0.246	0.088:0.419:0.432:0.061	0.012:0.979:0:0.009	0.007:0.639:0.35:0.003

Dinosauria	0.001:0.151:0.105:0.744	0:0.27:0.441:0.288	0.001:0.57:0.185:0.245	0.001:0.498:0.442:0.059	0.008:0.983:0:0.009	0.008:0.711:0.279:0.002
Saurischia	0:0.163:0.104:0.733	0:0.293:0.425:0.283	0:0.61:0.169:0.221	0:0.532:0.415:0.054	0.005:0.984:0:0.011	0.003:0.769:0.225:0.003
Theropoda	0:0.018:0.013:0.969	0:0.051:0.066:0.882	0:0.071:0.036:0.893	0:0.067:0.08:0.853	0.059:0.109:0.002:0.83	0.051:0.07:0.048:0.832

Table S15b. Continuation of Table S15a.

Character	Reproduction mode + egg shell mineralisation		EER	
Model	equal-ER (ind)	equal-ER (sw)	equal-EER ER	equal-EER ARD
Log-likelihood range over 100 trees (mean)	-104.507 – -101.869 (-103.345)	-106.363 – -103.293 (-104.932)	-28.644 – -27.579 (-28.046)	-24.492 – -23.502 (-23.984)
AIC range over 100 trees (mean)	205.739 – 211.013 (208.690)	208.586 – 214.725 (211.864)	57.158 – 59.289 (58.093)	51.005 – 52.984 (51.969)
Amniota	0.977:0.021:0.001:0.001	0.933:0.062:0.003:0.002	0.001:0.999	0:1
Mammalia	0.943:0.027:0.014:0.016	0.879:0.049:0.046:0.027	0.036:0.964	0:1
Reptilia	0.978:0.021:0:0	0.934:0.063:0.001:0.001	0:1	0:1
Diapsida s.l./s.s.	0.974:0.016:0.005:0.006	0.924:0.051:0.012:0.013	0:1	0:1
Lepidosauria	0.341:0.021:0.551:0.087	0.395:0.041:0.421:0.143	0.004:0.996	0:1
Marine reptiles +	0.981:0.01:0.003:0.	0.934:0.041:0.009:0	0:1	0:1

Archelosauria	006	.015		
Archelosauria	0.891:0.011:0.013:0.085	0.77:0.045:0.075:0.11	0.087:0.913	0:1
Archosauromorpha	0.895:0.011:0.012:0.082	0.776:0.045:0.071:0.108	0.084:0.916	0:1
Archosauria	0.135:0.074:0.09:0.701	0.091:0.112:0.453:0.343	0.877:0.123	0.998:0.002
Dinosauria	0.001:0.116:0.089:0.794	0.001:0.186:0.424:0.389	0.999:0.001	0.999:0.001
Saurischia	0:0.126:0.088:0.786	0:0.202:0.412:0.386	1:0	0.999:0.001
Theropoda	0.001:0.012:0.009:0.978	0.001:0.026:0.045:0.928	1:0	0.986:0.014

Table S16a. Same as Table S3 but adding extinct marine reptiles as sister taxon to Archosauromorpha (equal dating method).

Character	Reproduction mode + egg shell mineralisation					
Model	equal-CER (ind)	equal-CER (sw)	equal-CSYM (ind)	equal-CSYM (sw)	equal-CARD (ind)	equal-CARD (sw)
Log-likelihood range over 100 trees (mean)	-103.783 – -101.364 (-102.464)	-104.843 – -101.954 (-103.340)	-102.719 – -99.417 (-101.296)	-103.036 – -99.613 (-101.498)	-96.676 – -88.281 (-88.999)	-90.214 – -87.299 (-88.892)
AIC range over 100 trees (mean)	206.728 – 211.566 (208.927)	207.907 – 213.687 (210.679)	206.835 – 213.437 (210.592)	207.226 – 214.071 (210.995)	192.562 – 209.352 (193.998)	190.598 – 196.428 (193.783)
Amniota	0.884:0.11:0.002:0.003	0.786:0.205:0.005:0.004	0.771:0.185:0.04:0.004	0.733:0.192:0.073:0.003	1:0:0:0	1:0:0:0
Mammalia	0.879:0.083:0.017:0.021	0.807:0.121:0.041:0.031	0.795:0.088:0.086:0.032	0.771:0.099:0.101:0.029	1:0:0:0	1:0:0:0
Reptilia	0.885:0.111:0.002:0.003	0.785:0.208:0.003:0.003	0.77:0.197:0.03:0.002	0.732:0.204:0.063:0.001	1:0:0:0	1:0:0:0
Diapsida s.l./s.s.	0.866:0.097:0.014:0.023	0.764:0.189:0.025:0.022	0.755:0.17:0.061:0.015	0.715:0.18:0.098:0.007	1:0:0:0	1:0:0:0
Lepidosauria	0.273:0.065:0.516:0.147	0.294:0.156:0.385:0.165	0.234:0.301:0.314:0.151	0.273:0.308:0.347:0.072	1:0:0:0	1:0:0:0
Archelosauria	0.872:0.086:0.013:0.03	0.771:0.174:0.026:0.029	0.766:0.149:0.062:0.022	0.724:0.165:0.1:0.011	1:0:0:0	1:0:0:0
Marine reptiles + Archosauromorpha	0.964:0.024:0.003:0.008	0.92:0.062:0.008:0.011	0.936:0.039:0.019:0.006	0.927:0.04:0.028:0.005	1:0:0:0	1:0:0:0
Archosauromorpha	0.959:0.024:0.005:0.012	0.914:0.061:0.011:0.013	0.933:0.038:0.022:0.007	0.924:0.039:0.031:0.005	1:0:0:0	1:0:0:0

Archosauria	0.097:0.263:0.165:0.475	0.061:0.323:0.403:0.214	0.094:0.487:0.255:0.165	0.068:0.465:0.418:0.05	0.012:0.973:0.008:0.007	0.007:0.634:0.355:0.004
Dinosauria	0.001:0.329:0.156:0.514	0:0.398:0.371:0.23	0.001:0.584:0.253:0.163	0:0.528:0.424:0.048	0.008:0.978:0.007:0.007	0.007:0.7:0.29:0.003
Saurischia	0:0.341:0.153:0.505	0:0.417:0.357:0.226	0:0.62:0.23:0.149	0:0.551:0.405:0.044	0.005:0.981:0.005:0.009	0.003:0.753:0.241:0.004
Theropoda	0:0.041:0.022:0.936	0:0.07:0.061:0.868	0:0.077:0.041:0.881	0:0.072:0.071:0.857	0.056:0.098:0.006:0.839	0.05:0.068:0.048:0.835

Table S16b. Continuation of Table S16a.

Character	Reproduction mode + egg shell mineralisation		EER	
Model	equal-ER (ind)	equal-ER (sw)	equal-EER ER	equal-EER ARD
Log-likelihood range over 100 trees (mean)	-103.885 – -101.628 (-102.620)	-105.165 – -102.744 (-103.818)	-27.973 – -26.783 (-27.308)	-24.072 – -22.994 (-23.484)
AIC range over 100 trees (mean)	205.255 – 209.769 (207.239)	207.488 – 212.330 (209.637)	55.566 – 57.945 (56.617)	49.989 – 52.144 (50.968)
Amniota	0.918:0.077:0.002:0.003	0.829:0.161:0.005:0.005	0.001:0.999	0:1
Mammalia	0.899:0.065:0.015:0.021	0.82:0.106:0.045:0.029	0.036:0.964	0:1
Reptilia	0.919:0.078:0.001:0.002	0.83:0.163:0.003:0.004	0:1	0:1
Diapsida s.l./s.s.	0.901:0.064:0.013:0.022	0.805:0.137:0.027:0.031	0:1	0:1
Lepidosauria	0.322:0.033:0.541:0	0.337:0.064:0.468:0	0.005:0.995	0:1

	.104	.131		
Archelosauria	0.905:0.054:0.01:0.03	0.811:0.12:0.026:0.043	0.002:0.998	0:1
Marine reptiles + Archosauromorpha	0.972:0.016:0.003:0.009	0.928:0.047:0.008:0.016	0.001:0.999	0:1
Archosauromorpha	0.965:0.016:0.004:0.014	0.92:0.046:0.013:0.021	0.008:0.992	0:1
Archosauria	0.108:0.18:0.147:0.566	0.065:0.21:0.425:0.3	0.901:0.099	0.998:0.002
Dinosauria	0.001:0.235:0.138:0.626	0.001:0.279:0.388:0.332	0.999:0.001	0.999:0.001
Saurischia	0:0.244:0.136:0.619	0:0.293:0.376:0.331	1:0	0.999:0.001
Theropoda	0.001:0.024:0.015:0.961	0.001:0.035:0.042:0.923	1:0	0.986:0.014

Table S17a. Same as Table S3 but adding extinct marine reptiles as sister taxon to Lepidosauria (equal dating method).

Character	Reproduction mode + egg shell mineralisation					
Model	equal-CER (ind)	equal-CER (sw)	equal-CSYM (ind)	equal-CSYM (sw)	equal-CARD (ind)	equal-CARD (sw)
Log-likelihood range over 100 trees (mean)	-103.97 – -101.14 (-102.70)	-105.288 – -101.742 (-103.642)	-103.186 – -99.521 (-101.669)	-103.533 – -99.809 (-101.883)	-89.992 – -88.464 (-89.118)	-90.293 – -87.479 (-89.121)
AIC range over 100 trees (mean)	206.280 – 211.940 (209.401)	207.484 – 214.576 (211.283)	207.041 – 214.371 (211.337)	207.618 – 215.066 (211.766)	192.928 – 195.984 (194.237)	190.957 – 196.587 (194.242)
Amniota	0.955:0.043:0.001:0.002	0.888:0.107:0.003:0.002	0.902:0.084:0.012:0.002	0.887:0.09:0.021:0.001	1:0:0:0	1:0:0:0
Mammalia	0.93:0.04:0.014:0.017	0.872:0.068:0.037:0.022	0.891:0.045:0.044:0.019	0.88:0.05:0.054:0.015	1:0:0:0	1:0:0:0
Reptilia	0.956:0.043:0.001:0.001	889:0.108:0.001:0.001	0.903:0.089:0.008:0.001	0.888:0.096:0.015:0.001	1:0:0:0	1:0:0:0
Diapsida s.l./s.s.	0.948:0.036:0.007:0.009	0.877:0.097:0.013:0.013	0.896:0.073:0.025:0.006	0.88:0.076:0.038:0.005	1:0:0:0	1:0:0:0
Marine reptiles + Lepidosauria	0.95:0.029:0.011:0.009	0.883:0.083:0.021:0.014	0.903:0.06:0.031:0.007	0.889:0.063:0.043:0.006	1:0:0:0	1:0:0:0
Lepidosauria	0.349:0.04:0.485:0.125	0.391:0.113:0.337:0.158	0.331:0.251:0.274:0.144	0.397:0.253:0.286:0.064	1:0:0:0	1:0:0:0
Archelosauria	0.867:0.041:0.026:0.066	0.767:0.109:0.071:0.053	0.82:0.092:0.049:0.039	0.782:0.108:0.086:0.023	1:0:0:0	1:0:0:0
Archosauromorpha	0.868:0.042:0.024:0.066	0.771:0.11:0.067:0.053	0.824:0.089:0.054:0.033	0.788:0.102:0.09:0.02	1:0:0:0	1:0:0:0
Archosauria	0.09:0.2:0.166:0.543	0.055:0.25:0.462:0.233	0.085:0.473:0.258:0.184	0.06:0.456:0.428:0.056	0.011:0.977:0.005:0.007	0.007:0.64:0.35:0.003

Dinosauria	0.001:0.259:0.156:0.584	0:0.328:0.423:0.249	0.001:0.57:0.252:0.178	0:0.527:0.42:0.053	0.008:0.982:0.004:0.007	0.007:0.713:0.277:0.002
Saurischia	0:0.271:0.154:0.575	0:0.348:0.407:0.245	0:0.613:0.226:0.161	0:0.561:0.39:0.048	0.005:0.984:0.003:0.009	0.003:0.771:0.223:0.003
Theropoda	0.001:0.032:0.02:0.948	0:0.058:0.064:0.878	0.001:0.072:0.044:0.884	0:0.069:0.07:0.861	0.056:0.098:0.002:0.844	0.05:0.069:0.047:0.834

Table S17b. Continuation of Table S17a.

Character	Reproduction mode + egg shell mineralisation		EER	
Model	equal-ER (ind)	equal-ER (sw)	equal-EER ER	equal-EER ARD
Log-likelihood range over 100 trees (mean)	-104.000 – -101.343 (-102.808)	-105.529 – -102.412 (-104.053)	-27.711 – -26.848 (-27.280)	-24.043 – -23.076 (-23.557)
AIC range over 100 trees (mean)	204.685 – 210.000 (207.616)	206.824 – 213.057 (210.105)	55.697 – 57.423 (56.559)	50.152 – 52.087 (51.113)
Amniota	0.964:0.033:0.001:0.002	0.902:0.092:0.004:0.002	0.001:0.999	0:1
Mammalia	0.932:0.036:0.014:0.019	0.861:0.065:0.048:0.025	0.036:0.964	0:1
Reptilia	0.965:0.033:0.001:0.00	0.904:0.093:0.002:0.002	0:1	0:1
Diapsida s.l./s.s.	0.957:0.027:0.006:0.009	0.889:0.078:0.017:0.017	0.001:0.999	0:1
Marine reptiles + Lepidosauria	0.96:0.021:0.011:0.008	0.895:0.064:0.025:0.016	0:1	0:1
Lepidosauria	0.389:0.024:0.495:0	0.436:0.052:0.385:0	0.004:0.996	0:1

	.092	.126		
Archelosauria	0.873:0.029:0.024:0.074	0.758:0.079:0.091:0.071	0.088:0.912	0:1
Archosauromorpha	0.873:0.03:0.023:0.075	0.761:0.08:0.085:0.073	0.089:0.911	0:1
Archosauria	0.098:0.146:0.146:0.609	0.057:0.17:0.479:0.294	0.908:0.092	0.998:0.002
Dinosauria	0.001:0.196:0.137:0.666	0:0.242:0.435:0.322	0.999:0.001	0.999:0.001
Saurischia	0:0.206:0.135:0.659	0:0.257:0.422:0.321	1:0	0.999:0.001
Theropoda	0.001:0.02:0.014:0.965	0.001:0.032:0.047:0.92	1:0	0.987:0.013

Table S18a. Same as Table S4 but adding extinct marine reptiles as sister taxon to Archelosauria (mbl dating method).

Character	Reproduction mode + egg shell mineralisation					
Model	mbl-CER (ind)	mbl-CER (sw)	mbl-CSYM (ind)	mbl-CSYM (sw)	mbl-CARD (ind)	mbl-CARD (sw)
Log-likelihood range over 100 trees (mean)	-105.769 – -101.330 (-103.370)	-106.187 – -101.622 (-103.744)	-103.692 – -99.315 (-101.315)	-104.065 – -99.619 (-101.650)	-96.846 – -92.738 (-94.498)	-96.849 – -92.567 (-94.394)
AIC range over 100 trees (mean)	206.659 – 215.538 (210.740)	207.244 – 216.373 (211.488)	206.630 – 215.385 (210.630)	207.238 – 216.131 (211.300)	201.476 – 209.692 (204.997)	201.134 – 209.697 (204.788)
Amniota	0.979:0.019:0.001:0.001	0.979:0.02:0.001:0.001	0.98:0.016:0.004:0.001	0.98:0.016:0.004:0	0.994:0.005:0.001:0	0.995:0.004:0.001:0
Mammalia	0.993:0.003:0.002:0.002	0.993:0.002:0.002:0.002	0.993:0.003:0.003:0.002	0.993:0.002:0.002:0.002	0.997:0.001:0.001:0.001	0.997:0.001:0.001:0.001
Reptilia	0.98:0.019:0:0	0.98:0.02:0:0	0.981:0.017:0.002:0	0.981:0.017:0.002:0	0.986:0.013:0.001:0	0.985:0.014:0.001:0
Diapsida s.l./s.s.	0.997:0.002:0.001:0.001	0.997:0.002:0.001:0	0.997:0.001:0.001:0	0.997:0.002:0.002:0	0.999:0:0:0	0.997:0.001:0.001:0
Lepidosauria	0.047:0.095:0.101:0.757	0.022:0.14:0.117:0.721	0.047:0.069:0.089:0.796	0.018:0.101:0.128:0.753	0.551:0.091:0.035:0.323	0.226:0.164:0.075:0.536
Marine reptiles + Archelosauria	1:0:0:0	0.999:0:0:0	1:0:0:0	0.999:0:0:0	1:0:0:0	1:0:0:0
Archelosauria	0.995:0.003:0.001:0.001	0.994:0.004:0.001:0.001	0.995:0.002:0.002:0.001	0.994:0.003:0.003:0.001	0.989:0.005:0.005:0.002	0.985:0.007:0.007:0.002
Archosauromorpha	0.995:0.003:0.001:0.001	0.994:0.004:0.001:0.001	0.995:0.002:0.002:0.001	0.994:0.003:0.002:0.001	0.995:0.002:0.002:0.001	0.994:0.002:0.003:0.001
Archosauria	0.123:0.709:0.106:0.062	0.055:0.706:0.155:0.084	0.124:0.558:0.291:0.027	0.101:0.517:0.351:0.031	0.012:0.703:0.264:0.02	0.012:0.724:0.246:0.018
Dinosauria	0.011:0.84:0.1:0.049	0.005:0.787:0.147:0.	0.011:0.705:0.266:0.	0.008:0.653:0.32:0.0	0.008:0.794:0.187:0.	0.008:0.809:0.173:0.

		061	018	19	011	009
Saurischia	0.002:0.868:0.091:0.038	0.001:0.821:0.131:0.047	0.003:0.761:0.224:0.013	0.001:0.719:0.267:0.013	0.005:0.843:0.145:0.006	0.003:0.857:0.135:0.005
Theropoda	0:0.002:0.002:0.995	0:0.003:0.003:0.994	0:0.001:0.001:0.998	0:0.001:0.002:0.998	0.001:0.014:0.001:0.985	0:0.015:0.002:0.983

Table S18b. Continuation of Table S18a.

Character	Reproduction mode + egg shell mineralisation		EER	
Model	mbl-ER (ind)	mbl-ER (sw)	mbl-EER ER	mbl-EER ARD
Log-likelihood range over 100 trees (mean)	-108.304 – -103.531 (-105.699)	-109.286 – -104.470 (-106.677)	-33.563 – -31.140 (-32.142)	-31.671 – -29.541 (-30.282)
AIC range over 100 trees (mean)	209.061 – 218.609 (213.398)	210.941 – 220.572 (215.353)	64.281 – 69.126 (66.284)	63.082 – 67.343 (64.563)
Amniota	0.923:0.072:0.002:0.002	0.923:0.072:0.002:0.002	0.002:0.998	0:1
Mammalia	0.987:0.006:0.003:0.003	0.987:0.006:0.003:0.003	0.007:0.993	0.001:0.999
Reptilia	0.928:0.071:0:0.001	0.928:0.071:0.001:0.001	0:1	0:1
Diapsida s.l./s.s.	0.994:0.004:0.001:0.001	0.993:0.006:0.001:0.001	0:1	0:1
Lepidosauria	0.165:0.045:0.121:0.669	0.087:0.081:0.194:0.638	0.011:0.989	0.001:0.999
Marine reptiles + Archelosauria	0.999:0.001:0:0	0.999:0.001:0:0	0:1	0:1
Archelosauria	0.994:0.004:0.001:0	0.991:0.006:0.002:0	0.005:0.995	0.005:0.995

	.001	.001		
Archosauromorpha	0.994:0.004:0.001:0.001	0.991:0.006:0.002:0.001	0.004:0.996	0.001:0.999
Archosauria	0.157:0.691:0.109:0.044	0.06:0.687:0.169:0.084	0.873:0.127	0.997:0.003
Dinosauria	0.043:0.811:0.11:0.036	0.015:0.752:0.17:0.064	0.987:0.013	0.998:0.002
Saurischia	0.017:0.845:0.107:0.031	0.004:0.777:0.164:0.055	0.997:0.003	0.999:0.001
Theropoda	0:0.001:0:0.999	0:0.001:0.001:0.999	1:0	1:0

Table S19a. Same as Table S4 but adding extinct marine reptiles as sister taxon to Archosauromorpha (mbl dating method).

Character	Reproduction mode + egg shell mineralisation					
Model	mbl-CER (ind)	mbl-CER (sw)	mbl-CSYM (ind)	mbl-CSYM (sw)	mbl-CARD (ind)	mbl-CARD (sw)
Log-likelihood range over 100 trees (mean)	-105.655 – -101.007 (-103.211)	-106.058 – -101.316 (-103.573)	-103.555 – -99.090 (-101.160)	-103.917 – -99.434 (-101.484)	-96.893 – -92.633 (-94.371)	-97.057 – -92.389 (-94.232)
AIC range over 100 trees (mean)	206.013 – 215.311 (210.422)	206.632 – 216.116 (211.145)	206.180 – 215.111 (210.320)	206.869 – 215.835 (210.969)	201.267 – 209.785 (204.743)	200.779 – 210.114 (204.464)
Amniota	0.975:0.023:0.001:0.001	0.973:0.025:0.001:0.001	0.975:0.02:0.004:0.001	0.973:0.021:0.005:0.001	0.994:0.005:0.001:0	0.995:0.004:0.001:0
Mammalia	0.993:0.003:0.002:0.002	0.993:0.002:0.002:0.002	0.993:0.003:0.003:0.002	0.993:0.002:0.002:0.002	0.997:0.001:0.001:0.001	0.997:0.001:0.001:0.001
Reptilia	0.976:0.023:0:0	0.974:0.025:0:0	0.977:0.021:0.002:0	0.974:0.023:0.003:0	0.986:0.014:0.001:0	0.984:0.016:0.001:0
Diapsida s.l./s.s.	0.99:0.006:0.002:0.002	0.989:0.007:0.002:0.001	0.99:0.004:0.004:0.001	0.986:0.007:0.007:0.001	0.997:0.001:0.001:0.001	0.992:0.004:0.003:0
Lepidosauria	0.045:0.095:0.1:0.759	0.021:0.139:0.117:0.723	0.045:0.069:0.088:0.797	0.017:0.102:0.127:0.754	0.536:0.091:0.037:0.336	0.217:0.161:0.077:0.544
Archelosauria	0.992:0.005:0.002:0.002	0.991:0.006:0.002:0.001	0.993:0.003:0.003:0.001	0.989:0.005:0.005:0.001	0.998:0.001:0.001:0	0.997:0.002:0.001:0
Marine reptiles + Archosauromorpha	0.999:0:0:0	0.999:0:0:0	0.999:0:0:0	0.999:0:0:0	1:0:0:0	1:0:0:0
Archosauromorpha	0.998:0.001:0:0	0.998:0.001:0:0.001	0.998:0.001:0.001:0	0.998:0.001:0.001:0	0.995:0.002:0.002:0.001	0.994:0.002:0.002:0.001
Archosauria	0.122:0.711:0.106:0.	0.056:0.709:0.155:0.	0.122:0.563:0.289:0.	0.101:0.522:0.348:0.	0.012:0.707:0.259:0.	0.011:0.731:0.238:0.

	061	081	026	029	022	02
Dinosauria	0.011:0.841:0.101:0.048	0.005:0.789:0.147:0.059	0.011:0.709:0.263:0.017	0.008:0.658:0.316:0.018	0.008:0.797:0.183:0.012	0.008:0.816:0.166:0.011
Saurischia	0.002:0.869:0.092:0.037	0.001:0.822:0.131:0.046	0.002:0.764:0.221:0.012	0.001:0.723:0.263:0.012	0.005:0.846:0.142:0.007	0.003:0.862:0.129:0.006
Theropoda	0:0.002:0.002:0.996	0:0.003:0.003:0.994	0:0.001:0.001:0.998	0:0.001:0.001:0.998	0.001:0.013:0.001:0.985	0:0.014:0.002:0.983

Table S19b. Continuation of Table S19a.

Character	Reproduction mode + egg shell mineralisation		EER	
Model	mbl-ER (ind)	mbl-ER (sw)	mbl-EER ER	mbl-EER ARD
Log-likelihood range over 100 trees (mean)	-108.047 – -103.406 (-105.532)	-109.003 – -104.297 (-106.509)	-33.502 – -31.138 (-32.009)	-31.745 – -29.401 (-30.145)
AIC range over 100 trees (mean)	208.811 – 218.093 (213.065)	210.594 – 220.007 (215.017)	64.277 – 69.003 (66.017)	62.802 – 67.491 (64.290)
Amniota	0.921:0.074:0.002:0.002	0.916:0.079:0.002:0.002	0.002:0.998	0:1
Mammalia	0.987:0.006:0.003:0.003	0.987:0.006:0.003:0.003	0.007:0.993	0.001:0.999
Reptilia	0.925:0.074:0:0.001	0.92:0.078:0.001:0.001	0:1	0:1
Diapsida s.l./s.s.	0.987:0.01:0.001:0.002	0.98:0.016:0.003:0.002	0:1	0:1
Lepidosauria	0.158:0.044:0.125:0.673	0.083:0.081:0.199:0.637	0.01:0.99	0.001:0.999
Archelosauria	0.992:0.006:0.001:0	0.986:0.011:0.002:0	0:1	0.001:0.999

	.001	.001		
Marine reptiles + Archosauromorpha	0.999:0.001:0:0	0.998:0.001:0:0	0:1	0:1
Archosauromorpha	0.997:0.002:0.001:0.001	0.995:0.003:0.001:0.001	0.002:0.998	0.001:0.999
Archosauria	0.155:0.697:0.109:0.04	0.059:0.696:0.167:0.077	0.874:0.126	0.997:0.003
Dinosauria	0.041:0.816:0.11:0.033	0.014:0.759:0.167:0.059	0.987:0.013	0.998:0.002
Saurischia	0.016:0.849:0.106:0.029	0.004:0.783:0.161:0.051	0.997:0.003	0.999:0.001
Theropoda	0:0.001:0:0.999	0:0.001:0:0.999	1:0	1:0

Table S20a. Same as Table S4 but adding extinct marine reptiles as sister taxon to Lepidosauria (mbl dating method).

Character	Reproduction mode + egg shell mineralisation					
Model	mbl-CER (ind)	mbl-CER (sw)	mbl-CSYM (ind)	mbl-CSYM (sw)	mbl-CARD (ind)	mbl-CARD (sw)
Log-likelihood range over 100 trees (mean)	-105.665 – -101.108 (-103.237)	-105.931 – -101.487 (-103.622)	-103.176 – -99.313 (-101.220)	-103.537 – -99.621 (-101.562)	-96.405 – -92.846 (-94.415)	-96.407 – -92.624 (-94.288)
AIC range over 100 trees (mean)	206.217 – 215.331 (210.474)	206.973 – 215.862 (211.245)	206.626 – 214.352 (210.440)	207.242 – 215.073 (211.124)	201.692 – 208.810 (204.831)	201.249 – 208.814 (204.575)
Amniota	0.98:0.018:0.001:0.001	0.98:0.019:0.001:0.001	0.981:0.015:0.003:0.001	0.981:0.015:0.004:0	0.995:0.005:0.001:0	0.996:0.004:0:0
Mammalia	0.993:0.003:0.002:0.002	0.993:0.002:0.002:0.002	0.993:0.003:0.003:0.002	0.993:0.002:0.002:0.002	0.997:0.001:0.001:0.001	0.997:0.001:0.001:0.001
Reptilia	0.982:0.018:0:0	0.981:0.019:0:0	0.982:0.016:0.002:0	0.983:0.016:0.002:0	0.987:0.013:0.001:0	0.986:0.014:0.001:0
Diapsida s.l./s.s.	0.999:0:0:0	0.999:0:0:0	0.999:0:0:0	0.999:0:0:0	1:0:0:0	1:0:0:0
Marine reptiles + Lepidosauria	1:0:0:0	0.999:0:0:0	1:0:0:0	0.999:0:0:0	0.999:0:0:0	0.998:0.001:0.001:0.001
Lepidosauria	0.046:0.095:0.1:0.759	0.022:0.14:0.116:0.722	0.046:0.069:0.089:0.797	0.017:0.101:0.129:0.753	0.542:0.089:0.037:0.332	0.215:0.161:0.078:0.546
Archelosauria	0.994:0.003:0.001:0.001	0.993:0.004:0.001:0.001	0.994:0.002:0.002:0.001	0.993:0.003:0.003:0.001	0.989:0.005:0.005:0.002	0.984:0.007:0.007:0.002
Archosauromorpha	0.995:0.003:0.001:0.001	0.993:0.004:0.001:0.001	0.995:0.002:0.002:0.001	0.993:0.003:0.003:0.001	0.995:0.002:0.002:0.001	0.994:0.003:0.003:0.001
Archosauria	0.126:0.71:0.103:0.061	0.056:0.71:0.151:0.083	0.126:0.562:0.285:0.028	0.102:0.519:0.347:0.031	0.012:0.715:0.25:0.023	0.012:0.741:0.227:0.021
Dinosauria	0.011:0.843:0.097:0.048	0.005:0.791:0.144:0.061	0.011:0.711:0.259:0.018	0.009:0.657:0.316:0.019	0.008:0.804:0.176:0.012	0.008:0.823:0.159:0.011

Saurischia	0.003:0.872:0.088:0.037	0.001:0.824:0.128:0.047	0.003:0.767:0.217:0.013	0.001:0.724:0.262:0.013	0.005:0.852:0.136:0.007	0.003:0.868:0.123:0.006
Theropoda	0:0.002:0.002:0.995	0:0.003:0.003:0.994	0:0.001:0.001:0.998	0:0.001:0.002:0.998	0.001:0.012:0.001:0.986	0:0.014:0.002:0.984

Table S20b. Continuation of Table S20a.

Character	Reproduction mode + egg shell mineralisation		EER	
Model	mbl-ER (ind)	mbl-ER (sw)	mbl-EER ER	mbl-EER ARD
Log-likelihood range over 100 trees (mean)	-108.191 – -103.511 (-105.551)	-109.061 – -104.481 (-106.542)	-33.233 – -31.272 (-32.127)	-31.491 – -29.578 (-30.247)
AIC range over 100 trees (mean)	209.022 – 218.382 (213.101)	210.961 – 220.121 (215.083)	64.544 – 68.466 (66.254)	63.155 – 66.981 (64.494)
Amniota	0.925:0.071:0.002:0.002	0.925:0.07:0.002:0.002	0.002:0.998	0:1
Mammalia	0.987:0.006:0.003:0.003	0.987:0.006:0.003:0.003	0.007:0.993	0.001:0.999
Reptilia	0.929:0.07:0:0.001	0.929:0.069:0.001:0.001	0:1	0:1
Diapsida s.l./s.s.	0.999:0.001:0:0	0.998:0.001:0:0	0:1	0:1
Marine reptiles + Lepidosauria	0.999:0.001:0:0	0.999:0.001:0:0	0:1	0:1
Lepidosauria	0.16:0.045:0.123:0.673	0.082:0.082:0.194:0.641	0.01:0.99	0.001:0.999
Archelosauria	0.993:0.005:0.001:0.001	0.989:0.007:0.002:0.002	0.005:0.995	0.005:0.995
Archosauromorpha	0.993:0.004:0.001:0.001	0.99:0.007:0.002:0.002	0.005:0.995	0.001:0.999

Archosauria	0.161:0.696:0.101:0.043	0.06:0.699:0.158:0.083	0.87:0.13	0.997:0.003
Dinosauria	0.044:0.819:0.101:0.035	0.015:0.764:0.158:0.063	0.987:0.013	0.998:0.002
Saurischia	0.017:0.854:0.098:0.031	0.004:0.789:0.152:0.054	0.997:0.003	0.999:0.001
Theropoda	0:0.001:0:0.999	0:0.001:0.001:0.999	1:0	1:0

Table S21. Parsimony-based ancestral state reconstruction of the four observable character states of the amalgamated character of reproduction mode and egg shell mineralisation (viviparity: membrane-shelled egg: parchment egg: rigid egg; based on six hidden character states) and the two states of EER (absence: presence) for several key nodes. Results for the amalgamated character are shown for the simple model (based on ACCTRAN), which allows all character transitions, and the structured models (based on MPR) which assume that reproduction mode and egg shell mineralisation evolve independently (Ind model) or a switch-on dependency (Sw model). EER results are based on a (simple) ACCTRAN approach.

Character	Reproduction mode + egg shell mineralisation			EER
Model	Simple model	Ind model	Sw model	ACCTRAN
Amniota	1:0:0:0	0.75:0.25:0:0	0.5:0.5:0:0	0:1
Mammalia	1:0:0:0	0.5:0.5:0:0	0.5:0.5:0:0	0:1
Reptilia	1:0:0:0	0.75:0.25:0:0	0.5:0.5:0:0	0:1
Diapsida s.l.	1:0:0:0	0.5:0.17:0.17:0.17	0.25:0.25:0.25:0.25	0:1
Diapsida s.s.	1:0:0:0	0.25:0.25:0.25:0.25	0:0.33:0.33:0.33	0:1
Lepidosauria	0:0:1:0	0:0:1:0	0:0:1:0	0:1
Archelosauria	1:0:0:0	0.33:0:0.33:0.33	0:0:0.5:0.5	0:1
Archosauromorpha	1:0:0:0	0.33:0:0.33:0.33	0:0:0.5:0.5	0:1
Archosauria	0:0:0:1	0:0:0:1	0:0:0:1	1:0
Dinosauria	0:0:0:1	0:0:0:1	0:0:0:1	1:0
Saurischia	0:0:0:1	0:0:0:1	0:0:0:1	1:0
Theropoda	0:0:0:1	0:0:0:1	0:0:0:1	1:0

Table S22. Same as Table S21 but excluding *Mesosaurus tenuidens* from the analyses (parsimony-based ancestral state reconstruction).

Character	Reproduction mode + egg shell mineralisation			EER
Model	Simple model	Ind model	Sw model	ACCTRAN
Amniota	1:0:0:0	0.5:0.17:0.17:0.17	0.25:0.25:0.25:0.25	0:1
Mammalia	1:0:0:0	0.25:0.25:0.25:0.25	0.25:0.25:0.25:0.25	0:1
Reptilia/Diapsida s.l.	1:0:0:0	0.5:0.17:0.17:0.17	0.25:0.25:0.25:0.25	0:1
Diapsida s.s.	1:0:0:0	0.25:0.25:0.25:0.25	0:0.33:0.33:0.33	0:1
Lepidosauria	0:0:1:0	0:0:1:0	0:0:1:0	0:1
Archelosauria	1:0:0:0	0.33:0:0.33:0.33	0:0:0.5:0.5	0:1
Archosauromorpha	1:0:0:0	0.33:0:0.33:0.33	0:0:0.5:0.5	0:1
Archosauria	0:0:0:1	0:0:0:1	0:0:0:1	1:0
Dinosauria	0:0:0:1	0:0:0:1	0:0:0:1	1:0
Saurischia	0:0:0:1	0:0:0:1	0:0:0:1	1:0
Theropoda	0:0:0:1	0:0:0:1	0:0:0:1	1:0

Table S23. Same as Table S21 but excluding extinct marine reptiles from the analyses (parsimony-based ancestral state reconstruction).

Character	Reproduction mode + egg shell mineralisation			EER
Model	Simple model	Ind model	Sw model	ACCTRAN
Amniota	1:0:0:0	0:1:0:0	0:1:0:0	0:1
Mammalia	1:0:0:0	0:1:0:0	0:1:0:0	0:1
Reptilia	1:0:0:0	0:1:0:0	0:1:0:0	0:1
Diapsida s.l./s.s.	1:0:0:0	0:0.33:0.33:0.33	0:0.33:0.33:0.33	0:1
Lepidosauria	0:0:1:0	0:0:1:0	0:0:1:0	0:1
Archelosauria	1:0:0:0	0:0:0.5:0.5	0:0:0.5:0.5	0:1
Archosauromorpha	1:0:0:0	0.33:0:0.33:0.33	0:0:0.5:0.5	0:1
Archosauria	0:0:0:1	0:0:0:1	0:0:0:1	1:0
Dinosauria	0:0:0:1	0:0:0:1	0:0:0:1	1:0
Saurischia	0:0:0:1	0:0:0:1	0:0:0:1	1:0
Theropoda	0:0:0:1	0:0:0:1	0:0:0:1	1:0

Table S24. Same as Table S21 but adding extinct marine reptiles as sister taxon to Archelosauria (parsimony-based ancestral state reconstruction).

Character	Reproduction mode + egg shell mineralisation			EER
Model	Simple model	Ind model	Sw model	ACCTRAN
Amniota	1:0:0:0	0.67:0.33:0:0	0:1:0:0	0:1
Mammalia	1:0:0:0	0.5:0.5:0:0	0:1:0:0	0:1
Reptilia	1:0:0:0	0.67:0.33:0:0	0:1:0:0	0:1
Diapsida s.l./s.s.	1:0:0:0	0.25:0.25:0.25:0.25	0:0.33:0.33:0.33	0:1
Lepidosauria	0:0:1:0	0:0:1:0	0:0:1:0	0:1
Marine reptiles + Archelosauria	1:0:0:0	0.5:0.17:0.17:0.17	0:0.33:0.33:0.33	0:1
Archelosauria	1:0:0:0	0.33:0:0.33:0.33	0:0:0.5:0.5	0:1
Archosauromorpha	1:0:0:0	0.33:0:0.33:0.33	0:0:0.5:0.5	0:1
Archosauria	0:0:0:1	0:0:0:1	0:0:0:1	1:0
Dinosauria	0:0:0:1	0:0:0:1	0:0:0:1	1:0
Saurischia	0:0:0:1	0:0:0:1	0:0:0:1	1:0
Theropoda	0:0:0:1	0:0:0:1	0:0:0:1	1:0

Table S25. Same as Table S21 but adding extinct marine reptiles as sister taxon to Archosauromorpha (parsimony-based ancestral state reconstruction).

Character	Reproduction mode + egg shell mineralisation			EER
Model	Simple model	Ind model	Sw model	ACCTRAN
Amniota	1:0:0:0	0:1:0:0	0:1:0:0	0:1
Mammalia	1:0:0:0	0:1:0:0	0:1:0:0	0:1
Reptilia	1:0:0:0	0:1:0:0	0:1:0:0	0:1
Diapsida s.l./s.s.	1:0:0:0	0:0.33:0.33:0.33	0:0.33:0.33:0.33	0:1
Lepidosauria	0:0:1:0	0:0:1:0	0:0:1:0	0:1
Archelosauria	1:0:0:0	0:0:0:1	0:0:0.5:0.5	0:1
Marine reptiles + Archosauromorpha	1:0:0:0	1:0:0:0	0.5:0:0.25:0.25	0:1
Archosauromorpha	1:0:0:0	1:0:0:0	0.5:0:0.25:0.25	0:1
Archosauria	0:0:0:1	0:0:0:1	0:0:0.5:0.5	1:0
Dinosauria	0:0:0:1	0:0:0:1	0:0:0:1	1:0
Saurischia	0:0:0:1	0:0:0:1	0:0:0:1	1:0
Theropoda	0:0:0:1	0:0:0:1	0:0:0:1	1:0

Table S26. Same as Table S21 but adding extinct marine reptiles as sister taxon to Lepidosauria (parsimony-based ancestral state reconstruction).

Character	Reproduction mode + egg shell mineralisation			EER
Model	Simple model	Ind model	Sw model	ACCTRAN
Amniota	1:0:0:0	0.75:0.25:0:0	0:1:0:0	0:1
Mammalia	1:0:0:0	0.5:0.5:0:0	0:1:0:0	0:1
Reptilia	1:0:0:0	0.75:0.25:0:0	0:1:0:0	0:1
Diapsida s.l./s.s.	1:0:0:0	0.5:0.17:0.17:0.17	0:0.33:0.33:0.33	0:1
Marine reptiles + Lepidosauria	1:0:0:0	0.25:0.25:0.25:0.25	0:0.33:0.33:0.33	0:1
Lepidosauria	0:0:1:0	0:0:1:0	0:0:1:0	0:1
Archelosauria	1:0:0:0	0.33:0:0.33:0.33	0:0:0.5:0.5	0:1
Archosauromorpha	1:0:0:0	0.33:0:0.33:0.33	0:0:0.5:0.5	0:1
Archosauria	0:0:0:1	0:0:0:1	0:0:0:1	1:0
Dinosauria	0:0:0:1	0:0:0:1	0:0:0:1	1:0
Saurischia	0:0:0:1	0:0:0:1	0:0:0:1	1:0
Theropoda	0:0:0:1	0:0:0:1	0:0:0:1	1:0

Table S27. Best-fitting models (lowest AIC score) for the maximum likelihood based ancestral state reconstructions across 100 trees for all time-scaling methods (see also Table S1), including alternative topologies which exclude *Mesosaurus tenuidens* (noMeso), exclude extinct marine reptiles (noMar), or modify the position of extinct marine reptiles as sister taxon to Archelosauria, Archosauromorpha (Archosaurom.), or Lepidosauria.

Character	Reproduction mode + egg shell mineralisation								EER	
Model	ER (ind)	ER (sw)	CER (ind)	CER (sw)	CSYM (ind)	CSYM (sw)	CARD (ind)	CARD (sw)	ER	ARD
Equal	0	0	0	0	0	0	25	75	0	100
MBL	0	0	0	0	0	0	29	71	0	100
FBD (root only)	0	0	0	0	0	0	2	98	0	100
FBD (sev. nodes)	0	0	0	0	0	0	4	96	0	100
Equal noMeso	0	0	0	0	0	0	17	83	0	100
MBL noMeso	0	0	0	0	0	0	30	70	0	100
FBD (root only) noMeso	0	0	0	0	0	0	5	95	0	100
FBD (sev. nodes) noMeso	1	0	0	0	0	0	3	96	0	100
Equal noMar	0	0	0	0	0	0	41	59	0	100
MBL noMar	0	0	0	0	0	0	98	2	0	100
FBD (root only) noMar	0	0	0	0	0	0	11	89	0	100
FBD (sev. nodes) noMar	0	0	0	0	0	0	12	88	0	100
Equal (Archelosauria)	0	0	0	0	0	0	47	53	0	100
Equal (Archosaurom.)	0	0	0	0	0	0	46	54	0	100
Equal (Lepidosauria)	0	0	0	0	0	0	47	53	0	100
MBL (Archelosauria)	0	0	0	0	0	0	21	79	0	100
MBL (Archosaurom.)	0	0	0	0	0	0	18	82	0	100
MBL	0	0	0	0	0	0	13	87	0	100

(Lepidosauria)										

Table S28a. The ancestral states reconstructions using the equal (equal) dating method for 100 input trees, with mean Bayesian reconstructions of proportions of the four observable character states of the amalgamated character of reproduction mode and eggshell (a–c, e–g) mineralisation (viviparity: membrane-shelled egg: parchment egg: rigid egg; based on six hidden character states in the SMMs) and the two states of EER (absence: presence; d) for several key nodes across three MCMC chains per model. SMM results are presented for both unconstrained node analyses (a–c) and analyses with nodes of Lepidosauria and Squamata fixed to a non-viviparous state (e–g). Results for the amalgamated character are shown for component equal rates (CER), component symmetrical (CSYM), component all-rates-different (CARD), and equal rates (ER) evolutionary models. (ind) models assume that reproduction mode and eggshell mineralisation evolve independently, (sw) models assume a switch-on dependency. (hom) models assume homogeneous evolutionary rates across all branches, (het) models assume heterogeneous evolutionary rates which are allowed to differ between individual branches. EER results are shown for equal rates (EER ER) and all-rates-different (EER ARD) evolutionary models. The green column represents the best-fitting model, grey columns represent models whose log BF difference relative to the best-fitting model is smaller than 2 and whose log BF difference to the simple comparison models (ER (sw.hom) for SMM, EER ER (hom) for EER) exceeds 2.

Character	Reproduction mode + egg shell mineralisation					
Model	equal-CER (ind.hom)	equal-CER (ind.het)	equal-CER (sw.hom)	equal-CER (sw.het)	equal-CSYM (ind.hom)	equal-CSYM (ind.het)
Mean log marginal likelihood	-70.401	-69.84	-71.877	-71.196	-71.728	-71.147
Mean log Bayes Factor	3.068	4.191	0.115	1.477	0.413	1.577
Amniota	0.982:0.016:0.001:0.001	0.979:0.018:0.001:0.002	0.967:0.03:0.002:0.001	0.964:0.032:0.002:0.002	0.982:0.016:0.001:0.001	0.979:0.018:0.001:0.002
Mammali	0.725:0.144	0.724:0.145	0.723:0.145	0.719:0.146	0.723:0.163	0.721:0.164

a	:0.065:0.065	:0.066:0.066	:0.066:0.066	:0.068:0.068	:0.056:0.058	:0.054:0.061
Reptilia	0.978:0.02:0:0.001	0.977:0.021:0.001:0.001	0.977:0.021:0.001:0.001	0.975:0.023:0.001:0.001	0.981:0.018:0:0.001	0.98:0.019:0.001:0.001
Diapsida s.l.	0.852:0.014:0.047:0.088	0.877:0.011:0.036:0.076	0.832:0.015:0.064:0.089	0.859:0.013:0.048:0.08	0.855:0.012:0.052:0.08	0.88:0.01:0.04:0.07
Diapsida s.s.	0.06:0.052:0.297:0.591	0.156:0.043:0.247:0.555	0.054:0.048:0.376:0.522	0.148:0.041:0.309:0.503	0.068:0.044:0.35:0.538	0.161:0.035:0.282:0.522
Lepidosauria	0.057:0.035:0.85:0.059	0.081:0.043:0.815:0.06	0.121:0.029:0.805:0.045	0.16:0.036:0.755:0.049	0.062:0.031:0.84:0.066	0.086:0.034:0.82:0.06
Archelosauria	0.356:0.058:0.027:0.56	0.41:0.042:0.023:0.525	0.251:0.067:0.043:0.639	0.351:0.051:0.038:0.56	0.365:0.041:0.087:0.506	0.411:0.032:0.055:0.502
Archosauromorpha	0.904:0.041:0.015:0.039	0.854:0.045:0.018:0.082	0.85:0.061:0.03:0.06	0.795:0.064:0.033:0.108	0.903:0.025:0.028:0.045	0.853:0.028:0.031:0.088
Archosauria	0.002:0.456:0.045:0.497	0.005:0.344:0.052:0.599	0.002:0.467:0.045:0.485	0.005:0.363:0.057:0.574	0.002:0.269:0.196:0.533	0.005:0.218:0.145:0.631
Dinosauria	0.001:0.674:0.061:0.264	0.002:0.557:0.07:0.37	0.001:0.683:0.06:0.256	0.002:0.566:0.075:0.356	0.001:0.42:0.205:0.374	0.003:0.365:0.157:0.476
Saurischia	0.001:0.629:0.137:0.234	0.002:0.526:0.146:0.327	0.001:0.645:0.133:0.222	0.001:0.54:0.147:0.312	0.001:0.394:0.243:0.362	0.002:0.346:0.197:0.455
Theropoda	0.013:0.014:0.014:0.96	0.012:0.012:0.012:0.964	0.017:0.015:0.015:0.954	0.016:0.014:0.014:0.956	0.014:0.013:0.015:0.958	0.013:0.011:0.014:0.962

Table S28b. Continuation of Table S28a.

Character	Reproduction mode + egg shell mineralisation					
Model	equal-CSYM (sw.hom)	equal-CSYM (sw.het)	equal-CARD (ind.hom)	equal-CARD (ind.het)	equal-CARD (sw.hom)	equal-CARD (sw.het)
Mean log marginal likelihoo	-72.401	-71.85	-63.124	-61.44	-64.107	-62.386

d						
Mean log Bayes Factor	-0.932	0.17	17.622	20.989	15.655	19.098
Amniota	0.969:0.027:0.003:0.001	0.965:0.03:0.003:0.002	1:0:0:0	1:0:0:0	1:0:0:0	1:0:0:0
Mammalia	0.716:0.176:0.061:0.046	0.719:0.168:0.058:0.055	1:0:0:0	1:0:0:0	1:0:0:0	1:0:0:0
Reptilia	0.981:0.017:0.001:0	0.978:0.021:0.001:0.001	1:0:0:0	1:0:0:0	1:0:0:0	1:0:0:0
Diapsida s.l.	0.854:0.012:0.068:0.065	0.866:0.012:0.053:0.07	1:0:0:0	1:0:0:0	1:0:0:0	1:0:0:0
Diapsida s.s.	0.101:0.033:0.509:0.356	0.169:0.038:0.367:0.426	1:0:0:0	1:0:0:0	1:0:0:0	1:0:0:0
Lepidosauria	0.15:0.028:0.755:0.066	0.161:0.033:0.751:0.055	1:0:0:0	1:0:0:0	0.999:0:0:0	0.999:0:0:0
Archelosauria	0.28:0.034:0.232:0.454	0.361:0.035:0.122:0.481	1:0:0:0	1:0:0:0	1:0:0:0	1:0:0:0
Archosauromorpha	0.871:0.023:0.054:0.052	0.804:0.033:0.056:0.107	1:0:0:0	1:0:0:0	1:0:0:0	1:0:0:0
Archosauria	0.002:0.17:0.427:0.401	0.006:0.177:0.28:0.537	0.012:0.945:0.032:0.011	0.039:0.893:0.049:0.019	0.01:0.748:0.239:0.004	0.129:0.578:0.288:0.005
Dinosauria	0.002:0.283:0.434:0.281	0.003:0.305:0.29:0.402	0.008:0.955:0.03:0.007	0.027:0.917:0.044:0.012	0.011:0.763:0.223:0.003	0.118:0.621:0.257:0.004
Saurischia	0.001:0.276:0.447:0.276	0.001:0.294:0.316:0.388	0.005:0.954:0.034:0.006	0.019:0.933:0.037:0.01	0.003:0.734:0.26:0.002	0.055:0.689:0.253:0.003
Theropoda	0.021:0.008:0.017:0.954	0.018:0.01:0.015:0.957	0.061:0.062:0.052:0.825	0.1:0.073:0.061:0.767	0.062:0.058:0.056:0.824	0.196:0.111:0.08:0.613

Table S28c. Continuation of Table S28b.

Character	Reproduction mode + egg shell mineralisation			
Model	equal-ER (ind.hom)	equal-ER (ind.het)	equal-ER (sw.hom)	equal-ER (sw.het)
Mean log marginal likelihood	-71.244	-70.69	-71.935	-71.36
Mean log Bayes Factor	1.383	2.491	0	1.15
Amniota	0.981:0.016:0.001:0.001	0.98:0.017:0.001:0.002	0.967:0.029:0.002:0.002	0.962:0.032:0.003:0.003
Mammalia	0.724:0.142:0.067:0.067	0.722:0.144:0.067:0.067	0.72:0.143:0.068:0.068	0.713:0.145:0.071:0.071
Reptilia	0.978:0.021:0:0.001	0.978:0.02:0.001:0.001	0.978:0.021:0.001:0.001	0.974:0.024:0.001:0.001
Diapsida s.l.	0.854:0.014:0.046:0.085	0.882:0.011:0.035:0.072	0.842:0.015:0.061:0.082	0.861:0.013:0.049:0.077
Diapsida s.s.	0.064:0.055:0.297:0.583	0.158:0.042:0.249:0.551	0.07:0.053:0.376:0.502	0.156:0.041:0.315:0.488
Lepidosauria	0.06:0.037:0.84:0.063	0.086:0.044:0.808:0.061	0.142:0.033:0.77:0.054	0.167:0.036:0.747:0.05
Archelosauria	0.362:0.061:0.028:0.549	0.411:0.043:0.023:0.522	0.267:0.075:0.05:0.608	0.356:0.054:0.041:0.55
Archosauromorpha	0.903:0.043:0.016:0.039	0.853:0.048:0.02:0.079	0.848:0.063:0.032:0.056	0.795:0.067:0.036:0.102
Archosauria	0.002:0.47:0.047:0.48	0.005:0.356:0.051:0.587	0.002:0.488:0.053:0.457	0.006:0.376:0.062:0.557
Dinosauria	0.001:0.679:0.063:0.257	0.002:0.56:0.07:0.368	0.002:0.686:0.068:0.244	0.002:0.572:0.081:0.345
Saurischia	0.001:0.633:0.138:0.228	0.002:0.531:0.142:0.325	0.001:0.641:0.141:0.218	0.001:0.538:0.156:0.304
Theropoda	0.013:0.014:0.014:0.958	0.012:0.012:0.012:0.964	0.019:0.017:0.017:0.947	0.017:0.014:0.014:0.955

Table S28d. Continuation of Table S28c.

Character	EER			
Model	equal-EER ER (hom)	equal-EER ER (het)	equal-EER ARD (hom)	equal-EER ARD (het)
Mean log marginal likelihood	-31.636	-31.605	-27.741	-27.522
Mean log Bayes Factor	0	0.063	7.791	8.23

Amniota	0.002:0.998	0.003:0.997	0:1	0:1
Mammalia	0.271:0.729	0.276:0.724	0.012:0.988	0.011:0.989
Reptilia	0:1	0:1	0:1	0:1
Diapsida s.l.	0.005:0.995	0.009:0.991	0:1	0:1
Diapsida s.s.	0.181:0.819	0.185:0.815	0.008:0.992	0.007:0.993
Lepidosauria	0.08:0.92	0.107:0.893	0.003:0.997	0.004:0.996
Archelosauria	0.652:0.348	0.519:0.481	0.03:0.97	0.021:0.979
Archosauromorpha	0.088:0.912	0.096:0.904	0.004:0.996	0.004:0.996
Archosauria	0.998:0.002	0.997:0.003	0.997:0.003	0.973:0.027
Dinosauria	0.999:0.001	0.999:0.001	0.998:0.002	0.981:0.019
Saurischia	0.999:0.001	0.999:0.001	0.999:0.001	0.986:0.014
Theropoda	0.989:0.011	0.99:0.01	0.981:0.019	0.933:0.067

Table S28e. Continuation of Table S28d. Nodes of Lepidosauria and Squamata fixed to a non-viviparous state.

Character	Reproduction mode + egg shell mineralisation					
Model	equal-CER (ind.hom)	equal-CER (ind.het)	equal-CER (sw.hom)	equal-CER (sw.het)	equal-CSYM (ind.hom)	equal-CSYM (ind.het)
Mean log marginal likelihood	-70.714	-70.178	-72.129	-71.47	-72.059	-71.504
Mean log Bayes Factor	3.046	4.119	0.217	1.535	0.356	1.467
Amniota	0.977:0.021:0.001:0.001	0.974:0.023:0.001:0.002	0.959:0.038:0.001:0.002	0.959:0.037:0.002:0.002	0.977:0.021:0.001:0.001	0.973:0.024:0.001:0.002
Mammalia	0.728:0.145:0.064:0.064	0.727:0.148:0.063:0.063	0.726:0.147:0.063:0.063	0.727:0.146:0.063:0.063	0.726:0.165:0.052:0.057	0.723:0.168:0.051:0.058
Reptilia	0.972:0.026:0.001:0.001	0.971:0.028:0.001:0.001	0.97:0.028:0.001:0.001	0.97:0.028:0.001:0.001	0.975:0.023:0.001:0.001	0.974:0.024:0.001:0.001
Diapsida s.l.	0.808:0.018:0.061:0.114	0.833:0.015:0.051:0.101	0.786:0.019:0.081:0.114	0.822:0.016:0.061:0.101	0.808:0.016:0.069:0.108	0.839:0.013:0.055:0.093
Diapsida s.s.	0.02:0.049:0.31:0.621	0.067:0.04:0.284:0.609	0.012:0.047:0.383:0.558	0.045:0.043:0.333:0.58	0.022:0.04:0.358:0.58	0.075:0.037:0.313:0.575
Lepidosa	0:0.032:0.9	0:0.038:0.9	0:0.03:0.92	0:0.04:0.90	0:0.028:0.9	0:0.033:0.9

uria	14:0.054	08:0.054	2:0.047	7:0.054	14:0.058	12:0.055
Archelosauria	0.344:0.053:0.024:0.579	0.353:0.04:0.022:0.585	0.234:0.061:0.037:0.668	0.285:0.049:0.034:0.632	0.35:0.036:0.078:0.537	0.362:0.031:0.056:0.551
Archosauromorpha	0.905:0.04:0.015:0.04	0.833:0.051:0.021:0.094	0.849:0.06:0.028:0.063	0.774:0.07:0.036:0.12	0.904:0.024:0.025:0.047	0.837:0.031:0.033:0.1
Archosauria	0.002:0.447:0.04:0.511	0.003:0.329:0.046:0.621	0.002:0.45:0.04:0.509	0.004:0.347:0.048:0.601	0.002:0.264:0.17:0.565	0.004:0.202:0.146:0.648
Dinosauria	0.001:0.675:0.057:0.268	0.002:0.548:0.069:0.381	0.001:0.679:0.055:0.265	0.002:0.561:0.068:0.37	0.001:0.426:0.181:0.392	0.002:0.355:0.158:0.484
Saurischia	0.001:0.628:0.133:0.238	0.001:0.52:0.146:0.332	0:0.643:0.128:0.229	0.001:0.536:0.142:0.321	0.001:0.398:0.222:0.379	0.002:0.336:0.2:0.462
Theropoda	0.011:0.012:0.012:0.964	0.011:0.012:0.012:0.966	0.014:0.013:0.013:0.96	0.014:0.012:0.012:0.962	0.012:0.011:0.013:0.964	0.012:0.011:0.013:0.964

Table S28f. Continuation of Table S28e.

Character	Reproduction mode + egg shell mineralisation					
Model	equal-CSYM (sw.hom)	equal-CSYM (sw.het)	equal-CARD (ind.hom)	equal-CARD (ind.het)	equal-CARD (sw.hom)	equal-CARD (sw.het)
Mean log marginal likelihood	-72.801	-72.227	-71.509	-70.584	-70.964	-70.076
Mean log Bayes Factor	-1.127	0.021	1.457	3.306	2.547	4.322
Amniota	0.957:0.04:0.002:0.002	0.956:0.039:0.002:0.003	0.88:0.12:0:0	0.944:0.055:0:0	0.953:0.047:0:0	0.973:0.026:0:0
Mammalia	0.723:0.175:0.053:0.05	0.721:0.171:0.052:0.056	0.719:0.243:0.019:0.019	0.807:0.16:0.016:0.017	0.851:0.119:0.015:0.014	0.895:0.084:0.01:0.01
Reptilia	0.972:0.026:0.001:0.001	0.972:0.026:0.001:0.001	0.838:0.161:0:0	0.924:0.075:0:0	0.868:0.132:0:0	0.938:0.061:0:0

Diapsida s.l.	0.788:0.018:0.097:0.097	0.823:0.015:0.07:0.092	0.818:0.143:0.018:0.022	0.909:0.061:0.012:0.019	0.849:0.129:0.013:0.01	0.931:0.054:0.007:0.007
Diapsida s.s.	0.015:0.041:0.487:0.457	0.047:0.037:0.387:0.529	0.191:0.624:0.08:0.104	0.489:0.343:0.065:0.103	0.155:0.74:0.065:0.041	0.534:0.378:0.046:0.041
Lepidosauria	0:0.03:0.909:0.061	0:0.032:0.914:0.054	0:0.111:0.718:0.171	0:0.16:0.68:0.16	0:0.138:0.754:0.108	0:0.184:0.712:0.105
Archelosauria	0.253:0.039:0.175:0.534	0.289:0.034:0.104:0.573	0.515:0.387:0.017:0.081	0.701:0.201:0.013:0.085	0.387:0.555:0.018:0.041	0.706:0.245:0.011:0.038
Archosauromorpha	0.862:0.026:0.042:0.069	0.785:0.035:0.052:0.128	0.87:0.117:0.004:0.009	0.895:0.082:0.005:0.018	0.864:0.128:0.004:0.005	0.894:0.093:0.005:0.009
Archosauria	0.002:0.19:0.305:0.503	0.004:0.168:0.208:0.619	0.006:0.893:0.019:0.082	0.024:0.849:0.025:0.101	0.006:0.951:0.011:0.032	0.063:0.873:0.019:0.045
Dinosauria	0.001:0.316:0.314:0.368	0.002:0.3:0.219:0.479	0.004:0.914:0.021:0.061	0.016:0.882:0.025:0.078	0.006:0.958:0.012:0.024	0.049:0.899:0.019:0.034
Saurischia	0.001:0.303:0.335:0.361	0.001:0.288:0.249:0.462	0.002:0.906:0.033:0.059	0.012:0.879:0.035:0.075	0.002:0.956:0.019:0.023	0.014:0.929:0.025:0.032
Theropoda	0.016:0.01:0.014:0.959	0.015:0.01:0.013:0.963	0.031:0.031:0.017:0.921	0.068:0.047:0.019:0.866	0.038:0.052:0.017:0.892	0.096:0.064:0.018:0.821

Table S28g. Continuation of Table S28f.

Character	Reproduction mode + egg shell mineralisation			
Model	equal-ER (ind.hom)	equal-ER (ind.het)	equal-ER (sw.hom)	equal-ER (sw.het)
Mean log marginal likelihood	-71.568	-71.037	-72.237	-71.668
Mean log Bayes Factor	1.338	2.4	0	1.138
Amniota	0.977:0.021:0.001:0.001	0.972:0.024:0.001:0.002	0.958:0.038:0.002:0.002	0.959:0.037:0.002:0.003
Mammalia	0.726:0.144:0.065:0.065	0.726:0.145:0.064:0.064	0.724:0.145:0.065:0.065	0.724:0.146:0.065:0.065
Reptilia	0.971:0.027:0.001	0.969:0.028:0.001	0.969:0.029:0.001	0.97:0.028:0.001:

	:0.001	:0.002	:0.001	0.001
Diapsida s.l.	0.809:0.018:0.061:0.112	0.834:0.015:0.051:0.1	0.789:0.02:0.081:0.11	0.822:0.016:0.063:0.098
Diapsida s.s.	0.022:0.053:0.31:0.616	0.075:0.042:0.285:0.598	0.014:0.05:0.391:0.544	0.045:0.042:0.347:0.566
Lepidosauria	0:0.035:0.905:0.06	0:0.039:0.906:0.055	0:0.035:0.909:0.056	0:0.038:0.908:0.053
Archelosauria	0.352:0.055:0.025:0.568	0.366:0.042:0.022:0.569	0.245:0.066:0.043:0.647	0.282:0.051:0.036:0.632
Archosauromorpha	0.904:0.041:0.015:0.04	0.831:0.053:0.021:0.095	0.847:0.062:0.031:0.06	0.773:0.071:0.037:0.12
Archosauria	0.002:0.452:0.043:0.503	0.004:0.338:0.047:0.611	0.002:0.465:0.045:0.488	0.004:0.356:0.051:0.589
Dinosauria	0.001:0.675:0.059:0.265	0.002:0.554:0.067:0.377	0.001:0.682:0.061:0.255	0.002:0.566:0.072:0.361
Saurischia	0.001:0.629:0.135:0.235	0.002:0.525:0.143:0.331	0.001:0.637:0.136:0.227	0.001:0.534:0.147:0.318
Theropoda	0.012:0.013:0.013:0.962	0.011:0.011:0.011:0.966	0.016:0.014:0.014:0.956	0.016:0.013:0.013:0.959

Table S29a. Same as Table S28 but based on 100 trees time-scaled using the minimum branch length (mbl) dating method.

Character	Reproduction mode + egg shell mineralisation					
Model	mbl-CER (ind.hom)	mbl-CER (ind.het)	mbl-CER (sw.hom)	mbl-CER (sw.het)	mbl-CSYM (ind.hom)	mbl-CSYM (ind.het)
Mean log marginal likelihood	-71.142	-70.157	-71.807	-70.594	-73.033	-71.920
Mean log Bayes Factor	2.472	4.442	1.141	3.566	-1.311	0.915
Amniota	0.915:0.079:0.003:0.003	0.913:0.078:0.005:0.005	0.909:0.084:0.003:0.004	0.906:0.083:0.005:0.006	0.919:0.074:0.003:0.003	0.918:0.071:0.005:0.005
Mammalia	0.983:0.008:0.005:0.005	0.977:0.011:0.006:0.006	0.983:0.008:0.005:0.005	0.975:0.011:0.007:0.007	0.983:0.007:0.005:0.004	0.977:0.01:0.006:0.006
Reptilia	0.91:0.088:0.001:0.001	0.91:0.087:0.002:0.002	0.914:0.083:0.001:0.001	0.911:0.084:0.002:0.002	0.915:0.083:0.001:0.001	0.915:0.08:0.002:0.002
Diapsida s.l.	0.998:0.001:0:0	0.997:0.001:0.001:0.001	0.998:0.001:0.001:0.001	0.996:0.002:0.001:0.001	0.998:0.001:0:0	0.997:0.001:0.001:0.001
Diapsida s.s.	0.859:0.083:0.028:0.03	0.863:0.069:0.032:0.036	0.807:0.1:0.051:0.042	0.829:0.079:0.048:0.044	0.842:0.088:0.036:0.033	0.847:0.071:0.041:0.041
Lepidosauria	0.142:0.046:0.168:0.644	0.21:0.066:0.221:0.503	0.119:0.052:0.209:0.621	0.207:0.073:0.249:0.471	0.135:0.048:0.163:0.655	0.193:0.077:0.211:0.519
Archelosauria	0.916:0.055:0.015:0.014	0.912:0.05:0.018:0.021	0.887:0.066:0.027:0.02	0.89:0.058:0.027:0.025	0.912:0.055:0.018:0.015	0.909:0.048:0.022:0.022
Archosauromorpha	0.955:0.031:0.008:0.006	0.954:0.029:0.01:0.008	0.949:0.032:0.013:0.007	0.948:0.03:0.013:0.009	0.955:0.03:0.009:0.006	0.953:0.028:0.011:0.008
Archosauria	0.017:0.853:0.087:0.043	0.033:0.78:0.111:0.075	0.018:0.842:0.088:0.052	0.034:0.764:0.113:0.089	0.016:0.834:0.108:0.042	0.032:0.748:0.142:0.079
Dinosauria	0.01:0.883:0.072:0.035	0.019:0.83:0.091:0.061	0.012:0.879:0.071:0.03	0.019:0.82:0.091:0.07	0.01:0.871:0.086:0.033	0.018:0.806:0.114:0.06

			9			2
Saurischia	0.006:0.882:0.079:0.033	0.012:0.834:0.097:0.058	0.005:0.876:0.081:0.038	0.009:0.822:0.101:0.069	0.006:0.871:0.092:0.03	0.012:0.811:0.119:0.058
Theropoda	0.001:0.001:0.001:0.997	0.001:0.001:0.001:0.997	0.001:0.001:0.001:0.997	0.001:0.001:0.001:0.997	0.001:0.001:0.002:0.996	0.001:0.001:0.002:0.996

Table S29b. Continuation of Table S29a.

Character	Reproduction mode + egg shell mineralisation					
Model	mbl-CSYM (sw.hom)	mbl-CSYM (sw.het)	mbl-CARD (ind.hom)	mbl-CARD (ind.het)	mbl-CARD (sw.hom)	mbl-CARD (sw.het)
Mean log marginal likelihood	-73.354	-72.010	-67.929	-65.734	-70.276	-67.711
Mean log Bayes Factor	-1.954	0.736	8.898	13.288	4.203	9.333
Amniota	0.92:0.074:0.004:0.003	0.916:0.072:0.006:0.006	0.999:0.001:0:0	1:0:0:0	0.972:0.027:0:0	0.995:0.005:0:0
Mammalia	0.983:0.008:0.005:0.005	0.975:0.011:0.007:0.007	1:0:0:0	1:0:0:0	0.996:0.003:0.001:0	0.999:0.001:0:0
Reptilia	0.923:0.074:0.001:0.001	0.92:0.075:0.003:0.002	0.998:0.001:0:0	1:0:0:0	0.95:0.05:0:0	0.99:0.01:0:0
Diapsida s.l.	0.998:0.001:0.001:0.001	0.996:0.001:0.001:0.001	1:0:0:0	1:0:0:0	0.999:0.001:0:0	1:0:0:0
Diapsida s.s.	0.779:0.108:0.065:0.048	0.806:0.084:0.059:0.051	0.998:0.001:0:0	0.999:0.001:0:0	0.924:0.068:0.005:0.003	0.987:0.012:0.001:0
Lepidosauria	0.105:0.058:0.201:0.637	0.181:0.09:0.225:0.504	0.989:0.001:0.003:0.007	0.996:0:0.001:0.002	0.677:0.021:0.078:0.224	0.933:0.007:0.018:0.042
Archelosauria	0.881:0.065:0.032:0.022	0.885:0.055:0.032:0.028	0.999:0.001:0:0	0.999:0:0:0	0.962:0.034:0.003:0.001	0.993:0.007:0:0
Archosauromorpha	0.951:0.029:0.013:0.00	0.948:0.027:0.014:0.01	0.999:0:0:0	1:0:0:0	0.984:0.014:0.001:0	0.997:0.003:0:0

	7					
Archosauria	0.016:0.815:0.117:0.052	0.031:0.723:0.15:0.096	0.019:0.888:0.085:0.008	0.058:0.827:0.095:0.021	0.047:0.882:0.063:0.008	0.192:0.719:0.073:0.015
Dinosauria	0.011:0.862:0.09:0.037	0.017:0.793:0.118:0.072	0.012:0.915:0.067:0.005	0.038:0.872:0.076:0.014	0.028:0.919:0.048:0.005	0.114:0.816:0.06:0.01
Saurischia	0.005:0.863:0.097:0.035	0.009:0.799:0.123:0.069	0.008:0.92:0.067:0.005	0.027:0.891:0.071:0.011	0.006:0.941:0.049:0.004	0.026:0.909:0.057:0.008
Theropoda	0.001:0.002:0.003:0.994	0.001:0.001:0.003:0.995	0.001:0.001:0.001:0.997	0.003:0.003:0.003:0.991	0.001:0.003:0.002:0.994	0.004:0.004:0.004:0.988

Table S29c. Continuation of Table S29b.

Character	Reproduction mode + egg shell mineralisation			
Model	mbl-ER (ind.hom)	mbl-ER (ind.het)	mbl-ER (sw.hom)	mbl-ER (sw.het)
Mean log marginal likelihood	-71.538	-70.537	-72.378	-71.163
Mean log Bayes Factor	1.680	3.681	0.000	2.430
Amniota	0.907:0.086:0.004:0.004	0.898:0.089:0.007:0.007	0.9:0.092:0.003:0.004	0.884:0.1:0.007:0.008
Mammalia	0.983:0.008:0.005:0.005	0.974:0.011:0.008:0.008	0.982:0.008:0.005:0.005	0.971:0.012:0.008:0.008
Reptilia	0.904:0.094:0.001:0.001	0.898:0.097:0.003:0.003	0.906:0.091:0.001:0.002	0.894:0.1:0.003:0.003
Diapsida s.l.	0.998:0.001:0:0	0.996:0.002:0.001:0.001	0.998:0.001:0.001:0.001	0.995:0.002:0.001:0.002
Diapsida s.s.	0.874:0.076:0.024:0.027	0.872:0.066:0.03:0.033	0.827:0.09:0.045:0.038	0.846:0.072:0.043:0.04
Lepidosauria	0.16:0.047:0.149:0.644	0.239:0.065:0.196:0.5	0.133:0.05:0.202:0.615	0.239:0.068:0.23:0.463
Archelosauria	0.92:0.052:0.014:0.014	0.913:0.049:0.017:0.02	0.893:0.062:0.025:0.019	0.895:0.055:0.026:0.024
Archosauromorpha	0.955:0.031:0.008:0.006	0.95:0.03:0.01:0.009	0.949:0.031:0.013:0.007	0.946:0.03:0.014:0.01
Archosauria	0.019:0.848:0.089:0.044	0.038:0.776:0.11:0.076	0.021:0.84:0.087:0.052	0.041:0.757:0.113:0.088
Dinosauria	0.012:0.88:0.073:0.035	0.021:0.827:0.091:0.062	0.014:0.877:0.07:0.039	0.022:0.816:0.092:0.07

Saurischia	0.007:0.881:0.079 :0.032	0.013:0.833:0.096 :0.058	0.006:0.875:0.081 :0.038	0.011:0.818:0.102 :0.069
Theropoda	0.001:0.001:0.001 :0.997	0.001:0.001:0.001 :0.997	0.001:0.001:0.001 :0.997	0.001:0.001:0.001 :0.997

Table S29d. Continuation of Table S29c.

Character	EER			
Model	mbl-EER ER (hom)	mbl-EER ER (het)	mbl-EER ARD (hom)	mbl-EER ARD (het)
Mean log marginal likelihood	-34.222	-33.274	-32.933	-32.155
Mean log Bayes Factor	0	1.895	2.577	4.134
Amniota	0.006:0.994	0.01:0.99	0.002:0.998	0.004:0.996
Mammalia	0.015:0.985	0.024:0.976	0.007:0.993	0.011:0.989
Reptilia	0.001:0.999	0.003:0.997	0:1	0.001:0.999
Diapsida s.l.	0.001:0.999	0.002:0.998	0:1	0.001:0.999
Diapsida s.s.	0.058:0.942	0.08:0.92	0.024:0.976	0.04:0.96
Lepidosauria	0.168:0.832	0.27:0.73	0.06:0.94	0.124:0.876
Archelosauria	0.107:0.893	0.109:0.891	0.05:0.95	0.058:0.942
Archosauromorpha	0.044:0.956	0.049:0.951	0.019:0.981	0.024:0.976
Archosauria	0.988:0.012	0.986:0.014	0.994:0.006	0.982:0.018
Dinosauria	0.993:0.007	0.992:0.008	0.996:0.004	0.988:0.012
Saurischia	0.996:0.004	0.995:0.005	0.998:0.002	0.992:0.008
Theropoda	0.999:0.001	0.999:0.001	1:0	0.999:0.001

Table S29e. Continuation of Table S29d. Nodes of Lepidosauria and Squamata fixed to a non-viviparous state.

Character	Reproduction mode + egg shell mineralisation					
Model	mbl-CER (ind.hom)	mbl-CER (ind.het)	mbl-CER (sw.hom)	mbl-CER (sw.het)	mbl-CSYM (ind.hom)	mbl-CSYM (ind.het)
Mean log marginal likelihood	-71.361	-70.458	-71.922	-70.818	-73.242	-72.201
Mean log Bayes Factor	2.301	4.107	1.178	3.387	-1.461	0.621
Amniota	0.918:0.076 :0.003:0.003	0.919:0.072 :0.004:0.005	0.91:0.084: 0.003:0.003	0.906:0.084 :0.005:0.006	0.923:0.071 :0.003:0.003	0.925:0.066 :0.005:0.004

Mammalia	0.984:0.007:0.004:0.004	0.978:0.01:0.006:0.006	0.983:0.008:0.005:0.005	0.977:0.01:0.006:0.006	0.984:0.007:0.005:0.004	0.978:0.01:0.006:0.006
Reptilia	0.913:0.086:0.001:0.001	0.915:0.082:0.002:0.002	0.912:0.086:0.001:0.001	0.909:0.086:0.002:0.002	0.918:0.08:0.001:0.001	0.921:0.075:0.002:0.002
Diapsida s.l.	0.998:0.001:0:0	0.997:0.001:0.001:0.001	0.998:0.001:0.001:0.001	0.996:0.002:0.001:0.001	0.998:0.001:0:0	0.997:0.001:0.001:0.001
Diapsida s.s.	0.841:0.094:0.031:0.034	0.837:0.082:0.038:0.043	0.791:0.109:0.054:0.046	0.81:0.088:0.053:0.049	0.824:0.098:0.041:0.038	0.823:0.082:0.047:0.048
Lepidosauria	0:0.054:0.191:0.755	0:0.081:0.278:0.64	0:0.058:0.236:0.706	0:0.09:0.314:0.596	0:0.055:0.188:0.757	0:0.093:0.252:0.655
Archelosauria	0.914:0.056:0.015:0.015	0.908:0.052:0.018:0.022	0.884:0.068:0.028:0.021	0.887:0.059:0.027:0.026	0.91:0.055:0.019:0.016	0.907:0.049:0.022:0.022
Archosauromorpha	0.955:0.031:0.008:0.006	0.952:0.03:0.01:0.008	0.949:0.032:0.013:0.007	0.946:0.031:0.014:0.009	0.955:0.03:0.009:0.006	0.955:0.027:0.011:0.008
Archosauria	0.015:0.856:0.086:0.042	0.031:0.785:0.111:0.073	0.017:0.846:0.086:0.051	0.033:0.768:0.114:0.085	0.015:0.833:0.108:0.043	0.03:0.753:0.139:0.078
Dinosauria	0.009:0.885:0.071:0.034	0.018:0.835:0.09:0.058	0.011:0.881:0.07:0.038	0.018:0.825:0.091:0.066	0.009:0.872:0.085:0.033	0.017:0.813:0.11:0.06
Saurischia	0.006:0.883:0.079:0.032	0.01:0.837:0.097:0.055	0.005:0.877:0.081:0.037	0.008:0.825:0.102:0.065	0.006:0.871:0.092:0.032	0.011:0.82:0.113:0.057
Theropoda	0.001:0.001:0.001:0.997	0.001:0.001:0.001:0.998	0.001:0.001:0.001:0.997	0.001:0.001:0.001:0.997	0.001:0.001:0.002:0.996	0.001:0.001:0.002:0.996

Table S29f. Continuation of Table S29e.

Character	Reproduction mode + egg shell mineralisation					
Model	mbl-CSYM (sw.hom)	mbl-CSYM (sw.het)	mbl-CARD (ind.hom)	mbl-CARD (ind.het)	mbl-CARD (sw.hom)	mbl-CARD (sw.het)
Mean log marginal likelihood	-73.467	-72.196	-72.526	-71.716	-71.304	-70.294

Mean log Bayes Factor	-1.913	0.631	-0.030	1.591	2.413	4.434
Amniota	0.92:0.074:0.003:0.003	0.92:0.07:0.005:0.005	0.893:0.103:0.003:0.001	0.913:0.082:0.003:0.002	0.926:0.072:0.001:0	0.941:0.057:0.001:0
Mammalia	0.983:0.007:0.005:0.005	0.978:0.01:0.007:0.006	0.984:0.01:0.004:0.002	0.981:0.012:0.004:0.003	0.99:0.008:0.001:0.001	0.988:0.009:0.001:0.001
Reptilia	0.921:0.077:0.001:0.001	0.922:0.074:0.002:0.002	0.884:0.115:0.001:0	0.899:0.099:0.001:0.001	0.866:0.133:0:0	0.89:0.11:0:0
Diapsida s.l.	0.998:0.001:0.001:0.001	0.996:0.001:0.001:0.001	0.998:0.001:0:0	0.998:0.002:0:0	0.998:0.002:0:0	0.997:0.002:0:0
Diapsida s.s.	0.765:0.117:0.067:0.051	0.788:0.092:0.064:0.056	0.865:0.108:0.018:0.009	0.867:0.102:0.018:0.013	0.8:0.181:0.013:0.006	0.839:0.142:0.011:0.007
Lepidosauria	0:0.064:0.221:0.715	0:0.111:0.284:0.605	0:0.061:0.188:0.751	0:0.092:0.24:0.668	0:0.063:0.245:0.692	0:0.108:0.287:0.604
Archelosauria	0.879:0.067:0.032:0.022	0.886:0.055:0.032:0.027	0.925:0.061:0.01:0.004	0.923:0.061:0.01:0.006	0.901:0.09:0.007:0.003	0.912:0.079:0.006:0.004
Archosauromorpha	0.95:0.03:0.013:0.007	0.95:0.026:0.014:0.01	0.959:0.034:0.006:0.002	0.958:0.034:0.006:0.002	0.959:0.037:0.003:0.001	0.961:0.035:0.003:0.001
Archosauria	0.015:0.819:0.114:0.051	0.027:0.729:0.149:0.095	0.02:0.904:0.069:0.008	0.038:0.873:0.071:0.018	0.041:0.929:0.025:0.004	0.079:0.88:0.031:0.01
Dinosauria	0.01:0.864:0.089:0.037	0.015:0.796:0.118:0.07	0.013:0.928:0.054:0.006	0.023:0.907:0.057:0.013	0.023:0.955:0.02:0.003	0.043:0.926:0.024:0.008
Saurischia	0.004:0.865:0.096:0.035	0.007:0.804:0.122:0.067	0.008:0.929:0.058:0.006	0.015:0.912:0.06:0.013	0.005:0.969:0.023:0.003	0.013:0.953:0.026:0.008
Theropoda	0.001:0.002:0.003:0.994	0.001:0.002:0.003:0.995	0.001:0.001:0.001:0.996	0.001:0.002:0.001:0.996	0.001:0.001:0.001:0.996	0.002:0.002:0.002:0.994

Table S29g. Continuation of Table S29f.

Character	Reproduction mode + egg shell mineralisation			
Model	mbl-ER (ind.hom)	mbl-ER (ind.het)	mbl-ER (sw.hom)	mbl-ER (sw.het)

Mean log marginal likelihood	-71.778	-70.882	-72.511	-71.448
Mean log Bayes Factor	1.467	3.258	0.000	2.127
Amniota	0.912:0.082:0.003:0.003	0.906:0.082:0.006:0.006	0.899:0.094:0.003:0.004	0.886:0.101:0.006:0.007
Mammalia	0.983:0.008:0.005:0.005	0.976:0.011:0.007:0.007	0.982:0.008:0.005:0.005	0.972:0.012:0.008:0.008
Reptilia	0.907:0.09:0.001:0.001	0.905:0.091:0.002:0.002	0.903:0.094:0.001:0.001	0.892:0.103:0.002:0.003
Diapsida s.l.	0.998:0.001:0:0	0.997:0.001:0.001:0.001	0.997:0.001:0.001:0.001	0.995:0.002:0.001:0.001
Diapsida s.s.	0.855:0.087:0.028:0.031	0.849:0.077:0.034:0.04	0.813:0.099:0.047:0.041	0.83:0.082:0.045:0.043
Lepidosauria	0:0.055:0.182:0.763	0:0.082:0.26:0.657	0:0.058:0.232:0.71	0:0.089:0.301:0.609
Archelosauria	0.918:0.054:0.014:0.014	0.91:0.051:0.018:0.021	0.891:0.064:0.025:0.019	0.894:0.057:0.025:0.024
Archosauromorpha	0.954:0.031:0.008:0.006	0.951:0.031:0.01:0.008	0.949:0.032:0.013:0.007	0.945:0.031:0.014:0.01
Archosauria	0.018:0.853:0.087:0.042	0.035:0.782:0.111:0.073	0.02:0.843:0.086:0.051	0.038:0.771:0.106:0.084
Dinosauria	0.011:0.884:0.071:0.034	0.019:0.831:0.09:0.059	0.013:0.879:0.069:0.038	0.022:0.824:0.087:0.067
Saurischia	0.007:0.883:0.079:0.032	0.012:0.834:0.098:0.057	0.006:0.876:0.081:0.037	0.01:0.825:0.098:0.067
Theropoda	0.001:0.001:0.001:0.998	0.001:0.001:0.001:0.998	0.001:0.001:0.001:0.997	0.001:0.001:0.001:0.997

Table S30a. Same as Table S28 but based on 100 trees time-scaled using the fossilised birth-death (FBD) tip-dating method with the root age constrained.

Character	Reproduction mode + egg shell mineralisation					
Model	FBD-CER (ind.hom)	FBD-CER (ind.het)	FBD-CER (sw.hom)	FBD-CER (sw.het)	FBD-CSYM (ind.hom)	FBD-CSYM (ind.het)
Mean log marginal likelihood	-70.913	-70.360	-71.733	-71.004	-72.408	-71.821
Mean log Bayes Factor	3.375	4.482	1.735	3.193	0.385	1.559
Amniota	0.951:0.031:0.009:0.01	0.945:0.031:0.011:0.013	0.905:0.058:0.025:0.011	0.907:0.05:0.026:0.017	0.956:0.023:0.011:0.011	0.95:0.025:0.011:0.015
Mammalia	0.817:0.103:0.04:0.04	0.801:0.112:0.044:0.044	0.816:0.101:0.042:0.042	0.796:0.109:0.048:0.048	0.812:0.117:0.035:0.036	0.801:0.124:0.035:0.04
Reptilia	0.949:0.037:0.007:0.007	0.95:0.034:0.008:0.009	0.946:0.035:0.012:0.007	0.943:0.034:0.013:0.01	0.962:0.024:0.007:0.007	0.961:0.023:0.007:0.009
Diapsida s.l.	0.847:0.027:0.06:0.066	0.871:0.021:0.048:0.06	0.821:0.026:0.091:0.061	0.849:0.022:0.069:0.061	0.855:0.017:0.063:0.066	0.874:0.014:0.049:0.063
Diapsida s.s.	0.15:0.139:0.334:0.376	0.229:0.112:0.291:0.368	0.133:0.114:0.455:0.298	0.223:0.096:0.374:0.307	0.154:0.082:0.369:0.394	0.222:0.069:0.305:0.405
Lepidosauria	0.108:0.064:0.712:0.116	0.136:0.071:0.684:0.109	0.191:0.062:0.638:0.109	0.222:0.064:0.618:0.097	0.117:0.052:0.698:0.133	0.138:0.055:0.698:0.109
Archelosauria	0.375:0.152:0.087:0.386	0.43:0.113:0.075:0.382	0.225:0.174:0.179:0.422	0.34:0.126:0.139:0.394	0.365:0.087:0.144:0.404	0.41:0.068:0.097:0.424
Archosauromorpha	0.752:0.116:0.06:0.072	0.749:0.099:0.055:0.097	0.631:0.147:0.13:0.093	0.656:0.122:0.106:0.116	0.742:0.069:0.094:0.096	0.737:0.062:0.07:0.131
Archosauria	0.01:0.581:0.077:0.331	0.02:0.461:0.092:0.427	0.01:0.58:0.087:0.323	0.023:0.468:0.102:0.407	0.011:0.325:0.238:0.427	0.019:0.271:0.17:0.539
Dinosauri	0.003:0.655	0.005:0.552	0.003:0.652	0.006:0.557	0.003:0.397	0.006:0.348

a	:0.089:0.253	:0.101:0.341	:0.098:0.247	:0.11:0.327	:0.244:0.356	:0.175:0.471
Saurischia	0.002:0.634:0.168:0.196	0.003:0.541:0.176:0.279	0.001:0.633:0.174:0.192	0.003:0.542:0.183:0.273	0.002:0.394:0.281:0.323	0.004:0.343:0.216:0.437
Theropoda	0.008:0.009:0.009:0.975	0.006:0.007:0.007:0.98	0.011:0.009:0.009:0.971	0.01:0.008:0.008:0.974	0.009:0.007:0.01:0.974	0.007:0.007:0.009:0.977

Table S30b. Continuation of Table S30a.

Character	Reproduction mode + egg shell mineralisation					
Model	FBD-CSYM (sw.hom)	FBD-CSYM (sw.het)	FBD-CARD (ind.hom)	FBD-CARD (ind.het)	FBD-CARD (sw.hom)	FBD-CARD (sw.het)
Mean log marginal likelihood	-72.789	-72.138	-65.994	-64.396	-66.876	-65.265
Mean log Bayes Factor	-0.377	0.925	13.213	16.409	11.449	14.672
Amniota	0.919:0.039:0.032:0.01	0.914:0.041:0.028:0.017	1:0:0:0	1:0:0:0	0.999:0.001:0:0	1:0:0:0
Mammalia	0.81:0.117:0.042:0.031	0.793:0.123:0.044:0.04	0.999:0.001:0:0	1:0:0:0	0.999:0.001:0:0	0.999:0:0:0
Reptilia	0.961:0.021:0.012:0.005	0.953:0.025:0.012:0.01	0.999:0.001:0:0	1:0:0:0	0.998:0.002:0:0	1:0:0:0
Diapsida s.l.	0.852:0.015:0.082:0.051	0.863:0.016:0.065:0.055	0.999:0.001:0:0	1:0:0:0	0.998:0.002:0:0	0.999:0:0:0
Diapsida s.s.	0.187:0.061:0.485:0.267	0.242:0.067:0.396:0.296	0.997:0.003:0:0	1:0:0:0	0.994:0.006:0:0	0.998:0.001:0:0
Lepidosauria	0.206:0.057:0.584:0.153	0.217:0.06:0.609:0.114	0.996:0.001:0.002:0.001	0.999:0:0.001:0	0.995:0.001:0.003:0.001	0.997:0:0.002:0.001
Archelosauria	0.272:0.083:0.301:0.344	0.359:0.078:0.199:0.365	0.997:0.002:0:0	1:0:0:0	0.995:0.005:0:0	0.998:0.001:0:0
Archosau	0.687:0.071	0.681:0.073	0.999:0.001	1:0:0:0	0.997:0.003	0.999:0.001

romorpha	:0.16:0.082	:0.126:0.12	:0:0		:0:0	:0:0
Archosauria	0.011:0.277:0.423:0.288	0.021:0.274:0.29:0.415	0.035:0.839:0.099:0.026	0.052:0.85:0.054:0.044	0.036:0.721:0.216:0.027	0.135:0.612:0.219:0.035
Dinosauria	0.003:0.348:0.425:0.223	0.006:0.353:0.298:0.342	0.012:0.876:0.092:0.02	0.022:0.895:0.051:0.032	0.016:0.758:0.207:0.019	0.083:0.682:0.21:0.024
Saurischia	0.002:0.357:0.445:0.197	0.003:0.351:0.333:0.314	0.009:0.863:0.108:0.021	0.016:0.898:0.056:0.03	0.006:0.73:0.244:0.021	0.034:0.718:0.225:0.022
Theropoda	0.014:0.006:0.013:0.967	0.011:0.006:0.011:0.973	0.032:0.035:0.03:0.903	0.042:0.038:0.034:0.887	0.039:0.038:0.037:0.885	0.102:0.065:0.056:0.777

Table S30c. Continuation of Table S30b.

Character	Reproduction mode + egg shell mineralisation			
Model	FBD-ER (ind.hom)	FBD-ER (ind.het)	FBD-ER (sw.hom)	FBD-ER (sw.het)
Mean log marginal likelihood	-71.616	-71.074	-72.601	-71.876
Mean log Bayes Factor	1.970	3.052	0	1.449
Amniota	0.948:0.032:0.009:0.01	0.941:0.032:0.012:0.015	0.905:0.057:0.026:0.012	0.906:0.049:0.028:0.017
Mammalia	0.82:0.1:0.04:0.04	0.797:0.111:0.046:0.046	0.815:0.1:0.043:0.043	0.795:0.108:0.048:0.048
Reptilia	0.948:0.038:0.007:0.007	0.949:0.033:0.008:0.01	0.947:0.034:0.012:0.007	0.943:0.033:0.013:0.011
Diapsida s.l.	0.85:0.028:0.058:0.064	0.877:0.02:0.045:0.059	0.826:0.026:0.088:0.059	0.856:0.021:0.064:0.058
Diapsida s.s.	0.159:0.146:0.328:0.367	0.24:0.111:0.283:0.366	0.145:0.116:0.446:0.292	0.237:0.096:0.365:0.302
Lepidosauria	0.116:0.07:0.688:0.127	0.141:0.071:0.677:0.112	0.202:0.064:0.621:0.113	0.232:0.064:0.607:0.098
Archelosauria	0.381:0.159:0.091:0.37	0.437:0.113:0.074:0.376	0.235:0.177:0.182:0.406	0.348:0.127:0.14:0.384
Archosauromorpha	0.748:0.12:0.061:0.071	0.749:0.099:0.055:0.098	0.634:0.146:0.13:0.09	0.66:0.121:0.105:0.114
Archosauria	0.011:0.59:0.082:0.317	0.022:0.463:0.094:0.421	0.011:0.586:0.089:0.314	0.025:0.47:0.103:0.402
Dinosauria	0.003:0.659:0.093	0.006:0.556:0.102	0.004:0.657:0.1:0.	0.007:0.556:0.112

	:0.244	:0.336	24	:0.325
Saurischia	0.002:0.639:0.17:0.189	0.004:0.541:0.175:0.28	0.002:0.639:0.173:0.186	0.003:0.542:0.183:0.272
Theropoda	0.008:0.009:0.009:0.973	0.008:0.008:0.008:0.976	0.012:0.01:0.01:0.969	0.01:0.008:0.008:0.973

Table S30d. Continuation of Table S30c.

Character	EER			
Model	FBD-EER ER (hom)	FBD-EER ER (het)	FBD-EER ARD (hom)	FBD-EER ARD (het)
Mean log marginal likelihood	-31.92	-31.832	-28.569	-28.469
Mean log Bayes Factor	0	0.177	6.703	6.902
Amniota	0.015:0.985	0.02:0.98	0.001:0.999	0.002:0.998
Mammalia	0.216:0.784	0.218:0.782	0.016:0.984	0.017:0.983
Reptilia	0.002:0.998	0.003:0.997	0:1	0.001:0.999
Diapsida s.l.	0.016:0.984	0.025:0.975	0.001:0.999	0.002:0.998
Diapsida s.s.	0.347:0.653	0.341:0.659	0.026:0.974	0.026:0.974
Lepidosauria	0.152:0.848	0.18:0.82	0.011:0.989	0.014:0.986
Archelosauria	0.63:0.37	0.563:0.437	0.047:0.953	0.044:0.956
Archosauromorpha	0.231:0.769	0.233:0.767	0.017:0.983	0.017:0.983
Archosauria	0.992:0.008	0.988:0.012	0.986:0.014	0.954:0.046
Dinosauria	0.997:0.003	0.997:0.003	0.995:0.005	0.979:0.021
Saurischia	0.999:0.001	0.999:0.001	0.998:0.002	0.989:0.011
Theropoda	0.992:0.008	0.994:0.006	0.987:0.013	0.958:0.042

Table S30e. Continuation of Table S30d. Nodes of Lepidosauria and Squamata fixed to a non-viviparous state.

Character	Reproduction mode + egg shell mineralisation					
Model	FBD-CER (ind.hom)	FBD-CER (ind.het)	FBD-CER (sw.hom)	FBD-CER (sw.het)	FBD-CSYM (ind.hom)	FBD-CSYM (ind.het)
Mean log marginal likelihood	-71.387	-70.817	-72.246	-71.531	-72.887	-72.289
Mean log Bayes Factor	3.484	4.624	1.766	3.196	0.484	1.680
Amniota	0.935:0.043	0.935:0.039	0.872:0.089	0.882:0.073	0.946:0.03:	0.941:0.031

	:0.01:0.012	:0.012:0.014	:0.023:0.016	:0.023:0.021	0.012:0.012	:0.012:0.016
Mammalia	0.8:0.112:0.044:0.044	0.79:0.119:0.046:0.046	0.794:0.113:0.047:0.047	0.788:0.117:0.048:0.048	0.795:0.128:0.038:0.039	0.785:0.133:0.038:0.044
Reptilia	0.926:0.053:0.01:0.011	0.935:0.043:0.01:0.012	0.909:0.06:0.019:0.013	0.913:0.053:0.018:0.016	0.946:0.034:0.01:0.01	0.947:0.031:0.01:0.012
Diapsida s.l.	0.785:0.038:0.084:0.093	0.826:0.027:0.064:0.083	0.727:0.041:0.137:0.095	0.777:0.033:0.099:0.091	0.804:0.023:0.083:0.089	0.833:0.018:0.067:0.082
Diapsida s.s.	0.086:0.145:0.36:0.41	0.148:0.111:0.323:0.418	0.051:0.121:0.486:0.343	0.108:0.108:0.41:0.374	0.087:0.083:0.391:0.438	0.144:0.076:0.344:0.436
Lepidosauria	0:0.063:0.816:0.121	0:0.065:0.829:0.107	0:0.066:0.811:0.123	0:0.073:0.811:0.116	0:0.048:0.814:0.138	0:0.057:0.825:0.118
Archelosauria	0.36:0.148:0.086:0.407	0.407:0.106:0.07:0.417	0.207:0.165:0.171:0.456	0.303:0.125:0.129:0.444	0.349:0.083:0.14:0.428	0.389:0.069:0.099:0.443
Archosauromorpha	0.745:0.117:0.061:0.077	0.739:0.099:0.054:0.107	0.622:0.146:0.132:0.1	0.63:0.129:0.106:0.135	0.737:0.067:0.091:0.105	0.726:0.062:0.072:0.14
Archosauria	0.01:0.568:0.077:0.345	0.018:0.446:0.086:0.45	0.009:0.561:0.083:0.347	0.019:0.45:0.095:0.435	0.01:0.311:0.226:0.453	0.018:0.27:0.168:0.544
Dinosauria	0.003:0.645:0.089:0.263	0.005:0.541:0.097:0.357	0.003:0.642:0.095:0.261	0.005:0.544:0.106:0.344	0.003:0.385:0.233:0.38	0.005:0.351:0.174:0.469
Saurischia	0.002:0.623:0.17:0.206	0.003:0.525:0.174:0.297	0.001:0.619:0.175:0.205	0.002:0.53:0.183:0.284	0.002:0.378:0.272:0.348	0.003:0.348:0.216:0.433
Theropoda	0.008:0.008:0.008:0.975	0.007:0.007:0.007:0.979	0.01:0.009:0.009:0.972	0.01:0.008:0.008:0.974	0.008:0.007:0.01:0.975	0.007:0.007:0.008:0.978

Table S30f. Continuation of Table S30e.

Character	Reproduction mode + egg shell mineralisation					
Model	FBD-CSYM (sw.hom)	FBD-CSYM (sw.het)	FBD-CARD (ind.hom)	FBD-CARD (ind.het)	FBD-CARD (sw.hom)	FBD-CARD (sw.het)
Mean log	-73.422	-72.695	-72.283	-71.858	-71.834	-71.316

marginal likelihood						
Mean log Bayes Factor	-0.587	0.868	1.693	2.542	2.590	3.626
Amniota	0.889:0.068:0.028:0.016	0.891:0.061:0.025:0.024	0.856:0.136:0.005:0.003	0.891:0.1:0.004:0.005	0.852:0.143:0.004:0.001	0.909:0.084:0.004:0.003
Mammalia	0.787:0.131:0.044:0.038	0.776:0.134:0.044:0.045	0.789:0.181:0.017:0.013	0.795:0.173:0.016:0.016	0.86:0.124:0.01:0.006	0.865:0.117:0.01:0.009
Reptilia	0.928:0.042:0.019:0.011	0.926:0.041:0.018:0.015	0.826:0.168:0.004:0.003	0.871:0.121:0.004:0.004	0.736:0.259:0.004:0.002	0.846:0.147:0.003:0.003
Diapsida s.l.	0.754:0.028:0.13:0.088	0.789:0.025:0.098:0.088	0.794:0.155:0.029:0.021	0.848:0.107:0.021:0.025	0.69:0.27:0.027:0.013	0.819:0.146:0.02:0.015
Diapsida s.s.	0.062:0.077:0.517:0.344	0.107:0.077:0.432:0.384	0.155:0.625:0.125:0.095	0.295:0.476:0.108:0.121	0.101:0.76:0.098:0.041	0.294:0.558:0.085:0.064
Lepidosauria	0:0.062:0.777:0.161	0:0.066:0.803:0.132	0:0.09:0.699:0.211	0:0.131:0.647:0.222	0:0.121:0.661:0.218	0:0.162:0.626:0.212
Archelosauria	0.239:0.094:0.257:0.41	0.307:0.082:0.175:0.436	0.422:0.458:0.043:0.076	0.514:0.35:0.032:0.105	0.243:0.67:0.048:0.038	0.445:0.46:0.034:0.061
Archosauromorpha	0.656:0.082:0.148:0.114	0.641:0.08:0.121:0.158	0.733:0.228:0.022:0.017	0.748:0.201:0.019:0.032	0.572:0.394:0.024:0.009	0.679:0.281:0.019:0.021
Archosauria	0.01:0.305:0.319:0.366	0.017:0.27:0.228:0.484	0.017:0.878:0.04:0.065	0.032:0.815:0.039:0.114	0.021:0.93:0.026:0.023	0.056:0.859:0.029:0.056
Dinosauria	0.003:0.376:0.326:0.295	0.005:0.346:0.234:0.415	0.005:0.9:0.039:0.056	0.01:0.855:0.038:0.098	0.009:0.947:0.025:0.019	0.026:0.9:0.028:0.047
Saurischia	0.001:0.374:0.359:0.266	0.002:0.339:0.274:0.385	0.003:0.881:0.063:0.053	0.007:0.844:0.057:0.093	0.003:0.94:0.039:0.018	0.007:0.908:0.04:0.045
Theropoda	0.012:0.007:0.013:0.967	0.01:0.007:0.011:0.972	0.013:0.012:0.01:0.965	0.019:0.016:0.011:0.955	0.019:0.018:0.013:0.95	0.032:0.023:0.012:0.932

Table S30g. Continuation of Table S30f.

Character	Reproduction mode + egg shell mineralisation			
Model	FBD-ER (ind.hom)	FBD-ER (ind.het)	FBD-ER (sw.hom)	FBD-ER (sw.het)
Mean log marginal likelihood	-72.102	-71.561	-73.129	-72.411
Mean log Bayes Factor	2.053	3.135	0	1.435
Amniota	0.935:0.043:0.011:0.012	0.932:0.04:0.013:0.015	0.871:0.089:0.023:0.016	0.88:0.073:0.024:0.023
Mammalia	0.797:0.112:0.045:0.045	0.788:0.118:0.047:0.047	0.795:0.112:0.046:0.046	0.782:0.117:0.051:0.051
Reptilia	0.926:0.053:0.01:0.011	0.932:0.045:0.01:0.013	0.909:0.06:0.019:0.013	0.911:0.054:0.018:0.017
Diapsida s.l.	0.792:0.038:0.08:0.09	0.824:0.027:0.065:0.083	0.729:0.041:0.135:0.094	0.777:0.033:0.098:0.092
Diapsida s.s.	0.091:0.149:0.354:0.407	0.152:0.116:0.319:0.413	0.054:0.123:0.483:0.34	0.109:0.11:0.403:0.378
Lepidosauria	0:0.064:0.811:0.125	0:0.067:0.822:0.111	0:0.067:0.807:0.125	0:0.076:0.8:0.124
Archelosauria	0.364:0.15:0.086:0.4	0.401:0.11:0.071:0.418	0.213:0.168:0.172:0.448	0.305:0.126:0.128:0.44
Archosauromorpha	0.743:0.118:0.061:0.077	0.732:0.103:0.056:0.109	0.62:0.148:0.133:0.099	0.632:0.128:0.106:0.133
Archosauria	0.01:0.57:0.078:0.342	0.019:0.45:0.088:0.443	0.01:0.565:0.083:0.341	0.021:0.451:0.093:0.435
Dinosauria	0.003:0.647:0.09:0.26	0.005:0.546:0.097:0.352	0.003:0.643:0.096:0.258	0.006:0.543:0.102:0.349
Saurischia	0.002:0.623:0.17:0.205	0.003:0.532:0.174:0.291	0.001:0.622:0.175:0.202	0.003:0.527:0.178:0.293
Theropoda	0.008:0.009:0.009:0.975	0.007:0.007:0.007:0.979	0.011:0.009:0.009:0.97	0.009:0.007:0.007:0.976

Table S31a. Same as Table S28 but based on 100 trees time-scaled using the fossilised birth-death (FBD) tip-dating method with the root age and the node age of major extant clades constrained.

Character	Reproduction mode + egg shell mineralisation					
Model	FBD_c-CER (ind.hom)	FBD_c-CER (ind.het)	FBD_c-CER (sw.hom)	FBD_c-CER (sw.het)	FBD_c-CSYM (ind.hom)	FBD_c-CSYM (ind.het)
Mean log marginal likelihood	-69.827	-69.827	-70.344	-69.817	-71.029	-70.585
Mean log Bayes Factor	2.828	3.79	1.795	2.847	0.425	1.313
Amniota	0.948:0.032:0.009:0.011	0.938:0.035:0.011:0.015	0.91:0.057:0.021:0.012	0.907:0.053:0.022:0.018	0.954:0.026:0.009:0.011	0.947:0.028:0.01:0.015
Mammalia	0.747:0.143:0.055:0.055	0.739:0.147:0.057:0.057	0.749:0.139:0.056:0.056	0.738:0.144:0.059:0.059	0.756:0.161:0.039:0.043	0.745:0.164:0.042:0.05
Reptilia	0.941:0.039:0.009:0.011	0.94:0.038:0.009:0.013	0.939:0.039:0.012:0.01	0.938:0.036:0.012:0.013	0.955:0.025:0.009:0.011	0.955:0.024:0.008:0.012
Diapsida s.l.	0.777:0.028:0.089:0.107	0.812:0.022:0.067:0.098	0.767:0.028:0.114:0.091	0.803:0.022:0.083:0.091	0.773:0.016:0.098:0.112	0.82:0.014:0.069:0.097
Diapsida s.s.	0.051:0.093:0.387:0.47	0.108:0.082:0.328:0.482	0.051:0.084:0.495:0.37	0.108:0.07:0.408:0.414	0.051:0.04:0.426:0.482	0.109:0.042:0.349:0.499
Lepidosauria	0.092:0.067:0.746:0.094	0.12:0.074:0.708:0.097	0.167:0.063:0.683:0.087	0.194:0.064:0.659:0.082	0.094:0.043:0.768:0.094	0.12:0.048:0.744:0.089
Archelosauria	0.204:0.123:0.089:0.584	0.268:0.098:0.076:0.558	0.129:0.139:0.154:0.578	0.21:0.102:0.124:0.564	0.203:0.052:0.169:0.575	0.262:0.048:0.113:0.577
Archosauromorpha	0.631:0.138:0.088:0.144	0.616:0.119:0.078:0.186	0.514:0.164:0.161:0.161	0.525:0.133:0.133:0.21	0.63:0.06:0.13:0.18	0.612:0.059:0.102:0.227
Archosauria	0.007:0.436:0.082:0.474	0.016:0.349:0.089:0.545	0.008:0.458:0.09:0.445	0.018:0.355:0.097:0.529	0.008:0.183:0.243:0.566	0.018:0.17:0.177:0.634

Dinosauria	0.005:0.502:0.108:0.385	0.007:0.427:0.108:0.458	0.005:0.518:0.117:0.359	0.008:0.439:0.117:0.437	0.005:0.241:0.249:0.505	0.008:0.234:0.183:0.575
Saurischia	0.002:0.499:0.204:0.295	0.004:0.43:0.193:0.374	0.002:0.51:0.21:0.279	0.003:0.436:0.204:0.357	0.003:0.235:0.29:0.472	0.005:0.231:0.228:0.536
Theropoda	0.006:0.006:0.006:0.982	0.005:0.006:0.006:0.984	0.007:0.006:0.006:0.982	0.007:0.005:0.005:0.983	0.005:0.004:0.006:0.984	0.005:0.004:0.006:0.985

Table S31b. Continuation of Table S31a.

Character	Reproduction mode + egg shell mineralisation					
Model	FBD_c-CSYM (sw.hom)	FBD_c-CSYM (sw.het)	FBD_c-CARD (ind.hom)	FBD_c-CARD (ind.het)	FBD_c-CARD (sw.hom)	FBD_c-CARD (sw.het)
Mean log marginal likelihood	-71.281	-70.781	-65.073	-63.602	-65.263	-63.973
Mean log Bayes Factor	-0.079	0.92	12.336	15.278	11.956	14.537
Amniota	0.916:0.048:0.024:0.012	0.912:0.046:0.023:0.02	1:0:0:0	1:0:0:0	0.999:0.001:0:0	1:0:0:0
Mammalia	0.754:0.16:0.049:0.037	0.742:0.16:0.049:0.048	1:0:0:0	0.999:0:0:0	0.998:0.001:0:0	0.999:0.001:0:0
Reptilia	0.953:0.026:0.013:0.008	0.949:0.026:0.012:0.013	1:0:0:0	1:0:0:0	0.998:0.002:0:0	1:0:0:0
Diapsida s.l.	0.776:0.017:0.123:0.084	0.809:0.016:0.084:0.091	1:0:0:0	1:0:0:0	0.998:0.002:0:0	1:0:0:0
Diapsida s.s.	0.07:0.036:0.573:0.321	0.12:0.041:0.432:0.407	0.998:0.001:0:0	0.999:0.001:0.001:0	0.993:0.006:0.001:0.001	0.999:0.001:0:0
Lepidosauria	0.173:0.051:0.674:0.102	0.194:0.051:0.666:0.088	0.998:0:0.001:0	0.998:0:0.001:0	0.994:0.001:0.005:0.001	0.998:0:0.001:0
Archelosauria	0.145:0.056:0.317:0.48	0.219:0.054:0.194:0.53	0.999:0.001:0:0	0.999:0.001:0:0	0.993:0.006:0:0.001	0.999:0.001:0:0

	2	3				
Archosauromorpha	0.556:0.07:0.214:0.159	0.537:0.069:0.163:0.231	0.999:0.001:0:0	0.999:0:0:0	0.996:0.004:0:0	0.999:0.001:0:0
Archosauria	0.008:0.186:0.392:0.414	0.018:0.178:0.254:0.551	0.034:0.816:0.11:0.039	0.059:0.82:0.061:0.06	0.031:0.637:0.305:0.027	0.157:0.533:0.272:0.038
Dinosauria	0.006:0.242:0.397:0.355	0.008:0.245:0.263:0.483	0.018:0.841:0.107:0.033	0.029:0.864:0.059:0.048	0.022:0.651:0.304:0.023	0.114:0.595:0.263:0.028
Saurischia	0.002:0.24:0.434:0.324	0.003:0.242:0.308:0.446	0.01:0.834:0.124:0.033	0.018:0.873:0.065:0.044	0.007:0.614:0.355:0.024	0.053:0.637:0.284:0.026
Theropoda	0.007:0.003:0.006:0.983	0.007:0.004:0.007:0.982	0.028:0.029:0.024:0.919	0.037:0.033:0.029:0.901	0.028:0.027:0.027:0.918	0.105:0.062:0.051:0.782

Table S31c. Continuation of Table S31b.

Character	Reproduction mode + egg shell mineralisation			
Model	FBD_c-ER (ind.hom)	FBD_c-ER (ind.het)	FBD_c-ER (sw.hom)	FBD_c-ER (sw.het)
Mean log marginal likelihood	-70.552	-70.083	-71.241	-70.72
Mean log Bayes Factor	1.379	2.316	0	1.043
Amniota	0.948:0.032:0.009:0.011	0.939:0.033:0.012:0.016	0.909:0.058:0.021:0.012	0.903:0.053:0.024:0.019
Mammalia	0.748:0.141:0.055:0.055	0.735:0.146:0.06:0.06	0.749:0.139:0.056:0.056	0.733:0.142:0.062:0.062
Reptilia	0.942:0.039:0.009:0.01	0.944:0.035:0.009:0.012	0.938:0.039:0.013:0.01	0.935:0.038:0.013:0.014
Diapsida s.l.	0.783:0.028:0.087:0.102	0.825:0.022:0.063:0.09	0.764:0.028:0.115:0.093	0.803:0.023:0.083:0.091
Diapsida s.s.	0.052:0.096:0.388:0.464	0.116:0.085:0.325:0.473	0.051:0.083:0.493:0.373	0.115:0.075:0.406:0.404
Lepidosauria	0.095:0.068:0.739:0.098	0.128:0.077:0.695:0.1	0.167:0.063:0.683:0.087	0.207:0.067:0.64:0.086
Archelosauria	0.208:0.129:0.091:0.573	0.273:0.101:0.079:0.547	0.128:0.138:0.152:0.581	0.209:0.11:0.13:0.551
Archosauromo	0.63:0.142:0.088:	0.615:0.121:0.08:	0.511:0.165:0.16:	0.515:0.141:0.138

rpha	0.14	0.184	0.164	:0.207
Archosauria	0.008:0.449:0.084:0.459	0.017:0.354:0.094:0.535	0.008:0.457:0.088:0.447	0.019:0.364:0.1:0.517
Dinosauria	0.005:0.511:0.11:0.374	0.008:0.432:0.111:0.449	0.005:0.52:0.115:0.359	0.008:0.44:0.119:0.433
Saurischia	0.003:0.506:0.205:0.286	0.004:0.429:0.197:0.369	0.002:0.512:0.207:0.279	0.003:0.437:0.204:0.356
Theropoda	0.005:0.006:0.006:0.984	0.005:0.005:0.005:0.984	0.007:0.006:0.006:0.98	0.007:0.006:0.006:0.981

Table S31d. Continuation of Table S31c.

Character	EER			
Model	FBD_c-EER ER (hom)	FBD_c-EER ER (het)	FBD_c-EER ARD (hom)	FBD_c-EER ARD (het)
Mean log marginal likelihood	-31.700	-31.662	-28.227	-28.107
Mean log Bayes Factor	0	0.076	6.945	7.186
Amniota	0.014:0.986	0.02:0.98	0.001:0.999	0.001:0.999
Mammalia	0.268:0.732	0.273:0.727	0.017:0.983	0.02:0.98
Reptilia	0.002:0.998	0.004:0.996	0:1	0:1
Diapsida s.l.	0.022:0.978	0.032:0.968	0.002:0.998	0.002:0.998
Diapsida s.s.	0.503:0.497	0.467:0.533	0.033:0.967	0.033:0.967
Lepidosauria	0.21:0.79	0.236:0.764	0.013:0.987	0.017:0.983
Archelosauria	0.756:0.244	0.673:0.327	0.049:0.951	0.048:0.952
Archosauromorpha	0.336:0.664	0.329:0.671	0.022:0.978	0.021:0.979
Archosauria	0.991:0.009	0.985:0.015	0.986:0.014	0.946:0.054
Dinosauria	0.997:0.003	0.997:0.003	0.995:0.005	0.975:0.025
Saurischia	0.999:0.001	0.999:0.001	0.998:0.002	0.986:0.014
Theropoda	0.992:0.008	0.993:0.007	0.986:0.014	0.95:0.05

Table S31e. Continuation of Table S31d. Nodes of Lepidosauria and Squamata fixed to a non-viviparous state.

Character	Reproduction mode + egg shell mineralisation					
Model	FBD_c-CER (ind.hom)	FBD_c-CER (ind.het)	FBD_c-CER (sw.hom)	FBD_c-CER (sw.het)	FBD_c-CSYM (ind.hom)	FBD_c-CSYM (ind.het)
Mean log marginal likelihood	-70.146	-69.719	-70.637	-70.162	-71.358	-70.935

Mean log Bayes Factor	2.812	3.665	1.829	2.779	0.387	1.233
Amniota	0.938:0.040:0.010:0.012	0.934:0.039:0.011:0.016	0.891:0.076:0.019:0.015	0.887:0.072:0.02:0.021	0.945:0.033:0.01:0.012	0.937:0.035:0.011:0.017
Mammalia	0.749:0.145:0.053:0.053	0.738:0.151:0.056:0.056	0.752:0.141:0.053:0.053	0.74:0.147:0.057:0.057	0.758:0.163:0.037:0.042	0.747:0.167:0.038:0.048
Reptilia	0.926:0.05:0.011:0.013	0.93:0.043:0.011:0.016	0.914:0.056:0.016:0.014	0.912:0.053:0.016:0.018	0.941:0.033:0.012:0.014	0.94:0.031:0.011:0.017
Diapsida s.l.	0.723:0.034:0.111:0.132	0.768:0.026:0.083:0.123	0.69:0.037:0.15:0.123	0.735:0.03:0.112:0.123	0.715:0.021:0.123:0.141	0.76:0.017:0.093:0.13
Diapsida s.s.	0.018:0.087:0.401:0.494	0.046:0.074:0.351:0.528	0.011:0.079:0.506:0.403	0.029:0.072:0.429:0.47	0.018:0.038:0.432:0.512	0.045:0.038:0.367:0.55
Lepidosauria	0:0.069:0.835:0.096	0:0.074:0.831:0.095	0:0.071:0.831:0.098	0:0.077:0.826:0.097	0:0.045:0.859:0.096	0:0.045:0.868:0.087
Archelosauria	0.194:0.112:0.082:0.612	0.23:0.086:0.069:0.614	0.118:0.125:0.138:0.619	0.166:0.097:0.11:0.628	0.192:0.048:0.157:0.602	0.224:0.043:0.105:0.629
Archosauromorpha	0.639:0.131:0.084:0.146	0.6:0.117:0.078:0.205	0.513:0.159:0.155:0.173	0.489:0.14:0.133:0.238	0.636:0.058:0.121:0.185	0.591:0.056:0.1:0.252
Archosauria	0.007:0.423:0.077:0.494	0.014:0.331:0.083:0.572	0.007:0.44:0.081:0.473	0.013:0.339:0.086:0.561	0.007:0.181:0.223:0.589	0.013:0.152:0.162:0.673
Dinosauria	0.004:0.491:0.105:0.4	0.006:0.415:0.105:0.474	0.005:0.504:0.111:0.381	0.007:0.419:0.111:0.463	0.004:0.236:0.231:0.529	0.006:0.213:0.17:0.611
Saurischia	0.002:0.486:0.205:0.306	0.004:0.413:0.197:0.387	0.002:0.494:0.208:0.296	0.003:0.414:0.202:0.381	0.002:0.228:0.273:0.496	0.004:0.208:0.214:0.574
Theropoda	0.005:0.005:0.005:0.985	0.005:0.005:0.005:0.985	0.006:0.005:0.005:0.983	0.006:0.005:0.005:0.984	0.005:0.004:0.006:0.985	0.005:0.004:0.005:0.986

Table S31f. Continuation of Table S31e.

Character	Reproduction mode + egg shell mineralisation

Model	FBD_c-CSYM (sw.hom)	FBD_c-CSYM (sw.het)	FBD_c-CARD (ind.hom)	FBD_c-CARD (ind.het)	FBD_c-CARD (sw.hom)	FBD_c-CARD (sw.het)
Mean log marginal likelihood	-71.653	-71.144	-71.433	-71.159	-70.713	-70.548
Mean log Bayes Factor	-0.203	0.816	0.236	0.785	1.678	2.008
Amniota	0.892:0.069:0.021:0.017	0.894:0.063:0.019:0.024	0.787:0.201:0.007:0.006	0.838:0.143:0.007:0.012	0.835:0.158:0.004:0.003	0.874:0.114:0.005:0.006
Mammalia	0.759:0.159:0.043:0.04	0.745:0.163:0.043:0.05	0.694:0.261:0.024:0.021	0.719:0.233:0.022:0.026	0.787:0.19:0.013:0.01	0.8:0.172:0.014:0.014
Reptilia	0.93:0.039:0.018:0.014	0.93:0.037:0.015:0.018	0.743:0.243:0.007:0.007	0.812:0.169:0.007:0.012	0.714:0.279:0.005:0.003	0.798:0.191:0.005:0.006
Diapsida s.l.	0.685:0.024:0.163:0.128	0.744:0.022:0.11:0.125	0.664:0.229:0.058:0.05	0.742:0.153:0.043:0.062	0.648:0.288:0.04:0.024	0.746:0.192:0.031:0.03
Diapsida s.s.	0.013:0.04:0.547:0.4	0.033:0.044:0.438:0.486	0.035:0.613:0.185:0.166	0.12:0.489:0.155:0.237	0.024:0.779:0.129:0.069	0.136:0.637:0.115:0.111
Lepidosauria	0:0.056:0.834:0.11	0:0.055:0.846:0.099	0:0.093:0.737:0.17	0:0.129:0.664:0.207	0.024:0.779:0.129:0.069	0:0.148:0.668:0.184
Archelosauria	0.131:0.058:0.249:0.562	0.173:0.055:0.16:0.612	0.222:0.54:0.067:0.171	0.309:0.407:0.046:0.238	0.116:0.746:0.059:0.079	0.265:0.571:0.045:0.119
Archosauromorpha	0.539:0.074:0.182:0.206	0.504:0.074:0.145:0.278	0.57:0.336:0.042:0.052	0.59:0.281:0.035:0.095	0.435:0.502:0.035:0.028	0.51:0.402:0.033:0.054
Archosauria	0.007:0.196:0.291:0.505	0.015:0.177:0.191:0.616	0.01:0.785:0.057:0.148	0.02:0.694:0.05:0.236	0.011:0.904:0.031:0.054	0.041:0.817:0.034:0.108
Dinosauria	0.005:0.253:0.301:0.441	0.007:0.242:0.201:0.55	0.006:0.797:0.06:0.137	0.011:0.722:0.052:0.215	0.011:0.906:0.034:0.049	0.027:0.84:0.035:0.098
Saurischia	0.002:0.246:0.343:0.40	0.003:0.234:0.248:0.51	0.003:0.778:0.092:0.12	0.006:0.715:0.078:0.20	0.002:0.896:0.054:0.04	0.007:0.847:0.052:0.09

	9	5	7	1	7	3
Theropoda	0.006:0.004:0.006:0.984	0.006:0.004:0.006:0.984	0.007:0.007:0.006:0.98	0.011:0.009:0.007:0.973	0.009:0.01:0.007:0.973	0.021:0.015:0.008:0.956

Table S31g. Continuation of Table S31f.

Character	Reproduction mode + egg shell mineralisation			
Model	FBD_c-ER (ind.hom)	FBD_c-ER (ind.het)	FBD_c-ER (sw.hom)	FBD_c-ER (sw.het)
Mean log marginal likelihood	-70.889	-70.465	-71.552	-71.084
Mean log Bayes Factor	1.324	2.173	0	0.934
Amniota	0.937:0.041:0.01:0.012	0.926:0.043:0.013:0.018	0.889:0.077:0.019:0.015	0.891:0.067:0.02:0.022
Mammalia	0.749:0.144:0.053:0.053	0.736:0.149:0.058:0.058	0.751:0.141:0.054:0.054	0.739:0.144:0.059:0.059
Reptilia	0.924:0.051:0.012:0.014	0.924:0.047:0.012:0.017	0.913:0.057:0.016:0.014	0.917:0.049:0.016:0.018
Diapsida s.l.	0.721:0.035:0.112:0.133	0.759:0.028:0.086:0.126	0.691:0.037:0.149:0.122	0.745:0.029:0.107:0.118
Diapsida s.s.	0.018:0.089:0.401:0.492	0.045:0.078:0.349:0.528	0.012:0.083:0.504:0.401	0.032:0.074:0.427:0.467
Lepidosauria	0:0.07:0.831:0.098	0:0.08:0.816:0.104	0:0.074:0.825:0.101	0:0.079:0.82:0.101
Archelosauria	0.195:0.115:0.083:0.607	0.228:0.091:0.071:0.61	0.121:0.129:0.14:0.61	0.171:0.099:0.113:0.617
Archosauromorpha	0.635:0.134:0.085:0.146	0.587:0.122:0.081:0.21	0.513:0.163:0.155:0.169	0.493:0.14:0.136:0.231
Archosauria	0.007:0.427:0.078:0.488	0.014:0.331:0.086:0.57	0.007:0.448:0.082:0.462	0.015:0.346:0.088:0.552
Dinosauria	0.004:0.494:0.106:0.396	0.006:0.412:0.108:0.474	0.005:0.511:0.112:0.373	0.007:0.425:0.112:0.456
Saurischia	0.002:0.489:0.206:0.303	0.004:0.414:0.199:0.384	0.002:0.5:0.208:0.29	0.003:0.422:0.201:0.373
Theropoda	0.005:0.005:0.005:0.985	0.005:0.005:0.005:0.986	0.006:0.005:0.005:0.983	0.006:0.005:0.005:0.985

Table S32a. Same as Table S28 but excluding *Mesosaurus tenuidens* from the analyses (equal dating method).

Character	Reproduction mode + egg shell mineralisation					
Model	equal-CER (ind.hom)	equal-CER (ind.het)	equal-CER (sw.hom)	equal-CER (sw.het)	equal-CSYM (ind.hom)	equal-CSYM (ind.het)
Mean log marginal likelihood	-70.404	-69.817	-71.697	-70.958	-71.712	-71.096
Mean log Bayes Factor	2.944	4.118	0.357	1.836	0.329	1.559
Amniota	0.914:0.015:0.026:0.046	0.911:0.016:0.025:0.048	0.853:0.026:0.053:0.068	0.854:0.026:0.048:0.072	0.914:0.015:0.028:0.043	0.907:0.017:0.027:0.049
Mammalia	0.726:0.144:0.065:0.065	0.718:0.15:0.066:0.066	0.724:0.146:0.065:0.065	0.716:0.149:0.067:0.067	0.723:0.166:0.055:0.056	0.719:0.168:0.053:0.06
Reptilia/ Diapsida s.l.	0.855:0.013:0.047:0.086	0.875:0.011:0.038:0.077	0.833:0.014:0.064:0.089	0.849:0.013:0.052:0.087	0.859:0.011:0.052:0.078	0.871:0.01:0.044:0.076
Diapsida s.s.	0.06:0.047:0.302:0.591	0.156:0.039:0.253:0.553	0.053:0.042:0.376:0.529	0.139:0.037:0.315:0.509	0.072:0.037:0.362:0.529	0.161:0.035:0.284:0.519
Lepidosauria	0.055:0.033:0.856:0.056	0.081:0.044:0.814:0.062	0.115:0.028:0.814:0.044	0.148:0.034:0.771:0.046	0.063:0.029:0.841:0.067	0.083:0.033:0.827:0.057
Archelosauria	0.368:0.051:0.026:0.555	0.413:0.039:0.023:0.525	0.254:0.059:0.041:0.646	0.347:0.046:0.038:0.569	0.378:0.034:0.091:0.497	0.419:0.031:0.055:0.495
Archosauromorpha	0.909:0.037:0.015:0.039	0.857:0.043:0.02:0.08	0.856:0.054:0.029:0.06	0.799:0.059:0.035:0.107	0.908:0.021:0.028:0.043	0.855:0.027:0.032:0.085
Archosauria	0.002:0.432:0.047:0.519	0.005:0.333:0.053:0.608	0.002:0.437:0.045:0.516	0.006:0.343:0.057:0.594	0.003:0.237:0.215:0.546	0.005:0.2:0.151:0.643
Dinosauria	0.001:0.65:0.064:0.284	0.003:0.54:0.074:0.383	0.001:0.654:0.063:0.281	0.003:0.538:0.078:0.381	0.001:0.38:0.226:0.393	0.002:0.34:0.165:0.493
Saurischia	0.001:0.606:0.147:0.24	0.002:0.509:0.153:0.33	0.001:0.612:0.142:0.24	0.001:0.513:0.154:0.33	0.001:0.356:0.264:0.37	0.002:0.321:0.207:0.47

	7	6	5	2	9	
Theropoda	0.012:0.013:0.013:0.962	0.011:0.012:0.012:0.965	0.015:0.014:0.014:0.957	0.015:0.012:0.012:0.961	0.014:0.011:0.014:0.961	0.012:0.011:0.013:0.964

Table S32b. Continuation of Table S32a.

Character	Reproduction mode + egg shell mineralisation					
Model	equal-CSYM (sw.hom)	equal-CSYM (sw.het)	equal-CARD (ind.hom)	equal-CARD (ind.het)	equal-CARD (sw.hom)	equal-CARD (sw.het)
Mean log marginal likelihood	-72.236	-71.653	-63.198	-61.501	-63.779	-61.973
Mean log Bayes Factor	-0.720	0.445	17.355	20.749	16.193	19.806
Amniota	0.866:0.023:0.058:0.052	0.857:0.025:0.051:0.066	1:0:0:0	1:0:0:0	1:0:0:0	1:0:0:0
Mammalia	0.717:0.176:0.061:0.045	0.717:0.172:0.057:0.054	1:0:0:0	1:0:0:0	1:0:0:0	1:0:0:0
Reptilia/ Diapsida s.l.	0.858:0.011:0.067:0.064	0.854:0.011:0.058:0.076	1:0:0:0	1:0:0:0	1:0:0:0	1:0:0:0
Diapsida s.s.	0.104:0.026:0.513:0.357	0.172:0.027:0.373:0.427	1:0:0:0	0.999:0:0:0	1:0:0:0	1:0:0:0
Lepidosauria	0.147:0.029:0.757:0.068	0.163:0.03:0.755:0.052	1:0:0:0	0.999:0:0.001:0	0.999:0:0:0	0.999:0:0:0
Archelosauria	0.287:0.028:0.228:0.457	0.362:0.028:0.12:0.491	1:0:0:0	1:0:0:0	1:0:0:0	1:0:0:0
Archosauromorpha	0.879:0.02:0.051:0.049	0.813:0.027:0.055:0.105	1:0:0:0	1:0:0:0	1:0:0:0	1:0:0:0
Archosauria	0.003:0.155:0.431:0.412	0.006:0.156:0.286:0.552	0.013:0.933:0.04:0.014	0.039:0.889:0.049:0.023	0.01:0.733:0.252:0.005	0.138:0.554:0.303:0.006
Dinosauri	0.002:0.265	0.003:0.273	0.009:0.944	0.027:0.912	0.011:0.743	0.122:0.597

a	:0.443:0.29	:0.301:0.423	:0.038:0.01	:0.045:0.016	:0.242:0.004	:0.276:0.004
Saurischia	0.001:0.255:0.46:0.284	0.002:0.26:0.33:0.408	0.006:0.941:0.045:0.008	0.021:0.926:0.04:0.014	0.003:0.712:0.282:0.003	0.057:0.667:0.272:0.004
Theropoda	0.019:0.008:0.016:0.957	0.018:0.009:0.015:0.959	0.059:0.061:0.051:0.828	0.094:0.07:0.058:0.778	0.06:0.057:0.055:0.828	0.195:0.111:0.08:0.615

Table S32c. Continuation of Table S32b.

Character	Reproduction mode + egg shell mineralisation			
Model	equal-ER (ind.hom)	equal-ER (ind.het)	equal-ER (sw.hom)	equal-ER (sw.het)
Mean log marginal likelihood	-71.220	-70.642	-71.876	-71.272
Mean log Bayes Factor	1.313	2.468	0	1.207
Amniota	0.914:0.015:0.026:0.045	0.904:0.017:0.027:0.052	0.86:0.025:0.053:0.062	0.859:0.025:0.048:0.068
Mammalia	0.724:0.143:0.066:0.066	0.717:0.147:0.068:0.068	0.72:0.144:0.068:0.068	0.712:0.146:0.071:0.071
Reptilia/Diapsida s.l.	0.857:0.013:0.046:0.084	0.867:0.011:0.039:0.083	0.845:0.014:0.061:0.081	0.855:0.013:0.051:0.081
Diapsida s.s.	0.066:0.051:0.299:0.585	0.151:0.039:0.253:0.556	0.069:0.047:0.382:0.501	0.156:0.038:0.314:0.492
Lepidosauria	0.059:0.036:0.844:0.061	0.085:0.044:0.808:0.063	0.137:0.032:0.778:0.053	0.17:0.036:0.743:0.051
Archelosauria	0.373:0.055:0.028:0.544	0.402:0.041:0.024:0.533	0.275:0.067:0.049:0.608	0.361:0.049:0.042:0.548
Archosauromorpha	0.909:0.038:0.015:0.038	0.846:0.047:0.021:0.086	0.857:0.056:0.032:0.055	0.797:0.062:0.038:0.103
Archosauria	0.002:0.442:0.049:0.507	0.005:0.333:0.054:0.609	0.003:0.459:0.055:0.483	0.006:0.357:0.065:0.572
Dinosauria	0.001:0.652:0.066:0.281	0.002:0.529:0.075:0.393	0.002:0.662:0.072:0.265	0.003:0.546:0.084:0.367
Saurischia	0.001:0.608:0.148:0.244	0.002:0.503:0.155:0.341	0.001:0.615:0.151:0.233	0.001:0.515:0.162:0.321
Theropoda	0.013:0.014:0.014:0.959	0.012:0.012:0.012:0.964	0.018:0.016:0.016:0.95	0.016:0.014:0.014:0.957

Table S32d. Continuation of Table S32c.

Character	EER			
Model	equal-EER ER (hom)	equal-EER ER (het)	equal-EER ARD (hom)	equal-EER ARD (het)
Mean log marginal likelihood	-31.59	-31.552	-27.672	-27.458
Mean log Bayes Factor	0	0.075	7.835	8.264
Amniota	0.014:0.986	0.022:0.978	0.001:0.999	0.001:0.999
Mammalia	0.27:0.73	0.279:0.721	0.011:0.989	0.01:0.99
Reptilia/Diapsida s.l.	0.005:0.995	0.01:0.99	0:1	0:1
Diapsida s.s.	0.173:0.827	0.188:0.812	0.007:0.993	0.006:0.994
Lepidosauria	0.079:0.921	0.112:0.888	0.003:0.997	0.004:0.996
Archelosauria	0.644:0.356	0.515:0.485	0.027:0.973	0.017:0.983
Archosauromorpha	0.083:0.917	0.094:0.906	0.003:0.997	0.003:0.997
Archosauria	0.998:0.002	0.996:0.004	0.997:0.003	0.972:0.028
Dinosauria	0.999:0.001	0.999:0.001	0.998:0.002	0.979:0.021
Saurischia	0.999:0.001	0.999:0.001	0.999:0.001	0.985:0.015
Theropoda	0.989:0.011	0.991:0.009	0.982:0.018	0.929:0.071

Table S32e. Continuation of Table S32d. Nodes of Lepidosauria and Squamata fixed to a non-viviparous state.

Character	Reproduction mode + egg shell mineralisation					
Model	equal-CER (ind.hom)	equal-CER (ind.het)	equal-CER (sw.hom)	equal-CER (sw.het)	equal-CSYM (ind.hom)	equal-CSYM (ind.het)
Mean log marginal likelihood	-70.704	-70.136	-71.924	-71.246	-72.025	-71.418
Mean log Bayes Factor	2.911	4.047	0.471	1.827	0.268	1.484
Amniota	0.891:0.018:0.033:0.058	0.889:0.019:0.032:0.061	0.823:0.033:0.06:0.084	0.825:0.032:0.055:0.088	0.889:0.019:0.035:0.057	0.887:0.02:0.032:0.061
Mammalia	0.728:0.146:0.063:0.063	0.724:0.15:0.063:0.063	0.727:0.148:0.063:0.063	0.722:0.15:0.064:0.064	0.726:0.167:0.051:0.056	0.724:0.169:0.049:0.058
Reptilia/Diapsida s.l.	0.811:0.016:0.061:0.112	0.832:0.014:0.051:0.102	0.789:0.018:0.08:0.113	0.805:0.016:0.067:0.112	0.811:0.014:0.069:0.106	0.83:0.013:0.056:0.1
Diapsida	0.021:0.045	0.075:0.038	0.012:0.041	0.045:0.037	0.023:0.036	0.07:0.032:

s.s.	:0.314:0.621	:0.282:0.605	:0.386:0.56	:0.337:0.581	:0.363:0.579	0.312:0.586
Lepidosauria	0:0.031:0.916:0.052	0:0.038:0.908:0.054	0:0.029:0.926:0.045	0:0.038:0.911:0.051	0:0.028:0.915:0.058	0:0.031:0.914:0.055
Archelosauria	0.357:0.046:0.024:0.573	0.367:0.037:0.022:0.575	0.238:0.054:0.036:0.672	0.281:0.043:0.033:0.643	0.363:0.031:0.079:0.528	0.364:0.028:0.056:0.552
Archosauromorpha	0.911:0.035:0.014:0.04	0.84:0.047:0.021:0.092	0.855:0.054:0.028:0.063	0.773:0.063:0.036:0.127	0.91:0.02:0.024:0.046	0.839:0.027:0.035:0.098
Archosauria	0.002:0.414:0.042:0.542	0.004:0.313:0.049:0.634	0.002:0.424:0.039:0.535	0.004:0.322:0.048:0.626	0.002:0.232:0.177:0.589	0.004:0.183:0.142:0.672
Dinosauria	0.001:0.645:0.061:0.293	0.002:0.523:0.072:0.403	0.001:0.653:0.058:0.288	0.002:0.532:0.069:0.397	0.001:0.383:0.19:0.426	0.002:0.328:0.157:0.513
Saurischia	0.001:0.6:0.144:0.255	0.002:0.494:0.153:0.351	0:0.611:0.137:0.251	0.001:0.506:0.147:0.346	0.001:0.357:0.231:0.41	0.002:0.31:0.2:0.489
Theropoda	0.011:0.012:0.012:0.966	0.01:0.011:0.011:0.968	0.014:0.012:0.012:0.962	0.013:0.011:0.011:0.965	0.012:0.011:0.013:0.965	0.011:0.01:0.012:0.967

Table S32f. Continuation of Table S32e.

Character	Reproduction mode + egg shell mineralisation					
Model	equal-CSYM (sw.hom)	equal-CSYM (sw.het)	equal-CARD (ind.hom)	equal-CARD (ind.het)	equal-CARD (sw.hom)	equal-CARD (sw.het)
Mean log marginal likelihood	-72.614	-71.996	-71.586	-70.699	-71.038	-70.129
Mean log Bayes Factor	-0.908	0.327	1.147	2.921	2.243	4.062
Amniota	0.818:0.034:0.069:0.079	0.823:0.034:0.056:0.087	0.862:0.102:0.016:0.021	0.903:0.055:0.015:0.027	0.932:0.049:0.01:0.008	0.961:0.025:0.006:0.008
Mammalia	0.724:0.175:0.051:0.05	0.719:0.174:0.051:0.057	0.718:0.233:0.025:0.024	0.786:0.171:0.019:0.023	0.845:0.123:0.017:0.015	0.89:0.086:0.012:0.012
Reptilia/	0.791:0.017	0.807:0.015	0.819:0.126	0.881:0.062	0.847:0.124	0.927:0.053

Diapsida s.l.	:0.095:0.097	:0.075:0.102	:0.024:0.03	:0.021:0.036	:0.017:0.012	:0.01:0.011
Diapsida s.s.	0.015:0.039:0.481:0.466	0.047:0.035:0.386:0.531	0.199:0.58:0.102:0.118	0.462:0.312:0.087:0.139	0.168:0.706:0.08:0.045	0.516:0.38:0.053:0.051
Lepidosauria	0:0.029:0.912:0.059	0:0.032:0.914:0.054	0:0.11:0.723:0.167	0:0.153:0.677:0.17	0:0.143:0.754:0.103	0:0.175:0.724:0.1
Archelosauria	0.258:0.035:0.166:0.541	0.294:0.031:0.101:0.574	0.529:0.353:0.023:0.095	0.679:0.184:0.019:0.118	0.41:0.518:0.024:0.047	0.693:0.247:0.014:0.046
Archosauromorpha	0.868:0.023:0.04:0.069	0.791:0.031:0.051:0.128	0.873:0.105:0.007:0.014	0.877:0.082:0.01:0.032	0.867:0.122:0.005:0.006	0.892:0.092:0.005:0.01
Archosauria	0.002:0.175:0.293:0.53	0.005:0.158:0.202:0.635	0.006:0.867:0.029:0.098	0.024:0.801:0.033:0.142	0.007:0.937:0.019:0.038	0.067:0.86:0.021:0.052
Dinosauria	0.001:0.295:0.306:0.398	0.002:0.284:0.214:0.5	0.004:0.89:0.03:0.076	0.015:0.838:0.033:0.113	0.007:0.945:0.019:0.028	0.053:0.885:0.021:0.042
Saurischia	0.001:0.281:0.329:0.389	0.001:0.27:0.247:0.482	0.003:0.88:0.046:0.072	0.011:0.832:0.046:0.11	0.002:0.942:0.029:0.027	0.014:0.918:0.028:0.04
Theropoda	0.016:0.01:0.014:0.961	0.015:0.009:0.013:0.963	0.031:0.031:0.017:0.921	0.062:0.043:0.017:0.878	0.038:0.051:0.017:0.894	0.092:0.064:0.019:0.825

Table S32g. Continuation of Table S32f.

Character	Reproduction mode + egg shell mineralisation			
Model	equal-ER (ind.hom)	equal-ER (ind.het)	equal-ER (sw.hom)	equal-ER (sw.het)
Mean log marginal likelihood	-71.530	-70.971	-72.16	-71.568
Mean log Bayes Factor	1.258	2.378	0	1.182
Amniota	0.89:0.019:0.033:0.058	0.884:0.02:0.033:0.063	0.825:0.033:0.062:0.081	0.82:0.033:0.057:0.09
Mammalia	0.727:0.145:0.064:0.064	0.721:0.149:0.065:0.065	0.724:0.146:0.065:0.065	0.714:0.151:0.068:0.068
Reptilia/Diapsida s.l.	0.811:0.017:0.061:0.111	0.827:0.015:0.052:0.106	0.792:0.019:0.081:0.109	0.803:0.017:0.069:0.111
Diapsida s.s.	0.022:0.047:0.314	0.071:0.04:0.28:0.	0.015:0.045:0.398	0.049:0.039:0.343

	:0.618	609	:0.543	:0.57
Lepidosauria	0:0.033:0.91:0.05 6	0:0.04:0.902:0.05 8	0:0.034:0.912:0.0 55	0:0.04:0.905:0.05 5
Archelosauria	0.362:0.048:0.025 :0.565	0.365:0.039:0.022 :0.574	0.253:0.059:0.043 :0.645	0.295:0.044:0.035 :0.626
Archosauromo rpha	0.91:0.036:0.015: 0.039	0.829:0.052:0.022 :0.096	0.856:0.055:0.03: 0.059	0.778:0.064:0.038 :0.12
Archosauria	0.002:0.425:0.044 :0.529	0.004:0.322:0.048 :0.625	0.002:0.44:0.047: 0.511	0.005:0.332:0.053 :0.61
Dinosauria	0.001:0.649:0.062 :0.288	0.002:0.528:0.071 :0.399	0.001:0.659:0.065 :0.275	0.002:0.541:0.075 :0.382
Saurischia	0.001:0.603:0.145 :0.251	0.001:0.498:0.151 :0.349	0.001:0.613:0.145 :0.241	0.001:0.51:0.155: 0.334
Theropoda	0.011:0.012:0.012 :0.964	0.01:0.011:0.011: 0.968	0.015:0.013:0.013 :0.958	0.014:0.012:0.012 :0.963

Table S33a. Same as Table S29 but excluding *Mesosaurus tenuidens* from the analyses (mbl dating method).

Model	Reproduction mode + egg shell mineralisation					
Character	mbl-CER (ind.hom)	mbl-CER (ind.het)	mbl-CER (sw.hom)	mbl-CER (sw.het)	mbl-CSYM (ind.hom)	mbl-CSYM (ind.het)
Mean log marginal likelihood	-71.076	-70.063	-71.721	-70.480	-72.980	-71.817
Mean log Bayes Factor	2.413	4.440	1.124	3.606	-1.394	0.932
Amniota	0.833:0.071:0.047:0.048	0.821:0.069:0.054:0.056	0.822:0.075:0.05:0.054	0.804:0.075:0.058:0.063	0.836:0.069:0.048:0.047	0.829:0.065:0.053:0.053
Mammalia	0.983:0.008:0.005:0.005	0.978:0.01:0.006:0.006	0.983:0.008:0.005:0.005	0.975:0.011:0.007:0.007	0.983:0.008:0.005:0.005	0.977:0.01:0.007:0.006
Reptilia/ Diapsida s.l.	0.998:0.001:0:0	0.997:0.001:0.001:0.001	0.998:0.001:0.001:0.001	0.996:0.001:0.001:0.001	0.998:0.001:0:0	0.997:0.001:0.001:0.001
Diapsida s.s.	0.858:0.082:0.03:0.031	0.864:0.067:0.034:0.036	0.809:0.094:0.055:0.042	0.833:0.074:0.049:0.043	0.844:0.084:0.038:0.033	0.849:0.068:0.042:0.04
Lepidosauria	0.139:0.047:0.164:0.65	0.203:0.067:0.217:0.513	0.114:0.051:0.213:0.622	0.2:0.073:0.245:0.483	0.136:0.048:0.153:0.663	0.191:0.077:0.199:0.533
Archelosauria	0.916:0.053:0.016:0.015	0.914:0.048:0.018:0.02	0.887:0.063:0.029:0.02	0.892:0.055:0.028:0.025	0.913:0.052:0.02:0.015	0.91:0.046:0.023:0.021
Archosauromorpha	0.955:0.03:0.009:0.006	0.954:0.028:0.01:0.008	0.95:0.03:0.013:0.007	0.946:0.029:0.015:0.01	0.955:0.029:0.01:0.006	0.953:0.027:0.012:0.008
Archosauria	0.017:0.838:0.1:0.045	0.031:0.769:0.123:0.077	0.017:0.829:0.1:0.054	0.034:0.748:0.127:0.092	0.018:0.817:0.121:0.044	0.034:0.731:0.154:0.081
Dinosauria	0.011:0.877:0.077:0.035	0.018:0.823:0.096:0.063	0.012:0.874:0.075:0.039	0.02:0.81:0.098:0.072	0.011:0.862:0.093:0.033	0.019:0.8:0.12:0.062
Saurischia	0.006:0.877:0.084:0.032	0.011:0.827:0.103:0.059	0.005:0.87:0.087:0.037	0.01:0.812:0.108:0.07	0.007:0.865:0.099:0.03	0.012:0.808:0.123:0.057

Theropoda	0.001:0.001:0.001:0.997	0.001:0.001:0.001:0.997	0.001:0.001:0.001:0.997	0.001:0.001:0.001:0.997	0.001:0.001:0.002:0.996	0.001:0.001:0.002:0.997

Table S33b. Continuation of Table S33a.

Character	Reproduction mode + egg shell mineralisation					
Model	mbl-CSYM (sw.hom)	mbl-CSYM (sw.het)	mbl-CARD (ind.hom)	mbl-CARD (ind.het)	mbl-CARD (sw.hom)	mbl-CARD (sw.het)
Mean log marginal likelihood	-73.278	-71.887	-68.007	-65.836	-70.375	-67.913
Mean log Bayes Factor	-1.990	0.792	8.551	12.894	3.816	8.739
Amniota	0.831:0.067:0.052:0.049	0.819:0.066:0.059:0.056	0.997:0.002:0.001:0	0.999:0.001:0:0	0.967:0.025:0.006:0.003	0.991:0.007:0.002:0.001
Mammalia	0.983:0.008:0.005:0.005	0.976:0.01:0.007:0.007	1:0:0:0	1:0:0:0	0.996:0.003:0.001:0	0.999:0.001:0:0
Reptilia/ Diapsida s.l.	0.998:0.001:0.001:0.001	0.996:0.001:0.001:0.001	1:0:0:0	1:0:0:0	0.999:0.001:0:0	1:0:0:0
Diapsida s.s.	0.788:0.1:0.066:0.046	0.812:0.079:0.059:0.05	0.998:0.001:0:0	0.999:0:0:0	0.937:0.054:0.006:0.003	0.982:0.015:0.002:0.001
Lepidosauria	0.105:0.055:0.196:0.644	0.184:0.088:0.229:0.499	0.987:0.001:0.002:0.009	0.995:0.001:0.001:0.003	0.711:0.019:0.068:0.202	0.917:0.008:0.024:0.051
Archelosauria	0.884:0.062:0.033:0.021	0.888:0.053:0.033:0.027	0.999:0.001:0:0	1:0:0:0	0.968:0.027:0.003:0.001	0.99:0.008:0.001:0.001
Archosauromorpha	0.95:0.028:0.014:0.007	0.948:0.026:0.015:0.01	0.999:0:0:0	1:0:0:0	0.987:0.012:0.002:0	0.995:0.004:0:0
Archosauria	0.016:0.802:0.13:0.052	0.031:0.71:0.162:0.097	0.02:0.868:0.105:0.008	0.062:0.798:0.117:0.022	0.038:0.873:0.082:0.008	0.153:0.723:0.103:0.02
Dinosauria	0.011:0.856:0.096:0.036	0.018:0.783:0.125:0.073	0.013:0.903:0.079:0.005	0.041:0.853:0.093:0.014	0.024:0.91:0.062:0.005	0.096:0.809:0.083:0.013
Saurischi	0.005:0.858	0.009:0.789	0.008:0.907	0.028:0.873	0.006:0.927	0.027:0.883

a	:0.104:0.03 4	:0.131:0.07 1	:0.08:0.004	:0.088:0.01 1	:0.063:0.00 4	:0.08:0.011
Theropod a	0.001:0.001 :0.003:0.99 4	0.001:0.001 :0.002:0.99 5	0.001:0.001 :0.001:0.99 6	0.004:0.004 :0.004:0.98 9	0.001:0.003 :0.002:0.99 4	0.004:0.005 :0.004:0.98 7

Table S33c. Continuation of Table S33b.

Character	Reproduction mode + egg shell mineralisation			
Model	mbl-ER (ind.hom)	mbl-ER (ind.het)	mbl-ER (sw.hom)	mbl-ER (sw.het)
Mean log marginal likelihood	-71.461	-70.427	-72.283	-71.032
Mean log Bayes Factor	1.643	3.712	0	2.501
Amniota	0.818:0.077:0.052 :0.053	0.791:0.079:0.064 :0.066	0.801:0.084:0.055 :0.06	0.768:0.088:0.069 :0.075
Mammalia	0.983:0.008:0.005 :0.005	0.975:0.011:0.007 :0.007	0.982:0.008:0.005 :0.005	0.971:0.012:0.008 :0.008
Reptilia/Diapsida s.l.	0.998:0.001:0:0	0.996:0.002:0.001 :0.001	0.998:0.001:0.001 :0.001	0.995:0.002:0.001 :0.002
Diapsida s.s.	0.874:0.073:0.026 :0.027	0.875:0.062:0.03: 0.034	0.835:0.082:0.046 :0.037	0.848:0.068:0.043 :0.04
Lepidosauria	0.155:0.047:0.15: 0.648	0.238:0.068:0.19: 0.505	0.131:0.052:0.196 :0.621	0.234:0.07:0.223: 0.473
Archelosauria	0.921:0.05:0.015: 0.014	0.915:0.047:0.018 :0.02	0.896:0.058:0.027 :0.019	0.895:0.053:0.027 :0.025
Archosauromorpha	0.955:0.03:0.009: 0.006	0.951:0.029:0.011 :0.009	0.95:0.03:0.013:0. 007	0.945:0.03:0.015: 0.01
Archosauria	0.019:0.835:0.101 :0.045	0.041:0.757:0.123 :0.079	0.021:0.824:0.1:0. 054	0.043:0.74:0.124: 0.092
Dinosauria	0.012:0.874:0.079 :0.035	0.022:0.817:0.098 :0.063	0.014:0.87:0.076: 0.04	0.024:0.805:0.098 :0.073
Saurischia	0.007:0.876:0.085 :0.032	0.014:0.824:0.103 :0.059	0.006:0.87:0.086: 0.038	0.011:0.811:0.107 :0.071
Theropoda	0.001:0.001:0.001 :0.997	0.001:0.001:0.001 :0.997	0.001:0.001:0.001 :0.996	0.001:0.001:0.001 :0.997

Table S33d. Continuation of Table S33c.

Character	EER			
Model	mbl-EER ER (hom)	mbl-EER ER (het)	mbl-EER ARD (hom)	mbl-EER ARD (het)
Mean log marginal	-34.079	-33.100	-32.891	-32.072

likelihood				
Mean log Bayes Factor	0	1.957	2.377	4.014
Amniota	0.129:0.871	0.152:0.848	0.054:0.946	0.074:0.926
Mammalia	0.016:0.984	0.024:0.976	0.007:0.993	0.013:0.987
Reptilia/Diapsida s.l.	0.001:0.999	0.002:0.998	0:1	0.001:0.999
Diapsida s.s.	0.058:0.942	0.081:0.919	0.028:0.972	0.045:0.955
Lepidosauria	0.195:0.805	0.287:0.713	0.076:0.924	0.146:0.854
Archelosauria	0.102:0.898	0.106:0.894	0.054:0.946	0.064:0.936
Archosauromorpha	0.043:0.957	0.05:0.95	0.021:0.979	0.028:0.972
Archosauria	0.986:0.014	0.986:0.014	0.993:0.007	0.979:0.021
Dinosauria	0.991:0.009	0.992:0.008	0.996:0.004	0.987:0.013
Saurischia	0.995:0.005	0.995:0.005	0.997:0.003	0.991:0.009
Theropoda	0.999:0.001	0.999:0.001	1:0	0.998:0.002

Table S33e. Continuation of Table S33d. Nodes of Lepidosauria and Squamata fixed to a non-viviparous state.

Character	Reproduction mode + egg shell mineralisation					
Model	mbl-CER (ind.hom)	mbl-CER (ind.het)	mbl-CER (sw.hom)	mbl-CER (sw.het)	mbl-CSYM (ind.hom)	mbl-CSYM (ind.het)
Mean log marginal likelihood	-71.291	-70.361	-71.847	-70.732	-73.180	-72.112
Mean log Bayes Factor	2.265	4.127	1.153	3.384	-1.512	0.623
Amniota	0.839:0.069:0.045:0.046	0.83:0.066:0.051:0.053	0.826:0.077:0.045:0.051	0.817:0.076:0.05:0.058	0.844:0.065:0.046:0.044	0.842:0.06:0.049:0.049
Mammalia	0.984:0.007:0.004:0.004	0.977:0.01:0.006:0.006	0.983:0.008:0.005:0.005	0.976:0.011:0.007:0.007	0.984:0.007:0.005:0.004	0.978:0.01:0.006:0.006
Reptilia/ Diapsida s.l.	0.998:0.001:0:0	0.997:0.001:0.001:0.001	0.998:0.001:0.001:0.001	0.996:0.002:0.001:0.001	0.998:0.001:0:0	0.997:0.001:0.001:0.001
Diapsida s.s.	0.843:0.09:0.033:0.034	0.843:0.076:0.038:0.043	0.797:0.101:0.057:0.045	0.819:0.082:0.052:0.047	0.826:0.094:0.043:0.037	0.826:0.078:0.049:0.047
Lepidosauria	0:0.055:0.19:0.755	0:0.083:0.273:0.644	0:0.06:0.233:0.707	0:0.093:0.313:0.595	0:0.055:0.178:0.767	0:0.094:0.254:0.652
Archelos	0.915:0.054	0.91:0.049:	0.887:0.063	0.893:0.055	0.911:0.054	0.907:0.047

auria	:0.016:0.015	0.019:0.022	:0.029:0.02	:0.027:0.025	:0.02:0.015	:0.023:0.023
Archosauromorpha	0.955:0.03:0.009:0.006	0.953:0.028:0.01:0.008	0.95:0.03:0.013:0.007	0.949:0.028:0.014:0.009	0.956:0.029:0.01:0.006	0.955:0.026:0.011:0.008
Archosauria	0.016:0.84:0.1:0.044	0.03:0.774:0.122:0.074	0.017:0.829:0.1:0.054	0.033:0.757:0.123:0.088	0.016:0.82:0.122:0.043	0.029:0.735:0.153:0.083
Dinosauria	0.01:0.879:0.076:0.035	0.017:0.829:0.095:0.059	0.012:0.873:0.076:0.039	0.018:0.819:0.095:0.067	0.01:0.865:0.092:0.032	0.017:0.801:0.118:0.064
Saurischia	0.006:0.877:0.085:0.032	0.01:0.831:0.102:0.056	0.005:0.87:0.087:0.038	0.008:0.821:0.106:0.065	0.006:0.866:0.098:0.03	0.01:0.809:0.121:0.06
Theropoda	0.001:0.001:0.001:0.998	0.001:0.001:0.001:0.998	0.001:0.001:0.001:0.996	0.001:0.001:0.001:0.997	0.001:0.001:0.002:0.996	0.001:0.001:0.002:0.996

Table S33f. Continuation of Table S33e.

Character	Reproduction mode + egg shell mineralisation					
Model	mbl-CSYM (sw.hom)	mbl-CSYM (sw.het)	mbl-CARD (ind.hom)	mbl-CARD (ind.het)	mbl-CARD (sw.hom)	mbl-CARD (sw.het)
Mean log marginal likelihood	-73.398	-72.111	-72.554	-71.748	-71.532	-70.496
Mean log Bayes Factor	-1.949	0.625	-0.260	1.352	1.783	3.855
Amniota	0.839:0.068:0.046:0.046	0.834:0.065:0.049:0.052	0.828:0.097:0.048:0.028	0.857:0.075:0.039:0.029	0.903:0.074:0.016:0.007	0.918:0.059:0.014:0.009
Mammalia	0.983:0.008:0.005:0.005	0.977:0.01:0.007:0.006	0.984:0.009:0.004:0.003	0.981:0.011:0.005:0.003	0.989:0.008:0.002:0.001	0.987:0.01:0.002:0.001
Reptilia/ Diapsida s.l.	0.998:0.001:0.001:0.001	0.996:0.002:0.001:0.001	0.998:0.001:0:0	0.998:0.002:0.001:0	0.998:0.002:0:0	0.997:0.002:0:0
Diapsida s.s.	0.769:0.11:0.07:0.05	0.792:0.088:0.065:0.055	0.872:0.098:0.02:0.009	0.869:0.095:0.022:0.014	0.806:0.168:0.019:0.008	0.848:0.129:0.015:0.008
Lepidosa	0:0.063:0.2	0:0.111:0.2	0:0.065:0.1	0:0.095:0.2	0:0.067:0.2	0:0.109:0.2

uria	19:0.718	81:0.608	69:0.766	37:0.668	34:0.699	66:0.625
Archelos auria	0.881:0.063 :0.034:0.02 1	0.887:0.054 :0.033:0.02 7	0.927:0.057 :0.012:0.00 4	0.924:0.058 :0.012:0.00 7	0.903:0.084 :0.009:0.00 3	0.916:0.072 :0.008:0.00 4
Archosau romorpha	0.951:0.028 :0.014:0.00 7	0.949:0.026 :0.015:0.01	0.959:0.032 :0.007:0.00 2	0.958:0.032 :0.007:0.00 3	0.96:0.035: 0.004:0.001	0.962:0.033 :0.004:0.00 2
Archosau ria	0.016:0.803 :0.129:0.05 3	0.027:0.716 :0.159:0.09 9	0.022:0.886 :0.084:0.00 8	0.039:0.854 :0.089:0.01 8	0.039:0.917 :0.039:0.00 5	0.074:0.869 :0.045:0.01 2
Dinosauri a	0.011:0.856 :0.096:0.03 7	0.015:0.789 :0.122:0.07 4	0.014:0.917 :0.064:0.00 5	0.024:0.893 :0.07:0.013	0.023:0.945 :0.029:0.00 3	0.042:0.915 :0.034:0.00 9
Saurischi a	0.004:0.857 :0.104:0.03 5	0.007:0.796 :0.127:0.07	0.008:0.918 :0.069:0.00 5	0.016:0.897 :0.075:0.01 3	0.006:0.957 :0.034:0.00 3	0.014:0.938 :0.038:0.00 9
Theropod a	0.001:0.002 :0.003:0.99 4	0.001:0.002 :0.003:0.99 5	0.001:0.001 :0.001:0.99 6	0.002:0.002 :0.002:0.99 5	0.001:0.002 :0.001:0.99 6	0.002:0.002 :0.002:0.99 4

Table S33g. Continuation of Table S33f.

Character	Reproduction mode + egg shell mineralisation			
Model	mbl-ER (ind.hom)	mbl-ER (ind.het)	mbl-ER (sw.hom)	mbl-ER (sw.het)
Mean log marginal likelihood	-71.697	-70.775	-72.424	-71.317
Mean log Bayes Factor	1.455	3.297	0	2.213
Amniota	0.824:0.075:0.05: 0.051	0.803:0.076:0.06: 0.062	0.811:0.084:0.049 :0.056	0.78:0.09:0.06:0.0 71
Mammalia	0.983:0.008:0.005 :0.005	0.976:0.01:0.007: 0.007	0.982:0.008:0.005 :0.005	0.973:0.012:0.008 :0.008
Reptilia/Diaps ida s.l.	0.998:0.001:0:0	0.996:0.002:0.001 :0.001	0.997:0.001:0.001 :0.001	0.995:0.002:0.002 :0.002
Diapsida s.s.	0.859:0.081:0.029 :0.03	0.855:0.071:0.035 :0.039	0.817:0.092:0.051 :0.04	0.834:0.076:0.046 :0.043
Lepidosauria	0:0.057:0.176:0.7 68	0:0.088:0.249:0.6 64	0:0.059:0.23:0.71 1	0:0.093:0.291:0.6 15
Archelosauria	0.92:0.051:0.015: 0.014	0.913:0.048:0.018 :0.021	0.892:0.061:0.028 :0.019	0.895:0.054:0.027 :0.025
Archosauromo rpha	0.955:0.03:0.009: 0.006	0.952:0.029:0.011 :0.008	0.949:0.03:0.014: 0.007	0.946:0.03:0.014: 0.01

Archosauria	0.018:0.836:0.101:0.044	0.038:0.764:0.122:0.076	0.02:0.827:0.1:0.053	0.04:0.745:0.123:0.092
Dinosauria	0.012:0.876:0.078:0.035	0.021:0.824:0.095:0.06	0.013:0.873:0.075:0.039	0.024:0.806:0.096:0.074
Saurischia	0.007:0.875:0.086:0.032	0.013:0.83:0.102:0.056	0.006:0.87:0.087:0.037	0.011:0.81:0.107:0.073
Theropoda	0.001:0.001:0.001:0.997	0.001:0.001:0.001:0.998	0.001:0.001:0.001:0.997	0.001:0.001:0.001:0.997

Table S34a. Same as Table S30 but excluding *Mesosaurus tenuidens* from the analyses (FBD tip-dating method with root age constrained).

Character	Reproduction mode + egg shell mineralisation					
Model	FBD-CER (ind.hom)	FBD-CER (ind.het)	FBD-CER (sw.hom)	FBD-CER (sw.het)	FBD-CSYM (ind.hom)	FBD-CSYM (ind.het)
Mean log marginal likelihood	-69.704	-69.236	-70.438	-69.830	-71.098	-70.644
Mean log Bayes Factor	3.224	4.158	1.754	2.972	0.434	1.342
Amniota	0.903:0.025:0.036:0.036	0.89:0.028:0.038:0.044	0.813:0.05:0.088:0.049	0.811:0.047:0.082:0.06	0.9:0.021:0.041:0.039	0.892:0.023:0.038:0.048
Mammalia	0.803:0.119:0.039:0.039	0.795:0.124:0.041:0.041	0.801:0.117:0.041:0.041	0.793:0.119:0.044:0.044	0.814:0.125:0.03:0.031	0.801:0.132:0.032:0.035
Reptilia/ Diapsida s.l.	0.879:0.02:0.049:0.051	0.879:0.019:0.046:0.057	0.851:0.021:0.074:0.053	0.844:0.021:0.071:0.064	0.878:0.012:0.058:0.051	0.882:0.012:0.049:0.057
Diapsida s.s.	0.169:0.131:0.357:0.344	0.238:0.108:0.301:0.353	0.15:0.108:0.466:0.276	0.217:0.093:0.392:0.298	0.166:0.071:0.413:0.351	0.223:0.063:0.343:0.371
Lepidosauria	0.072:0.039:0.801:0.088	0.098:0.047:0.768:0.087	0.148:0.037:0.732:0.083	0.184:0.043:0.697:0.076	0.083:0.032:0.787:0.098	0.105:0.038:0.772:0.085
Archelosauria	0.421:0.138:0.086:0.354	0.449:0.111:0.073:0.367	0.254:0.165:0.184:0.397	0.333:0.127:0.146:0.395	0.406:0.071:0.157:0.366	0.428:0.062:0.111:0.398
Archosauromorpha	0.771:0.103:0.06:0.066	0.746:0.098:0.057:0.098	0.656:0.131:0.128:0.085	0.645:0.123:0.115:0.118	0.758:0.055:0.098:0.089	0.734:0.057:0.082:0.127
Archosauria	0.009:0.582:0.085:0.324	0.019:0.464:0.099:0.418	0.009:0.582:0.092:0.318	0.021:0.467:0.109:0.403	0.01:0.296:0.268:0.425	0.02:0.256:0.204:0.52
Dinosauria	0.004:0.667:0.105:0.224	0.007:0.574:0.113:0.306	0.005:0.664:0.112:0.22	0.007:0.571:0.123:0.298	0.004:0.379:0.278:0.338	0.007:0.351:0.21:0.431
Saurischia	0.002:0.622:0.193:0.18	0.004:0.543:0.195:0.25	0.002:0.624:0.197:0.17	0.003:0.544:0.202:0.25	0.002:0.357:0.318:0.32	0.004:0.333:0.257:0.40

	3	9	8		3	7
Theropoda	0.003:0.003:0.003:0.991	0.003:0.003:0.003:0.99	0.004:0.004:0.004:0.989	0.005:0.004:0.004:0.988	0.004:0.003:0.004:0.989	0.003:0.003:0.004:0.99

Table S34b. Continuation of Table S34a.

Character	Reproduction mode + egg shell mineralisation					
Model	FBD-CSYM (sw.hom)	FBD-CSYM (sw.het)	FBD-CARD (ind.hom)	FBD-CARD (ind.het)	FBD-CARD (sw.hom)	FBD-CARD (sw.het)
Mean log marginal likelihood	-71.447	-70.910	-64.855	-63.596	-65.394	-64.354
Mean log Bayes Factor	-0.264	0.812	12.920	15.439	11.842	13.922
Amniota	0.819:0.039:0.099:0.043	0.817:0.039:0.084:0.059	0.999:0:0:0	1:0:0:0	1:0:0:0	0.999:0:0:0
Mammalia	0.819:0.119:0.036:0.025	0.806:0.123:0.037:0.033	0.999:0.001:0:0	1:0:0:0	0.999:0:0:0	0.999:0.001:0:0
Reptilia/ Diapsida s.l.	0.873:0.012:0.073:0.042	0.86:0.014:0.07:0.057	0.999:0:0:0	1:0:0:0	0.999:0.001:0:0	0.999:0.001:0:0
Diapsida s.s.	0.195:0.052:0.532:0.221	0.241:0.057:0.416:0.286	0.997:0.002:0.001:0	0.999:0.001:0:0	0.997:0.003:0:0	0.996:0.003:0.001:0
Lepidosauria	0.181:0.037:0.666:0.116	0.199:0.041:0.671:0.089	0.997:0:0.002:0	0.999:0:0:0	0.997:0:0.002:0	0.995:0.001:0.003:0.001
Archelosauria	0.286:0.074:0.324:0.316	0.354:0.07:0.207:0.369	0.998:0.001:0:0.001	0.999:0.001:0:0	0.997:0.003:0:0	0.996:0.003:0:0
Archosauromorpha	0.694:0.062:0.17:0.074	0.667:0.068:0.138:0.127	0.999:0.001:0:0	1:0:0:0	0.998:0.001:0:0	0.998:0.002:0:0
Archosauria	0.01:0.258:0.461:0.271	0.022:0.257:0.311:0.41	0.028:0.856:0.093:0.024	0.044:0.857:0.053:0.046	0.024:0.72:0.231:0.025	0.094:0.658:0.205:0.043
Dinosauri	0.005:0.338	0.008:0.35:	0.017:0.871	0.023:0.89:	0.021:0.728	0.066:0.71:

a	:0.461:0.196	0.317:0.324	:0.093:0.02	0.052:0.036	:0.23:0.021	0.194:0.03
Saurischia	0.002:0.33:0.489:0.179	0.003:0.338:0.358:0.302	0.009:0.862:0.109:0.02	0.015:0.895:0.058:0.032	0.005:0.7:0.274:0.021	0.022:0.736:0.215:0.028
Theropoda	0.005:0.002:0.005:0.988	0.005:0.003:0.005:0.987	0.012:0.013:0.011:0.964	0.019:0.017:0.016:0.948	0.014:0.013:0.013:0.959	0.048:0.033:0.028:0.891

Table S34c. Continuation of Table S34b.

Character	Reproduction mode + egg shell mineralisation			
Model	FBD-ER (ind.hom)	FBD-ER (ind.het)	FBD-ER (sw.hom)	FBD-ER (sw.het)
Mean log marginal likelihood	-70.402	-69.949	-71.315	-70.694
Mean log Bayes Factor	1.827	2.734	0	1.243
Amniota	0.903:0.026:0.036:0.036	0.889:0.028:0.037:0.046	0.813:0.049:0.09:0.048	0.813:0.047:0.081:0.059
Mammalia	0.8:0.121:0.04:0.04	0.797:0.121:0.041:0.041	0.802:0.116:0.041:0.041	0.794:0.118:0.044:0.044
Reptilia/Diapsida s.l.	0.881:0.02:0.048:0.05	0.877:0.019:0.045:0.058	0.854:0.021:0.074:0.051	0.851:0.021:0.068:0.061
Diapsida s.s.	0.175:0.134:0.354:0.338	0.239:0.109:0.298:0.353	0.155:0.108:0.468:0.269	0.231:0.093:0.381:0.295
Lepidosauria	0.072:0.039:0.799:0.09	0.105:0.05:0.756:0.09	0.154:0.039:0.721:0.085	0.198:0.044:0.679:0.079
Archelosauria	0.43:0.14:0.086:0.344	0.444:0.112:0.075:0.369	0.258:0.167:0.187:0.387	0.348:0.126:0.145:0.381
Archosauromorpha	0.773:0.103:0.059:0.064	0.736:0.101:0.06:0.102	0.658:0.131:0.129:0.082	0.649:0.122:0.114:0.116
Archosauria	0.009:0.588:0.085:0.319	0.02:0.462:0.099:0.419	0.009:0.585:0.094:0.312	0.021:0.467:0.111:0.402
Dinosauria	0.004:0.67:0.105:0.22	0.008:0.568:0.113:0.311	0.005:0.665:0.113:0.217	0.009:0.569:0.126:0.297
Saurischia	0.002:0.626:0.192:0.179	0.004:0.541:0.193:0.263	0.002:0.627:0.198:0.174	0.003:0.542:0.205:0.25
Theropoda	0.003:0.003:0.003:0.991	0.003:0.003:0.003:0.991	0.004:0.004:0.004:0.988	0.004:0.004:0.004:0.989

Table S34d. Continuation of Table S34c.

Character	EER			
Model	FBD-EER ER (hom)	FBD-EER ER (het)	FBD-EER ARD (hom)	FBD-EER ARD (het)
Mean log marginal likelihood	-31.671	-31.603	-28.298	-28.213
Mean log Bayes Factor	0	0.136	6.746	6.916
Amniota	0.035:0.965	0.047:0.953	0.002:0.998	0.004:0.996
Mammalia	0.193:0.807	0.198:0.802	0.013:0.987	0.015:0.985
Reptilia/Diapsida s.l.	0.015:0.985	0.025:0.975	0.001:0.999	0.003:0.997
Diapsida s.s.	0.36:0.64	0.351:0.649	0.026:0.974	0.03:0.97
Lepidosauria	0.134:0.866	0.167:0.833	0.009:0.991	0.013:0.987
Archelosauria	0.665:0.335	0.59:0.41	0.049:0.951	0.049:0.951
Archosauromorpha	0.23:0.77	0.236:0.764	0.017:0.983	0.02:0.98
Archosauria	0.993:0.007	0.988:0.012	0.989:0.011	0.959:0.041
Dinosauria	0.997:0.003	0.997:0.003	0.996:0.004	0.981:0.019
Saurischia	0.999:0.001	0.999:0.001	0.999:0.001	0.991:0.009
Theropoda	0.993:0.007	0.994:0.006	0.988:0.012	0.962:0.038

Table S34e. Continuation of Table S34d. Nodes of Lepidosauria and Squamata fixed to a non-viviparous state.

Character	Reproduction mode + egg shell mineralisation					
Model	FBD-CER (ind.hom)	FBD-CER (ind.het)	FBD-CER (sw.hom)	FBD-CER (sw.het)	FBD-CSYM (ind.hom)	FBD-CSYM (ind.het)
Mean log marginal likelihood	-69.943	-69.522	-70.778	-70.153	-71.385	-70.934
Mean log Bayes Factor	3.442	4.283	1.771	3.020	0.558	1.460
Amniota	0.887:0.029:0.042:0.042	0.875:0.03:0.043:0.052	0.771:0.065:0.1:0.064	0.776:0.06:0.087:0.078	0.882:0.024:0.048:0.046	0.869:0.026:0.046:0.059
Mammalia	0.795:0.126:0.04:0.04	0.796:0.126:0.039:0.039	0.798:0.121:0.04:0.04	0.793:0.124:0.042:0.042	0.803:0.134:0.031:0.032	0.798:0.135:0.031:0.036
Reptilia/ Diapsida s.l.	0.852:0.025:0.06:0.063	0.847:0.022:0.058:0.073	0.791:0.03:0.105:0.074	0.791:0.029:0.092:0.088	0.846:0.015:0.074:0.065	0.842:0.015:0.067:0.076
Diapsida	0.136:0.135	0.178:0.106	0.086:0.117	0.13:0.103:	0.128:0.072	0.172:0.062

s.s.	:0.368:0.361	:0.327:0.389	:0.491:0.307	0.409:0.358	:0.429:0.371	:0.355:0.411
Lepidosauria	0:0.036:0.877:0.087	0:0.046:0.869:0.085	0:0.041:0.867:0.093	0:0.05:0.861:0.09	0:0.031:0.872:0.097	0:0.035:0.879:0.086
Archelosauria	0.43:0.132:0.081:0.356	0.435:0.102:0.07:0.394	0.252:0.161:0.176:0.411	0.317:0.123:0.13:0.431	0.413:0.068:0.149:0.37	0.418:0.058:0.105:0.419
Archosauromorpha	0.778:0.099:0.056:0.067	0.743:0.094:0.057:0.106	0.66:0.13:0.123:0.087	0.634:0.126:0.108:0.131	0.767:0.052:0.092:0.089	0.725:0.055:0.081:0.139
Archosauria	0.008:0.579:0.08:0.334	0.017:0.446:0.092:0.445	0.008:0.578:0.086:0.328	0.017:0.463:0.097:0.423	0.009:0.301:0.25:0.439	0.018:0.253:0.188:0.541
Dinosauria	0.004:0.666:0.102:0.229	0.006:0.562:0.108:0.324	0.004:0.667:0.106:0.223	0.007:0.57:0.114:0.309	0.004:0.385:0.263:0.347	0.007:0.349:0.198:0.446
Saurischia	0.002:0.621:0.191:0.186	0.004:0.533:0.19:0.273	0.001:0.627:0.191:0.181	0.003:0.542:0.195:0.261	0.002:0.361:0.305:0.332	0.004:0.329:0.242:0.424
Theropoda	0.003:0.003:0.003:0.991	0.003:0.003:0.003:0.991	0.004:0.003:0.003:0.989	0.004:0.003:0.003:0.989	0.003:0.002:0.004:0.99	0.004:0.003:0.004:0.99

Table S34f. Continuation of Table S34e.

Character	Reproduction mode + egg shell mineralisation					
Model	FBD-CSYM (sw.hom)	FBD-CSYM (sw.het)	FBD-CARD (ind.hom)	FBD-CARD (ind.het)	FBD-CARD (sw.hom)	FBD-CARD (sw.het)
Mean log marginal likelihood	-71.929	-71.321	-70.980	-70.742	-70.566	-70.197
Mean log Bayes Factor	-0.530	0.686	1.367	1.843	2.196	2.933
Amniota	0.759:0.057:0.114:0.069	0.77:0.051:0.092:0.087	0.865:0.104:0.018:0.014	0.872:0.084:0.019:0.024	0.863:0.109:0.018:0.009	0.9:0.072:0.015:0.013
Mammalia	0.812:0.126:0.035:0.028	0.802:0.129:0.035:0.035	0.786:0.189:0.015:0.01	0.784:0.185:0.015:0.016	0.856:0.128:0.009:0.006	0.866:0.118:0.009:0.007
Reptilia/	0.793:0.02:	0.794:0.02:	0.85:0.107:	0.864:0.082	0.762:0.199	0.845:0.118

Diapsida s.l.	0.116:0.071	0.098:0.088	0.025:0.018	:0.025:0.029	:0.026:0.013	:0.021:0.016
Diapsida s.s.	0.089:0.066:0.555:0.29	0.127:0.067:0.445:0.361	0.204:0.574:0.134:0.087	0.296:0.44:0.129:0.135	0.136:0.705:0.114:0.044	0.306:0.544:0.089:0.061
Lepidosauria	0:0.039:0.847:0.114	0:0.045:0.858:0.097	0:0.061:0.755:0.184	0:0.1:0.702:0.198	0:0.085:0.744:0.171	0:0.135:0.676:0.189
Archelosauria	0.267:0.084:0.271:0.378	0.322:0.073:0.18:0.425	0.476:0.407:0.044:0.073	0.516:0.323:0.04:0.121	0.272:0.629:0.054:0.046	0.444:0.459:0.037:0.06
Archosauromorpha	0.679:0.07:0.15:0.1	0.642:0.072:0.126:0.16	0.76:0.199:0.024:0.018	0.744:0.19:0.027:0.039	0.592:0.368:0.027:0.013	0.673:0.283:0.022:0.022
Archosauria	0.01:0.294:0.351:0.345	0.018:0.259:0.245:0.478	0.014:0.872:0.044:0.07	0.024:0.781:0.056:0.139	0.019:0.918:0.03:0.033	0.048:0.858:0.034:0.059
Dinosauria	0.004:0.38:0.36:0.256	0.007:0.353:0.254:0.386	0.007:0.89:0.045:0.059	0.01:0.818:0.054:0.117	0.012:0.932:0.03:0.025	0.029:0.89:0.033:0.048
Saurischia	0.001:0.364:0.396:0.239	0.003:0.336:0.297:0.364	0.003:0.869:0.071:0.057	0.006:0.805:0.076:0.113	0.003:0.924:0.049:0.025	0.006:0.899:0.047:0.047
Theropoda	0.005:0.003:0.005:0.987	0.004:0.003:0.005:0.988	0.004:0.004:0.004:0.988	0.007:0.006:0.004:0.983	0.008:0.008:0.005:0.979	0.014:0.011:0.005:0.97

Table S34g. Continuation of Table S34f.

Character	Reproduction mode + egg shell mineralisation			
Model	FBD-ER (ind.hom)	FBD-ER (ind.het)	FBD-ER (sw.hom)	FBD-ER (sw.het)
Mean log marginal likelihood	-70.662	-70.248	-71.664	-71.070
Mean log Bayes Factor	2.004	2.832	0	1.186
Amniota	0.884:0.031:0.043:0.043	0.871:0.032:0.044:0.053	0.77:0.066:0.101:0.063	0.78:0.059:0.084:0.077
Mammalia	0.794:0.125:0.041:0.041	0.789:0.127:0.042:0.042	0.8:0.12:0.04:0.04	0.794:0.121:0.043:0.043
Reptilia/Diapsida s.l.	0.85:0.026:0.061:0.063	0.847:0.024:0.057:0.072	0.791:0.031:0.105:0.073	0.797:0.028:0.088:0.087
Diapsida s.s.	0.141:0.141:0.365	0.189:0.112:0.32:	0.087:0.118:0.493	0.133:0.103:0.41:

	:0.353	0.38	:0.303	0.354
Lepidosauria	0:0.04:0.863:0.09<br>7	0:0.047:0.863:0.0<br>9	0:0.042:0.864:0.0<br>94	0:0.05:0.858:0.09<br>2
Archelosauria	0.437:0.138:0.084<br>:0.342	0.443:0.108:0.071<br>:0.378	0.255:0.163:0.177<br>:0.405	0.318:0.123:0.132<br>:0.427
Archosauromo<br>rpha	0.777:0.101:0.058<br>:0.064	0.743:0.098:0.058<br>:0.101	0.663:0.13:0.123:<br>0.085	0.631:0.127:0.111<br>:0.132
Archosauria	0.008:0.59:0.083:<br>0.32	0.017:0.46:0.094:<br>0.429	0.008:0.583:0.087<br>:0.322	0.017:0.462:0.101<br>:0.42
Dinosauria	0.004:0.671:0.104<br>:0.221	0.007:0.57:0.109:<br>0.314	0.004:0.671:0.106<br>:0.218	0.007:0.576:0.115<br>:0.301
Saurischia	0.002:0.626:0.192<br>:0.18	0.004:0.542:0.189<br>:0.265	0.001:0.63:0.191:<br>0.177	0.003:0.547:0.195<br>:0.256
Theropoda	0.003:0.003:0.003<br>:0.991	0.003:0.003:0.003<br>:0.991	0.004:0.003:0.003<br>:0.989	0.004:0.003:0.003<br>:0.989

Table S35a. Same as Table S31 but excluding *Mesosaurus tenuidens* from the analyses (FBD tip-dating method with root age and node age of major extant clades constrained).

Character	Reproduction mode + egg shell mineralisation					
Model	FBD_c-CER (ind.hom)	FBD_c-CER (ind.het)	FBD_c-CER (sw.hom)	FBD_c-CER (sw.het)	FBD_c-CSYM (ind.hom)	FBD_c-CSYM (ind.het)
Mean log marginal likelihood	-69.938	-69.399	-70.347	-69.748	-71.210	-70.670
Mean log Bayes Factor	2.610	3.690	1.793	2.990	0.068	1.146
Amniota	0.827:0.038:0.065:0.07	0.831:0.036:0.057:0.076	0.737:0.066:0.117:0.081	0.751:0.058:0.098:0.093	0.828:0.031:0.064:0.077	0.828:0.032:0.056:0.084
Mammalia	0.773:0.125:0.051:0.051	0.753:0.135:0.056:0.056	0.77:0.126:0.052:0.052	0.747:0.137:0.058:0.058	0.775:0.144:0.04:0.041	0.759:0.15:0.043:0.048
Reptilia/Diapsida s.l.	0.739:0.034:0.11:0.117	0.78:0.027:0.082:0.112	0.724:0.034:0.141:0.102	0.756:0.028:0.107:0.109	0.743:0.02:0.114:0.123	0.776:0.018:0.087:0.119
Diapsida s.s.	0.055:0.108:0.399:0.437	0.113:0.091:0.328:0.467	0.057:0.095:0.503:0.346	0.118:0.081:0.41:0.391	0.06:0.049:0.433:0.458	0.118:0.05:0.35:0.481
Lepidosauria	0.098:0.069:0.735:0.099	0.131:0.078:0.688:0.103	0.177:0.064:0.67:0.089	0.212:0.067:0.635:0.086	0.105:0.048:0.741:0.106	0.136:0.055:0.704:0.105
Archelosauria	0.219:0.145:0.094:0.542	0.271:0.11:0.08:0.539	0.14:0.159:0.159:0.541	0.214:0.119:0.131:0.536	0.219:0.065:0.178:0.539	0.269:0.059:0.125:0.547
Archosauromorpha	0.609:0.155:0.091:0.145	0.593:0.13:0.083:0.194	0.498:0.181:0.163:0.159	0.503:0.147:0.139:0.211	0.602:0.072:0.142:0.184	0.593:0.068:0.112:0.228
Archosauria	0.006:0.476:0.083:0.435	0.013:0.367:0.094:0.526	0.006:0.493:0.09:0.411	0.016:0.377:0.102:0.505	0.006:0.214:0.253:0.526	0.015:0.189:0.195:0.601
Dinosauria	0.005:0.54:0.11:0.345	0.008:0.45:0.114:0.428	0.006:0.555:0.117:0.322	0.009:0.459:0.124:0.407	0.006:0.281:0.262:0.451	0.009:0.257:0.205:0.529
Saurischia	0.003:0.517:0.199:0.28	0.004:0.436:0.195:0.36	0.002:0.529:0.202:0.26	0.004:0.444:0.203:0.35	0.003:0.261:0.303:0.43	0.005:0.242:0.248:0.50

	1	5	7		3	4
Theropoda	0.005:0.005:0.005:0.984	0.005:0.005:0.005:0.985	0.007:0.006:0.006:0.982	0.006:0.005:0.005:0.983	0.006:0.004:0.006:0.984	0.005:0.004:0.007:0.984

Table S35b. Continuation of Table S35a.

Character	Reproduction mode + egg shell mineralisation					
Model	FBD_c-CSYM (sw.hom)	FBD_c-CSYM (sw.het)	FBD_c-CARD (ind.hom)	FBD_c-CARD (ind.het)	FBD_c-CARD (sw.hom)	FBD_c-CARD (sw.het)
Mean log marginal likelihood	-71.307	-70.792	-65.333	-63.823	-65.713	-64.366
Mean log Bayes Factor	-0.127	0.903	11.822	14.840	11.062	13.755
Amniota	0.74:0.055:0.118:0.087	0.741:0.053:0.103:0.102	0.999:0:0:0	1:0:0:0	0.998:0.002:0:0	0.999:0.001:0:0
Mammalia	0.771:0.145:0.047:0.036	0.747:0.155:0.051:0.047	0.999:0.001:0:0	1:0:0:0	0.998:0.002:0:0	0.999:0.001:0:0
Reptilia/ Diapsida s.l.	0.741:0.02:0.139:0.1	0.753:0.02:0.115:0.111	0.999:0:0:0	1:0:0:0	0.997:0.003:0:0	0.999:0.001:0:0
Diapsida s.s.	0.079:0.044:0.56:0.317	0.131:0.048:0.445:0.376	0.998:0.001:0.001:0.001	0.999:0.001:0:0	0.991:0.006:0.001:0.001	0.997:0.002:0:0
Lepidosauria	0.19:0.053:0.648:0.11	0.214:0.056:0.633:0.098	0.998:0:0.002:0	0.999:0:0.001:0	0.992:0.001:0.006:0.001	0.997:0.001:0.002:0
Archelosauria	0.158:0.07:0.313:0.459	0.226:0.066:0.212:0.497	0.998:0.001:0:0.001	0.999:0:0:0	0.992:0.006:0:0.002	0.998:0.002:0:0
Archosauromorpha	0.534:0.082:0.22:0.164	0.526:0.079:0.177:0.218	0.999:0.001:0:0	1:0:0:0	0.995:0.004:0:0.001	0.998:0.001:0:0
Archosauria	0.007:0.213:0.38:0.4	0.015:0.2:0.278:0.507	0.029:0.807:0.13:0.034	0.053:0.819:0.069:0.059	0.027:0.679:0.265:0.03	0.145:0.551:0.263:0.04
Dinosauria	0.007:0.277:0.384:0.332	0.01:0.273:0.288:0.429	0.02:0.826:0.126:0.028	0.031:0.856:0.068:0.045	0.024:0.687:0.264:0.025	0.098:0.615:0.258:0.03
Saurischia	0.002:0.261:0.42:0.316	0.004:0.257:0.332:0.408	0.013:0.814:0.144:0.03	0.022:0.862:0.073:0.042	0.008:0.655:0.311:0.027	0.042:0.649:0.28:0.029
Theropoda	0.008:0.003:0.007:0.981	0.008:0.004:0.007:0.981	0.027:0.027:0.023:0.923	0.039:0.033:0.029:0.9	0.034:0.032:0.031:0.903	0.123:0.07:0.061:0.746

Table S35c. Continuation of Table S35b.

Character	Reproduction mode + egg shell mineralisation			
Model	FBD_c-ER (ind.hom)	FBD_c-ER (ind.het)	FBD_c-ER (sw.hom)	FBD_c-ER (sw.het)
Mean log marginal likelihood	-70.660	-70.118	-71.243	-70.657
Mean log Bayes Factor	1.168	2.252	0	1.174
Amniota	0.827:0.039:0.065:0.069	0.831:0.037:0.057:0.075	0.738:0.066:0.116:0.08	0.756:0.056:0.099:0.089
Mammalia	0.77:0.125:0.052:0.052	0.747:0.136:0.058:0.058	0.767:0.126:0.054:0.054	0.746:0.135:0.06:0.06
Reptilia/Diapsida s.l.	0.743:0.035:0.108:0.114	0.788:0.027:0.078:0.107	0.729:0.033:0.138:0.1	0.76:0.028:0.107:0.105
Diapsida s.s.	0.058:0.114:0.397:0.43	0.127:0.093:0.322:0.457	0.06:0.097:0.499:0.344	0.118:0.083:0.413:0.385
Lepidosauria	0.102:0.072:0.722:0.104	0.139:0.08:0.675:0.106	0.184:0.065:0.659:0.092	0.214:0.068:0.63:0.088
Archelosauria	0.222:0.153:0.098:0.528	0.284:0.111:0.081:0.524	0.141:0.163:0.162:0.534	0.217:0.123:0.134:0.526
Archosauromorpha	0.604:0.161:0.093:0.142	0.598:0.13:0.083:0.189	0.495:0.183:0.164:0.158	0.503:0.15:0.141:0.206
Archosauria	0.007:0.488:0.087:0.419	0.015:0.37:0.097:0.518	0.007:0.497:0.092:0.405	0.015:0.386:0.102:0.497
Dinosauria	0.005:0.551:0.112:0.332	0.009:0.452:0.117:0.422	0.006:0.558:0.118:0.318	0.01:0.465:0.123:0.402
Saurischia	0.003:0.526:0.198:0.273	0.005:0.438:0.197:0.361	0.002:0.534:0.201:0.263	0.004:0.446:0.201:0.349
Theropoda	0.006:0.006:0.006:0.982	0.005:0.005:0.005:0.984	0.007:0.006:0.006:0.98	0.006:0.005:0.005:0.984

Table S35d. Continuation of Table S35c.

Character	EER			
Model	FBD_c-EER ER (hom)	FBD_c-EER ER (het)	FBD_c-EER ARD (hom)	FBD_c-EER ARD (het)
Mean log marginal likelihood	-31.603	-31.550	-28.139	-28.021
Mean log Bayes Factor	0	0.106	6.926	7.162
Amniota	0.036:0.964	0.05:0.95	0.002:0.998	0.003:0.997
Mammalia	0.243:0.757	0.248:0.752	0.015:0.985	0.016:0.984

Reptilia/Diapsida s.l.	0.022:0.978	0.035:0.965	0.001:0.999	0.002:0.998
Diapsida s.s.	0.494:0.506	0.459:0.541	0.032:0.968	0.029:0.971
Lepidosauria	0.204:0.796	0.233:0.767	0.013:0.987	0.014:0.986
Archelosauria	0.754:0.246	0.668:0.332	0.048:0.952	0.043:0.957
Archosauromorpha	0.33:0.67	0.321:0.679	0.02:0.98	0.021:0.979
Archosauria	0.991:0.009	0.987:0.013	0.987:0.013	0.951:0.049
Dinosauria	0.997:0.003	0.997:0.003	0.995:0.005	0.976:0.024
Saurischia	0.999:0.001	0.998:0.002	0.998:0.002	0.985:0.015
Theropoda	0.993:0.007	0.995:0.005	0.988:0.012	0.961:0.039

Table S35e. Continuation of Table S35d. Nodes of Lepidosauria and Squamata fixed to a non-viviparous state.

Character	Reproduction mode + egg shell mineralisation					
Model	FBD_c-CER (ind.hom)	FBD_c-CER (ind.het)	FBD_c-CER (sw.hom)	FBD_c-CER (sw.het)	FBD_c-CSYM (ind.hom)	FBD_c-CSYM (ind.het)
Mean log marginal likelihood	-70.275	-69.762	-70.648	-70.130	-71.549	-71.054
Mean log Bayes Factor	2.565	3.593	1.820	2.855	0.017	1.008
Amniota	0.776:0.049:0.084:0.09	0.777:0.046:0.077:0.1	0.674:0.085:0.137:0.105	0.69:0.074:0.119:0.117	0.776:0.04:0.085:0.1	0.777:0.041:0.073:0.11
Mammalia	0.778:0.124:0.049:0.049	0.754:0.139:0.054:0.054	0.776:0.125:0.05:0.05	0.752:0.138:0.055:0.055	0.779:0.145:0.037:0.039	0.759:0.154:0.04:0.047
Reptilia/Diapsida s.l.	0.658:0.045:0.143:0.154	0.698:0.035:0.116:0.151	0.623:0.046:0.189:0.142	0.664:0.038:0.151:0.146	0.658:0.025:0.154:0.163	0.697:0.023:0.12:0.16
Diapsida s.s.	0.02:0.106:0.413:0.46	0.048:0.088:0.364:0.5	0.013:0.094:0.519:0.374	0.03:0.083:0.449:0.438	0.021:0.046:0.453:0.48	0.048:0.048:0.379:0.525
Lepidosauria	0:0.073:0.824:0.103	0:0.079:0.818:0.104	0:0.074:0.824:0.102	0:0.08:0.818:0.102	0:0.051:0.839:0.11	0:0.055:0.839:0.106
Archelosauria	0.209:0.139:0.089:0.563	0.24:0.104:0.074:0.581	0.129:0.15:0.146:0.576	0.179:0.115:0.121:0.585	0.206:0.06:0.177:0.557	0.234:0.056:0.119:0.59
Archosau	0.605:0.153	0.57:0.133:	0.49:0.179:	0.482:0.153	0.599:0.069	0.561:0.07:

romorpha	:0.089:0.153	0.083:0.214	0.159:0.172	:0.136:0.23	:0.14:0.192	0.113:0.255
Archosauria	0.005:0.466:0.077:0.452	0.011:0.358:0.086:0.546	0.005:0.481:0.08:0.434	0.011:0.375:0.09:0.524	0.005:0.201:0.249:0.544	0.011:0.183:0.178:0.629
Dinosauria	0.005:0.532:0.106:0.357	0.007:0.441:0.11:0.442	0.005:0.546:0.11:0.339	0.008:0.46:0.116:0.416	0.005:0.268:0.258:0.469	0.008:0.256:0.189:0.547
Saurischia	0.002:0.513:0.197:0.289	0.004:0.429:0.194:0.373	0.002:0.522:0.198:0.277	0.003:0.444:0.198:0.355	0.003:0.251:0.299:0.448	0.005:0.24:0.234:0.522
Theropoda	0.005:0.005:0.005:0.985	0.005:0.005:0.005:0.985	0.006:0.005:0.005:0.984	0.006:0.005:0.005:0.984	0.005:0.004:0.005:0.987	0.005:0.004:0.005:0.986

Table S35f. Continuation of Table S35e.

Character	Reproduction mode + egg shell mineralisation					
Model	FBD_c-CSYM (sw.hom)	FBD_c-CSYM (sw.het)	FBD_c-CARD (ind.hom)	FBD_c-CARD (ind.het)	FBD_c-CARD (sw.hom)	FBD_c-CARD (sw.het)
Mean log marginal likelihood	-71.708	-71.178	-71.521	-71.180	-70.836	-70.595
Mean log Bayes Factor	-0.300	0.760	0.075	0.757	1.443	1.926
Amniota	0.655:0.075:0.146:0.124	0.682:0.067:0.113:0.137	0.684:0.219:0.052:0.046	0.725:0.159:0.048:0.069	0.762:0.185:0.032:0.02	0.798:0.137:0.03:0.035
Mammalia	0.775:0.144:0.042:0.038	0.757:0.151:0.044:0.047	0.69:0.259:0.028:0.023	0.696:0.24:0.03:0.034	0.789:0.186:0.015:0.01	0.792:0.176:0.016:0.016
Reptilia/ Diapsida s.l.	0.628:0.028:0.197:0.147	0.665:0.028:0.151:0.156	0.6:0.255:0.079:0.066	0.665:0.175:0.07:0.09	0.591:0.326:0.053:0.03	0.687:0.223:0.045:0.045
Diapsida s.s.	0.015:0.047:0.556:0.382	0.035:0.052:0.458:0.454	0.037:0.614:0.195:0.155	0.106:0.468:0.183:0.243	0.026:0.775:0.134:0.066	0.111:0.65:0.124:0.115
Lepidosauria	0:0.061:0.817:0.122	0:0.065:0.822:0.114	0:0.096:0.725:0.179	0:0.128:0.663:0.21	0:0.107:0.718:0.174	0:0.148:0.651:0.201
Archelos	0.146:0.07:	0.187:0.065	0.226:0.54:	0.288:0.406	0.121:0.743	0.239:0.59:

auria	0.26:0.524	:0.179:0.569	0.076:0.159	:0.059:0.246	:0.06:0.076	0.049:0.122
Archosauromorpha	0.518:0.085:0.195:0.202	0.496:0.082:0.158:0.265	0.532:0.357:0.054:0.057	0.54:0.301:0.049:0.11	0.42:0.511:0.039:0.03	0.475:0.429:0.038:0.058
Archosauria	0.006:0.221:0.297:0.476	0.011:0.197:0.216:0.576	0.008:0.789:0.07:0.133	0.016:0.69:0.059:0.236	0.009:0.903:0.033:0.055	0.03:0.824:0.036:0.109
Dinosauria	0.006:0.289:0.307:0.398	0.008:0.268:0.227:0.497	0.007:0.801:0.071:0.121	0.01:0.715:0.062:0.213	0.013:0.903:0.036:0.048	0.024:0.841:0.038:0.097
Saurischia	0.002:0.273:0.347:0.378	0.003:0.252:0.272:0.473	0.004:0.777:0.104:0.115	0.006:0.698:0.093:0.203	0.003:0.894:0.057:0.046	0.006:0.841:0.058:0.095
Theropoda	0.007:0.004:0.007:0.983	0.006:0.005:0.007:0.982	0.007:0.007:0.006:0.98	0.01:0.008:0.006:0.975	0.009:0.009:0.007:0.975	0.017:0.012:0.007:0.963

Table S35g. Continuation of Table S35f.

Character	Reproduction mode + egg shell mineralisation			
Model	FBD_c-ER (ind.hom)	FBD_c-ER (ind.het)	FBD_c-ER (sw.hom)	FBD_c-ER (sw.het)
Mean log marginal likelihood	-71.002	-70.508	-71.558	-71.039
Mean log Bayes Factor	1.112	2.100	0	1.039
Amniota	0.778:0.049:0.084:0.089	0.777:0.047:0.077:0.099	0.674:0.085:0.136:0.104	0.69:0.073:0.119:0.118
Mammalia	0.776:0.125:0.049:0.049	0.752:0.137:0.055:0.055	0.773:0.126:0.051:0.051	0.751:0.136:0.056:0.056
Reptilia/Diapsida s.l.	0.661:0.045:0.142:0.151	0.7:0.037:0.115:0.149	0.628:0.047:0.187:0.138	0.666:0.038:0.148:0.147
Diapsida s.s.	0.02:0.107:0.415:0.458	0.052:0.09:0.363:0.495	0.014:0.097:0.516:0.372	0.035:0.084:0.443:0.438
Lepidosauria	0:0.074:0.82:0.105	0:0.083:0.807:0.11	0:0.078:0.814:0.109	0:0.083:0.809:0.108
Archelosauria	0.21:0.14:0.09:0.56	0.25:0.105:0.078:0.567	0.133:0.154:0.151:0.562	0.181:0.116:0.123:0.58
Archosauromorpha	0.604:0.154:0.09:0.152	0.575:0.131:0.084:0.211	0.491:0.18:0.161:0.168	0.478:0.154:0.14:0.228
Archosauria	0.005:0.469:0.078	0.012:0.355:0.089	0.006:0.485:0.083	0.013:0.372:0.092

	:0.447	:0.544	:0.426	:0.524
Dinosauria	0.005:0.536:0.107 :0.352	0.008:0.439:0.112 :0.441	0.006:0.55:0.112: 0.332	0.008:0.454:0.116 :0.422
Saurischia	0.002:0.514:0.197 :0.286	0.004:0.429:0.195 :0.371	0.002:0.526:0.199 :0.273	0.003:0.44:0.2:0.3 56
Theropoda	0.005:0.005:0.005 :0.985	0.005:0.005:0.005 :0.986	0.006:0.005:0.005 :0.983	0.006:0.005:0.005 :0.984

Table S36a. Same as Table S28 but excluding extinct marine reptiles from the analyses (equal dating method).

Character	Reproduction mode + egg shell mineralisation					
Model	equal-CER (ind.hom)	equal-CER (ind.het)	equal-CER (sw.hom)	equal-CER (sw.het)	equal-CSYM (ind.hom)	equal-CSYM (ind.het)
Mean log marginal likelihood	-68.52	-67.817	-69.65	-68.797	-69.824	-69.102
Mean log Bayes Factor	2.286	3.693	0.026	1.733	-0.321	1.123
Amniota	0.607:0.378:0.006:0.010	0.649:0.330:0.008:0.013	0.456:0.528:0.007:0.009	0.546:0.432:0.009:0.013	0.623:0.362:0.007:0.008	0.665:0.316:0.008:0.012
Mammalia	0.721:0.141:0.069:0.069	0.695:0.153:0.076:0.076	0.720:0.145:0.068:0.068	0.689:0.155:0.078:0.078	0.716:0.161:0.062:0.061	0.691:0.171:0.066:0.072
Reptilia	0.541:0.434:0.009:0.016	0.609:0.363:0.010:0.018	0.525:0.451:0.010:0.014	0.600:0.372:0.010:0.017	0.598:0.378:0.010:0.014	0.655:0.318:0.010:0.017
Diapsida s.l./s.s.	0.076:0.065:0.290:0.569	0.161:0.057:0.242:0.540	0.065:0.056:0.370:0.510	0.159:0.051:0.287:0.503	0.095:0.063:0.346:0.496	0.185:0.055:0.265:0.496
Lepidosauria	0.070:0.043:0.813:0.074	0.114:0.057:0.748:0.080	0.135:0.032:0.783:0.051	0.200:0.045:0.696:0.060	0.082:0.040:0.797:0.082	0.124:0.049:0.747:0.079
Archelosauria	0.369:0.071:0.032:0.528	0.380:0.060:0.028:0.532	0.258:0.077:0.046:0.619	0.338:0.064:0.041:0.557	0.385:0.055:0.095:0.464	0.401:0.048:0.062:0.489
Archosauromorpha	0.899:0.046:0.017:0.038	0.816:0.067:0.025:0.091	0.845:0.067:0.030:0.058	0.773:0.076:0.038:0.113	0.897:0.030:0.031:0.041	0.821:0.043:0.040:0.096
Archosauria	0.003:0.492:0.051:0.454	0.006:0.397:0.060:0.537	0.002:0.491:0.047:0.459	0.008:0.390:0.061:0.541	0.003:0.303:0.218:0.476	0.008:0.256:0.162:0.575
Dinosauria	0.002:0.688:0.065:0.245	0.003:0.588:0.075:0.335	0.001:0.693:0.061:0.244	0.003:0.578:0.076:0.343	0.002:0.442:0.223:0.333	0.004:0.393:0.168:0.435
Saurischia	0.001:0.645:0.136:0.21	0.002:0.556:0.144:0.29	0.001:0.657:0.130:0.21	0.002:0.555:0.142:0.30	0.001:0.419:0.257:0.32	0.002:0.377:0.205:0.41

	8	9	3	1	2	5
Theropoda	0.015:0.017:0.017:0.951	0.014:0.015:0.015:0.957	0.019:0.017:0.017:0.947	0.019:0.015:0.015:0.950	0.018:0.014:0.017:0.950	0.017:0.014:0.016:0.953

Table S36b. Continuation of Table S36a.

Character	Reproduction mode + egg shell mineralisation					
Model	equal-CSYM (sw.hom)	equal-CSYM (sw.het)	equal-CARD (ind.hom)	equal-CARD (ind.het)	equal-CARD (sw.hom)	equal-CARD (sw.het)
Mean log marginal likelihood	-70.117	-69.409	-59.487	-58.442	-59.93	-57.965
Mean log Bayes Factor	-0.908	0.509	20.352	22.442	19.467	23.397
Amniota	0.508:0.475:0.009:0.007	0.547:0.431:0.010:0.012	1.000:0.000:0.000:0.000	1.000:0.000:0.000:0.000	1.000:0.000:0.000:0.000	1.000:0.000:0.000:0.000
Mammalia	0.714:0.176:0.062:0.049	0.686:0.184:0.065:0.065	1.000:0.000:0.000:0.000	1.000:0.000:0.000:0.000	1.000:0.000:0.000:0.000	1.000:0.000:0.000:0.000
Reptilia	0.622:0.353:0.013:0.012	0.642:0.330:0.012:0.016	1.000:0.000:0.000:0.000	1.000:0.000:0.000:0.000	1.000:0.000:0.000:0.000	1.000:0.000:0.000:0.000
Diapsida s.l./s.s.	0.107:0.051:0.481:0.361	0.166:0.053:0.350:0.431	1.000:0.000:0.000:0.000	1.000:0.000:0.000:0.000	1.000:0.000:0.000:0.000	1.000:0.000:0.000:0.000
Lepidosauria	0.160:0.030:0.743:0.066	0.194:0.035:0.712:0.059	0.999:0.000:0.000:0.000	1.000:0.000:0.000:0.000	1.000:0.000:0.000:0.000	0.999:0.000:0.000:0.000
Archelosauria	0.284:0.047:0.218:0.451	0.336:0.049:0.123:0.492	1.000:0.000:0.000:0.000	1.000:0.000:0.000:0.000	1.000:0.000:0.000:0.000	1.000:0.000:0.000:0.000
Archosauromorpha	0.865:0.027:0.053:0.055	0.777:0.043:0.062:0.118	1.000:0.000:0.000:0.000	1.000:0.000:0.000:0.000	1.000:0.000:0.000:0.000	1.000:0.000:0.000:0.000
Archosauria	0.003:0.188:0.401:0.408	0.008:0.193:0.268:0.531	0.020:0.900:0.061:0.019	0.051:0.853:0.076:0.021	0.021:0.769:0.206:0.004	0.163:0.543:0.290:0.004
Dinosauri	0.002:0.303	0.004:0.312	0.014:0.918	0.036:0.882	0.021:0.791	0.147:0.595

a	:0.404:0.292	:0.274:0.410	:0.056:0.012	:0.069:0.013	:0.185:0.003	:0.255:0.003
Saurischia	0.001:0.295:0.416:0.288	0.002:0.302:0.298:0.398	0.009:0.922:0.059:0.009	0.026:0.903:0.060:0.011	0.006:0.788:0.204:0.003	0.071:0.699:0.227:0.002
Theropoda	0.023:0.010:0.018:0.949	0.022:0.011:0.016:0.951	0.088:0.083:0.073:0.756	0.117:0.083:0.071:0.729	0.091:0.076:0.065:0.768	0.237:0.121:0.069:0.574

Table S36c. Continuation of Table S36b.

Character	Reproduction mode + egg shell mineralisation			
Model	equal-ER (ind.hom)	equal-ER (ind.het)	equal-ER (sw.hom)	equal-ER (sw.het)
Mean log marginal likelihood	-69.343	-68.586	-69.663	-68.912
Mean log Bayes Factor	0.64	2.154	0	1.502
Amniota	0.612:0.371:0.006:0.010	0.660:0.318:0.008:0.014	0.487:0.494:0.009:0.010	0.562:0.410:0.012:0.016
Mammalia	0.718:0.140:0.071:0.071	0.688:0.151:0.081:0.081	0.715:0.142:0.071:0.071	0.682:0.152:0.083:0.083
Reptilia	0.545:0.430:0.009:0.016	0.619:0.353:0.010:0.018	0.546:0.428:0.012:0.015	0.608:0.360:0.013:0.019
Diapsida s.l./s.s.	0.085:0.070:0.289:0.557	0.183:0.060:0.231:0.526	0.088:0.061:0.369:0.482	0.182:0.055:0.284:0.479
Lepidosauria	0.076:0.046:0.797:0.081	0.133:0.065:0.713:0.089	0.163:0.038:0.737:0.062	0.222:0.050:0.660:0.068
Archelosauria	0.377:0.076:0.034:0.513	0.398:0.063:0.030:0.509	0.284:0.085:0.054:0.577	0.354:0.068:0.045:0.533
Archosauromorpha	0.898:0.048:0.017:0.037	0.821:0.067:0.026:0.086	0.845:0.068:0.033:0.054	0.771:0.081:0.041:0.107
Archosauria	0.003:0.507:0.054:0.436	0.008:0.404:0.064:0.524	0.003:0.511:0.058:0.428	0.009:0.413:0.067:0.511
Dinosauria	0.002:0.693:0.068:0.237	0.004:0.581:0.079:0.336	0.002:0.694:0.072:0.232	0.004:0.590:0.083:0.324
Saurischia	0.001:0.648:0.138:0.213	0.002:0.553:0.146:0.299	0.001:0.651:0.141:0.208	0.002:0.561:0.149:0.289
Theropoda	0.017:0.018:0.018:0.947	0.015:0.016:0.016:0.954	0.023:0.019:0.019:0.938	0.021:0.017:0.017:0.946

Table S36d. Continuation of Table S36c.

Character	EER			
Model	equal-EER ER (hom)	equal-EER ER (het)	equal-EER ARD (hom)	equal-EER ARD (het)
Mean log marginal likelihood	-30.473	-30.067	-25.928	-25.456
Mean log Bayes Factor	0	0.813	9.09	10.034
Amniota	0.003:0.997	0.014:0.986	0.000:1.000	0.000:1.000
Mammalia	0.283:0.717	0.315:0.685	0.006:0.994	0.007:0.993
Reptilia	0.001:0.999	0.008:0.992	0.000:1.000	0.000:1.000
Diapsida s.l./s.s.	0.184:0.816	0.227:0.773	0.004:0.996	0.004:0.996
Lepidosauria	0.100:0.900	0.174:0.826	0.002:0.998	0.004:0.996
Archelosauria	0.613:0.387	0.487:0.513	0.012:0.988	0.012:0.988
Archosauromorpha	0.096:0.904	0.130:0.870	0.002:0.998	0.003:0.997
Archosauria	0.996:0.004	0.994:0.006	0.995:0.005	0.962:0.038
Dinosauria	0.998:0.002	0.998:0.002	0.997:0.003	0.972:0.028
Saurischia	0.999:0.001	0.999:0.001	0.998:0.002	0.979:0.021
Theropoda	0.981:0.019	0.987:0.013	0.971:0.029	0.909:0.091

Table S36e. Continuation of Table S36d. Nodes of Lepidosauria and Squamata fixed to a non-viviparous state.

Character	Reproduction mode + egg shell mineralisation					
Model	equal-CER (ind.hom)	equal-CER (ind.het)	equal-CER (sw.hom)	equal-CER (sw.het)	equal-CSYM (ind.hom)	equal-CSYM (ind.het)
Mean log marginal likelihood	-68.706	-68.077	-69.77	-69.013	-70.037	-69.37
Mean log Bayes Factor	2.232	3.49	0.106	1.618	-0.428	0.906
Amniota	0.533:0.449:0.007:0.012	0.572:0.404:0.009:0.015	0.393:0.589:0.008:0.010	0.456:0.519:0.010:0.015	0.541:0.440:0.008:0.011	0.576:0.399:0.010:0.015
Mammalia	0.723:0.141:0.068:0.068	0.697:0.156:0.073:0.073	0.723:0.145:0.066:0.066	0.692:0.159:0.075:0.075	0.720:0.160:0.059:0.060	0.700:0.173:0.061:0.067
Reptilia	0.465:0.506:0.011:0.019	0.527:0.440:0.011:0.022	0.455:0.518:0.012:0.016	0.510:0.458:0.013:0.020	0.513:0.456:0.012:0.018	0.564:0.400:0.014:0.022
Diapsida	0.025:0.068	0.062:0.061	0.015:0.056	0.044:0.057	0.027:0.068	0.062:0.062

s.l./s.s.	:0.307:0.600	:0.269:0.608	:0.386:0.543	:0.326:0.573	:0.365:0.540	:0.308:0.568
Lepidosauria	0.000:0.045:0.878:0.077	0.000:0.059:0.856:0.085	0.000:0.036:0.905:0.058	0.000:0.057:0.868:0.076	0.000:0.041:0.879:0.080	0.000:0.051:0.871:0.078
Archelosauria	0.361:0.069:0.030:0.541	0.343:0.058:0.027:0.573	0.246:0.072:0.042:0.640	0.277:0.062:0.038:0.622	0.371:0.054:0.087:0.489	0.343:0.047:0.065:0.544
Archosauromorpha	0.900:0.046:0.016:0.038	0.808:0.068:0.025:0.098	0.844:0.065:0.030:0.060	0.753:0.083:0.039:0.124	0.899:0.029:0.028:0.043	0.811:0.044:0.043:0.103
Archosauria	0.002:0.487:0.048:0.462	0.004:0.380:0.052:0.564	0.002:0.479:0.044:0.475	0.005:0.381:0.052:0.562	0.003:0.304:0.192:0.502	0.006:0.239:0.159:0.597
Dinosauria	0.001:0.689:0.063:0.247	0.002:0.579:0.070:0.349	0.001:0.691:0.058:0.250	0.002:0.577:0.069:0.352	0.002:0.450:0.200:0.348	0.003:0.379:0.170:0.448
Saurischia	0.001:0.644:0.135:0.221	0.002:0.548:0.142:0.308	0.001:0.654:0.128:0.217	0.001:0.553:0.137:0.308	0.001:0.425:0.238:0.336	0.002:0.360:0.210:0.428
Theropoda	0.014:0.016:0.016:0.955	0.012:0.013:0.013:0.961	0.017:0.015:0.015:0.953	0.016:0.014:0.014:0.957	0.015:0.014:0.016:0.955	0.014:0.013:0.014:0.958

Table S36f. Continuation of Table S36e.

Character	Reproduction mode + egg shell mineralisation					
Model	equal-CSYM (sw.hom)	equal-CSYM (sw.het)	equal-CARD (ind.hom)	equal-CARD (ind.het)	equal-CARD (sw.hom)	equal-CARD (sw.het)
Mean log marginal likelihood	-70.346	-69.667	-68.018	-67.295	-67.364	-66.299
Mean log Bayes Factor	-1.047	0.312	3.608	5.055	4.918	7.047
Amniota	0.399:0.582:0.010:0.009	0.454:0.521:0.010:0.015	0.534:0.464:0.001:0.001	0.721:0.276:0.001:0.002	0.770:0.229:0.000:0.000	0.898:0.101:0.000:0.000
Mammalia	0.719:0.174:0.055:0.052	0.692:0.184:0.059:0.065	0.722:0.224:0.027:0.026	0.805:0.155:0.020:0.021	0.875:0.090:0.018:0.017	0.921:0.059:0.010:0.009

Reptilia	0.519:0.449:0.016:0.016	0.555:0.411:0.014:0.020	0.439:0.559:0.001:0.001	0.629:0.367:0.001:0.002	0.592:0.407:0.000:0.000	0.784:0.215:0.001:0.001
Diapsida s.l./s.s.	0.017:0.059:0.487:0.437	0.042:0.063:0.376:0.518	0.384:0.554:0.034:0.027	0.582:0.328:0.039:0.051	0.506:0.454:0.027:0.013	0.742:0.225:0.019:0.014
Lepidosauria	0.000:0.034:0.899:0.067	0.000:0.044:0.885:0.070	0.000:0.163:0.677:0.160	0.000:0.200:0.633:0.167	0.000:0.194:0.718:0.088	0.000:0.216:0.680:0.104
Archelosauria	0.265:0.051:0.177:0.507	0.287:0.050:0.104:0.558	0.645:0.327:0.008:0.021	0.753:0.199:0.007:0.040	0.720:0.262:0.007:0.012	0.866:0.117:0.005:0.012
Archosauromorpha	0.862:0.029:0.043:0.066	0.761:0.045:0.057:0.137	0.867:0.126:0.003:0.004	0.894:0.093:0.004:0.009	0.920:0.076:0.002:0.002	0.951:0.045:0.002:0.003
Archosauria	0.002:0.204:0.313:0.481	0.005:0.200:0.205:0.590	0.017:0.943:0.015:0.025	0.040:0.878:0.024:0.058	0.036:0.943:0.008:0.013	0.130:0.832:0.014:0.023
Dinosauria	0.001:0.323:0.321:0.355	0.003:0.326:0.214:0.458	0.012:0.955:0.015:0.018	0.028:0.907:0.023:0.043	0.031:0.951:0.007:0.010	0.093:0.876:0.014:0.017
Saurischia	0.001:0.311:0.340:0.348	0.001:0.315:0.240:0.444	0.007:0.955:0.020:0.017	0.020:0.910:0.029:0.041	0.007:0.973:0.011:0.009	0.026:0.942:0.017:0.015
Theropoda	0.019:0.012:0.015:0.954	0.018:0.012:0.014:0.956	0.067:0.054:0.022:0.857	0.098:0.062:0.023:0.817	0.100:0.070:0.019:0.811	0.152:0.084:0.020:0.744

Table S36g. Continuation of Table S36f.

Character	Reproduction mode + egg shell mineralisation			
Model	equal-ER (ind.hom)	equal-ER (ind.het)	equal-ER (sw.hom)	equal-ER (sw.het)
Mean log marginal likelihood	-69.544	-68.876	-69.823	-69.165
Mean log Bayes Factor	0.558	1.894	0	1.315
Amniota	0.534:0.447:0.007:0.011	0.576:0.397:0.010:0.017	0.404:0.575:0.009:0.012	0.457:0.514:0.012:0.017
Mammalia	0.721:0.141:0.069:0.069	0.696:0.153:0.076:0.076	0.719:0.143:0.069:0.069	0.689:0.157:0.077:0.077
Reptilia	0.466:0.506:0.010	0.528:0.436:0.012	0.455:0.514:0.014	0.507:0.455:0.015

	:0.018	:0.023	:0.018	:0.023
Diapsida s.l./s.s.	0.026:0.070:0.306 :0.598	0.064:0.059:0.278 :0.599	0.018:0.064:0.391 :0.528	0.046:0.060:0.334 :0.559
Lepidosauria	0.000:0.046:0.873 :0.080	0.000:0.062:0.849 :0.088	0.000:0.044:0.883 :0.073	0.000:0.061:0.855 :0.084
Archelosauria	0.364:0.070:0.031 :0.535	0.344:0.056:0.028 :0.572	0.263:0.081:0.050 :0.607	0.290:0.067:0.042 :0.601
Archosauromo rpha	0.899:0.047:0.016 :0.038	0.808:0.067:0.026 :0.099	0.843:0.067:0.032 :0.057	0.749:0.089:0.043 :0.119
Archosauria	0.002:0.493:0.049 :0.456	0.005:0.381:0.056 :0.558	0.002:0.499:0.051 :0.447	0.006:0.399:0.058 :0.537
Dinosauria	0.001:0.690:0.063 :0.246	0.003:0.570:0.075 :0.352	0.001:0.695:0.066 :0.238	0.003:0.589:0.076 :0.332
Saurischia	0.001:0.645:0.135 :0.219	0.002:0.540:0.146 :0.312	0.001:0.651:0.136 :0.213	0.002:0.556:0.145 :0.297
Theropoda	0.015:0.016:0.016 :0.953	0.014:0.014:0.014 :0.958	0.020:0.017:0.017 :0.946	0.018:0.015:0.015 :0.952

Table S37a. Same as Table S29 but excluding extinct marine reptiles from the analyses (mbl dating method).

Model	Reproduction mode + egg shell mineralisation					
Character	mbl-CER (ind.hom)	mbl-CER (ind.het)	mbl-CER (sw.hom)	mbl-CER (sw.het)	mbl-CSYM (ind.hom)	mbl-CSYM (ind.het)
Mean log marginal likelihood	-68.976	-67.547	-69.448	-67.766	-70.834	-69.308
Mean log Bayes Factor	1.736	4.594	0.792	4.156	-1.98	1.072
Amniota	0.841:0.148:0.006:0.006	0.812:0.160:0.014:0.014	0.814:0.173:0.006:0.007	0.784:0.182:0.016:0.017	0.831:0.155:0.008:0.006	0.804:0.164:0.017:0.015
Mammalia	0.981:0.008:0.006:0.006	0.965:0.013:0.011:0.011	0.979:0.009:0.006:0.006	0.957:0.016:0.013:0.013	0.979:0.009:0.006:0.006	0.959:0.015:0.013:0.012
Reptilia.	0.833:0.162:0.002:0.002	0.813:0.173:0.007:0.007	0.819:0.175:0.003:0.003	0.795:0.188:0.008:0.009	0.826:0.168:0.003:0.003	0.807:0.177:0.009:0.008
Diapsida s.l./s.s.	0.887:0.067:0.022:0.024	0.877:0.060:0.030:0.033	0.848:0.079:0.038:0.034	0.854:0.067:0.038:0.040	0.887:0.063:0.026:0.024	0.872:0.058:0.035:0.035
Lepidosauria	0.198:0.055:0.120:0.627	0.294:0.085:0.147:0.473	0.164:0.061:0.174:0.600	0.287:0.092:0.182:0.438	0.212:0.05:0.116:0.623	0.308:0.081:0.147:0.464
Archelosauria	0.924:0.048:0.014:0.014	0.910:0.047:0.021:0.022	0.903:0.056:0.023:0.018	0.893:0.053:0.027:0.027	0.924:0.046:0.017:0.013	0.907:0.045:0.024:0.023
Archosauromorpha	0.954:0.030:0.009:0.007	0.944:0.031:0.013:0.012	0.949:0.030:0.013:0.008	0.938:0.032:0.017:0.014	0.954:0.03:0.01:0.006	0.943:0.031:0.014:0.012
Archosauria	0.028:0.827:0.094:0.051	0.056:0.738:0.117:0.089	0.032:0.813:0.094:0.061	0.063:0.718:0.117:0.102	0.035:0.807:0.112:0.046	0.067:0.709:0.14:0.083
Dinosauria	0.017:0.865:0.078:0.040	0.030:0.802:0.096:0.072	0.020:0.858:0.076:0.045	0.031:0.795:0.093:0.082	0.022:0.852:0.092:0.035	0.039:0.78:0.115:0.066
Saurischia	0.011:0.875:0.080:0.034	0.018:0.819:0.096:0.067	0.010:0.866:0.083:0.042	0.016:0.809:0.097:0.079	0.014:0.862:0.094:0.03	0.026:0.797:0.116:0.061

Theropoda	0.001:0.001:0.001:0.996	0.001:0.001:0.001:0.997	0.002:0.002:0.002:0.994	0.002:0.001:0.001:0.996	0.002:0.001:0.002:0.994	0.002:0.001:0.002:0.995

Table S37b. Continuation of Table S37a.

Character	Reproduction mode + egg shell mineralisation					
Model	mbl-CSYM (sw.hom)	mbl-CSYM (sw.het)	mbl-CARD (ind.hom)	mbl-CARD (ind.het)	mbl-CARD (sw.hom)	mbl-CARD (sw.het)
Mean log marginal likelihood	-71.009	-69.252	-64.02	-62.774	-66.419	-64.287
Mean log Bayes Factor	-2.329	1.184	11.649	14.139	6.851	11.115
Amniota	0.809:0.175:0.009:0.007	0.779:0.185:0.019:0.017	0.999:0.001:0:0	0.999:0.001:0:0	0.976:0.023:0.001:0	0.99:0.01:0:0
Mammalia	0.976:0.01:0.007:0.006	0.955:0.017:0.014:0.013	1:0:0:0	1:0:0:0	0.998:0.001:0:0	0.999:0.001:0:0
Reptilia	0.816:0.176:0.005:0.003	0.791:0.19:0.01:0.009	0.999:0.001:0:0	0.999:0.001:0:0	0.965:0.034:0:0	0.985:0.015:0:0
Diapsida s.l./s.s.	0.849:0.074:0.043:0.034	0.848:0.067:0.044:0.041	1:0:0:0	1:0:0:0	0.982:0.015:0.002:0.001	0.992:0.006:0.001:0.001
Lepidosauria	0.179:0.057:0.167:0.597	0.296:0.091:0.179:0.435	0.997:0:0:0.002	0.999:0:0:0.001	0.874:0.012:0.024:0.09	0.951:0.007:0.012:0.03
Archelosauria	0.904:0.052:0.026:0.018	0.89:0.053:0.03:0.027	1:0:0:0	1:0:0:0	0.99:0.009:0.001:0	0.995:0.004:0.001:0
Archosauromorpha	0.95:0.029:0.013:0.008	0.935:0.033:0.018:0.014	1:0:0:0	1:0:0:0	0.995:0.004:0.001:0	0.997:0.002:0:0
Archosauria	0.04:0.784:0.122:0.054	0.071:0.688:0.14:0.1	0.031:0.857:0.102:0.01	0.072:0.777:0.125:0.026	0.105:0.814:0.071:0.009	0.192:0.698:0.09:0.019
Dinosauria	0.025:0.841:0.095:0.039	0.038:0.77:0.112:0.08	0.02:0.893:0.081:0.006	0.048:0.836:0.1:0.017	0.055:0.882:0.057:0.006	0.115:0.799:0.073:0.012

Saurischia	0.013:0.849:0.102:0.036	0.021:0.785:0.115:0.079	0.013:0.906:0.076:0.005	0.033:0.862:0.091:0.014	0.012:0.93:0.054:0.005	0.032:0.889:0.069:0.01
Theropoda	0.003:0.002:0.004:0.991	0.002:0.001:0.002:0.995	0.002:0.002:0.002:0.995	0.004:0.004:0.004:0.988	0.002:0.003:0.003:0.991	0.005:0.005:0.005:0.986

Table S37c. Continuation of Table S37b.

Character	Reproduction mode + egg shell mineralisation			
Model	mbl-ER (ind.hom)	mbl-ER (ind.het)	mbl-ER (sw.hom)	mbl-ER (sw.het)
Mean log marginal likelihood	-69.24	-67.542	-69.844	-67.854
Mean log Bayes Factor	1.209	4.604	0	3.979
Amniota	0.836:0.151:0.007:0.007	0.787:0.172:0.02:0.02	0.808:0.177:0.007:0.008	0.759:0.193:0.023:0.024
Mammalia	0.98:0.008:0.006:0.006	0.953:0.017:0.015:0.015	0.977:0.009:0.007:0.007	0.944:0.02:0.018:0.018
Reptilia	0.832:0.163:0.003:0.003	0.794:0.185:0.01:0.01	0.815:0.178:0.003:0.004	0.774:0.2:0.013:0.013
Diapsida s.l./s.s.	0.894:0.062:0.021:0.023	0.869:0.061:0.034:0.037	0.864:0.07:0.034:0.031	0.856:0.065:0.038:0.041
Lepidosauria	0.214:0.057:0.113:0.616	0.326:0.097:0.139:0.437	0.185:0.065:0.164:0.587	0.327:0.104:0.166:0.403
Archelosauria	0.926:0.046:0.014:0.014	0.901:0.048:0.024:0.027	0.908:0.052:0.022:0.018	0.893:0.051:0.028:0.029
Archosauromorpha	0.954:0.029:0.009:0.007	0.937:0.033:0.016:0.014	0.949:0.029:0.013:0.009	0.935:0.032:0.018:0.016
Archosauria	0.032:0.818:0.097:0.053	0.071:0.708:0.121:0.1	0.039:0.798:0.098:0.065	0.079:0.686:0.122:0.112
Dinosauria	0.02:0.858:0.08:0.041	0.036:0.788:0.097:0.08	0.024:0.848:0.079:0.049	0.04:0.771:0.098:0.091
Saurischia	0.012:0.871:0.081:0.035	0.023:0.808:0.095:0.074	0.012:0.86:0.084:0.044	0.022:0.79:0.099:0.088
Theropoda	0.002:0.002:0.002:0.995	0.001:0.001:0.001:0.996	0.003:0.002:0.002:0.992	0.002:0.001:0.001:0.996

Table S37d. Continuation of Table S37c.

Character	EER			
Model	mbl-EER ER (hom)	mbl-EER ER (het)	mbl-EER ARD (hom)	mbl-EER ARD (het)

Mean log marginal likelihood	-32.215	-30.683	-31.273	-30.178
Mean log Bayes Factor	0	3.065	1.885	4.075
Amniota	0.022:0.978	0.051:0.949	0.007:0.993	0.018:0.982
Mammalia	0.028:0.972	0.053:0.947	0.011:0.989	0.023:0.977
Reptilia	0.01:0.99	0.025:0.975	0.003:0.997	0.008:0.992
Diapsida s.l./s.s.	0.076:0.924	0.125:0.875	0.034:0.966	0.064:0.936
Lepidosauria	0.393:0.607	0.463:0.537	0.159:0.841	0.243:0.757
Archelosauria	0.086:0.914	0.114:0.886	0.045:0.955	0.067:0.933
Archosauromorpha	0.047:0.953	0.069:0.931	0.023:0.977	0.037:0.963
Archosauria	0.948:0.052	0.97:0.03	0.981:0.019	0.972:0.028
Dinosauria	0.966:0.034	0.984:0.016	0.988:0.012	0.982:0.018
Saurischia	0.977:0.023	0.989:0.011	0.992:0.008	0.988:0.012
Theropoda	0.996:0.004	0.999:0.001	0.999:0.001	0.998:0.002

Table S37e. Continuation of Table S37d. Nodes of Lepidosauria and Squamata fixed to a non-viviparous state.

Character	Reproduction mode + egg shell mineralisation					
Model	mbl-CER (ind.hom)	mbl-CER (ind.het)	mbl-CER (sw.hom)	mbl-CER (sw.het)	mbl-CSYM (ind.hom)	mbl-CSYM (ind.het)
Mean log marginal likelihood	-69.251	67.959	-69.613	-68.093	-71.128	-69.734
Mean log Bayes Factor	1.563	4.149	0.839	3.881	-2.19	0.598
Amniota	0.841:0.148:0.005:0.005	0.814:0.159:0.013:0.013	0.811:0.177:0.006:0.006	0.787:0.183:0.014:0.015	0.833:0.154:0.007:0.005	0.81:0.161:0.015:0.014
Mammalia	0.981:0.008:0.005:0.005	0.966:0.013:0.011:0.011	0.98:0.009:0.006:0.006	0.963:0.014:0.011:0.011	0.98:0.009:0.006:0.006	0.964:0.014:0.011:0.011
Reptilia	0.832:0.164:0.002:0.002	0.812:0.175:0.006:0.006	0.813:0.181:0.003:0.003	0.794:0.191:0.007:0.008	0.824:0.171:0.003:0.002	0.809:0.176:0.008:0.007
Diapsida s.l./s.s.	0.872:0.076:0.025:0.027	0.861:0.069:0.033:0.037	0.838:0.086:0.04:0.036	0.845:0.074:0.04:0.041	0.867:0.075:0.031:0.028	0.858:0.066:0.039:0.038
Lepidosauria	0:0.067:0.152:0.781	0:0.121:0.214:0.665	0:0.073:0.209:0.718	0:0.13:0.258:0.612	0:0.06:0.149:0.791	0:0.116:0.21:0.675
Archelos	0.923:0.049	0.907:0.049	0.901:0.057	0.893:0.055	0.922:0.048	0.906:0.047

auria	:0.014:0.014	:0.021:0.023	:0.023:0.019	:0.026:0.026	:0.017:0.013	:0.024:0.023
Archosauromorpha	0.954:0.03:0.009:0.007	0.943:0.032:0.013:0.012	0.949:0.03:0.013:0.008	0.938:0.033:0.016:0.013	0.954:0.03:0.01:0.006	0.944:0.031:0.014:0.011
Archosauria	0.025:0.834:0.093:0.049	0.053:0.746:0.114:0.088	0.03:0.819:0.092:0.059	0.056:0.733:0.113:0.097	0.031:0.811:0.114:0.043	0.063:0.71:0.139:0.088
Dinosauria	0.015:0.87:0.077:0.038	0.028:0.808:0.093:0.071	0.019:0.862:0.075:0.044	0.029:0.801:0.092:0.078	0.02:0.855:0.092:0.033	0.037:0.777:0.116:0.07
Saurischia	0.009:0.877:0.08:0.034	0.018:0.822:0.094:0.066	0.009:0.868:0.082:0.041	0.015:0.814:0.096:0.075	0.012:0.863:0.095:0.029	0.024:0.793:0.117:0.066
Theropoda	0.001:0.001:0.001:0.996	0.001:0.001:0.001:0.997	0.002:0.002:0.002:0.994	0.001:0.001:0.001:0.996	0.002:0.001:0.002:0.995	0.001:0.001:0.002:0.996

Table S37f. Continuation of Table S37e.

Character	Reproduction mode + egg shell mineralisation					
Model	mbl-CSYM (sw.hom)	mbl-CSYM (sw.het)	mbl-CARD (ind.hom)	mbl-CARD (ind.het)	mbl-CARD (sw.hom)	mbl-CARD (sw.het)
Mean log marginal likelihood	-71.191	-69.598	-69.454	-68.631	-68.269	-67.226
Mean log Bayes Factor	-2.315	0.87	1.158	2.804	3.527	5.615
Amniota	0.807:0.179:0.008:0.006	0.783:0.183:0.018:0.016	0.853:0.138:0.006:0.003	0.845:0.141:0.009:0.006	0.88:0.115:0.003:0.001	0.893:0.1:0.004:0.002
Mammalia	0.977:0.01:0.007:0.006	0.957:0.017:0.013:0.013	0.987:0.006:0.004:0.003	0.979:0.009:0.006:0.005	0.99:0.006:0.002:0.002	0.987:0.008:0.003:0.003
Reptilia	0.811:0.182:0.004:0.003	0.792:0.19:0.009:0.009	0.847:0.15:0.003:0.001	0.832:0.161:0.004:0.003	0.827:0.171:0.002:0.001	0.834:0.162:0.002:0.001
Diapsida s.l./s.s.	0.834:0.083:0.047:0.036	0.839:0.072:0.046:0.043	0.938:0.046:0.011:0.005	0.918:0.054:0.016:0.012	0.909:0.078:0.009:0.004	0.916:0.068:0.01:0.006
Lepidosa	0:0.066:0.2	0:0.129:0.2	0:0.111:0.1	0:0.147:0.1	0:0.108:0.1	0:0.152:0.2

uria	07:0.727	48:0.623	33:0.757	83:0.67	84:0.708	25:0.623
Archelosauria	0.9:0.055:0.027:0.018	0.891:0.052:0.03:0.027	0.959:0.03:0.008:0.003	0.944:0.037:0.011:0.008	0.949:0.043:0.006:0.002	0.946:0.043:0.006:0.004
Archosauromorpha	0.949:0.03:0.013:0.008	0.938:0.032:0.017:0.013	0.975:0.018:0.005:0.002	0.965:0.024:0.007:0.004	0.975:0.021:0.003:0.001	0.972:0.022:0.004:0.002
Archosauria	0.035:0.793:0.12:0.053	0.063:0.702:0.14:0.095	0.061:0.831:0.092:0.015	0.09:0.776:0.101:0.033	0.088:0.848:0.054:0.01	0.133:0.792:0.055:0.02
Dinosauria	0.022:0.847:0.093:0.038	0.034:0.781:0.111:0.074	0.04:0.876:0.074:0.01	0.058:0.837:0.082:0.023	0.048:0.902:0.043:0.007	0.075:0.866:0.044:0.015
Saurischia	0.011:0.852:0.101:0.036	0.018:0.794:0.117:0.072	0.027:0.896:0.069:0.008	0.04:0.863:0.077:0.02	0.019:0.933:0.042:0.006	0.032:0.911:0.044:0.014
Theropoda	0.003:0.002:0.003:0.993	0.002:0.001:0.002:0.995	0.004:0.004:0.003:0.988	0.005:0.004:0.003:0.987	0.004:0.004:0.003:0.989	0.005:0.004:0.003:0.988

Table S37g. Continuation of Table S37f.

Character	Reproduction mode + egg shell mineralisation			
Model	mbl-ER (ind.hom)	mbl-ER (ind.het)	mbl-ER (sw.hom)	mbl-ER (sw.het)
Mean log marginal likelihood	-69.537	-67.994	-70.033	-68.255
Mean log Bayes Factor	0.993	4.078	0	3.556
Amniota	0.834:0.153:0.006:0.006	0.789:0.172:0.019:0.019	0.804:0.182:0.007:0.007	0.762:0.196:0.021:0.022
Mammalia	0.98:0.008:0.006:0.006	0.955:0.016:0.015:0.015	0.978:0.009:0.007:0.007	0.948:0.019:0.017:0.017
Reptilia	0.828:0.166:0.003:0.003	0.795:0.186:0.01:0.01	0.807:0.186:0.003:0.004	0.774:0.204:0.011:0.012
Diapsida s.l./s.s.	0.881:0.07:0.023:0.026	0.861:0.065:0.035:0.039	0.853:0.078:0.036:0.033	0.851:0.069:0.04:0.04
Lepidosauria	0:0.074:0.144:0.782	0:0.147:0.205:0.649	0:0.081:0.2:0.719	0:0.155:0.247:0.597
Archelosauria	0.925:0.047:0.014:0.014	0.902:0.048:0.024:0.026	0.906:0.054:0.022:0.018	0.893:0.052:0.028:0.027
Archosauromorpha	0.954:0.03:0.009:0.007	0.939:0.032:0.015:0.014	0.949:0.03:0.013:0.009	0.935:0.032:0.018:0.015

Archosauria	0.031:0.822:0.095:0.052	0.066:0.718:0.12:0.097	0.037:0.805:0.095:0.063	0.074:0.698:0.121:0.107
Dinosauria	0.019:0.861:0.079:0.041	0.035:0.788:0.097:0.08	0.023:0.852:0.078:0.047	0.038:0.777:0.098:0.087
Saurischia	0.012:0.872:0.08:0.035	0.022:0.808:0.096:0.074	0.011:0.863:0.083:0.043	0.02:0.795:0.101:0.085
Theropoda	0.002:0.002:0.002:0.995	0.001:0.001:0.001:0.996	0.003:0.002:0.002:0.993	0.002:0.001:0.001:0.996

Table S38a. Same as Table S30 but excluding extinct marine reptiles from the analyses (FBD tip-dating method with root age constrained).

Character	Reproduction mode + egg shell mineralisation					
Model	FBD-CER (ind.hom)	FBD-CER (ind.het)	FBD-CER (sw.hom)	FBD-CER (sw.het)	FBD-CSYM (ind.hom)	FBD-CSYM (ind.het)
Mean log marginal likelihood	-68.531	-67.703	-68.789	-67.885	-69.983	-69.137
Mean log Bayes Factor	2.228	3.883	1.710	3.519	-0.676	1.016
Amniota	0.542:0.337:0.056:0.065	0.577:0.278:0.062:0.083	0.391:0.465:0.081:0.063	0.466:0.366:0.085:0.083	0.596:0.266:0.068:0.071	0.598:0.242:0.069:0.091
Mammalia	0.806:0.103:0.046:0.046	0.774:0.114:0.056:0.056	0.8:0.106:0.047:0.047	0.776:0.114:0.055:0.055	0.791:0.123:0.044:0.042	0.763:0.133:0.051:0.053
Reptilia	0.456:0.39:0.07:0.083	0.518:0.315:0.069:0.098	0.438:0.377:0.104:0.081	0.495:0.313:0.095:0.098	0.548:0.27:0.088:0.094	0.564:0.244:0.081:0.11
Diapsida s.l./s.s.	0.161:0.155:0.31:0.373	0.226:0.133:0.258:0.383	0.133:0.121:0.429:0.317	0.201:0.111:0.343:0.345	0.178:0.089:0.347:0.385	0.227:0.092:0.283:0.399
Lepidosauria	0.129:0.077:0.648:0.145	0.175:0.088:0.597:0.14	0.19:0.064:0.629:0.117	0.247:0.073:0.567:0.114	0.143:0.059:0.636:0.163	0.186:0.073:0.599:0.142
Archelosauria	0.357:0.169:0.099:0.374	0.38:0.138:0.087:0.396	0.22:0.177:0.18:0.423	0.287:0.142:0.143:0.427	0.359:0.096:0.162:0.384	0.363:0.093:0.121:0.423
Archosauromorpha	0.722:0.131:0.069:0.078	0.681:0.129:0.07:0.12	0.611:0.154:0.135:0.1	0.59:0.147:0.122:0.14	0.713:0.08:0.11:0.096	0.668:0.091:0.098:0.143
Archosauria	0.013:0.584:0.091:0.312	0.025:0.469:0.103:0.403	0.012:0.569:0.093:0.326	0.026:0.462:0.105:0.406	0.016:0.33:0.278:0.375	0.027:0.3:0.208:0.465
Dinosauria	0.004:0.652:0.101:0.243	0.008:0.549:0.108:0.335	0.004:0.644:0.104:0.248	0.008:0.547:0.113:0.331	0.005:0.396:0.286:0.312	0.01:0.372:0.213:0.406
Saurischia	0.003:0.63:0.175:0.193	0.004:0.535:0.175:0.28	0.002:0.62:0.179:0.199	0.003:0.533:0.181:0.28	0.003:0.392:0.321:0.28	0.006:0.368:0.251:0.37

		5		3	4	5
Theropoda	0.01:0.011:0.011:0.968	0.008:0.008:0.008:0.975	0.014:0.011:0.011:0.964	0.011:0.009:0.009:0.971	0.014:0.008:0.013:0.964	0.011:0.008:0.01:0.971

Table S38b. Continuation of Table S38a.

Character	Reproduction mode + egg shell mineralisation					
Model	FBD-CSYM (sw.hom)	FBD-CSYM (sw.het)	FBD-CARD (ind.hom)	FBD-CARD (ind.het)	FBD-CARD (sw.hom)	FBD-CARD (sw.het)
Mean log marginal likelihood	-69.880	-69.038	-61.842	-61.088	-62.449	-61.219
Mean log Bayes Factor	-0.471	1.212	15.605	17.112	14.390	16.851
Amniota	0.466:0.37:0.103:0.061	0.499:0.313:0.096:0.092	1:0:0:0	1:0:0:0	0.999:0.001:0:0	0.999:0:0:0
Mammalia	0.787:0.128:0.047:0.037	0.76:0.137:0.052:0.051	1:0:0:0	1:0:0:0	1:0:0:0	1:0:0:0
Reptilia	0.546:0.256:0.119:0.08	0.556:0.232:0.106:0.106	0.999:0.001:0:0	1:0:0:0	0.998:0.002:0:0	0.999:0.001:0:0
Diapsida s.l./s.s.	0.179:0.068:0.454:0.299	0.226:0.075:0.359:0.339	0.999:0.001:0:0	1:0:0:0	0.997:0.003:0:0	0.999:0.001:0:0
Lepidosauria	0.207:0.055:0.589:0.15	0.252:0.062:0.565:0.122	0.999:0:0.001:0	1:0:0:0	0.997:0.001:0.002:0.001	0.998:0.001:0.001:0
Archelosauria	0.255:0.093:0.277:0.375	0.313:0.089:0.19:0.408	0.999:0:0:0	1:0:0:0	0.998:0.002:0:0	0.999:0.001:0:0
Archosauromorpha	0.65:0.085:0.166:0.1	0.614:0.091:0.141:0.154	1:0:0:0	1:0:0:0	0.999:0.001:0:0	0.999:0.001:0:0
Archosauria	0.015:0.292:0.376:0.317	0.026:0.27:0.266:0.438	0.053:0.759:0.151:0.037	0.071:0.774:0.094:0.062	0.06:0.69:0.223:0.026	0.154:0.577:0.238:0.03
Dinosauri	0.005:0.362	0.008:0.34:	0.022:0.813	0.031:0.837	0.029:0.74:	0.101:0.655

a	:0.386:0.247	0.273:0.379	:0.141:0.025	:0.086:0.046	0.213:0.018	:0.223:0.021
Saurischia	0.002:0.363:0.412:0.222	0.004:0.339:0.306:0.352	0.015:0.811:0.151:0.023	0.022:0.846:0.092:0.041	0.01:0.737:0.235:0.018	0.041:0.715:0.225:0.019
Theropoda	0.018:0.007:0.014:0.96	0.014:0.007:0.011:0.968	0.052:0.052:0.048:0.848	0.051:0.045:0.041:0.863	0.058:0.051:0.05:0.84	0.121:0.071:0.055:0.753

Table S38c. Continuation of Table S38b.

Character	Reproduction mode + egg shell mineralisation			
Model	FBD-ER (ind.hom)	FBD-ER (ind.het)	FBD-ER (sw.hom)	FBD-ER (sw.het)
Mean log marginal likelihood	-69.201	-68.325	-69.644	-68.671
Mean log Bayes Factor	0.888	2.639	0.000	1.947
Amniota	0.546:0.332:0.057:0.065	0.581:0.269:0.065:0.085	0.397:0.459:0.081:0.063	0.477:0.347:0.088:0.088
Mammalia	0.802:0.103:0.047:0.047	0.772:0.111:0.058:0.058	0.8:0.105:0.047:0.047	0.771:0.112:0.059:0.059
Reptilia	0.463:0.386:0.069:0.081	0.525:0.306:0.07:0.099	0.445:0.369:0.104:0.081	0.504:0.299:0.096:0.1
Diapsida s.l./s.s.	0.17:0.159:0.307:0.365	0.24:0.133:0.253:0.374	0.138:0.121:0.426:0.315	0.224:0.115:0.327:0.334
Lepidosauria	0.136:0.081:0.632:0.151	0.197:0.094:0.564:0.146	0.198:0.065:0.617:0.12	0.27:0.079:0.529:0.123
Archelosauria	0.366:0.173:0.102:0.359	0.387:0.138:0.088:0.387	0.222:0.177:0.181:0.42	0.304:0.146:0.143:0.406
Archosauromorpha	0.719:0.133:0.07:0.077	0.677:0.13:0.071:0.121	0.61:0.154:0.136:0.1	0.588:0.15:0.121:0.141
Archosauria	0.014:0.589:0.094:0.302	0.029:0.465:0.106:0.4	0.013:0.568:0.094:0.325	0.028:0.463:0.11:0.399
Dinosauria	0.005:0.653:0.105:0.237	0.009:0.543:0.11:0.338	0.004:0.642:0.106:0.248	0.01:0.542:0.117:0.332
Saurischia	0.003:0.63:0.176:0.191	0.005:0.533:0.175:0.287	0.002:0.619:0.18:0.2	0.003:0.53:0.183:0.284
Theropoda	0.011:0.012:0.012:0.964	0.009:0.009:0.009:0.973	0.015:0.012:0.012:0.961	0.012:0.009:0.009:0.971

Table S38d. Continuation of Table S38c.

Character	EER			
Model	FBD-EER ER (hom)	FBD-EER ER (het)	FBD-EER ARD (hom)	FBD-EER ARD (het)
Mean log marginal likelihood	-30.431	-29.842	-26.459	-26.203
Mean log Bayes Factor	0.000	1.178	7.943	8.455
Amniota	0.039:0.961	0.118:0.882	0.001:0.999	0.005:0.995
Mammalia	0.216:0.784	0.251:0.749	0.007:0.993	0.012:0.988
Reptilia	0.032:0.968	0.098:0.902	0.001:0.999	0.004:0.996
Diapsida s.l./s.s.	0.353:0.647	0.412:0.588	0.01:0.99	0.02:0.98
Lepidosauria	0.174:0.826	0.267:0.733	0.005:0.995	0.012:0.988
Archelosauria	0.608:0.392	0.566:0.434	0.018:0.982	0.028:0.972
Archosauromorpha	0.251:0.749	0.324:0.676	0.007:0.993	0.015:0.985
Archosauria	0.986:0.014	0.982:0.018	0.98:0.02	0.941:0.059
Dinosauria	0.995:0.005	0.995:0.005	0.993:0.007	0.973:0.027
Saurischia	0.998:0.002	0.998:0.002	0.997:0.003	0.984:0.016
Theropoda	0.987:0.013	0.991:0.009	0.98:0.02	0.944:0.056

Table S38e. Continuation of Table S38d. Nodes of Lepidosauria and Squamata fixed to a non-viviparous state.

Character	Reproduction mode + egg shell mineralisation					
Model	FBD-CER (ind.hom)	FBD-CER (ind.het)	FBD-CER (sw.hom)	FBD-CER (sw.het)	FBD-CSYM (ind.hom)	FBD-CSYM (ind.het)
Mean log marginal likelihood	-68.776	-68.032	-68.978	-68.179	-70.277	-69.479
Mean log Bayes Factor	2.133	3.621	1.728	3.328	-0.870	0.728
Amniota	0.443:0.411:0.067:0.078	0.477:0.35:0.075:0.098	0.303:0.54:0.085:0.071	0.367:0.447:0.091:0.095	0.495:0.335:0.084:0.086	0.508:0.301:0.081:0.11
Mammalia	0.79:0.11:0.05:0.05	0.77:0.119:0.056:0.056	0.79:0.112:0.049:0.049	0.763:0.123:0.057:0.057	0.774:0.134:0.046:0.045	0.755:0.14:0.049:0.055
Reptilia	0.356:0.462:0.083:0.099	0.405:0.388:0.088:0.119	0.314:0.463:0.123:0.1	0.371:0.394:0.115:0.12	0.442:0.329:0.112:0.117	0.462:0.3:0.102:0.137
Diapsida s.l./s.s.	0.086:0.164:0.337:0.41	0.118:0.143:0.307:0.43	0.051:0.131:0.46:0.358	0.086:0.126:0.386:0.40	0.091:0.096:0.384:0.42	0.122:0.1:0.326:0.453

	3	2		2	8	
Lepidosauria	0:0.082:0.756:0.162	0:0.101:0.732:0.166	0:0.078:0.78:0.142	0:0.096:0.754:0.15	0:0.064:0.755:0.18	0:0.081:0.758:0.161
Archelosauria	0.341:0.166:0.098:0.395	0.343:0.137:0.088:0.432	0.208:0.172:0.174:0.446	0.254:0.142:0.138:0.466	0.336:0.094:0.16:0.409	0.337:0.092:0.114:0.457
Archosauromorpha	0.715:0.133:0.07:0.083	0.665:0.134:0.073:0.128	0.607:0.154:0.135:0.104	0.574:0.153:0.122:0.151	0.711:0.079:0.108:0.102	0.665:0.089:0.091:0.155
Archosauria	0.012:0.573:0.088:0.327	0.023:0.464:0.101:0.411	0.011:0.56:0.088:0.341	0.021:0.461:0.099:0.419	0.013:0.323:0.264:0.4	0.022:0.293:0.184:0.5
Dinosauria	0.004:0.642:0.1:0.254	0.007:0.548:0.107:0.339	0.004:0.639:0.101:0.256	0.007:0.547:0.109:0.337	0.004:0.39:0.273:0.333	0.008:0.363:0.19:0.44
Saurischia	0.002:0.616:0.178:0.204	0.004:0.531:0.176:0.288	0.001:0.614:0.179:0.206	0.003:0.529:0.18:0.288	0.003:0.381:0.311:0.305	0.004:0.356:0.229:0.411
Theropoda	0.01:0.011:0.011:0.969	0.008:0.009:0.009:0.974	0.013:0.011:0.011:0.966	0.011:0.009:0.009:0.972	0.013:0.008:0.013:0.966	0.009:0.008:0.01:0.973

Table S38f. Continuation of Table S38e.

Character	Reproduction mode + egg shell mineralisation					
Model	FBD-CSYM (sw.hom)	FBD-CSYM (sw.het)	FBD-CARD (ind.hom)	FBD-CARD (ind.het)	FBD-CARD (sw.hom)	FBD-CARD (sw.het)
Mean log marginal likelihood	-70.191	-69.356	-68.842	-68.444	-67.963	-67.443
Mean log Bayes Factor	-0.697	0.973	2.002	2.798	3.760	4.799
Amniota	0.338:0.469:0.113:0.079	0.392:0.399:0.1:0.109	0.388:0.586:0.016:0.01	0.493:0.469:0.017:0.021	0.543:0.44:0.012:0.005	0.666:0.315:0.01:0.009
Mammalia	0.774:0.135:0.048:0.042	0.749:0.145:0.052:0.054	0.773:0.197:0.017:0.013	0.784:0.178:0.019:0.018	0.871:0.109:0.012:0.008	0.885:0.096:0.01:0.009
Reptilia	0.394:0.345:0.152:0.10	0.435:0.306:0.126:0.13	0.312:0.66:0.016:0.012	0.414:0.543:0.018:0.02	0.372:0.604:0.017:0.00	0.519:0.454:0.014:0.01

	8	3		4	8	3
Diapsida s.l./s.s.	0.063:0.084:0.495:0.358	0.099:0.091:0.399:0.411	0.216:0.648:0.077:0.058	0.306:0.52:0.078:0.096	0.258:0.628:0.077:0.037	0.408:0.478:0.064:0.049
Lepidosauria	0:0.068:0.756:0.177	0:0.081:0.761:0.158	0:0.125:0.62:0.255	0:0.167:0.571:0.262	0:0.159:0.622:0.219	0:0.194:0.582:0.224
Archelosauria	0.23:0.103:0.254:0.413	0.275:0.095:0.173:0.457	0.456:0.472:0.031:0.041	0.504:0.389:0.029:0.078	0.4:0.529:0.038:0.032	0.533:0.394:0.028:0.045
Archosauromorpha	0.634:0.092:0.158:0.115	0.588:0.099:0.138:0.175	0.716:0.255:0.018:0.01	0.718:0.237:0.019:0.027	0.651:0.319:0.021:0.009	0.716:0.251:0.017:0.016
Archosauria	0.012:0.315:0.312:0.36	0.022:0.285:0.216:0.478	0.028:0.9:0.038:0.035	0.045:0.83:0.043:0.082	0.06:0.89:0.027:0.023	0.101:0.824:0.028:0.046
Dinosauria	0.004:0.387:0.322:0.287	0.007:0.359:0.224:0.41	0.01:0.925:0.036:0.029	0.017:0.873:0.041:0.069	0.028:0.928:0.026:0.018	0.051:0.882:0.027:0.04
Saurischia	0.002:0.381:0.356:0.261	0.003:0.354:0.264:0.379	0.006:0.914:0.052:0.028	0.01:0.868:0.055:0.067	0.006:0.939:0.039:0.017	0.013:0.911:0.037:0.038
Theropoda	0.016:0.008:0.013:0.963	0.012:0.008:0.01:0.97	0.025:0.021:0.016:0.939	0.029:0.021:0.014:0.936	0.045:0.031:0.019:0.905	0.056:0.033:0.016:0.895

Table S38g. Continuation of Table S38f.

Character	Reproduction mode + egg shell mineralisation			
Model	FBD-ER (ind.hom)	FBD-ER (ind.het)	FBD-ER (sw.hom)	FBD-ER (sw.het)
Mean log marginal likelihood	-69.466	-68.676	-69.843	-69.011
Mean log Bayes Factor	0.752	2.333	0.000	1.664
Amniota	0.442:0.412:0.068:0.078	0.477:0.345:0.077:0.101	0.307:0.537:0.085:0.071	0.372:0.431:0.095:0.102
Mammalia	0.795:0.108:0.048:0.048	0.758:0.121:0.06:0.06	0.795:0.109:0.048:0.048	0.762:0.12:0.059:0.059
Reptilia	0.354:0.465:0.083:0.098	0.41:0.381:0.087:0.122	0.315:0.466:0.121:0.098	0.374:0.384:0.117:0.126
Diapsida s.l./s.s.	0.086:0.168:0.339:0.407	0.124:0.146:0.295:0.435	0.053:0.136:0.457:0.355	0.095:0.131:0.373:0.402

Lepidosauria	0:0.087:0.748:0.166	0:0.107:0.718:0.175	0:0.084:0.765:0.152	0:0.102:0.738:0.16
Archelosauria	0.347:0.168:0.099:0.385	0.342:0.139:0.088:0.431	0.212:0.176:0.177:0.435	0.264:0.144:0.138:0.454
Archosauromorpha	0.716:0.133:0.07:0.081	0.657:0.138:0.074:0.13	0.602:0.157:0.137:0.104	0.573:0.154:0.123:0.15
Archosauria	0.013:0.579:0.09:0.318	0.023:0.46:0.101:0.415	0.012:0.564:0.09:0.333	0.024:0.458:0.1:0.419
Dinosauria	0.004:0.647:0.101:0.248	0.007:0.54:0.109:0.343	0.004:0.638:0.103:0.254	0.007:0.545:0.11:0.338
Saurischia	0.003:0.622:0.177:0.198	0.004:0.525:0.179:0.292	0.002:0.614:0.18:0.204	0.003:0.528:0.18:0.29
Theropoda	0.01:0.011:0.011:0.969	0.008:0.009:0.009:0.975	0.014:0.011:0.011:0.963	0.011:0.009:0.009:0.971

Table S39a. Same as Table S31 but excluding extinct marine reptiles from the analyses (FBD tip-dating method with root age and node age of major extant clades constrained).

Character	Reproduction mode + egg shell mineralisation					
Model	FBD_c-CER (ind.hom)	FBD_c-CER (ind.het)	FBD_c-CER (sw.hom)	FBD_c-CER (sw.het)	FBD_c-CSYM (ind.hom)	FBD_c-CSYM (ind.het)
Mean log marginal likelihood	-67.388	-66.693	-67.481	-66.786	-68.649	-67.952
Mean log Bayes Factor	1.964	3.355	1.778	3.168	-0.557	0.836
Amniota	0.462:0.39:0.068:0.079	0.508:0.325:0.07:0.097	0.347:0.495:0.087:0.07	0.409:0.408:0.09:0.093	0.512:0.329:0.072:0.087	0.537:0.289:0.065:0.109
Mammalia	0.741:0.141:0.059:0.059	0.71:0.151:0.07:0.07	0.746:0.139:0.057:0.057	0.709:0.153:0.069:0.069	0.747:0.159:0.046:0.047	0.712:0.172:0.054:0.061
Reptilia	0.375:0.42:0.094:0.111	0.444:0.347:0.084:0.124	0.375:0.402:0.124:0.099	0.432:0.339:0.11:0.119	0.457:0.301:0.111:0.131	0.501:0.263:0.09:0.145
Diapsida s.l./s.s.	0.063:0.111:0.377:0.449	0.119:0.102:0.3:0.478	0.057:0.09:0.488:0.365	0.109:0.089:0.388:0.415	0.07:0.058:0.416:0.457	0.119:0.06:0.321:0.5
Lepidosauria	0.108:0.077:0.704:0.111	0.157:0.091:0.632:0.12	0.176:0.066:0.667:0.092	0.223:0.074:0.607:0.096	0.119:0.054:0.711:0.116	0.156:0.061:0.672:0.111
Archelosauria	0.217:0.143:0.098:0.542	0.25:0.12:0.086:0.545	0.136:0.147:0.156:0.561	0.195:0.123:0.13:0.552	0.219:0.071:0.179:0.532	0.248:0.067:0.119:0.566
Archosauromorpha	0.62:0.151:0.092:0.138	0.572:0.142:0.088:0.198	0.509:0.171:0.161:0.16	0.488:0.156:0.141:0.215	0.613:0.076:0.141:0.171	0.57:0.078:0.112:0.24
Archosauria	0.009:0.466:0.091:0.434	0.02:0.369:0.101:0.51	0.009:0.469:0.091:0.431	0.022:0.377:0.103:0.499	0.01:0.221:0.259:0.51	0.021:0.198:0.181:0.6
Dinosauria	0.006:0.525:0.114:0.355	0.01:0.438:0.116:0.437	0.006:0.531:0.116:0.348	0.009:0.453:0.119:0.419	0.007:0.278:0.264:0.452	0.01:0.259:0.186:0.545
Saurischia	0.003:0.524:0.201:0.27	0.005:0.444:0.191:0.36	0.002:0.526:0.203:0.26	0.003:0.453:0.196:0.34	0.004:0.275:0.303:0.41	0.005:0.258:0.228:0.50

	2		9	8	9	9
Theropoda	0.007:0.007:0.007:0.979	0.006:0.006:0.006:0.981	0.008:0.007:0.007:0.978	0.007:0.006:0.006:0.981	0.007:0.005:0.007:0.98	0.006:0.005:0.007:0.982

Table S39b. Continuation of Table S39a.

Character	Reproduction mode + egg shell mineralisation					
Model	FBD_c-CSYM (sw.hom)	FBD_c-CSYM (sw.het)	FBD_c-CARD (ind.hom)	FBD_c-CARD (ind.het)	FBD_c-CARD (sw.hom)	FBD_c-CARD (sw.het)
Mean log marginal likelihood	-68.459	-67.792	-61.105	-60.352	-61.086	-59.965
Mean log Bayes Factor	-0.178	1.158	14.532	16.037	14.569	16.811
Amniota	0.389:0.438:0.098:0.075	0.43:0.375:0.091:0.104	0.999:0.001:0:0	1:0:0:0	0.999:0.001:0:0	1:0:0:0
Mammalia	0.746:0.161:0.051:0.042	0.712:0.173:0.057:0.059	0.999:0.001:0:0	1:0:0:0	1:0:0:0	1:0:0:0
Reptilia	0.463:0.29:0.14:0.107	0.487:0.266:0.117:0.13	0.999:0.001:0:0	1:0:0:0	0.999:0.001:0:0	0.999:0.001:0:0
Diapsida s.l./s.s.	0.08:0.048:0.53:0.342	0.118:0.055:0.413:0.414	0.998:0.001:0:0	1:0:0:0	0.998:0.002:0:0	0.999:0.001:0:0
Lepidosauria	0.191:0.052:0.652:0.105	0.226:0.055:0.624:0.095	0.998:0:0.001:0	1:0:0:0	0.998:0:0.001:0	0.999:0:0.001:0
Archelosauria	0.154:0.07:0.286:0.49	0.204:0.07:0.189:0.537	0.999:0.001:0:0	1:0:0:0	0.998:0.002:0:0	0.999:0.001:0:0
Archosauromorpha	0.539:0.081:0.204:0.176	0.497:0.087:0.169:0.247	0.999:0.001:0:0	1:0:0:0	0.999:0.001:0:0	0.999:0:0:0
Archosauria	0.011:0.209:0.346:0.435	0.022:0.2:0.235:0.543	0.061:0.734:0.156:0.048	0.079:0.734:0.103:0.084	0.059:0.619:0.292:0.031	0.196:0.48:0.292:0.032
Dinosauria	0.007:0.266:0.35:0.376	0.011:0.263:0.242:0.484	0.029:0.789:0.148:0.034	0.042:0.795:0.098:0.065	0.038:0.656:0.282:0.024	0.145:0.552:0.278:0.025

Saurischia	0.003:0.266:0.387:0.345	0.004:0.26:0.286:0.45	0.016:0.797:0.159:0.029	0.027:0.814:0.102:0.057	0.011:0.653:0.314:0.022	0.072:0.623:0.283:0.023
Theropoda	0.01:0.004:0.008:0.978	0.009:0.005:0.007:0.979	0.046:0.046:0.04:0.868	0.05:0.042:0.036:0.872	0.048:0.041:0.04:0.871	0.125:0.069:0.047:0.76

Table S39c. Continuation of Table S39b.

Character	Reproduction mode + egg shell mineralisation			
Model	FBD_c-ER (ind.hom)	FBD_c-ER (ind.het)	FBD_c-ER (sw.hom)	FBD_c-ER (sw.het)
Mean log marginal likelihood	-68.099	-67.365	-68.371	-67.630
Mean log Bayes Factor	0.543	2.011	0.000	1.481
Amniota	0.465:0.387:0.069:0.079	0.51:0.317:0.072:0.101	0.352:0.49:0.088:0.07	0.42:0.39:0.093:0.097
Mammalia	0.742:0.139:0.06:0.06	0.71:0.147:0.071:0.071	0.743:0.139:0.059:0.059	0.702:0.151:0.073:0.073
Reptilia	0.376:0.42:0.094:0.11	0.447:0.34:0.086:0.127	0.379:0.4:0.124:0.098	0.442:0.33:0.11:0.118
Diapsida s.l./s.s.	0.066:0.116:0.376:0.442	0.126:0.105:0.296:0.473	0.061:0.094:0.485:0.36	0.119:0.09:0.376:0.415
Lepidosauria	0.113:0.079:0.693:0.115	0.169:0.094:0.612:0.125	0.185:0.068:0.653:0.095	0.244:0.077:0.578:0.1
Archelosauria	0.22:0.15:0.101:0.529	0.253:0.123:0.088:0.536	0.139:0.153:0.161:0.548	0.201:0.125:0.131:0.543
Archosauromorpha	0.616:0.155:0.093:0.135	0.571:0.143:0.088:0.197	0.505:0.174:0.163:0.158	0.483:0.158:0.141:0.218
Archosauria	0.01:0.475:0.094:0.421	0.022:0.372:0.104:0.502	0.009:0.477:0.093:0.421	0.023:0.375:0.105:0.498
Dinosauria	0.006:0.53:0.117:0.347	0.009:0.443:0.115:0.432	0.006:0.535:0.118:0.34	0.01:0.445:0.121:0.423
Saurischia	0.003:0.528:0.202:0.268	0.005:0.447:0.19:0.358	0.002:0.532:0.203:0.263	0.004:0.445:0.197:0.354
Theropoda	0.007:0.007:0.007:0.979	0.006:0.007:0.007:0.98	0.009:0.007:0.007:0.977	0.008:0.006:0.006:0.98

Table S39d. Continuation of Table S39c.

Character	EER			
Model	FBD_c-EER ER	FBD_c-EER ER	FBD_c-EER	FBD_c-EER

	(hom)	(het)	ARD (hom)	ARD (het)
Mean log marginal likelihood	-30.188	-29.658	-26.035	-25.784
Mean log Bayes Factor	0.000	1.061	8.307	8.808
Amniota	0.046:0.954	0.116:0.884	0.001:0.999	0.003:0.997
Mammalia	0.279:0.721	0.312:0.688	0.008:0.992	0.011:0.989
Reptilia	0.045:0.955	0.101:0.899	0.001:0.999	0.003:0.997
Diapsida s.l./s.s.	0.495:0.505	0.512:0.488	0.015:0.985	0.02:0.98
Lepidosauria	0.227:0.773	0.299:0.701	0.007:0.993	0.011:0.989
Archelosauria	0.727:0.273	0.668:0.332	0.022:0.978	0.028:0.972
Archosauromorpha	0.356:0.644	0.412:0.588	0.01:0.99	0.015:0.985
Archosauria	0.984:0.016	0.978:0.022	0.976:0.024	0.925:0.075
Dinosauria	0.994:0.006	0.995:0.005	0.991:0.009	0.966:0.034
Saurischia	0.998:0.002	0.998:0.002	0.997:0.003	0.98:0.02
Theropoda	0.985:0.015	0.991:0.009	0.977:0.023	0.933:0.067

Table S39e. Continuation of Table S39d. Nodes of Lepidosauria and Squamata fixed to a non-viviparous state.

Character	Reproduction mode + egg shell mineralisation					
Model	FBD_c-CER (ind.hom)	FBD_c-CER (ind.het)	FBD_c-CER (sw.hom)	FBD_c-CER (sw.het)	FBD_c-CSYM (ind.hom)	FBD_c-CSYM (ind.het)
Mean log marginal likelihood	-67.537	-66.941	-67.611	-67.000	-68.828	-68.211
Mean log Bayes Factor	1.925	3.118	1.776	2.999	-0.657	0.578
Amniota	0.392:0.442:0.077:0.089	0.424:0.384:0.08:0.111	0.291:0.541:0.092:0.077	0.336:0.466:0.095:0.103	0.436:0.382:0.079:0.102	0.457:0.341:0.077:0.125
Mammalia	0.743:0.141:0.058:0.058	0.712:0.154:0.067:0.067	0.746:0.141:0.057:0.057	0.712:0.156:0.066:0.066	0.747:0.162:0.044:0.048	0.722:0.171:0.049:0.058
Reptilia	0.304:0.467:0.105:0.124	0.353:0.403:0.1:0.144	0.298:0.451:0.138:0.113	0.342:0.396:0.125:0.136	0.379:0.343:0.124:0.154	0.408:0.31:0.11:0.172
Diapsida s.l./s.s.	0.022:0.114:0.396:0.46	0.043:0.107:0.338:0.51	0.014:0.094:0.502:0.38	0.03:0.092:0.416:0.462	0.023:0.059:0.428:0.49	0.04:0.062:0.356:0.542

	8	3	9			
Lepidosauria	0:0.086:0.791:0.123	0:0.104:0.758:0.138	0:0.082:0.804:0.114	0:0.098:0.777:0.125	0:0.059:0.819:0.122	0:0.069:0.805:0.126
Archelosauria	0.208:0.141:0.096:0.555	0.223:0.119:0.084:0.574	0.129:0.142:0.151:0.578	0.169:0.117:0.122:0.592	0.207:0.069:0.164:0.56	0.214:0.066:0.118:0.603
Archosauromorpha	0.618:0.151:0.091:0.141	0.55:0.147:0.09:0.213	0.506:0.17:0.16:0.165	0.469:0.159:0.141:0.232	0.617:0.075:0.13:0.179	0.553:0.08:0.112:0.255
Archosauria	0.008:0.465:0.088:0.439	0.016:0.367:0.097:0.52	0.008:0.463:0.088:0.441	0.017:0.368:0.095:0.52	0.009:0.221:0.231:0.54	0.016:0.193:0.176:0.615
Dinosauria	0.005:0.521:0.113:0.361	0.008:0.438:0.114:0.439	0.006:0.525:0.114:0.355	0.009:0.446:0.114:0.432	0.006:0.278:0.236:0.479	0.008:0.249:0.182:0.561
Saurischia	0.003:0.519:0.202:0.277	0.004:0.44:0.194:0.361	0.002:0.52:0.203:0.276	0.003:0.448:0.193:0.355	0.003:0.275:0.279:0.443	0.005:0.247:0.225:0.524
Theropoda	0.006:0.007:0.007:0.981	0.005:0.006:0.006:0.983	0.008:0.007:0.007:0.979	0.007:0.006:0.006:0.981	0.006:0.005:0.007:0.982	0.006:0.005:0.006:0.984

Table S39f. Continuation of Table S39e.

Character	Reproduction mode + egg shell mineralisation					
Model	FBD_c-CSYM (sw.hom)	FBD_c-CSYM (sw.het)	FBD_c-CARD (ind.hom)	FBD_c-CARD (ind.het)	FBD_c-CARD (sw.hom)	FBD_c-CARD (sw.het)
Mean log marginal likelihood	-68.643	-68.02	-67.970	-67.667	-67.182	-66.912
Mean log Bayes Factor	-0.287	0.96	1.058	1.666	2.636	3.176
Amniota	0.306:0.504:0.103:0.087	0.347:0.438:0.095:0.12	0.28:0.684:0.02:0.016	0.373:0.572:0.021:0.034	0.414:0.563:0.014:0.009	0.534:0.432:0.016:0.018
Mammalia	0.748:0.161:0.047:0.043	0.716:0.176:0.052:0.057	0.693:0.269:0.022:0.017	0.707:0.244:0.024:0.026	0.792:0.182:0.015:0.011	0.806:0.162:0.016:0.015
Reptilia	0.365:0.346:0.161:0.12	0.394:0.313:0.135:0.15	0.203:0.75:0.025:0.022	0.295:0.636:0.025:0.04	0.235:0.731:0.021:0.01	0.376:0.576:0.023:0.02

	9	8		3	3	4
Diapsida s.l./s.s.	0.016:0.054:0.544:0.386	0.031:0.06:0.436:0.473	0.084:0.722:0.113:0.081	0.149:0.596:0.106:0.149	0.103:0.752:0.098:0.047	0.226:0.591:0.098:0.084
Lepidosauria	0:0.064:0.811:0.125	0:0.07:0.812:0.118	0:0.122:0.679:0.198	0:0.161:0.606:0.232	0:0.135:0.693:0.172	0:0.178:0.623:0.199
Archelosauria	0.144:0.072:0.255:0.529	0.17:0.069:0.173:0.588	0.285:0.595:0.043:0.077	0.337:0.485:0.037:0.14	0.225:0.678:0.045:0.051	0.356:0.518:0.041:0.085
Archosauromorpha	0.532:0.085:0.187:0.196	0.477:0.088:0.161:0.274	0.582:0.366:0.028:0.024	0.579:0.333:0.029:0.059	0.497:0.456:0.029:0.018	0.561:0.372:0.029:0.038
Archosauria	0.009:0.219:0.295:0.477	0.018:0.193:0.208:0.581	0.019:0.877:0.04:0.064	0.032:0.784:0.046:0.138	0.035:0.898:0.028:0.039	0.078:0.811:0.033:0.078
Dinosauria	0.006:0.275:0.304:0.414	0.009:0.255:0.214:0.522	0.011:0.89:0.041:0.058	0.016:0.813:0.045:0.126	0.025:0.91:0.029:0.035	0.048:0.846:0.034:0.071
Saurischia	0.002:0.272:0.344:0.382	0.004:0.251:0.255:0.49	0.005:0.88:0.061:0.053	0.01:0.811:0.062:0.117	0.005:0.918:0.044:0.033	0.013:0.87:0.049:0.068
Theropoda	0.008:0.005:0.007:0.98	0.007:0.005:0.007:0.981	0.013:0.012:0.008:0.968	0.019:0.014:0.009:0.958	0.022:0.017:0.01:0.951	0.036:0.023:0.01:0.931

Table S39g. Continuation of Table S39f.

Character	Reproduction mode + egg shell mineralisation			
Model	FBD_c-ER (ind.hom)	FBD_c-ER (ind.het)	FBD_c-ER (sw.hom)	FBD_c-ER (sw.het)
Mean log marginal likelihood	-68.264	-67.629	-68.5	-67.858
Mean log Bayes Factor	0.471	1.741	0.0	1.282
Amniota	0.394:0.439:0.077:0.089	0.424:0.374:0.085:0.117	0.295:0.537:0.092:0.076	0.342:0.456:0.096:0.106
Mammalia	0.743:0.14:0.059:0.059	0.706:0.152:0.071:0.071	0.744:0.14:0.058:0.058	0.704:0.156:0.07:0.07
Reptilia	0.306:0.467:0.105:0.122	0.354:0.395:0.102:0.149	0.301:0.453:0.136:0.11	0.348:0.391:0.124:0.137
Diapsida s.l./s.s.	0.024:0.118:0.395:0.463	0.044:0.109:0.328:0.519	0.016:0.098:0.502:0.384	0.034:0.096:0.408:0.462

Lepidosauria	0:0.09:0.78:0.13	0:0.11:0.745:0.145	0:0.086:0.793:0.121	0:0.105:0.759:0.136
Archelosauria	0.213:0.145:0.1:0.542	0.222:0.12:0.084:0.574	0.136:0.147:0.156:0.561	0.175:0.12:0.126:0.578
Archosauromorpha	0.615:0.154:0.093:0.138	0.548:0.149:0.09:0.213	0.507:0.172:0.161:0.16	0.466:0.158:0.143:0.233
Archosauria	0.009:0.47:0.092:0.429	0.017:0.371:0.097:0.515	0.009:0.47:0.091:0.43	0.018:0.367:0.098:0.517
Dinosauria	0.006:0.524:0.116:0.354	0.008:0.442:0.112:0.437	0.006:0.531:0.116:0.347	0.009:0.442:0.117:0.432
Saurischia	0.003:0.522:0.203:0.271	0.005:0.445:0.192:0.358	0.002:0.527:0.203:0.268	0.003:0.444:0.196:0.356
Theropoda	0.006:0.007:0.007:0.981	0.006:0.006:0.006:0.983	0.008:0.007:0.007:0.978	0.007:0.006:0.006:0.981

Table S40a. Same as Table S28 but adding extinct marine reptiles as sister taxon to Archelosauria (equal dating method).

Character	Reproduction mode + egg shell mineralisation					
Model	equal-CER (ind.hom)	equal-CER (ind.het)	equal-CER (sw.hom)	equal-CER (sw.het)	equal-CSYM (ind.hom)	equal-CSYM (ind.het)
Mean log marginal likelihood	-70.280	-69.702	-71.920	-71.133	-71.516	-70.958
Mean log Bayes Factor	4.212	5.369	0.932	2.507	1.740	2.857
Amniota	0.977:0.021:0.001:0.001	0.971:0.025:0.002:0.002	0.939:0.055:0.003:0.002	0.939:0.053:0.004:0.003	0.982:0.015:0.001:0.002	0.977:0.019:0.002:0.002
Mammalia	0.727:0.145:0.064:0.064	0.72:0.15:0.065:0.065	0.72:0.143:0.069:0.069	0.718:0.143:0.07:0.07	0.724:0.169:0.048:0.059	0.722:0.167:0.05:0.061
Reptilia	0.972:0.026:0.001:0.001	0.97:0.027:0.001:0.001	0.961:0.036:0.002:0.002	0.958:0.037:0.002:0.002	0.981:0.017:0.001:0.001	0.978:0.019:0.001:0.001
Diapsida s.l./s.s.	0.748:0.016:0.107:0.13	0.797:0.013:0.093:0.097	0.675:0.019:0.143:0.164	0.749:0.015:0.11:0.126	0.759:0.01:0.113:0.118	0.804:0.009:0.095:0.092
Lepidosauria	0.055:0.033:0.86:0.053	0.08:0.042:0.822:0.056	0.153:0.036:0.757:0.055	0.18:0.04:0.728:0.052	0.064:0.025:0.858:0.053	0.086:0.029:0.833:0.052
Marine reptiles + Archelosauria	0.973:0.003:0.002:0.021	0.97:0.003:0.003:0.024	0.958:0.006:0.005:0.031	0.959:0.005:0.005:0.031	0.973:0.003:0.004:0.021	0.969:0.003:0.003:0.025
Archelosauria	0.323:0.051:0.027:0.598	0.447:0.036:0.021:0.495	0.255:0.072:0.055:0.617	0.419:0.053:0.046:0.482	0.338:0.033:0.066:0.563	0.453:0.025:0.043:0.479
Archosauromorpha	0.892:0.043:0.018:0.048	0.887:0.034:0.015:0.064	0.836:0.064:0.038:0.062	0.834:0.054:0.034:0.079	0.891:0.023:0.026:0.06	0.881:0.021:0.023:0.075
Archosauria	0.002:0.421:0.044:0.533	0.004:0.315:0.047:0.633	0.003:0.465:0.058:0.475	0.006:0.361:0.067:0.565	0.002:0.218:0.144:0.636	0.005:0.175:0.125:0.695
Dinosauri	0.001:0.642	0.002:0.525	0.002:0.655	0.003:0.545	0.001:0.357	0.002:0.31:

a	:0.064:0.293	:0.07:0.403	:0.077:0.266	:0.09:0.361	:0.155:0.486	0.14:0.548
Saurischia	0.001:0.593:0.149:0.257	0.002:0.49:0.152:0.356	0.001:0.603:0.159:0.237	0.001:0.505:0.171:0.323	0.001:0.329:0.195:0.475	0.002:0.289:0.181:0.529
Theropoda	0.011:0.012:0.012:0.965	0.01:0.01:0.01:0.969	0.018:0.016:0.016:0.95	0.015:0.013:0.013:0.958	0.013:0.011:0.013:0.963	0.011:0.01:0.012:0.967

Table S40b. Continuation of Table S40a.

Character	Reproduction mode + egg shell mineralisation					
Model	equal-CSYM (sw.hom)	equal-CSYM (sw.het)	equal-CARD (ind.hom)	equal-CARD (ind.het)	equal-CARD (sw.hom)	equal-CARD (sw.het)
Mean log marginal likelihood	-72.400	-71.817	-63.082	-62.151	-63.934	-62.410
Mean log Bayes Factor	-0.028	1.139	18.608	20.471	16.904	19.952
Amniota	0.965:0.029:0.004:0.002	0.956:0.035:0.005:0.004	1:0:0:0	1:0:0:0	1:0:0:0	1:0:0:0
Mammalia	0.712:0.176:0.067:0.044	0.712:0.17:0.063:0.055	1:0:0:0	1:0:0:0	1:0:0:0	1:0:0:0
Reptilia	0.977:0.02:0.001:0.001	0.971:0.025:0.002:0.002	1:0:0:0	1:0:0:0	0.999:0.001:0:0	1:0:0:0
Diapsida s.l./s.s.	0.755:0.011:0.132:0.101	0.775:0.011:0.113:0.101	1:0:0:0	1:0:0:0	0.999:0.001:0:0	1:0:0:0
Lepidosauria	0.188:0.035:0.696:0.081	0.194:0.035:0.712:0.059	1:0:0:0	0.999:0:0:0	0.998:0:0.001:0	0.999:0:0:0
Marine reptiles + Archelosauria	0.958:0.003:0.016:0.023	0.961:0.004:0.01:0.026	1:0:0:0	1:0:0:0	1:0:0:0	1:0:0:0
Archelos	0.278:0.031	0.451:0.03:	1:0:0:0	1:0:0:0	0.999:0.001	1:0:0:0

auria	:0.273:0.418	0.133:0.386			:0:0	
Archosauromorpha	0.862:0.023:0.07:0.045	0.854:0.024:0.053:0.07	1:0:0:0	1:0:0:0	1:0:0:0	1:0:0:0
Archosauria	0.003:0.154:0.503:0.34	0.008:0.162:0.337:0.493	0.012:0.939:0.036:0.013	0.035:0.875:0.059:0.032	0.011:0.733:0.251:0.005	0.126:0.554:0.312:0.008
Dinosauria	0.002:0.253:0.515:0.231	0.003:0.269:0.357:0.37	0.008:0.948:0.034:0.009	0.025:0.897:0.054:0.024	0.012:0.749:0.236:0.004	0.117:0.592:0.286:0.006
Saurischia	0.001:0.24:0.529:0.23	0.002:0.253:0.384:0.362	0.005:0.946:0.04:0.009	0.02:0.911:0.048:0.021	0.003:0.713:0.28:0.004	0.054:0.65:0.291:0.005
Theropoda	0.023:0.008:0.018:0.951	0.019:0.009:0.015:0.956	0.058:0.059:0.049:0.834	0.086:0.062:0.045:0.807	0.06:0.057:0.054:0.829	0.183:0.107:0.083:0.627

Table S40c. Continuation of Table S40b.

Character	Reproduction mode + egg shell mineralisation			
Model	equal-ER (ind.hom)	equal-ER (ind.het)	equal-ER (sw.hom)	equal-ER (sw.het)
Mean log marginal likelihood	-71.093	-70.519	-72.386	-71.642
Mean log Bayes Factor	2.587	3.734	0	1.488
Amniota	0.976:0.021:0.001:0.001	0.969:0.026:0.002:0.002	0.941:0.053:0.003:0.002	0.938:0.053:0.005:0.004
Mammalia	0.726:0.144:0.065:0.065	0.717:0.148:0.068:0.068	0.716:0.142:0.071:0.071	0.712:0.143:0.073:0.073
Reptilia	0.972:0.026:0.001:0.001	0.966:0.03:0.001:0.002	0.962:0.035:0.001:0.002	0.957:0.038:0.002:0.003
Diapsida s.l./s.s.	0.752:0.016:0.105:0.127	0.787:0.014:0.097:0.102	0.694:0.019:0.134:0.153	0.753:0.015:0.108:0.124
Lepidosauria	0.058:0.035:0.851:0.056	0.086:0.044:0.811:0.059	0.173:0.04:0.723:0.064	0.204:0.043:0.697:0.056
Marine reptiles + Archelosauria	0.973:0.003:0.002:0.021	0.966:0.004:0.003:0.027	0.957:0.006:0.005:0.031	0.957:0.006:0.005:0.033
Archelosauria	0.328:0.054:0.028:0.59	0.436:0.039:0.023:0.502	0.263:0.084:0.061:0.591	0.426:0.054:0.047:0.473

Archosauromorpha	0.891:0.044:0.018:0.047	0.879:0.037:0.017:0.067	0.828:0.071:0.04:0.061	0.831:0.055:0.033:0.081
Archosauria	0.002:0.428:0.046:0.523	0.005:0.319:0.052:0.624	0.003:0.488:0.063:0.445	0.007:0.364:0.068:0.561
Dinosauria	0.001:0.644:0.066:0.288	0.003:0.525:0.074:0.398	0.002:0.669:0.08:0.25	0.003:0.545:0.09:0.362
Saurischia	0.001:0.595:0.151:0.254	0.002:0.494:0.153:0.351	0.001:0.62:0.157:0.222	0.001:0.512:0.166:0.32
Theropoda	0.012:0.013:0.013:0.963	0.011:0.011:0.011:0.966	0.022:0.018:0.018:0.941	0.017:0.014:0.014:0.955

Table S40d. Continuation of Table S40c.

Character	EER			
Model	equal-EER ER (hom)	equal-EER ER (het)	equal-EER ARD (hom)	equal-EER ARD (het)
Mean log marginal likelihood	-31.41	-31.381	-27.643	-27.477
Mean log Bayes Factor	0	0.059	7.535	7.866
Amniota	0.002:0.998	0.003:0.997	0:1	0:1
Mammalia	0.27:0.73	0.278:0.722	0.016:0.984	0.015:0.985
Reptilia	0:1	0:1	0:1	0:1
Diapsida s.l./s.s.	0.007:0.993	0.013:0.987	0:1	0.001:0.999
Lepidosauria	0.082:0.918	0.116:0.884	0.005:0.995	0.007:0.993
Marine reptiles + Archelosauria	0.026:0.974	0.033:0.967	0.001:0.999	0.002:0.998
Archelosauria	0.686:0.314	0.566:0.434	0.04:0.96	0.033:0.967
Archosauromorpha	0.103:0.897	0.119:0.881	0.006:0.994	0.007:0.993
Archosauria	0.998:0.002	0.997:0.003	0.997:0.003	0.978:0.022
Dinosauria	0.999:0.001	0.999:0.001	0.998:0.002	0.984:0.016
Saurischia	0.999:0.001	0.999:0.001	0.999:0.001	0.989:0.011
Theropoda	0.99:0.01	0.991:0.009	0.982:0.018	0.941:0.059

Table S40e. Continuation of Table S40d. Nodes of Lepidosauria and Squamata fixed to a non-viviparous state.

Character	Reproduction mode + egg shell mineralisation					
Model	equal-CER (ind.hom)	equal-CER (ind.het)	equal-CER (sw.hom)	equal-CER (sw.het)	equal-CSYM (ind.hom)	equal-CSYM (ind.het)
Mean log marginal	-70.759	-70.149	-72.503	-71.632	-72.020	-71.426

likelihood						
Mean log Bayes Factor	4.516	5.736	1.029	2.770	1.994	3.182
Amniota	0.964:0.033:0.001:0.002	0.959:0.037:0.002:0.002	0.894:0.099:0.003:0.004	0.9:0.091:0.004:0.005	0.974:0.023:0.001:0.002	0.967:0.028:0.002:0.002
Mammalia	0.729:0.146:0.063:0.063	0.731:0.148:0.061:0.061	0.724:0.145:0.065:0.065	0.722:0.148:0.065:0.065	0.727:0.171:0.044:0.058	0.728:0.168:0.045:0.058
Reptilia	0.956:0.041:0.001:0.002	0.954:0.042:0.002:0.002	0.928:0.066:0.003:0.003	0.929:0.064:0.003:0.004	0.972:0.025:0.001:0.002	0.966:0.03:0.002:0.002
Diapsida s.l./s.s.	0.646:0.023:0.149:0.182	0.711:0.018:0.132:0.139	0.499:0.029:0.219:0.253	0.621:0.021:0.165:0.192	0.651:0.014:0.164:0.172	0.719:0.013:0.14:0.128
Lepidosauria	0:0.033:0.914:0.053	0:0.035:0.918:0.047	0:0.038:0.904:0.058	0:0.04:0.906:0.053	0:0.023:0.927:0.049	0:0.026:0.93:0.044
Marine reptiles + Archelosauria	0.973:0.003:0.002:0.022	0.967:0.003:0.003:0.027	0.958:0.005:0.004:0.033	0.954:0.005:0.004:0.037	0.973:0.002:0.003:0.021	0.968:0.003:0.003:0.026
Archelosauria	0.319:0.048:0.025:0.608	0.435:0.034:0.02:0.512	0.234:0.063:0.047:0.657	0.389:0.042:0.035:0.534	0.328:0.028:0.058:0.586	0.436:0.023:0.037:0.505
Archosauromorpha	0.894:0.041:0.017:0.048	0.885:0.033:0.015:0.067	0.836:0.062:0.035:0.067	0.832:0.05:0.028:0.09	0.894:0.021:0.022:0.062	0.888:0.018:0.019:0.075
Archosauria	0.002:0.41:0.041:0.547	0.004:0.307:0.045:0.644	0.002:0.441:0.049:0.507	0.005:0.326:0.053:0.615	0.002:0.201:0.124:0.673	0.005:0.167:0.107:0.721
Dinosauria	0.001:0.639:0.062:0.298	0.002:0.52:0.069:0.41	0.001:0.65:0.07:0.279	0.002:0.529:0.076:0.392	0.001:0.345:0.136:0.518	0.002:0.308:0.121:0.569
Saurischia	0.001:0.589:0.148:0.263	0.002:0.487:0.152:0.359	0.001:0.597:0.153:0.25	0.001:0.493:0.157:0.349	0.001:0.315:0.177:0.507	0.001:0.287:0.165:0.547
Theropoda	0.01:0.011:0.011:0.967	0.009:0.01:0.01:0.971	0.015:0.014:0.014:0.957	0.013:0.011:0.011:0.965	0.011:0.01:0.012:0.967	0.011:0.009:0.011:0.969

Table S40f. Continuation of Table S40e.

Character	Reproduction mode + egg shell mineralisation					
Model	equal-CSYM (sw.hom)	equal-CSYM (sw.het)	equal-CARD (ind.hom)	equal-CARD (ind.het)	equal-CARD (sw.hom)	equal-CARD (sw.het)
Mean log marginal likelihood	-73.198	-72.404	-71.109	-70.476	-70.648	-69.943
Mean log Bayes Factor	-0.362	1.227	3.817	5.082	4.739	6.148
Amniota	0.931:0.062:0.004:0.004	0.931:0.06:0.004:0.006	0.877:0.122:0:0	0.924:0.075:0:0.001	0.937:0.062:0:0	0.957:0.042:0:0
Mammalia	0.72:0.175:0.053:0.052	0.719:0.171:0.051:0.059	0.77:0.193:0.019:0.018	0.809:0.158:0.015:0.018	0.896:0.079:0.013:0.012	0.904:0.074:0.011:0.011
Reptilia	0.95:0.045:0.003:0.003	0.95:0.043:0.003:0.004	0.824:0.175:0:0	0.889:0.11:0:0.001	0.838:0.161:0:0	0.899:0.101:0:0
Diapsida s.l./s.s.	0.525:0.022:0.244:0.209	0.64:0.016:0.172:0.172	0.785:0.163:0.028:0.024	0.852:0.097:0.027:0.024	0.81:0.161:0.018:0.01	0.88:0.097:0.013:0.01
Lepidosauria	0:0.033:0.899:0.068	0:0.032:0.914:0.054	0:0.113:0.757:0.13	0:0.128:0.754:0.118	0:0.157:0.757:0.086	0:0.16:0.751:0.089
Marine reptiles + Archelosauria	0.958:0.004:0.011:0.028	0.956:0.004:0.007:0.033	0.949:0.045:0.002:0.004	0.97:0.023:0.001:0.006	0.974:0.024:0.001:0.002	0.98:0.017:0.001:0.003
Archelosauria	0.25:0.04:0.175:0.535	0.411:0.03:0.082:0.478	0.628:0.292:0.012:0.068	0.746:0.171:0.009:0.075	0.657:0.307:0.011:0.025	0.789:0.178:0.007:0.025
Archosauromorpha	0.847:0.027:0.049:0.077	0.84:0.025:0.036:0.098	0.897:0.092:0.003:0.007	0.918:0.067:0.003:0.012	0.919:0.075:0.003:0.003	0.934:0.059:0.002:0.005
Archosauria	0.003:0.179:0.303:0.515	0.006:0.158:0.191:0.645	0.009:0.906:0.015:0.071	0.022:0.849:0.023:0.107	0.013:0.956:0.008:0.023	0.051:0.885:0.024:0.04
Dinosauria	0.002:0.292:0.317:0.39	0.003:0.277:0.206:0.514	0.006:0.923:0.016:0.054	0.014:0.881:0.023:0.082	0.014:0.958:0.009:0.019	0.044:0.901:0.023:0.032
Saurischi	0.001:0.271	0.001:0.257	0.004:0.914	0.01:0.877:	0.003:0.962	0.014:0.924

a	:0.341:0.388	:0.237:0.505	:0.028:0.054	0.033:0.08	:0.015:0.019	:0.03:0.032
Theropoda	0.017:0.011:0.015:0.957	0.015:0.01:0.013:0.962	0.04:0.035:0.016:0.909	0.059:0.041:0.019:0.882	0.059:0.057:0.015:0.868	0.088:0.061:0.021:0.83

Table S40g. Continuation of Table S40f.

Character	Reproduction mode + egg shell mineralisation			
Model	equal-ER (ind.hom)	equal-ER (ind.het)	equal-ER (sw.hom)	equal-ER (sw.het)
Mean log marginal likelihood	-71.581	-70.971	-73.017	-72.192
Mean log Bayes Factor	2.872	4.093	0	1.650
Amniota	0.964:0.033:0.001:0.002	0.958:0.038:0.002:0.002	0.895:0.098:0.003:0.004	0.9:0.089:0.004:0.006
Mammalia	0.728:0.146:0.063:0.063	0.724:0.15:0.063:0.063	0.721:0.144:0.068:0.068	0.714:0.147:0.069:0.069
Reptilia	0.956:0.041:0.001:0.002	0.953:0.043:0.002:0.002	0.926:0.068:0.003:0.003	0.926:0.066:0.004:0.004
Diapsida s.l./s.s.	0.645:0.023:0.149:0.182	0.716:0.019:0.132:0.133	0.512:0.031:0.211:0.246	0.631:0.023:0.159:0.186
Lepidosauria	0:0.035:0.911:0.055	0:0.037:0.912:0.05	0:0.044:0.888:0.068	0:0.048:0.888:0.064
Marine reptiles + Archelosauria	0.973:0.003:0.002:0.022	0.968:0.003:0.003:0.026	0.957:0.006:0.005:0.033	0.953:0.006:0.005:0.037
Archelosauria	0.318:0.05:0.026:0.606	0.436:0.034:0.02:0.51	0.241:0.071:0.052:0.636	0.401:0.049:0.038:0.511
Archosauromorpha	0.893:0.042:0.017:0.048	0.885:0.034:0.016:0.065	0.829:0.067:0.038:0.066	0.83:0.053:0.031:0.086
Archosauria	0.002:0.415:0.042:0.541	0.005:0.31:0.046:0.639	0.002:0.462:0.053:0.482	0.006:0.348:0.057:0.589
Dinosauria	0.001:0.641:0.062:0.296	0.002:0.526:0.069:0.403	0.001:0.66:0.072:0.267	0.003:0.544:0.079:0.375
Saurischia	0.001:0.593:0.147:0.26	0.001:0.492:0.151:0.356	0.001:0.611:0.153:0.236	0.001:0.509:0.158:0.331
Theropoda	0.011:0.012:0.012:0.966	0.01:0.01:0.01:0.969	0.018:0.015:0.015:0.952	0.015:0.013:0.013:0.96

Table S41a. Same as Table S28 but adding extinct marine reptiles as sister taxon to Archosauromorpha (equal dating method).

Character	Reproduction mode + egg shell mineralisation					
Model	equal-CER (ind.hom)	equal-CER (ind.het)	equal-CER (sw.hom)	equal-CER (sw.het)	equal-CSYM (ind.hom)	equal-CSYM (ind.het)
Mean log marginal likelihood	-69.467	-68.981	-71.125	-70.445	-70.742	-70.289
Mean log Bayes Factor	3.639	4.611	0.322	1.683	1.088	1.993
Amniota	0.919:0.076:0.002:0.003	0.917:0.075:0.003:0.005	0.847:0.143:0.005:0.005	0.847:0.139:0.006:0.007	0.94:0.055:0.002:0.003	0.936:0.055:0.003:0.005
Mammalia	0.726:0.143:0.065:0.065	0.72:0.149:0.066:0.066	0.721:0.143:0.068:0.068	0.712:0.147:0.07:0.07	0.723:0.165:0.053:0.059	0.719:0.167:0.053:0.061
Reptilia	0.899:0.093:0.003:0.005	0.907:0.082:0.004:0.007	0.892:0.098:0.004:0.005	0.885:0.102:0.006:0.007	0.929:0.064:0.003:0.005	0.933:0.058:0.003:0.006
Diapsida s.l./s.s.	0.435:0.041:0.185:0.339	0.534:0.029:0.163:0.274	0.418:0.037:0.252:0.293	0.506:0.029:0.199:0.265	0.456:0.029:0.198:0.317	0.549:0.022:0.164:0.265
Lepidosauria	0.059:0.037:0.845:0.059	0.081:0.042:0.822:0.056	0.149:0.035:0.762:0.054	0.179:0.039:0.73:0.051	0.069:0.03:0.84:0.061	0.092:0.034:0.819:0.055
Archelosauria	0.861:0.017:0.009:0.114	0.847:0.014:0.01:0.128	0.853:0.018:0.017:0.112	0.839:0.016:0.017:0.127	0.862:0.011:0.017:0.11	0.851:0.01:0.014:0.125
Marine reptiles + Archosauromorpha	0.995:0.002:0.001:0.002	0.993:0.003:0.001:0.003	0.993:0.003:0.002:0.003	0.988:0.004:0.003:0.005	0.994:0.002:0.002:0.002	0.993:0.002:0.002:0.004
Archosauromorpha	0.728:0.111:0.046:0.116	0.766:0.076:0.036:0.122	0.661:0.128:0.078:0.133	0.71:0.091:0.062:0.138	0.727:0.068:0.071:0.134	0.758:0.05:0.051:0.14
Archosauria	0.002:0.45:0.044:0.504	0.005:0.345:0.049:0.601	0.003:0.461:0.054:0.482	0.006:0.361:0.063:0.57	0.003:0.26:0.169:0.569	0.005:0.205:0.129:0.66
Dinosauri	0.001:0.655	0.002:0.542	0.002:0.654	0.003:0.543	0.002:0.397	0.003:0.341

a	:0.064:0.28	:0.071:0.385	:0.073:0.271	:0.086:0.368	:0.181:0.42	:0.144:0.512
Saurischia	0.001:0.604:0.145:0.25	0.002:0.508:0.15:0.341	0.001:0.606:0.153:0.24	0.002:0.505:0.166:0.327	0.001:0.368:0.222:0.409	0.002:0.318:0.187:0.493
Theropoda	0.012:0.013:0.013:0.962	0.011:0.012:0.012:0.966	0.018:0.016:0.016:0.95	0.016:0.014:0.014:0.956	0.014:0.012:0.014:0.961	0.012:0.01:0.012:0.966

Table S41b. Continuation of Table S41a.

Character	Reproduction mode + egg shell mineralisation					
Model	equal-CSYM (sw.hom)	equal-CSYM (sw.het)	equal-CARD (ind.hom)	equal-CARD (ind.het)	equal-CARD (sw.hom)	equal-CARD (sw.het)
Mean log marginal likelihood	-71.603	-71.132	-62.337	-61.680	-63.579	-62.670
Mean log Bayes Factor	-0.634	0.309	17.898	19.212	15.415	17.233
Amniota	0.908:0.082:0.006:0.004	0.894:0.091:0.007:0.007	1:0:0:0	1:0:0:0	1:0:0:0	1:0:0:0
Mammalia	0.714:0.175:0.066:0.045	0.709:0.172:0.063:0.056	1:0:0:0	0.999:0:0:0	0.999:0:0:0	1:0:0:0
Reptilia	0.931:0.061:0.005:0.004	0.921:0.066:0.006:0.007	1:0:0:0	1:0:0:0	0.999:0.001:0:0	1:0:0:0
Diapsida s.l./s.s.	0.494:0.028:0.285:0.193	0.555:0.025:0.21:0.21	1:0:0:0	1:0:0:0	0.999:0.001:0:0	1:0:0:0
Lepidosauria	0.177:0.035:0.714:0.075	0.191:0.037:0.713:0.059	0.999:0:0.001:0	0.998:0:0.001:0.001	0.998:0:0.001:0	0.998:0:0.001:0
Archelosauria	0.829:0.01:0.077:0.084	0.84:0.011:0.043:0.107	1:0:0:0	1:0:0:0	1:0:0:0	1:0:0:0
Marine reptiles + Archosau	0.993:0.001:0.004:0.002	0.99:0.002:0.004:0.004	1:0:0:0	1:0:0:0	1:0:0:0	1:0:0:0

romorpha						
Archosauromorpha	0.693:0.054:0.155:0.098	0.728:0.048:0.103:0.122	1:0:0:0	1:0:0:0	0.999:0:0:0	1:0:0:0
Archosauria	0.003:0.166:0.473:0.358	0.006:0.175:0.315:0.504	0.012:0.95:0.029:0.009	0.024:0.922:0.029:0.025	0.01:0.738:0.245:0.006	0.096:0.601:0.291:0.012
Dinosauria	0.002:0.264:0.487:0.247	0.003:0.285:0.333:0.379	0.008:0.957:0.028:0.007	0.015:0.94:0.027:0.018	0.011:0.75:0.234:0.004	0.089:0.633:0.27:0.008
Saurischia	0.001:0.253:0.503:0.243	0.002:0.27:0.36:0.369	0.005:0.955:0.034:0.007	0.012:0.945:0.027:0.016	0.003:0.715:0.277:0.004	0.04:0.665:0.287:0.009
Theropoda	0.021:0.008:0.017:0.953	0.019:0.01:0.015:0.956	0.054:0.056:0.048:0.841	0.062:0.053:0.044:0.842	0.059:0.056:0.053:0.831	0.155:0.097:0.082:0.667

Table S41c. Continuation of Table S41b.

Character	Reproduction mode + egg shell mineralisation			
Model	equal-ER (ind.hom)	equal-ER (ind.het)	equal-ER (sw.hom)	equal-ER (sw.het)
Mean log marginal likelihood	-70.311	-69.824	-71.286	-70.698
Mean log Bayes Factor	1.950	2.925	0	1.177
Amniota	0.919:0.076:0.002:0.003	0.917:0.074:0.003:0.006	0.849:0.141:0.005:0.005	0.851:0.134:0.007:0.008
Mammalia	0.725:0.143:0.066:0.066	0.714:0.148:0.069:0.069	0.717:0.142:0.071:0.071	0.707:0.147:0.073:0.073
Reptilia	0.899:0.094:0.003:0.005	0.908:0.082:0.004:0.007	0.891:0.1:0.004:0.005	0.888:0.098:0.006:0.008
Diapsida s.l./s.s.	0.443:0.042:0.183:0.332	0.542:0.03:0.156:0.272	0.446:0.041:0.24:0.274	0.528:0.03:0.187:0.256
Lepidosauria	0.063:0.039:0.837:0.062	0.094:0.047:0.796:0.063	0.168:0.039:0.731:0.062	0.189:0.04:0.717:0.053
Archelosauria	0.861:0.017:0.009:0.113	0.843:0.015:0.011:0.131	0.852:0.022:0.018:0.108	0.843:0.017:0.017:0.123
Marine reptiles + Archosauromo	0.995:0.002:0.001:0.002	0.992:0.003:0.002:0.004	0.992:0.003:0.002:0.003	0.988:0.004:0.003:0.005

rpha				
Archosauromorpha	0.726:0.113:0.047:0.114	0.758:0.079:0.038:0.125	0.655:0.141:0.08:0.125	0.706:0.097:0.062:0.135
Archosauria	0.002:0.458:0.045:0.495	0.005:0.351:0.051:0.592	0.003:0.498:0.057:0.442	0.006:0.385:0.063:0.546
Dinosauria	0.001:0.656:0.065:0.277	0.002:0.548:0.072:0.378	0.002:0.673:0.075:0.25	0.003:0.563:0.087:0.348
Saurischia	0.001:0.606:0.146:0.248	0.002:0.512:0.149:0.338	0.001:0.624:0.151:0.225	0.001:0.527:0.163:0.309
Theropoda	0.013:0.014:0.014:0.96	0.012:0.013:0.013:0.963	0.021:0.018:0.018:0.943	0.018:0.014:0.014:0.954

Table S41d. Continuation of Table S41c.

Character	EER			
Model	equal-EER ER (hom)	equal-EER ER (het)	equal-EER ARD (hom)	equal-EER ARD (het)
Mean log marginal likelihood	-30.684	-30.677	-27.133	-27.221
Mean log Bayes Factor	0	0.014	7.103	6.925
Amniota	0.002:0.998	0.003:0.997	0:1	0:1
Mammalia	0.27:0.73	0.275:0.725	0.016:0.984	0.022:0.978
Reptilia	0:1	0:1	0:1	0:1
Diapsida s.l./s.s.	0.027:0.973	0.042:0.958	0.002:0.998	0.004:0.996
Lepidosauria	0.083:0.917	0.116:0.884	0.005:0.995	0.009:0.991
Archelosauria	0.139:0.861	0.14:0.86	0.008:0.992	0.012:0.988
Marine reptiles + Archosauromorpha	0.004:0.996	0.006:0.994	0:1	0.001:0.999
Archosauromorpha	0.259:0.741	0.235:0.765	0.015:0.985	0.021:0.979
Archosauria	0.998:0.002	0.997:0.003	0.997:0.003	0.985:0.015
Dinosauria	0.999:0.001	0.999:0.001	0.998:0.002	0.989:0.011
Saurischia	0.999:0.001	0.999:0.001	0.999:0.001	0.992:0.008
Theropoda	0.99:0.01	0.992:0.008	0.983:0.017	0.956:0.044

Table S41e. Continuation of Table S41d. Nodes of Lepidosauria and Squamata fixed to a non-viviparous state.

Character	Reproduction mode + egg shell mineralisation					
Model	equal-CER (ind.hom)	equal-CER (ind.het)	equal-CER (sw.hom)	equal-CER (sw.het)	equal-CSYM (ind.hom)	equal-CSYM (ind.het)
Mean log	-69.91	-69.391	-71.608	-70.88	-71.215	-70.721

marginal likelihood						
Mean log Bayes Factor	3.78	4.818	0.385	1.84	1.171	2.159
Amniota	0.876:0.116:0.003:0.004	0.884:0.107:0.004:0.006	0.757:0.229:0.006:0.007	0.784:0.198:0.007:0.011	0.907:0.085:0.003:0.005	0.903:0.087:0.004:0.007
Mammalia	0.727:0.145:0.064:0.064	0.725:0.152:0.062:0.062	0.724:0.145:0.065:0.065	0.718:0.15:0.066:0.066	0.726:0.166:0.051:0.058	0.721:0.17:0.049:0.06
Reptilia	0.847:0.141:0.004:0.007	0.87:0.117:0.005:0.008	0.817:0.167:0.007:0.009	0.829:0.151:0.008:0.012	0.891:0.098:0.004:0.007	0.897:0.089:0.006:0.008
Diapsida s.l./s.s.	0.312:0.05:0.225:0.412	0.44:0.034:0.198:0.329	0.251:0.047:0.32:0.382	0.384:0.036:0.246:0.334	0.319:0.037:0.249:0.395	0.435:0.028:0.212:0.325
Lepidosauria	0:0.038:0.902:0.06	0:0.036:0.915:0.049	0:0.038:0.905:0.057	0:0.043:0.902:0.055	0:0.03:0.91:0.06	0:0.03:0.921:0.049
Archelosauria	0.86:0.016:0.009:0.116	0.841:0.013:0.009:0.137	0.851:0.016:0.015:0.118	0.832:0.015:0.015:0.137	0.861:0.01:0.016:0.113	0.845:0.01:0.013:0.132
Marine reptiles + Archosauromorpha	0.995:0.002:0.001:0.002	0.992:0.003:0.001:0.004	0.993:0.003:0.002:0.003	0.988:0.004:0.003:0.005	0.995:0.001:0.001:0.002	0.993:0.002:0.002:0.004
Archosauromorpha	0.73:0.107:0.045:0.118	0.762:0.072:0.035:0.13	0.661:0.125:0.074:0.14	0.713:0.087:0.055:0.145	0.731:0.065:0.067:0.138	0.763:0.049:0.047:0.141
Archosauria	0.002:0.437:0.042:0.519	0.004:0.327:0.046:0.624	0.002:0.441:0.047:0.51	0.005:0.344:0.052:0.599	0.002:0.25:0.157:0.591	0.004:0.21:0.111:0.674
Dinosauria	0.001:0.65:0.062:0.287	0.002:0.536:0.069:0.393	0.001:0.649:0.067:0.282	0.002:0.543:0.075:0.38	0.001:0.394:0.171:0.433	0.002:0.35:0.127:0.52
Saurischia	0.001:0.599:0.143:0.257	0.002:0.5:0.149:0.349	0.001:0.6:0.149:0.25	0.001:0.51:0.153:0.336	0.001:0.364:0.214:0.421	0.002:0.326:0.172:0.5
Theropoda	0.011:0.012:0.012:0.964	0.01:0.011:0.011:0.969	0.015:0.014:0.014:0.957	0.014:0.012:0.012:0.963	0.012:0.011:0.013:0.965	0.011:0.01:0.011:0.968

Table S41f. Continuation of Table S41e.

Character	Reproduction mode + egg shell mineralisation					
Model	equal-CSYM (sw.hom)	equal-CSYM (sw.het)	equal-CARD (ind.hom)	equal-CARD (ind.het)	equal-CARD (sw.hom)	equal-CARD (sw.het)
Mean log marginal likelihood	-72.304	-71.67	-69.814	-69.56	-69.624	-69.264
Mean log Bayes Factor	-1.007	0.26	3.972	4.48	4.354	5.073
Amniota	0.825:0.16:0.008:0.007	0.831:0.148:0.009:0.012	0.666:0.334:0:0	0.762:0.237:0.001:0.001	0.78:0.22:0:0	0.849:0.15:0:0.001
Mammalia	0.721:0.171:0.056:0.052	0.718:0.171:0.053:0.058	0.721:0.251:0.014:0.014	0.755:0.214:0.015:0.016	0.866:0.112:0.012:0.01	0.878:0.1:0.011:0.011
Reptilia	0.864:0.12:0.009:0.008	0.868:0.111:0.01:0.012	0.559:0.44:0:0.001	0.688:0.31:0.001:0.001	0.587:0.412:0.001:0	0.721:0.278:0.001:0.001
Diapsida s.l./s.s.	0.259:0.047:0.373:0.322	0.388:0.036:0.265:0.311	0.509:0.427:0.03:0.034	0.626:0.289:0.039:0.045	0.542:0.413:0.031:0.014	0.673:0.273:0.032:0.022
Lepidosauria	0:0.037:0.894:0.068	0:0.037:0.906:0.057	0:0.106:0.756:0.138	0:0.13:0.732:0.138	0:0.138:0.768:0.094	0:0.153:0.743:0.104
Archelosauria	0.837:0.013:0.051:0.099	0.833:0.012:0.03:0.125	0.846:0.141:0.003:0.01	0.864:0.112:0.004:0.02	0.872:0.119:0.004:0.005	0.891:0.095:0.004:0.01
Marine reptiles + Archosauromorpha	0.993:0.002:0.003:0.003	0.989:0.002:0.003:0.005	0.933:0.065:0.001:0.001	0.954:0.043:0.002:0.002	0.979:0.02:0:0	0.985:0.013:0.001:0.001
Archosauromorpha	0.679:0.066:0.113:0.141	0.718:0.052:0.076:0.155	0.74:0.245:0.006:0.009	0.79:0.185:0.008:0.017	0.791:0.197:0.007:0.005	0.842:0.142:0.006:0.01
Archosauria	0.003:0.211:0.298:0.489	0.005:0.182:0.193:0.619	0.007:0.944:0.01:0.039	0.015:0.892:0.016:0.077	0.01:0.962:0.01:0.018	0.029:0.916:0.017:0.039
Dinosauri	0.002:0.327	0.003:0.305	0.004:0.954	0.009:0.917	0.011:0.964	0.026:0.928

a	:0.313:0.359	:0.21:0.482	:0.011:0.03	:0.016:0.058	:0.01:0.014	:0.017:0.03
Saurischia	0.001:0.308:0.341:0.351	0.001:0.287:0.246:0.467	0.003:0.948:0.019:0.03	0.007:0.909:0.026:0.058	0.003:0.966:0.017:0.014	0.008:0.938:0.024:0.031
Theropoda	0.017:0.011:0.014:0.957	0.015:0.01:0.013:0.962	0.032:0.032:0.017:0.92	0.041:0.033:0.016:0.91	0.051:0.049:0.016:0.885	0.063:0.049:0.016:0.872

Table S41g. Continuation of Table S41f.

Character	Reproduction mode + egg shell mineralisation			
Model	equal-ER (ind.hom)	equal-ER (ind.het)	equal-ER (sw.hom)	equal-ER (sw.het)
Mean log marginal likelihood	-70.765	-70.256	-71.8	-71.172
Mean log Bayes Factor	2.070	3.089	0	1.257
Amniota	0.875:0.117:0.003:0.004	0.879:0.11:0.005:0.007	0.753:0.233:0.006:0.007	0.777:0.204:0.008:0.011
Mammalia	0.726:0.144:0.065:0.065	0.72:0.15:0.065:0.065	0.722:0.144:0.067:0.067	0.71:0.151:0.07:0.07
Reptilia	0.846:0.143:0.004:0.007	0.863:0.122:0.006:0.009	0.81:0.175:0.007:0.009	0.823:0.156:0.009:0.012
Diapsida s.l./s.s.	0.317:0.052:0.224:0.407	0.432:0.037:0.201:0.329	0.262:0.053:0.313:0.372	0.382:0.039:0.244:0.335
Lepidosauria	0:0.039:0.898:0.063	0:0.04:0.905:0.054	0:0.042:0.892:0.066	0:0.045:0.895:0.06
Archelosauria	0.86:0.016:0.009:0.115	0.843:0.014:0.01:0.132	0.85:0.019:0.016:0.115	0.827:0.017:0.016:0.14
Marine reptiles + Archosauromorpha	0.995:0.002:0.001:0.002	0.992:0.003:0.001:0.004	0.993:0.003:0.002:0.003	0.987:0.004:0.003:0.006
Archosauromorpha	0.727:0.11:0.046:0.117	0.761:0.077:0.036:0.126	0.656:0.134:0.075:0.135	0.705:0.095:0.056:0.144
Archosauria	0.002:0.446:0.042:0.509	0.004:0.34:0.046:0.611	0.002:0.469:0.048:0.48	0.005:0.366:0.053:0.576
Dinosauria	0.001:0.654:0.063:0.282	0.002:0.544:0.068:0.386	0.001:0.664:0.068:0.266	0.003:0.557:0.076:0.364
Saurischia	0.001:0.604:0.143	0.001:0.507:0.148	0.001:0.614:0.146	0.001:0.521:0.152

	:0.252	:0.344	:0.239	:0.326
Theropoda	0.012:0.013:0.013:0.963	0.011:0.011:0.011:0.967	0.017:0.015:0.015:0.953	0.015:0.012:0.012:0.961

Table S42a. Same as Table S28 but adding extinct marine reptiles as sister taxon to Lepidosauria (equal dating method).

Character	Reproduction mode + egg shell mineralisation					
Model	equal-CER (ind.hom)	equal-CER (ind.het)	equal-CER (sw.hom)	equal-CER (sw.het)	equal-CSYM (ind.hom)	equal-CSYM (ind.het)
Mean log marginal likelihood	-69.719	-69.176	-71.064	-70.384	-70.919	-70.417
Mean log Bayes Factor	3.618	4.703	0.926	2.286	1.217	2.220
Amniota	0.964:0.033:0.001:0.002	0.962:0.034:0.002:0.003	0.911:0.083:0.004:0.002	0.918:0.073:0.005:0.004	0.974:0.022:0.001:0.002	0.97:0.025:0.002:0.003
Mammalia	0.726:0.144:0.065:0.065	0.725:0.146:0.065:0.065	0.72:0.143:0.069:0.069	0.715:0.144:0.07:0.07	0.723:0.17:0.05:0.057	0.721:0.168:0.051:0.061
Reptilia	0.956:0.041:0.001:0.002	0.957:0.039:0.001:0.003	0.94:0.055:0.002:0.002	0.941:0.053:0.003:0.003	0.971:0.026:0.001:0.002	0.97:0.026:0.001:0.002
Diapsida s.l./s.s.	0.661:0.024:0.123:0.192	0.739:0.017:0.09:0.154	0.59:0.027:0.19:0.193	0.695:0.02:0.134:0.151	0.675:0.014:0.134:0.177	0.744:0.011:0.097:0.148
Marine reptiles + Lepidosauria	0.89:0.009:0.09:0.011	0.896:0.01:0.084:0.011	0.929:0.006:0.057:0.007	0.922:0.008:0.063:0.008	0.892:0.006:0.09:0.011	0.899:0.007:0.083:0.01
Lepidosauria	0.058:0.036:0.849:0.057	0.078:0.042:0.824:0.056	0.151:0.035:0.759:0.055	0.175:0.038:0.738:0.049	0.067:0.027:0.847:0.059	0.084:0.03:0.835:0.051
Archelosauria	0.205:0.087:0.048:0.661	0.307:0.066:0.042:0.585	0.176:0.106:0.079:0.64	0.313:0.082:0.073:0.532	0.214:0.05:0.106:0.629	0.307:0.044:0.071:0.578
Archosauromorpha	0.733:0.105:0.046:0.115	0.749:0.078:0.038:0.134	0.665:0.13:0.079:0.126	0.702:0.097:0.065:0.136	0.732:0.056:0.074:0.138	0.744:0.047:0.056:0.154
Archosau	0.002:0.436	0.004:0.337	0.003:0.474	0.006:0.375	0.003:0.213	0.005:0.184

ria	:0.046:0.516	:0.049:0.61	:0.057:0.467	:0.065:0.554	:0.191:0.594	:0.14:0.671
Dinosauria	0.001:0.649:0.065:0.285	0.002:0.534:0.072:0.391	0.002:0.662:0.076:0.261	0.003:0.557:0.088:0.352	0.002:0.341:0.202:0.455	0.002:0.318:0.151:0.529
Saurischia	0.001:0.6:0.148:0.251	0.002:0.502:0.152:0.344	0.001:0.608:0.157:0.234	0.001:0.519:0.167:0.313	0.001:0.315:0.237:0.447	0.002:0.299:0.189:0.511
Theropoda	0.012:0.013:0.013:0.962	0.011:0.011:0.011:0.966	0.018:0.016:0.016:0.95	0.016:0.013:0.013:0.957	0.013:0.011:0.014:0.962	0.012:0.01:0.012:0.966

Table S42b. Continuation of Table S42a.

Character	Reproduction mode + egg shell mineralisation					
Model	equal-CSYM (sw.hom)	equal-CSYM (sw.het)	equal-CARD (ind.hom)	equal-CARD (ind.het)	equal-CARD (sw.hom)	equal-CARD (sw.het)
Mean log marginal likelihood	-71.535	-71.065	-62.632	-61.844	-63.718	-62.398
Mean log Bayes Factor	-0.015	0.925	17.791	19.366	15.618	18.258
Amniota	0.951:0.043:0.005:0.002	0.939:0.052:0.005:0.004	1:0:0:0	1:0:0:0	1:0:0:0	1:0:0:0
Mammalia	0.711:0.177:0.069:0.043	0.716:0.168:0.063:0.053	1:0:0:0	1:0:0:0	1:0:0:0	1:0:0:0
Reptilia	0.966:0.03:0.002:0.001	0.955:0.039:0.003:0.003	1:0:0:0	1:0:0:0	1:0:0:0	1:0:0:0
Diapsida s.l./s.s.	0.681:0.015:0.187:0.117	0.72:0.015:0.144:0.121	1:0:0:0	1:0:0:0	1:0:0:0	1:0:0:0
Marine reptiles + Lepidosauria	0.934:0.006:0.051:0.009	0.92:0.007:0.063:0.009	1:0:0:0	1:0:0:0	1:0:0:0	1:0:0:0
Lepidosa	0.192:0.035	0.192:0.035	0.999:0:0.0	0.999:0:0.0	0.998:0.001	0.998:0:0.0

uria	:0.693:0.08	:0.714:0.059	01:0	01:0	:0.001:0.001	01:0
Archelosauria	0.177:0.042:0.307:0.474	0.314:0.045:0.18:0.462	1:0:0:0	1:0:0:0	0.999:0.001:0:0	0.999:0.001:0:0
Archosauromorpha	0.699:0.049:0.168:0.084	0.71:0.05:0.119:0.122	1:0:0:0	1:0:0:0	1:0:0:0	1:0:0:0
Archosauria	0.003:0.152:0.531:0.314	0.007:0.163:0.354:0.476	0.011:0.943:0.034:0.011	0.031:0.894:0.049:0.026	0.01:0.737:0.245:0.007	0.11:0.584:0.299:0.008
Dinosauria	0.002:0.247:0.541:0.21	0.004:0.274:0.371:0.352	0.008:0.951:0.033:0.008	0.02:0.916:0.045:0.019	0.011:0.751:0.233:0.005	0.105:0.616:0.274:0.006
Saurischia	0.001:0.237:0.552:0.21	0.002:0.258:0.398:0.342	0.005:0.948:0.04:0.007	0.016:0.925:0.042:0.018	0.003:0.715:0.277:0.005	0.047:0.665:0.283:0.006
Theropoda	0.023:0.007:0.018:0.952	0.02:0.008:0.016:0.955	0.054:0.056:0.047:0.843	0.075:0.058:0.043:0.824	0.058:0.055:0.053:0.834	0.171:0.103:0.08:0.647

Table S42c. Continuation of Table S42b.

Character	Reproduction mode + egg shell mineralisation			
Model	equal-ER (ind.hom)	equal-ER (ind.het)	equal-ER (sw.hom)	equal-ER (sw.het)
Mean log marginal likelihood	-70.524	-69.997	-71.527	-70.908
Mean log Bayes Factor	2.006	3.060	0	1.239
Amniota	0.963:0.034:0.001:0.002	0.961:0.034:0.002:0.003	0.914:0.08:0.004:0.002	0.922:0.069:0.005:0.004
Mammalia	0.725:0.143:0.066:0.066	0.719:0.147:0.067:0.067	0.717:0.142:0.071:0.071	0.712:0.143:0.073:0.073
Reptilia	0.955:0.042:0.001:0.002	0.957:0.04:0.001:0.002	0.941:0.054:0.002:0.002	0.942:0.052:0.003:0.004
Diapsida s.l./s.s.	0.665:0.025:0.122:0.188	0.74:0.018:0.092:0.151	0.61:0.028:0.182:0.181	0.707:0.02:0.13:0.144
Marine reptiles + Lepidosauria	0.891:0.01:0.088:0.011	0.897:0.01:0.083:0.011	0.933:0.006:0.053:0.008	0.924:0.007:0.061:0.008

Lepidosauria	0.061:0.038:0.84:0.061	0.084:0.044:0.814:0.058	0.169:0.039:0.73:0.062	0.192:0.04:0.714:0.054
Archelosauria	0.206:0.092:0.05:0.651	0.309:0.07:0.044:0.578	0.181:0.117:0.086:0.616	0.314:0.088:0.076:0.522
Archosauromorpha	0.729:0.11:0.048:0.114	0.745:0.082:0.04:0.132	0.659:0.137:0.081:0.122	0.69:0.101:0.068:0.141
Archosauria	0.002:0.451:0.047:0.5	0.005:0.346:0.053:0.597	0.003:0.494:0.061:0.443	0.007:0.382:0.07:0.541
Dinosauria	0.001:0.659:0.065:0.275	0.002:0.541:0.074:0.383	0.002:0.672:0.078:0.249	0.003:0.562:0.089:0.345
Saurischia	0.001:0.61:0.146:0.243	0.002:0.51:0.153:0.335	0.001:0.623:0.155:0.221	0.002:0.524:0.166:0.309
Theropoda	0.012:0.013:0.013:0.961	0.011:0.012:0.012:0.965	0.021:0.018:0.018:0.944	0.018:0.015:0.015:0.952

Table S42d. Continuation of Table S42c.

Character	EER			
Model	equal-EER ER (hom)	equal-EER ER (het)	equal-EER ARD (hom)	equal-EER ARD (het)
Mean log marginal likelihood	-30.666	-30.695	-27.206	-27.105
Mean log Bayes Factor	0	-0.058	6.920	7.122
Amniota	0.002:0.998	0.003:0.997	0:1	0:1
Mammalia	0.27:0.73	0.275:0.725	0.021:0.979	0.017:0.983
Reptilia	0:1	0.001:0.999	0:1	0:1
Diapsida s.l./s.s.	0.053:0.947	0.061:0.939	0.004:0.996	0.004:0.996
Marine reptiles + Lepidosauria	0.002:0.998	0.004:0.996	0:1	0:1
Lepidosauria	0.083:0.917	0.109:0.891	0.006:0.994	0.006:0.994
Archelosauria	0.808:0.192	0.707:0.293	0.063:0.937	0.043:0.957
Archosauromorpha	0.256:0.744	0.249:0.751	0.02:0.98	0.016:0.984
Archosauria	0.998:0.002	0.997:0.003	0.997:0.003	0.979:0.021
Dinosauria	0.999:0.001	0.999:0.001	0.998:0.002	0.985:0.015
Saurischia	0.999:0.001	0.999:0.001	0.999:0.001	0.989:0.011
Theropoda	0.99:0.01	0.991:0.009	0.983:0.017	0.944:0.056

Table S42e. Continuation of Table S42d. Nodes of Lepidosauria and Squamata fixed to a non-viviparous state.

Character	Reproduction mode + egg shell mineralisation					
Model	equal-CER	equal-CER	equal-CER	equal-CER	equal-	equal-

	(ind.hom)	(ind.het)	(sw.hom)	(sw.het)	CSYM (ind.hom)	CSYM (ind.het)
Mean log marginal likelihood	-70.277	-69.681	-71.712	-70.945	-71.510	-70.938
Mean log Bayes Factor	3.889	5.081	1.019	2.554	1.423	2.567
Amniota	0.943:0.053:0.001:0.002	0.946:0.049:0.002:0.003	0.841:0.151:0.004:0.004	0.873:0.117:0.004:0.006	0.96:0.037:0.002:0.002	0.958:0.037:0.002:0.003
Mammalia	0.728:0.145:0.064:0.064	0.724:0.15:0.063:0.063	0.724:0.145:0.066:0.066	0.717:0.148:0.068:0.068	0.726:0.171:0.047:0.056	0.726:0.171:0.046:0.058
Reptilia	0.929:0.066:0.002:0.003	0.94:0.055:0.002:0.003	0.884:0.108:0.004:0.004	0.903:0.088:0.004:0.005	0.954:0.042:0.002:0.003	0.956:0.039:0.002:0.003
Diapsida s.l./s.s.	0.532:0.033:0.17:0.265	0.661:0.022:0.122:0.196	0.401:0.043:0.255:0.301	0.577:0.029:0.171:0.223	0.539:0.021:0.189:0.251	0.668:0.015:0.128:0.189
Marine reptiles + Lepidosauria	0.82:0.016:0.146:0.019	0.843:0.014:0.128:0.015	0.819:0.017:0.145:0.02	0.835:0.016:0.131:0.018	0.819:0.011:0.151:0.019	0.841:0.01:0.134:0.015
Lepidosauria	0:0.036:0.908:0.056	0:0.036:0.915:0.049	0:0.038:0.903:0.058	0:0.043:0.9:0.057	0:0.026:0.919:0.055	0:0.025:0.934:0.042
Archelosauria	0.199:0.081:0.044:0.676	0.297:0.061:0.039:0.602	0.163:0.095:0.069:0.673	0.286:0.075:0.06:0.58	0.205:0.047:0.097:0.651	0.306:0.039:0.063:0.593
Archosauromorpha	0.735:0.102:0.045:0.118	0.75:0.075:0.037:0.138	0.664:0.127:0.074:0.135	0.686:0.097:0.059:0.158	0.736:0.054:0.069:0.141	0.755:0.045:0.049:0.151
Archosauria	0.002:0.425:0.042:0.53	0.004:0.32:0.047:0.629	0.002:0.454:0.049:0.496	0.005:0.352:0.055:0.588	0.002:0.208:0.174:0.616	0.004:0.173:0.124:0.698
Dinosauria	0.001:0.648:0.062:0.29	0.002:0.533:0.071:0.394	0.001:0.658:0.069:0.272	0.002:0.546:0.078:0.374	0.001:0.343:0.187:0.469	0.002:0.309:0.137:0.552
Saurischia	0.001:0.599:0.145:0.255	0.001:0.503:0.15:0.346	0.001:0.605:0.15:0.244	0.001:0.508:0.158:0.333	0.001:0.316:0.224:0.459	0.001:0.288:0.177:0.534

Theropoda	0.011:0.012:0.012:0.965	0.01:0.011:0.011:0.969	0.016:0.014:0.014:0.957	0.014:0.012:0.012:0.963	0.012:0.01:0.012:0.966	0.011:0.009:0.011:0.969

Table S42f. Continuation of Table S42e.

Character	Reproduction mode + egg shell mineralisation					
Model	equal-CSYM (sw.hom)	equal-CSYM (sw.het)	equal-CARD (ind.hom)	equal-CARD (ind.het)	equal-CARD (sw.hom)	equal-CARD (sw.het)
Mean log marginal likelihood	-72.433	-71.740	-70.389	-69.980	-70.019	-69.518
Mean log Bayes Factor	-0.423	0.962	3.665	4.482	4.406	5.407
Amniota	0.893:0.098:0.005:0.004	0.907:0.082:0.005:0.006	0.757:0.242:0.001:0	0.844:0.155:0:0.001	0.842:0.157:0:0	0.903:0.097:0:0
Mammalia	0.72:0.172:0.056:0.052	0.719:0.17:0.053:0.058	0.694:0.27:0.019:0.017	0.749:0.215:0.018:0.019	0.852:0.123:0.013:0.011	0.868:0.106:0.014:0.012
Reptilia	0.917:0.075:0.004:0.004	0.928:0.062:0.004:0.005	0.689:0.31:0.001:0	0.807:0.192:0:0.001	0.708:0.291:0:0	0.834:0.165:0:0
Diapsida s.l./s.s.	0.422:0.035:0.291:0.252	0.593:0.024:0.183:0.2	0.643:0.292:0.035:0.031	0.768:0.174:0.026:0.032	0.67:0.288:0.027:0.015	0.806:0.158:0.02:0.016
Marine reptiles + Lepidosauria	0.818:0.014:0.146:0.022	0.834:0.014:0.132:0.019	0.778:0.031:0.152:0.04	0.842:0.025:0.105:0.028	0.859:0.024:0.094:0.022	0.892:0.021:0.07:0.018
Lepidosauria	0:0.036:0.895:0.069	0:0.035:0.91:0.056	0:0.102:0.758:0.141	0:0.125:0.743:0.131	0:0.145:0.753:0.101	0:0.153:0.743:0.104
Archelosauria	0.165:0.059:0.197:0.58	0.289:0.047:0.12:0.544	0.397:0.512:0.02:0.071	0.539:0.353:0.018:0.09	0.454:0.491:0.019:0.037	0.612:0.327:0.017:0.044
Archosauromorpha	0.682:0.063:0.116:0.139	0.702:0.053:0.083:0.162	0.714:0.261:0.011:0.014	0.776:0.192:0.01:0.023	0.776:0.209:0.008:0.007	0.825:0.156:0.008:0.011
Archosau	0.002:0.204	0.005:0.171	0.006:0.92:	0.015:0.869	0.009:0.956	0.034:0.9:0.

ria	:0.313:0.481	:0.216:0.607	0.019:0.054	:0.022:0.094	:0.014:0.021	023:0.043
Dinosauria	0.002:0.321:0.326:0.351	0.003:0.289:0.235:0.473	0.004:0.935:0.02:0.042	0.009:0.894:0.022:0.074	0.01:0.959:0.014:0.016	0.029:0.914:0.023:0.034
Saurischia	0.001:0.301:0.352:0.347	0.001:0.27:0.267:0.461	0.002:0.925:0.031:0.042	0.007:0.886:0.034:0.073	0.002:0.959:0.022:0.017	0.008:0.925:0.031:0.036
Theropoda	0.017:0.011:0.015:0.957	0.016:0.01:0.013:0.96	0.03:0.03:0.016:0.925	0.044:0.034:0.017:0.905	0.049:0.048:0.016:0.887	0.067:0.051:0.019:0.863

Table S42g. Continuation of Table S42f.

Character	Reproduction mode + egg shell mineralisation			
Model	equal-ER (ind.hom)	equal-ER (ind.het)	equal-ER (sw.hom)	equal-ER (sw.het)
Mean log marginal likelihood	-71.092	-70.504	-72.222	-71.525
Mean log Bayes Factor	2.260	3.435	0	1.394
Amniota	0.943:0.053:0.001:0.002	0.945:0.05:0.002:0.003	0.842:0.15:0.004:0.004	0.867:0.122:0.005:0.006
Mammalia	0.727:0.145:0.064:0.064	0.722:0.149:0.065:0.065	0.721:0.144:0.067:0.067	0.714:0.148:0.069:0.069
Reptilia	0.929:0.066:0.002:0.003	0.938:0.056:0.002:0.004	0.882:0.11:0.004:0.004	0.898:0.092:0.004:0.006
Diapsida s.l./s.s.	0.533:0.033:0.17:0.264	0.663:0.022:0.122:0.193	0.413:0.045:0.25:0.292	0.574:0.03:0.172:0.224
Marine reptiles + Lepidosauria	0.819:0.016:0.146:0.019	0.84:0.015:0.129:0.017	0.819:0.018:0.142:0.021	0.83:0.017:0.135:0.019
Lepidosauria	0:0.036:0.907:0.057	0:0.038:0.911:0.051	0:0.043:0.889:0.067	0:0.043:0.901:0.057
Archelosauria	0.2:0.082:0.045:0.673	0.304:0.064:0.04:0.591	0.168:0.104:0.074:0.654	0.282:0.077:0.06:0.581
Archosauromorpha	0.734:0.103:0.045:0.118	0.747:0.079:0.038:0.136	0.658:0.133:0.077:0.132	0.686:0.099:0.059:0.156
Archosauria	0.002:0.427:0.043:0.528	0.004:0.33:0.048:0.617	0.002:0.471:0.052:0.475	0.005:0.356:0.056:0.584
Dinosauria	0.001:0.647:0.063	0.002:0.536:0.07:	0.001:0.666:0.07:	0.002:0.553:0.078

	:0.289	0.391	0.262	:0.367
Saurischia	0.001:0.598:0.146:0.255	0.002:0.506:0.15:0.343	0.001:0.618:0.149:0.232	0.001:0.517:0.156:0.325
Theropoda	0.011:0.012:0.012:0.965	0.011:0.011:0.011:0.967	0.018:0.015:0.015:0.952	0.015:0.012:0.012:0.96

Table S43a. Same as Table S29 but adding extinct marine reptiles as sister taxon to Archelosauria (mbl dating method).

Character	Reproduction mode + egg shell mineralisation					
Model	mbl-CER (ind.hom)	mbl-CER (ind.het)	mbl-CER (sw.hom)	mbl-CER (sw.het)	mbl-CSYM (ind.hom)	mbl-CSYM (ind.het)
Mean log marginal likelihood	-71.231	-70.268	-71.879	-70.720	-73.156	-72.046
Mean log Bayes Factor	2.448	4.374	1.152	3.471	-1.401	0.817
Amniota	0.916:0.077:0.003:0.003	0.91:0.079:0.006:0.006	0.909:0.084:0.003:0.003	0.902:0.087:0.006:0.006	0.92:0.074:0.003:0.003	0.918:0.071:0.005:0.005
Mammalia	0.984:0.007:0.004:0.004	0.978:0.01:0.006:0.006	0.983:0.008:0.005:0.005	0.975:0.011:0.007:0.007	0.983:0.007:0.005:0.004	0.976:0.011:0.007:0.006
Reptilia	0.911:0.087:0.001:0.001	0.908:0.088:0.002:0.002	0.914:0.083:0.001:0.001	0.908:0.087:0.002:0.003	0.915:0.083:0.001:0.001	0.915:0.08:0.002:0.002
Diapsida s.l./s.s.	0.989:0.006:0.003:0.003	0.984:0.007:0.004:0.005	0.986:0.007:0.003:0.003	0.981:0.008:0.005:0.006	0.989:0.006:0.003:0.003	0.985:0.006:0.004:0.005
Lepidosauria	0.139:0.046:0.167:0.648	0.203:0.066:0.229:0.502	0.116:0.05:0.216:0.617	0.197:0.071:0.255:0.476	0.135:0.049:0.163:0.654	0.185:0.078:0.219:0.518
Marine reptiles + Archelosauria	0.999:0.001:0:0	0.998:0.001:0.001:0.001	0.998:0.001:0:0	0.997:0.001:0.001:0.001	0.999:0.001:0:0	0.998:0.001:0.001:0.001
Archelosauria	0.918:0.052:0.015:0.014	0.914:0.048:0.018:0.02	0.889:0.063:0.027:0.02	0.891:0.057:0.027:0.026	0.915:0.052:0.018:0.015	0.912:0.046:0.021:0.021
Archosauromorpha	0.956:0.03:0.008:0.006	0.955:0.028:0.01:0.008	0.951:0.03:0.013:0.007	0.948:0.029:0.014:0.009	0.957:0.028:0.009:0.006	0.956:0.026:0.01:0.008

Archosauria	0.016:0.849:0.092:0.043	0.031:0.775:0.118:0.076	0.018:0.84:0.091:0.052	0.033:0.762:0.117:0.088	0.016:0.829:0.111:0.044	0.03:0.747:0.141:0.082
Dinosauria	0.01:0.877:0.078:0.035	0.017:0.823:0.099:0.061	0.011:0.873:0.076:0.039	0.019:0.814:0.097:0.069	0.01:0.864:0.091:0.034	0.017:0.803:0.116:0.065
Saurischia	0.006:0.875:0.087:0.032	0.01:0.825:0.107:0.058	0.005:0.869:0.089:0.038	0.009:0.815:0.108:0.068	0.006:0.865:0.098:0.032	0.01:0.808:0.12:0.061
Theropoda	0.001:0.001:0.001:0.997	0.001:0.001:0.001:0.998	0.001:0.001:0.001:0.997	0.001:0.001:0.001:0.997	0.001:0.001:0.002:0.996	0.001:0.001:0.002:0.996

Table S43b. Continuation of Table S43a.

Character	Reproduction mode + egg shell mineralisation					
Model	mbl-CSYM (sw.hom)	mbl-CSYM (sw.het)	mbl-CARD (ind.hom)	mbl-CARD (ind.het)	mbl-CARD (sw.hom)	mbl-CARD (sw.het)
Mean log marginal likelihood	-73.462	-72.120	-68.184	-66.608	-70.461	-68.470
Mean log Bayes Factor	-2.013	0.669	8.542	11.694	3.988	7.969
Amniota	0.92:0.073:0.003:0.003	0.913:0.074:0.006:0.006	0.998:0.002:0:0	0.999:0.001:0:0	0.967:0.032:0:0	0.988:0.011:0:0
Mammalia	0.983:0.008:0.005:0.005	0.977:0.01:0.007:0.007	1:0:0:0	1:0:0:0	0.995:0.004:0.001:0	0.998:0.002:0:0
Reptilia	0.923:0.074:0.001:0.001	0.917:0.077:0.003:0.002	0.998:0.002:0:0	0.999:0.001:0:0	0.941:0.058:0:0	0.979:0.021:0:0
Diapsida s.l./s.s.	0.987:0.006:0.004:0.003	0.982:0.007:0.005:0.005	1:0:0:0	1:0:0:0	0.993:0.006:0:0	0.997:0.003:0:0
Lepidosauria	0.102:0.057:0.203:0.637	0.174:0.095:0.235:0.497	0.986:0.001:0.002:0.011	0.994:0.001:0.001:0.005	0.632:0.023:0.089:0.255	0.878:0.012:0.036:0.074
Marine reptiles +	0.998:0.001:0:0	0.997:0.001:0.001:0.00	1:0:0:0	1:0:0:0	0.999:0.001:0:0	1:0:0:0

Archelosauria		1				
Archelosauria	0.882:0.064:0.032:0.022	0.891:0.052:0.031:0.026	0.999:0.001:0:0	0.999:0:0:0	0.961:0.035:0.003:0.001	0.986:0.013:0.001:0.001
Archosauromorpha	0.952:0.028:0.013:0.007	0.95:0.026:0.014:0.01	0.999:0.001:0:0	1:0:0:0	0.984:0.014:0.001:0	0.994:0.005:0.001:0
Archosauria	0.016:0.815:0.116:0.053	0.027:0.723:0.154:0.096	0.019:0.877:0.095:0.008	0.055:0.814:0.109:0.022	0.046:0.881:0.065:0.008	0.12:0.768:0.093:0.019
Dinosauria	0.01:0.856:0.095:0.039	0.015:0.786:0.125:0.073	0.012:0.906:0.076:0.006	0.037:0.86:0.088:0.015	0.027:0.916:0.051:0.005	0.075:0.837:0.076:0.012
Saurischia	0.004:0.857:0.103:0.036	0.007:0.795:0.129:0.069	0.008:0.91:0.077:0.005	0.025:0.879:0.084:0.012	0.006:0.938:0.053:0.004	0.023:0.893:0.074:0.01
Theropoda	0.001:0.002:0.003:0.994	0.001:0.002:0.003:0.995	0.001:0.002:0.001:0.996	0.003:0.003:0.003:0.99	0.001:0.003:0.003:0.993	0.003:0.004:0.004:0.989

Table S43c. Continuation of Table S43b.

Character	Reproduction mode + egg shell mineralisation			
Model	mbl-ER (ind.hom)	mbl-ER (ind.het)	mbl-ER (sw.hom)	mbl-ER (sw.het)
Mean log marginal likelihood	-71.626	-70.635	-72.455	-71.255
Mean log Bayes Factor	1.659	3.641	0	2.401
Amniota	0.909:0.083:0.003:0.004	0.893:0.092:0.007:0.007	0.9:0.093:0.003:0.004	0.88:0.104:0.008:0.008
Mammalia	0.983:0.008:0.005:0.005	0.974:0.011:0.008:0.008	0.982:0.008:0.005:0.005	0.971:0.012:0.008:0.008
Reptilia	0.906:0.092:0.001:0.001	0.894:0.1:0.003:0.003	0.906:0.091:0.001:0.002	0.89:0.103:0.003:0.003
Diapsida s.l./s.s.	0.988:0.006:0.003:0.003	0.983:0.007:0.005:0.005	0.986:0.007:0.004:0.004	0.98:0.009:0.005:0.006
Lepidosauria	0.157:0.046:0.151:0.645	0.232:0.066:0.197:0.504	0.132:0.05:0.207:0.611	0.235:0.068:0.23:0.466
Marine reptiles +	0.999:0.001:0:0	0.997:0.001:0.001:0.001	0.998:0.001:0:0	0.996:0.002:0.001:0.001

Archelosauria				
Archelosauria	0.922:0.05:0.015:0.014	0.916:0.047:0.017:0.02	0.895:0.06:0.026:0.019	0.898:0.052:0.026:0.024
Archosauromorpha	0.956:0.029:0.008:0.006	0.953:0.029:0.01:0.008	0.951:0.03:0.013:0.007	0.949:0.028:0.014:0.01
Archosauria	0.019:0.843:0.094:0.044	0.037:0.771:0.114:0.078	0.021:0.837:0.09:0.052	0.04:0.754:0.117:0.089
Dinosauria	0.012:0.873:0.08:0.035	0.021:0.818:0.096:0.065	0.014:0.87:0.077:0.039	0.023:0.805:0.099:0.073
Saurischia	0.007:0.874:0.087:0.032	0.012:0.825:0.102:0.061	0.006:0.868:0.089:0.038	0.011:0.807:0.11:0.072
Theropoda	0.001:0.001:0.001:0.997	0.001:0.001:0.001:0.997	0.001:0.001:0.001:0.997	0.001:0.001:0.001:0.997

Table S43d. Continuation of Table S43c.

Character	EER			
Model	mbl-EER ER (hom)	mbl-EER ER (het)	mbl-EER ARD (hom)	mbl-EER ARD (het)
Mean log marginal likelihood	-34.257	-33.209	-33.016	-32.216
Mean log Bayes Factor	0	2.096	2.482	4.082
Amniota	0.006:0.994	0.015:0.985	0.002:0.998	0.006:0.994
Mammalia	0.015:0.985	0.028:0.972	0.007:0.993	0.012:0.988
Reptilia	0.001:0.999	0.006:0.994	0.001:0.999	0.002:0.998
Diapsida s.l./s.s.	0.005:0.995	0.009:0.991	0.002:0.998	0.004:0.996
Lepidosauria	0.174:0.826	0.289:0.711	0.069:0.931	0.148:0.852
Marine reptiles + Archelosauria	0.001:0.999	0.002:0.998	0:1	0.001:0.999
Archelosauria	0.103:0.897	0.108:0.892	0.054:0.946	0.065:0.935
Archosauromorpha	0.042:0.958	0.047:0.953	0.021:0.979	0.027:0.973
Archosauria	0.988:0.012	0.988:0.012	0.994:0.006	0.982:0.018
Dinosauria	0.993:0.007	0.993:0.007	0.996:0.004	0.989:0.011
Saurischia	0.996:0.004	0.996:0.004	0.998:0.002	0.992:0.008
Theropoda	0.999:0.001	0.999:0.001	1:0	0.999:0.001

Table S43e. Continuation of Table S43d. Nodes of Lepidosauria and Squamata fixed to a non-viviparous state.

Character	Reproduction mode + egg shell mineralisation					
Model	mbl-CER (ind.hom)	mbl-CER (ind.het)	mbl-CER (sw.hom)	mbl-CER (sw.het)	mbl-CSYM (ind.hom)	mbl-CSYM (ind.het)

Mean log marginal likelihood	-71.436	-70.559	-71.994	-70.941	-73.343	-72.302
Mean log Bayes Factor	2.299	4.051	1.183	3.289	-1.516	0.567
Amniota	0.919:0.075:0.003:0.003	0.916:0.074:0.005:0.005	0.91:0.084:0.003:0.003	0.902:0.087:0.005:0.006	0.924:0.07:0.003:0.003	0.923:0.066:0.005:0.005
Mammalia	0.984:0.007:0.004:0.004	0.977:0.01:0.006:0.006	0.983:0.008:0.005:0.005	0.975:0.011:0.007:0.007	0.984:0.007:0.005:0.004	0.977:0.01:0.007:0.006
Reptilia	0.913:0.085:0.001:0.001	0.913:0.084:0.002:0.002	0.913:0.085:0.001:0.001	0.906:0.09:0.002:0.002	0.918:0.08:0.001:0.001	0.92:0.076:0.002:0.002
Diapsida s.l./s.s.	0.988:0.006:0.003:0.003	0.983:0.007:0.004:0.005	0.986:0.007:0.004:0.003	0.981:0.009:0.005:0.006	0.988:0.006:0.003:0.003	0.983:0.007:0.005:0.005
Lepidosauria	0:0.054:0.194:0.752	0:0.08:0.285:0.635	0:0.058:0.242:0.7	0:0.09:0.319:0.59	0:0.056:0.185:0.759	0:0.097:0.264:0.639
Marine reptiles + Archelosauria	0.999:0.001:0:0	0.998:0.001:0.001:0.001	0.998:0.001:0:0	0.997:0.001:0.001:0.001	0.999:0.001:0:0	0.998:0.001:0.001:0.001
Archelosauria	0.917:0.053:0.015:0.015	0.91:0.05:0.018:0.022	0.887:0.065:0.028:0.021	0.89:0.058:0.027:0.025	0.912:0.054:0.019:0.016	0.908:0.047:0.023:0.023
Archosauromorpha	0.957:0.03:0.008:0.006	0.955:0.028:0.01:0.007	0.951:0.03:0.013:0.007	0.949:0.029:0.013:0.009	0.957:0.029:0.009:0.006	0.955:0.026:0.011:0.008
Archosauria	0.015:0.853:0.09:0.042	0.028:0.785:0.113:0.074	0.017:0.841:0.091:0.052	0.029:0.771:0.116:0.084	0.015:0.833:0.109:0.043	0.027:0.745:0.144:0.084
Dinosauria	0.009:0.879:0.077:0.034	0.015:0.83:0.094:0.06	0.011:0.874:0.076:0.039	0.016:0.822:0.096:0.066	0.009:0.866:0.091:0.034	0.016:0.802:0.117:0.066
Saurischia	0.006:0.875:0.087:0.032	0.009:0.831:0.103:0.057	0.004:0.869:0.089:0.037	0.008:0.82:0.107:0.066	0.005:0.867:0.097:0.032	0.009:0.808:0.121:0.062
Theropoda	0.001:0.001:0.001:0.99	0.001:0.001:0.001:0.99	0.001:0.001:0.001:0.99	0.001:0.001:0.001:0.99	0.001:0.001:0.002:0.99	0.001:0.001:0.002:0.99

	8	8	7	7	6	6

Table S43f. Continuation of Table S43e.

Character	Reproduction mode + egg shell mineralisation					
Model	mbl-CSYM (sw.hom)	mbl-CSYM (sw.het)	mbl-CARD (ind.hom)	mbl-CARD (ind.het)	mbl-CARD (sw.hom)	mbl-CARD (sw.het)
Mean log marginal likelihood	-73.566	-72.316	-72.638	-71.945	-71.372	-70.552
Mean log Bayes Factor	-1.963	0.537	-0.106	1.280	2.427	4.065
Amniota	0.919:0.075:0.003:0.003	0.918:0.072:0.005:0.005	0.893:0.103:0.003:0.001	0.904:0.09:0.004:0.002	0.925:0.074:0.001:0	0.931:0.067:0.001:0.001
Mammalia	0.983:0.007:0.005:0.005	0.978:0.01:0.007:0.006	0.984:0.01:0.004:0.002	0.98:0.012:0.004:0.003	0.99:0.008:0.001:0.001	0.987:0.011:0.002:0.001
Reptilia	0.92:0.078:0.001:0.001	0.919:0.076:0.003:0.002	0.883:0.115:0.001:0	0.89:0.108:0.001:0.001	0.863:0.136:0:0	0.873:0.126:0:0
Diapsida s.l./s.s.	0.986:0.007:0.004:0.003	0.982:0.008:0.005:0.005	0.988:0.008:0.002:0.001	0.985:0.01:0.003:0.002	0.985:0.014:0.001:0	0.982:0.016:0.001:0.001
Lepidosauria	0:0.066:0.222:0.712	0:0.116:0.286:0.598	0:0.061:0.19:0.749	0:0.089:0.253:0.658	0:0.063:0.242:0.695	0:0.099:0.288:0.613
Marine reptiles + Archelosauria	0.998:0.001:0:0	0.997:0.001:0.001:0.001	0.999:0.001:0:0	0.998:0.001:0:0	0.999:0.001:0:0	0.998:0.002:0:0
Archelosauria	0.882:0.064:0.032:0.022	0.886:0.054:0.032:0.028	0.926:0.059:0.01:0.004	0.924:0.06:0.01:0.007	0.906:0.084:0.007:0.003	0.908:0.082:0.006:0.003
Archosauromorpha	0.952:0.028:0.013:0.007	0.951:0.025:0.014:0.01	0.96:0.032:0.006:0.002	0.96:0.032:0.006:0.002	0.962:0.034:0.003:0.001	0.962:0.034:0.003:0.001
Archosauria	0.016:0.815:0.115:0.054	0.026:0.722:0.153:0.098	0.019:0.906:0.068:0.007	0.033:0.878:0.07:0.019	0.039:0.929:0.027:0.005	0.065:0.895:0.031:0.009
Dinosauri	0.01:0.856:	0.015:0.784	0.012:0.926	0.02:0.907:	0.022:0.952	0.036:0.933

a	0.094:0.04	:0.126:0.076	:0.057:0.005	0.058:0.015	:0.023:0.003	:0.024:0.007
Saurischia	0.004:0.858:0.101:0.037	0.007:0.793:0.129:0.071	0.007:0.926:0.062:0.005	0.012:0.911:0.062:0.014	0.005:0.965:0.027:0.003	0.011:0.954:0.027:0.007
Theropoda	0.001:0.002:0.003:0.994	0.001:0.002:0.003:0.995	0.001:0.001:0.001:0.997	0.001:0.001:0.001:0.996	0.001:0.002:0.001:0.996	0.002:0.002:0.002:0.995

Table S43g. Continuation of Table S43f.

Character	Reproduction mode + egg shell mineralisation			
Model	mbl-ER (ind.hom)	mbl-ER (ind.het)	mbl-ER (sw.hom)	mbl-ER (sw.het)
Mean log marginal likelihood	-71.855	-70.958	-72.585	-71.522
Mean log Bayes Factor	1.460	3.254	0	2.126
Amniota	0.913:0.08:0.003:0.003	0.9:0.087:0.006:0.006	0.9:0.094:0.003:0.004	0.883:0.103:0.006:0.008
Mammalia	0.983:0.008:0.005:0.005	0.974:0.011:0.008:0.008	0.982:0.008:0.005:0.005	0.971:0.012:0.008:0.008
Reptilia	0.909:0.089:0.001:0.001	0.899:0.096:0.003:0.003	0.904:0.094:0.001:0.001	0.891:0.104:0.003:0.003
Diapsida s.l./s.s.	0.988:0.007:0.003:0.003	0.982:0.008:0.005:0.005	0.985:0.007:0.004:0.004	0.979:0.009:0.005:0.006
Lepidosauria	0:0.054:0.184:0.762	0:0.086:0.258:0.656	0:0.058:0.23:0.712	0:0.091:0.304:0.605
Marine reptiles + Archelosauria	0.999:0.001:0:0	0.997:0.001:0.001:0.001	0.998:0.001:0:0	0.996:0.002:0.001:0.001
Archelosauria	0.92:0.051:0.015:0.014	0.916:0.047:0.017:0.02	0.895:0.06:0.026:0.019	0.896:0.054:0.026:0.024
Archosauromorpha	0.956:0.03:0.008:0.006	0.954:0.028:0.01:0.008	0.951:0.03:0.013:0.007	0.948:0.029:0.014:0.009
Archosauria	0.017:0.85:0.091:0.042	0.035:0.778:0.116:0.072	0.02:0.838:0.091:0.051	0.037:0.763:0.113:0.086
Dinosauria	0.011:0.877:0.078:0.034	0.019:0.825:0.097:0.059	0.013:0.873:0.076:0.038	0.02:0.815:0.095:0.07
Saurischia	0.006:0.875:0.087:0.032	0.011:0.828:0.106:0.055	0.005:0.869:0.088:0.037	0.01:0.815:0.107:0.069
Theropoda	0.001:0.001:0.001:0.998	0.001:0.001:0.001:0.998	0.001:0.001:0.001:0.997	0.001:0.001:0.001:0.997

Table S44a. Same as Table S29 but adding extinct marine reptiles as sister taxon to Archosauromorpha (mbl dating method).

Character	Reproduction mode + egg shell mineralisation					
Model	mbl-CER (ind.hom)	mbl-CER (ind.het)	mbl-CER (sw.hom)	mbl-CER (sw.het)	mbl-CSYM (ind.hom)	mbl-CSYM (ind.het)
Mean log marginal likelihood	-71.041	-70.110	-71.674	-70.546	-72.935	-71.868
Mean log Bayes Factor	2.435	4.296	1.168	3.423	-1.355	0.779
Amniota	0.913:0.081:0.003:0.003	0.906:0.083:0.005:0.006	0.902:0.091:0.003:0.003	0.893:0.095:0.006:0.006	0.918:0.076:0.003:0.003	0.914:0.075:0.006:0.005
Mammalia	0.984:0.007:0.004:0.004	0.975:0.011:0.007:0.007	0.983:0.008:0.005:0.005	0.975:0.011:0.007:0.007	0.984:0.007:0.005:0.004	0.976:0.01:0.007:0.006
Reptilia	0.907:0.091:0.001:0.001	0.902:0.094:0.002:0.002	0.907:0.09:0.001:0.001	0.9:0.095:0.003:0.003	0.911:0.086:0.001:0.001	0.91:0.086:0.003:0.002
Diapsida s.l./s.s.	0.974:0.013:0.006:0.007	0.965:0.015:0.009:0.011	0.966:0.017:0.01:0.006	0.956:0.019:0.013:0.011	0.972:0.013:0.007:0.007	0.962:0.015:0.011:0.012
Lepidosauria	0.133:0.046:0.167:0.654	0.198:0.068:0.229:0.505	0.112:0.052:0.217:0.62	0.193:0.072:0.26:0.476	0.127:0.047:0.163:0.663	0.181:0.078:0.219:0.521
Archelosauria	0.989:0.006:0.003:0.003	0.983:0.008:0.004:0.005	0.985:0.008:0.004:0.002	0.979:0.011:0.006:0.005	0.988:0.006:0.003:0.003	0.983:0.007:0.005:0.005
Marine reptiles + Archosauromorpha	0.999:0.001:0:0	0.998:0.001:0.001:0	0.999:0.001:0:0	0.998:0.001:0.001:0.001	0.999:0.001:0:0	0.998:0.001:0.001:0
Archosauromorpha	0.958:0.028:0.008:0.006	0.956:0.027:0.01:0.007	0.952:0.028:0.013:0.007	0.951:0.027:0.013:0.009	0.958:0.027:0.009:0.006	0.957:0.024:0.011:0.008
Archosauria	0.016:0.842:0.099:0.043	0.029:0.778:0.119:0.074	0.017:0.832:0.098:0.053	0.032:0.756:0.124:0.088	0.016:0.821:0.12:0.044	0.029:0.74:0.15:0.081
Dinosauria	0.01:0.877:0.078:0.035	0.016:0.826:0.097:0.06	0.011:0.873:0.077:0.03	0.018:0.815:0.097:0.07	0.01:0.862:0.094:0.033	0.017:0.801:0.119:0.06

			9			3
Saurischia	0.006:0.874:0.088:0.033	0.01:0.827:0.105:0.057	0.005:0.868:0.089:0.038	0.008:0.814:0.109:0.069	0.006:0.861:0.102:0.031	0.01:0.805:0.125:0.06
Theropoda	0.001:0.001:0.001:0.998	0.001:0.001:0.001:0.997	0.001:0.001:0.001:0.997	0.001:0.001:0.001:0.997	0.001:0.001:0.002:0.996	0.001:0.001:0.002:0.996

Table S44b. Continuation of Table S44a.

Character	Reproduction mode + egg shell mineralisation					
Model	mbl-CSYM (sw.hom)	mbl-CSYM (sw.het)	mbl-CARD (ind.hom)	mbl-CARD (ind.het)	mbl-CARD (sw.hom)	mbl-CARD (sw.het)
Mean log marginal likelihood	-73.231	-71.943	-68.080	-66.638	-70.300	-68.507
Mean log Bayes Factor	-1.947	0.629	8.356	11.241	3.915	7.501
Amniota	0.913:0.08:0.004:0.003	0.908:0.08:0.007:0.006	0.998:0.002:0:0	0.999:0.001:0:0	0.966:0.033:0:0	0.986:0.014:0:0
Mammalia	0.983:0.008:0.005:0.005	0.978:0.01:0.007:0.006	1:0:0:0	1:0:0:0	0.996:0.003:0.001:0	0.997:0.002:0:0
Reptilia	0.914:0.083:0.002:0.001	0.911:0.084:0.003:0.003	0.998:0.002:0:0	0.999:0.001:0:0	0.94:0.06:0:0	0.975:0.025:0:0
Diapsida s.l./s.s.	0.964:0.018:0.012:0.006	0.958:0.018:0.013:0.01	1:0:0:0	1:0:0:0	0.983:0.015:0.001:0	0.992:0.007:0.001:0
Lepidosauria	0.098:0.057:0.205:0.64	0.172:0.093:0.235:0.499	0.986:0.001:0.002:0.011	0.993:0.001:0.002:0.005	0.643:0.024:0.088:0.245	0.862:0.014:0.039:0.085
Archelosauria	0.986:0.008:0.004:0.002	0.982:0.009:0.006:0.004	1:0:0:0	1:0:0:0	0.994:0.005:0.001:0	0.997:0.003:0:0
Marine reptiles + Archosauromorpha	0.999:0.001:0:0	0.998:0.001:0.001:0	1:0:0:0	1:0:0:0	1:0:0:0	1:0:0:0
Archosau	0.954:0.026	0.954:0.024	0.999:0:0:0	1:0:0:0	0.984:0.014	0.994:0.006

romorpha	:0.013:0.007	:0.013:0.009			:0.001:0	:0.001:0
Archosauria	0.016:0.806:0.125:0.053	0.027:0.72:0.158:0.095	0.019:0.873:0.101:0.008	0.051:0.81:0.116:0.023	0.04:0.879:0.073:0.008	0.105:0.78:0.097:0.018
Dinosauria	0.01:0.855:0.096:0.038	0.015:0.787:0.125:0.072	0.012:0.905:0.078:0.005	0.033:0.859:0.093:0.015	0.025:0.914:0.056:0.005	0.069:0.841:0.079:0.012
Saurischia	0.004:0.856:0.104:0.036	0.007:0.793:0.131:0.069	0.007:0.908:0.08:0.005	0.022:0.875:0.091:0.012	0.005:0.932:0.058:0.004	0.02:0.892:0.078:0.01
Theropoda	0.001:0.002:0.003:0.994	0.001:0.002:0.003:0.995	0.001:0.001:0.001:0.997	0.002:0.003:0.003:0.992	0.001:0.003:0.002:0.993	0.003:0.004:0.003:0.99

Table S44c. Continuation of Table S44b.

Character	Reproduction mode + egg shell mineralisation			
Model	mbl-ER (ind.hom)	mbl-ER (ind.het)	mbl-ER (sw.hom)	mbl-ER (sw.het)
Mean log marginal likelihood	-71.443	-70.479	-72.258	-71.101
Mean log Bayes Factor	1.630	3.558	0	2.315
Amniota	0.907:0.086:0.003:0.004	0.889:0.097:0.007:0.007	0.893:0.099:0.004:0.004	0.874:0.11:0.008:0.008
Mammalia	0.983:0.008:0.005:0.005	0.973:0.011:0.008:0.008	0.982:0.008:0.005:0.005	0.971:0.012:0.008:0.008
Reptilia	0.902:0.095:0.001:0.001	0.889:0.105:0.003:0.003	0.9:0.098:0.001:0.002	0.884:0.11:0.003:0.003
Diapsida s.l./s.s.	0.974:0.013:0.006:0.007	0.962:0.016:0.01:0.012	0.967:0.017:0.01:0.006	0.956:0.02:0.013:0.011
Lepidosauria	0.147:0.046:0.156:0.651	0.228:0.066:0.201:0.505	0.124:0.05:0.207:0.619	0.233:0.068:0.235:0.464
Archelosauria	0.988:0.006:0.003:0.003	0.981:0.009:0.005:0.006	0.985:0.009:0.004:0.002	0.977:0.012:0.006:0.005
Marine reptiles + Archosauromorpha	0.999:0.001:0:0	0.997:0.001:0.001:0.001	0.999:0.001:0:0	0.997:0.002:0.001:0.001
Archosauromorpha	0.957:0.028:0.008:0.006	0.954:0.028:0.01:0.008	0.952:0.028:0.012:0.007	0.952:0.027:0.013:0.009

Archosauria	0.018:0.84:0.099:0.043	0.035:0.772:0.12:0.074	0.02:0.832:0.096:0.052	0.038:0.757:0.119:0.086
Dinosauria	0.011:0.875:0.079:0.035	0.02:0.822:0.097:0.061	0.013:0.872:0.076:0.039	0.022:0.811:0.097:0.069
Saurischia	0.007:0.874:0.087:0.032	0.012:0.826:0.105:0.058	0.006:0.868:0.089:0.038	0.01:0.813:0.109:0.068
Theropoda	0.001:0.001:0.001:0.998	0.001:0.001:0.001:0.998	0.001:0.001:0.001:0.997	0.001:0.001:0.001:0.997

Table S44d. Continuation of Table S44c.

Character	EER			
Model	mbl-EER ER (hom)	mbl-EER ER (het)	mbl-EER ARD (hom)	mbl-EER ARD (het)
Mean log marginal likelihood	-34.157	-33.093	-32.912	-32.171
Mean log Bayes Factor	0	2.129	2.492	3.972
Amniota	0.006:0.994	0.016:0.984	0.002:0.998	0.006:0.994
Mammalia	0.015:0.985	0.026:0.974	0.007:0.993	0.014:0.986
Reptilia	0.001:0.999	0.006:0.994	0.001:0.999	0.002:0.998
Diapsida s.l./s.s.	0.011:0.989	0.019:0.981	0.005:0.995	0.01:0.99
Lepidosauria	0.177:0.823	0.301:0.699	0.069:0.931	0.152:0.848
Archelosauria	0.011:0.989	0.015:0.985	0.005:0.995	0.009:0.991
Marine reptiles + Archosauromorpha	0.001:0.999	0.001:0.999	0:1	0.001:0.999
Archosauromorpha	0.041:0.959	0.045:0.955	0.02:0.98	0.028:0.972
Archosauria	0.988:0.012	0.988:0.012	0.994:0.006	0.985:0.015
Dinosauria	0.993:0.007	0.993:0.007	0.996:0.004	0.99:0.01
Saurischia	0.996:0.004	0.996:0.004	0.998:0.002	0.994:0.006
Theropoda	0.999:0.001	1:0	1:0	0.999:0.001

Table S44e. Continuation of Table S44d. Nodes of Lepidosauria and Squamata fixed to a non-viviparous state.

Character	Reproduction mode + egg shell mineralisation					
Model	mbl-CER (ind.hom)	mbl-CER (ind.het)	mbl-CER (sw.hom)	mbl-CER (sw.het)	mbl-CSYM (ind.hom)	mbl-CSYM (ind.het)
Mean log marginal likelihood	-71.236	-70.391	-71.788	-70.781	-73.119	-72.124
Mean log	2.293	3.982	1.189	3.202	-1.472	0.517

Bayes Factor						
Amniota	0.916:0.078:0.003:0.003	0.908:0.082:0.005:0.005	0.902:0.092:0.003:0.003	0.892:0.097:0.005:0.006	0.92:0.074:0.003:0.003	0.918:0.072:0.005:0.005
Mammalia	0.984:0.008:0.004:0.004	0.977:0.01:0.006:0.006	0.983:0.008:0.005:0.005	0.977:0.01:0.006:0.006	0.984:0.007:0.005:0.004	0.978:0.01:0.006:0.006
Reptilia	0.909:0.089:0.001:0.001	0.903:0.093:0.002:0.002	0.904:0.094:0.001:0.001	0.895:0.1:0.002:0.003	0.913:0.085:0.001:0.001	0.912:0.083:0.002:0.002
Diapsida s.l./s.s.	0.971:0.015:0.007:0.007	0.959:0.017:0.011:0.013	0.965:0.018:0.01:0.007	0.954:0.021:0.014:0.011	0.97:0.014:0.008:0.008	0.958:0.016:0.012:0.014
Lepidosauria	0:0.052:0.202:0.746	0:0.086:0.276:0.638	0:0.058:0.241:0.702	0:0.089:0.319:0.592	0:0.056:0.182:0.762	0:0.093:0.262:0.645
Archelosauria	0.989:0.006:0.002:0.003	0.983:0.008:0.004:0.005	0.986:0.008:0.004:0.002	0.98:0.01:0.006:0.004	0.989:0.006:0.003:0.003	0.983:0.007:0.005:0.005
Marine reptiles + Archosauromorpha	0.999:0.001:0:0	0.998:0.001:0.001:0	0.999:0.001:0:0	0.998:0.001:0.001:0	0.999:0.001:0:0	0.998:0.001:0:0
Archosauromorpha	0.958:0.028:0.008:0.005	0.955:0.027:0.01:0.008	0.953:0.028:0.013:0.007	0.952:0.027:0.013:0.008	0.958:0.027:0.009:0.006	0.958:0.024:0.01:0.007
Archosauria	0.014:0.847:0.096:0.042	0.027:0.786:0.116:0.071	0.016:0.836:0.096:0.052	0.03:0.766:0.118:0.086	0.014:0.824:0.118:0.043	0.027:0.736:0.154:0.083
Dinosauria	0.009:0.88:0.077:0.034	0.015:0.834:0.094:0.057	0.011:0.875:0.076:0.038	0.018:0.819:0.096:0.068	0.009:0.864:0.093:0.033	0.015:0.799:0.122:0.063
Saurischia	0.005:0.875:0.088:0.032	0.009:0.833:0.103:0.055	0.004:0.869:0.089:0.038	0.008:0.818:0.108:0.066	0.005:0.863:0.101:0.031	0.01:0.803:0.127:0.06
Theropoda	0.001:0.001:0.001:0.998	0.001:0.001:0.001:0.998	0.001:0.001:0.001:0.997	0.001:0.001:0.001:0.997	0.001:0.001:0.002:0.996	0.001:0.001:0.002:0.996

Table S44f. Continuation of Table S44e.

Character	Reproduction mode + egg shell mineralisation

Model	mbl-CSYM (sw.hom)	mbl-CSYM (sw.het)	mbl-CARD (ind.hom)	mbl-CARD (ind.het)	mbl-CARD (sw.hom)	mbl-CARD (sw.het)
Mean log marginal likelihood	-73.331	-72.140	-72.447	-71.816	-71.227	-70.450
Mean log Bayes Factor	-1.897	0.484	-0.130	1.133	2.310	3.865
Amniota	0.913:0.08:0.003:0.003	0.909:0.08:0.006:0.005	0.889:0.107:0.003:0.001	0.895:0.099:0.004:0.002	0.917:0.082:0.001:0	0.923:0.075:0.001:0.001
Mammalia	0.983:0.007:0.005:0.004	0.977:0.01:0.007:0.006	0.984:0.01:0.004:0.002	0.98:0.012:0.005:0.003	0.989:0.008:0.001:0.001	0.987:0.01:0.002:0.001
Reptilia	0.913:0.084:0.001:0.001	0.909:0.086:0.003:0.002	0.879:0.12:0.001:0	0.881:0.117:0.001:0.001	0.852:0.148:0:0	0.861:0.138:0.001:0
Diapsida s.l./s.s.	0.963:0.019:0.012:0.007	0.953:0.02:0.015:0.012	0.973:0.019:0.005:0.003	0.965:0.022:0.007:0.006	0.959:0.036:0.003:0.001	0.957:0.038:0.004:0.002
Lepidosauria	0:0.063:0.222:0.715	0:0.111:0.289:0.599	0:0.061:0.189:0.751	0:0.088:0.243:0.668	0:0.063:0.245:0.691	0:0.101:0.283:0.616
Archelosauria	0.986:0.008:0.004:0.002	0.98:0.009:0.006:0.005	0.989:0.008:0.002:0.001	0.985:0.01:0.003:0.002	0.985:0.013:0.001:0	0.982:0.015:0.002:0.001
Marine reptiles + Archosauromorpha	0.999:0.001:0:0	0.998:0.001:0.001:0	0.999:0.001:0:0	0.999:0.001:0:0	0.999:0.001:0:0	0.999:0.001:0:0
Archosauromorpha	0.954:0.026:0.013:0.007	0.955:0.023:0.013:0.009	0.961:0.031:0.006:0.002	0.96:0.032:0.006:0.003	0.961:0.034:0.003:0.001	0.961:0.034:0.003:0.002
Archosauria	0.014:0.808:0.125:0.053	0.026:0.721:0.156:0.097	0.019:0.897:0.077:0.007	0.033:0.865:0.083:0.02	0.038:0.928:0.03:0.004	0.059:0.897:0.035:0.01
Dinosauria	0.01:0.856:0.097:0.038	0.013:0.79:0.124:0.072	0.012:0.923:0.06:0.006	0.02:0.899:0.066:0.015	0.022:0.952:0.024:0.003	0.033:0.932:0.027:0.007
Saurischia	0.004:0.856:0.105:0.035	0.006:0.797:0.128:0.068	0.007:0.921:0.066:0.006	0.013:0.901:0.071:0.015	0.005:0.964:0.028:0.003	0.009:0.952:0.031:0.007

Theropoda	0.001:0.002:0.003:0.994	0.001:0.002:0.003:0.995	0.001:0.001:0.001:0.997	0.001:0.001:0.001:0.996	0.001:0.001:0.001:0.996	0.001:0.002:0.001:0.996

Table S44g. Continuation of Table S44f.

Character	Reproduction mode + egg shell mineralisation			
Model	mbl-ER (ind.hom)	mbl-ER (ind.het)	mbl-ER (sw.hom)	mbl-ER (sw.het)
Mean log marginal likelihood	-71.657	-70.804	-72.382	-71.365
Mean log Bayes Factor	1.450	3.157	0	2.035
Amniota	0.909:0.084:0.003:0.003	0.894:0.092:0.007:0.007	0.893:0.1:0.003:0.004	0.874:0.112:0.007:0.007
Mammalia	0.983:0.008:0.005:0.005	0.974:0.011:0.008:0.008	0.982:0.008:0.005:0.005	0.972:0.012:0.008:0.008
Reptilia	0.904:0.094:0.001:0.001	0.893:0.102:0.003:0.003	0.897:0.1:0.001:0.001	0.881:0.113:0.003:0.003
Diapsida s.l./s.s.	0.972:0.015:0.006:0.007	0.958:0.017:0.011:0.013	0.965:0.018:0.01:0.007	0.952:0.023:0.014:0.012
Lepidosauria	0:0.054:0.184:0.762	0:0.085:0.259:0.656	0:0.057:0.235:0.708	0:0.09:0.301:0.61
Archelosauria	0.988:0.006:0.003:0.003	0.981:0.009:0.005:0.006	0.985:0.009:0.004:0.002	0.977:0.012:0.006:0.005
Marine reptiles + Archosauromorpha	0.999:0.001:0:0	0.998:0.001:0.001:0.001	0.999:0.001:0:0	0.997:0.002:0.001:0.001
Archosauromorpha	0.958:0.028:0.008:0.006	0.955:0.028:0.01:0.008	0.952:0.028:0.012:0.007	0.952:0.026:0.013:0.009
Archosauria	0.017:0.844:0.097:0.042	0.031:0.78:0.117:0.071	0.019:0.836:0.095:0.05	0.036:0.762:0.12:0.083
Dinosauria	0.01:0.877:0.078:0.034	0.018:0.828:0.095:0.058	0.012:0.874:0.075:0.038	0.019:0.82:0.096:0.065
Saurischia	0.006:0.874:0.088:0.032	0.011:0.828:0.104:0.057	0.005:0.869:0.089:0.037	0.009:0.818:0.109:0.064
Theropoda	0.001:0.001:0.001:0.998	0.001:0.001:0.001:0.998	0.001:0.001:0.001:0.997	0.001:0.001:0.001:0.997

Table S45a. Same as Table S29 but adding extinct marine reptiles as sister taxon to Lepidosauria (mbl dating method).

Character	Reproduction mode + egg shell mineralisation

Model	mbl-CER (ind.hom)	mbl-CER (ind.het)	mbl-CER (sw.hom)	mbl-CER (sw.het)	mbl-CSYM (ind.hom)	mbl-CSYM (ind.het)
Mean log marginal likelihood	-71.129	-70.174	-71.792	-70.632	-73.031	-71.940
Mean log Bayes Factor	2.486	4.396	1.160	3.481	-1.319	0.864
Amniota	0.917:0.077:0.003:0.003	0.915:0.076:0.005:0.005	0.912:0.081:0.003:0.003	0.905:0.084:0.006:0.006	0.921:0.073:0.003:0.003	0.919:0.071:0.005:0.005
Mammalia	0.984:0.007:0.004:0.004	0.977:0.01:0.006:0.006	0.983:0.008:0.005:0.005	0.975:0.011:0.007:0.007	0.983:0.007:0.005:0.004	0.978:0.01:0.006:0.006
Reptilia	0.913:0.085:0.001:0.001	0.911:0.085:0.002:0.002	0.918:0.08:0.001:0.001	0.911:0.084:0.002:0.002	0.917:0.081:0.001:0.001	0.917:0.079:0.002:0.002
Diapsida s.l./s.s.	0.997:0.002:0.001:0.001	0.995:0.002:0.001:0.002	0.996:0.002:0.001:0.001	0.993:0.003:0.002:0.002	0.997:0.002:0.001:0.001	0.995:0.002:0.002:0.002
Marine reptiles + Lepidosauria	0.99:0.003:0.003:0.004	0.987:0.004:0.005:0.005	0.989:0.003:0.004:0.004	0.985:0.004:0.005:0.005	0.99:0.003:0.003:0.004	0.987:0.004:0.005:0.005
Lepidosauria	0.133:0.046:0.165:0.655	0.187:0.065:0.233:0.515	0.109:0.051:0.216:0.625	0.192:0.071:0.267:0.47	0.128:0.049:0.162:0.66	0.179:0.075:0.218:0.528
Archelosauria	0.916:0.054:0.015:0.015	0.911:0.05:0.018:0.021	0.884:0.066:0.029:0.021	0.891:0.057:0.028:0.025	0.912:0.054:0.019:0.016	0.907:0.048:0.023:0.022
Archosauromorpha	0.955:0.031:0.009:0.006	0.953:0.029:0.01:0.008	0.949:0.031:0.013:0.007	0.948:0.029:0.014:0.009	0.955:0.029:0.009:0.006	0.952:0.028:0.012:0.009
Archosauria	0.016:0.85:0.091:0.043	0.03:0.782:0.113:0.074	0.017:0.84:0.091:0.052	0.032:0.765:0.117:0.086	0.017:0.828:0.111:0.045	0.03:0.746:0.145:0.08
Dinosauria	0.01:0.881:0.075:0.035	0.017:0.833:0.091:0.06	0.011:0.878:0.072:0.039	0.018:0.822:0.093:0.066	0.01:0.867:0.088:0.034	0.018:0.804:0.116:0.062
Saurischia	0.006:0.88:0.082:0.032	0.01:0.836:0.097:0.056	0.005:0.875:0.083:0.03	0.008:0.823:0.103:0.06	0.006:0.869:0.093:0.03	0.011:0.811:0.12:0.059

			7	5	1	
Theropoda	0.001:0.001:0.001:0.998	0.001:0.001:0.001:0.997	0.001:0.001:0.001:0.997	0.001:0.001:0.001:0.997	0.001:0.001:0.002:0.996	0.001:0.001:0.002:0.996

Table S45b. Continuation of Table S45a.

Character	Reproduction mode + egg shell mineralisation					
Model	mbl-CSYM (sw.hom)	mbl-CSYM (sw.het)	mbl-CARD (ind.hom)	mbl-CARD (ind.het)	mbl-CARD (sw.hom)	mbl-CARD (sw.het)
Mean log marginal likelihood	-73.351	-72.030	-68.103	-66.565	-70.435	-68.505
Mean log Bayes Factor	-1.957	0.683	8.539	11.614	3.874	7.735
Amniota	0.922:0.071:0.003:0.003	0.917:0.072:0.006:0.005	0.998:0.002:0:0	0.999:0.001:0:0	0.969:0.03:0:0	0.987:0.013:0:0
Mammalia	0.983:0.007:0.005:0.005	0.976:0.01:0.007:0.007	1:0:0:0	1:0:0:0	0.996:0.003:0.001:0	0.998:0.002:0:0
Reptilia	0.925:0.072:0.001:0.001	0.921:0.074:0.002:0.002	0.998:0.002:0:0	0.999:0.001:0:0	0.947:0.053:0:0	0.977:0.023:0:0
Diapsida s.l./s.s.	0.996:0.002:0.001:0.001	0.993:0.003:0.002:0.002	1:0:0:0	1:0:0:0	0.998:0.001:0:0	0.999:0.001:0:0
Marine reptiles + Lepidosauria	0.989:0.003:0.004:0.004	0.986:0.004:0.005:0.005	1:0:0:0	1:0:0:0	0.996:0.001:0.001:0.002	0.998:0.001:0.001:0.001
Lepidosauria	0.097:0.058:0.201:0.644	0.159:0.094:0.24:0.508	0.988:0.001:0.002:0.008	0.993:0.001:0.002:0.005	0.648:0.023:0.085:0.244	0.854:0.014:0.041:0.091
Archelosauria	0.879:0.066:0.033:0.022	0.888:0.054:0.032:0.026	0.999:0.001:0:0	0.999:0.001:0:0	0.961:0.034:0.003:0.001	0.983:0.015:0.001:0.001
Archosauromorpha	0.95:0.029:0.014:0.007	0.95:0.026:0.014:0.01	0.999:0:0:0	1:0:0:0	0.984:0.014:0.002:0	0.993:0.006:0.001:0
Archosau	0.015:0.814	0.03:0.722:	0.019:0.877	0.055:0.813	0.043:0.879	0.116:0.774

ria	:0.117:0.05 3	0.152:0.097	:0.096:0.00 8	:0.109:0.02 3	:0.07:0.008	:0.092:0.01 8
Dinosauri a	0.01:0.86:0. 092:0.038	0.017:0.789 :0.12:0.074	0.012:0.908 :0.074:0.00 5	0.037:0.862 :0.086:0.01 5	0.027:0.913 :0.054:0.00 5	0.075:0.84: 0.075:0.011
Saurischi a	0.004:0.862 :0.098:0.03 6	0.008:0.797 :0.124:0.07	0.008:0.912 :0.076:0.00 5	0.025:0.882 :0.081:0.01 3	0.005:0.935 :0.056:0.00 4	0.022:0.896 :0.073:0.00 9
Theropod a	0.001:0.002 :0.003:0.99 4	0.001:0.002 :0.002:0.99 5	0.001:0.001 :0.001:0.99 7	0.003:0.003 :0.003:0.99 1	0.001:0.003 :0.002:0.99 4	0.003:0.004 :0.003:0.99

Table S45c. Continuation of Table S45b.

Character	Reproduction mode + egg shell mineralisation			
Model	mbl-ER (ind.hom)	mbl-ER (ind.het)	mbl-ER (sw.hom)	mbl-ER (sw.het)
Mean log marginal likelihood	-71.532	-70.562	-72.372	-71.195
Mean log Bayes Factor	1.679	3.619	0	2.354
Amniota	0.911:0.082:0.003 :0.003	0.899:0.087:0.007 :0.007	0.902:0.091:0.003 :0.004	0.884:0.101:0.007 :0.008
Mammalia	0.983:0.008:0.005 :0.005	0.973:0.011:0.008 :0.008	0.982:0.008:0.005 :0.005	0.972:0.012:0.008 :0.008
Reptilia	0.908:0.09:0.001: 0.001	0.9:0.095:0.003:0. 003	0.908:0.089:0.001 :0.001	0.894:0.1:0.003:0. 003
Diapsida s.l./s.s.	0.997:0.002:0.001 :0.001	0.993:0.003:0.002 :0.002	0.995:0.002:0.001 :0.001	0.991:0.004:0.003 :0.003
Marine reptiles + Lepidosauria	0.989:0.003:0.003 :0.004	0.985:0.005:0.005 :0.005	0.988:0.003:0.004 :0.004	0.984:0.005:0.005 :0.006
Lepidosauria	0.147:0.046:0.154 :0.653	0.218:0.064:0.206 :0.513	0.124:0.05:0.204: 0.622	0.219:0.066:0.243 :0.472
Archelosauria	0.919:0.052:0.015 :0.014	0.913:0.049:0.018 :0.021	0.892:0.062:0.027 :0.019	0.896:0.054:0.026 :0.024
Archosauromo rpha	0.955:0.031:0.009 :0.006	0.951:0.03:0.01:0. 008	0.949:0.031:0.013 :0.007	0.947:0.029:0.014 :0.01
Archosauria	0.018:0.847:0.091 :0.043	0.036:0.775:0.114 :0.075	0.021:0.837:0.09: 0.052	0.04:0.757:0.115: 0.088
Dinosauria	0.012:0.878:0.075 :0.035	0.02:0.825:0.093: 0.061	0.013:0.875:0.073 :0.039	0.022:0.814:0.094 :0.07

Saurischia	0.007:0.879:0.082:0.032	0.013:0.831:0.099:0.058	0.006:0.873:0.084:0.037	0.01:0.817:0.105:0.068
Theropoda	0.001:0.001:0.001:0.998	0.001:0.001:0.001:0.997	0.001:0.001:0.001:0.997	0.001:0.001:0.001:0.997

Table S45d. Continuation of Table S45c.

Character	EER			
Model	mbl-EER ER (hom)	mbl-EER ER (het)	mbl-EER ARD (hom)	mbl-EER ARD (het)
Mean log marginal likelihood	-34.269	-33.311	-33.029	-32.228
Mean log Bayes Factor	0	1.915	2.480	4.082
Amniota	0.006:0.994	0.01:0.99	0.002:0.998	0.005:0.995
Mammalia	0.016:0.984	0.023:0.977	0.007:0.993	0.011:0.989
Reptilia	0.001:0.999	0.003:0.997	0:1	0.001:0.999
Diapsida s.l./s.s.	0.002:0.998	0.004:0.996	0.001:0.999	0.002:0.998
Marine reptiles + Lepidosauria	0.004:0.996	0.008:0.992	0.002:0.998	0.004:0.996
Lepidosauria	0.176:0.824	0.254:0.746	0.069:0.931	0.137:0.863
Archelosauria	0.104:0.896	0.111:0.889	0.052:0.948	0.063:0.937
Archosauromorpha	0.042:0.958	0.05:0.95	0.02:0.98	0.025:0.975
Archosauria	0.988:0.012	0.987:0.013	0.994:0.006	0.982:0.018
Dinosauria	0.993:0.007	0.993:0.007	0.996:0.004	0.989:0.011
Saurischia	0.995:0.005	0.995:0.005	0.998:0.002	0.993:0.007
Theropoda	0.999:0.001	0.999:0.001	1:0	0.999:0.001

Table S45e. Continuation of Table S45d. Nodes of Lepidosauria and Squamata fixed to a non-viviparous state.

Character	Reproduction mode + egg shell mineralisation					
Model	mbl-CER (ind.hom)	mbl-CER (ind.het)	mbl-CER (sw.hom)	mbl-CER (sw.het)	mbl-CSYM (ind.hom)	mbl-CSYM (ind.het)
Mean log marginal likelihood	-71.330	-70.462	-71.903	-70.850	-73.219	-72.202
Mean log Bayes Factor	2.332	4.068	1.185	3.292	-1.446	0.587
Amniota	0.92:0.074:0.003:0.003	0.919:0.072:0.004:0.00	0.912:0.082:0.003:0.00	0.906:0.084:0.005:0.00	0.926:0.068:0.003:0.00	0.927:0.063:0.005:0.00

		5	3	6	3	5
Mammalia	0.984:0.007:0.004:0.004	0.979:0.01:0.006:0.006	0.983:0.008:0.005:0.005	0.976:0.011:0.006:0.006	0.984:0.007:0.004:0.004	0.978:0.01:0.006:0.006
Reptilia	0.915:0.083:0.001:0.001	0.916:0.081:0.002:0.002	0.915:0.083:0.001:0.001	0.911:0.085:0.002:0.002	0.92:0.078:0.001:0.001	0.924:0.072:0.002:0.002
Diapsida s.l./s.s.	0.997:0.002:0.001:0.001	0.995:0.002:0.001:0.002	0.996:0.002:0.001:0.001	0.993:0.003:0.002:0.002	0.997:0.002:0.001:0.001	0.994:0.002:0.002:0.002
Marine reptiles + Lepidosauria	0.989:0.003:0.004:0.004	0.986:0.004:0.005:0.005	0.988:0.003:0.004:0.004	0.985:0.004:0.005:0.005	0.989:0.003:0.004:0.004	0.985:0.004:0.005:0.005
Lepidosauria	0:0.054:0.189:0.758	0:0.081:0.282:0.637	0:0.058:0.241:0.701	0:0.09:0.317:0.593	0:0.055:0.189:0.756	0:0.094:0.267:0.64
Archelosauria	0.915:0.055:0.016:0.015	0.91:0.05:0.018:0.021	0.882:0.067:0.029:0.021	0.89:0.058:0.027:0.026	0.909:0.056:0.019:0.016	0.902:0.05:0.024:0.024
Archosauromorpha	0.955:0.031:0.008:0.006	0.954:0.029:0.01:0.008	0.949:0.031:0.013:0.007	0.949:0.029:0.013:0.009	0.956:0.03:0.009:0.006	0.953:0.027:0.012:0.008
Archosauria	0.015:0.853:0.09:0.043	0.029:0.784:0.114:0.074	0.016:0.842:0.09:0.052	0.03:0.772:0.113:0.085	0.014:0.835:0.107:0.043	0.027:0.743:0.147:0.082
Dinosauria	0.009:0.882:0.074:0.035	0.017:0.832:0.092:0.059	0.011:0.879:0.072:0.039	0.017:0.826:0.09:0.066	0.009:0.871:0.087:0.034	0.015:0.804:0.117:0.063
Saurischia	0.006:0.881:0.082:0.032	0.01:0.835:0.099:0.056	0.005:0.875:0.083:0.037	0.008:0.827:0.101:0.064	0.005:0.871:0.093:0.031	0.01:0.81:0.121:0.059
Theropoda	0.001:0.001:0.001:0.998	0.001:0.001:0.001:0.998	0.001:0.001:0.001:0.997	0.001:0.001:0.001:0.997	0.001:0.001:0.002:0.996	0.001:0.001:0.002:0.996

Table S45f. Continuation of Table S45e.

Character	Reproduction mode + egg shell mineralisation					
Model	mbl-CSYM (sw.hom)	mbl-CSYM (sw.het)	mbl-CARD (ind.hom)	mbl-CARD (ind.het)	mbl-CARD (sw.hom)	mbl-CARD (sw.het)
Mean log marginal	-73.450	-72.224	-72.567	-71.899	-71.384	-70.563

likelihood						
Mean log Bayes Factor	-1.908	0.544	-0.142	1.194	2.224	3.865
Amniota	0.923:0.071:0.003:0.003	0.922:0.068:0.005:0.005	0.895:0.101:0.003:0.001	0.909:0.085:0.004:0.002	0.927:0.072:0.001:0	0.934:0.064:0.001:0.001
Mammalia	0.984:0.007:0.005:0.004	0.978:0.01:0.006:0.006	0.984:0.01:0.004:0.002	0.981:0.012:0.004:0.003	0.99:0.008:0.001:0.001	0.987:0.01:0.001:0.001
Reptilia	0.924:0.073:0.001:0.001	0.923:0.072:0.002:0.002	0.887:0.112:0.001:0	0.897:0.101:0.001:0.001	0.869:0.131:0:0	0.88:0.119:0.001:0
Diapsida s.l./s.s.	0.996:0.002:0.001:0.001	0.994:0.003:0.002:0.002	0.997:0.002:0.001:0	0.995:0.003:0.001:0.001	0.996:0.003:0:0	0.995:0.004:0.001:0
Marine reptiles + Lepidosauria	0.989:0.003:0.004:0.004	0.985:0.005:0.005:0.005	0.989:0.003:0.003:0.004	0.987:0.004:0.004:0.005	0.989:0.003:0.003:0.005	0.987:0.004:0.004:0.005
Lepidosauria	0:0.063:0.225:0.712	0:0.108:0.292:0.6	0:0.06:0.187:0.753	0:0.089:0.249:0.662	0:0.065:0.247:0.689	0:0.101:0.291:0.608
Archelosauria	0.877:0.067:0.034:0.022	0.886:0.055:0.032:0.027	0.925:0.06:0.011:0.004	0.921:0.06:0.011:0.007	0.903:0.087:0.008:0.003	0.907:0.083:0.007:0.004
Archosauromorpha	0.95:0.029:0.014:0.007	0.95:0.026:0.014:0.01	0.959:0.033:0.006:0.002	0.959:0.032:0.006:0.002	0.96:0.035:0.003:0.001	0.96:0.035:0.003:0.001
Archosauria	0.014:0.816:0.116:0.053	0.025:0.731:0.152:0.092	0.02:0.9:0.073:0.007	0.034:0.867:0.08:0.018	0.039:0.928:0.029:0.005	0.062:0.894:0.033:0.011
Dinosauria	0.01:0.861:0.091:0.038	0.014:0.799:0.119:0.068	0.013:0.925:0.057:0.006	0.021:0.9:0.065:0.014	0.022:0.953:0.022:0.003	0.035:0.93:0.026:0.008
Saurischia	0.004:0.862:0.098:0.036	0.007:0.805:0.124:0.065	0.008:0.925:0.062:0.005	0.014:0.904:0.069:0.014	0.005:0.965:0.026:0.003	0.01:0.951:0.03:0.009
Theropoda	0.001:0.002:0.003:0.995	0.001:0.002:0.003:0.995	0.001:0.001:0.001:0.997	0.001:0.001:0.001:0.996	0.001:0.002:0.001:0.996	0.001:0.002:0.002:0.995

Table S45g. Continuation of Table S45f.

Character	Reproduction mode + egg shell mineralisation			
Model	mbl-ER (ind.hom)	mbl-ER (ind.het)	mbl-ER (sw.hom)	mbl-ER (sw.het)
Mean log marginal likelihood	-71.747	-70.888	-72.496	-71.453
Mean log Bayes Factor	1.497	3.215	0	2.086
Amniota	0.914:0.079:0.003:0.003	0.905:0.084:0.006:0.006	0.901:0.092:0.003:0.004	0.889:0.098:0.006:0.007
Mammalia	0.983:0.008:0.005:0.005	0.975:0.011:0.007:0.007	0.982:0.008:0.005:0.005	0.974:0.011:0.007:0.007
Reptilia	0.91:0.088:0.001:0.001	0.904:0.092:0.002:0.002	0.905:0.092:0.001:0.001	0.896:0.099:0.002:0.003
Diapsida s.l./s.s.	0.997:0.002:0.001:0.001	0.993:0.003:0.002:0.002	0.995:0.002:0.001:0.001	0.991:0.004:0.002:0.003
Marine reptiles + Lepidosauria	0.989:0.003:0.004:0.004	0.985:0.005:0.005:0.005	0.988:0.004:0.004:0.005	0.983:0.005:0.006:0.006
Lepidosauria	0:0.055:0.18:0.765	0:0.082:0.266:0.652	0:0.058:0.229:0.713	0:0.085:0.313:0.601
Archelosauria	0.918:0.053:0.015:0.014	0.914:0.049:0.017:0.02	0.892:0.062:0.027:0.019	0.892:0.057:0.026:0.025
Archosauromorpha	0.955:0.031:0.009:0.006	0.953:0.03:0.01:0.008	0.949:0.031:0.013:0.007	0.947:0.03:0.014:0.009
Archosauria	0.017:0.85:0.09:0.042	0.034:0.782:0.111:0.073	0.02:0.839:0.09:0.052	0.036:0.769:0.111:0.083
Dinosauria	0.011:0.88:0.074:0.034	0.019:0.83:0.091:0.06	0.013:0.876:0.073:0.039	0.02:0.823:0.09:0.067
Saurischia	0.006:0.88:0.082:0.032	0.011:0.833:0.099:0.057	0.005:0.874:0.084:0.037	0.01:0.823:0.101:0.066
Theropoda	0.001:0.001:0.001:0.998	0.001:0.001:0.001:0.998	0.001:0.001:0.001:0.997	0.001:0.001:0.001:0.997

References cited in Supplementary Tables

1 Hughes, R. L. Monotreme development with particular reference to the extraembryonic membranes. *Journal of Experimental Zoology* **266**, 480-494 (1993).

2 Kielan-Jaworowska, Z. Pelvic structure and nature of reproduction in Multituberculata. *Nature* **277**, 402-403 (1979).

3 Laurin, M. Embryo retention, character optimization, and the origin of the extra - embryonic membranes of the amniotic egg. *Journal of Natural History* **39**, 3151-3161 (2005).

4 O'Harra, C. C. A fossil mammal with unborn twins. *Science* **71**, 341-342 (1930).

5 Gingerich, P. D., von Koenigswald, W., Sanders, W. J., Smith, B. H. & Zalmout, I. S. New protocetid whale from the middle Eocene of Pakistan: birth on land, precocial development, and sexual dimorphism. *PLoS One* **4**, e4366 (2009).

6 Thewissen, J. & McLellan, W. A. Maiacetus: displaced fetus or last meal. *Comment on: Gingerich, PD, ul-Haq, M., W. von Koenigswald, WJ Sanders, BH Smith, and IS Zalmout. New protocetid whale from the middle Eocene of Pakistan: birth on land, precocial development, and sexual dimorphism. PLoS ONE* **4**, e4366 (2009).

7 Blackburn, D. G. & Sidor, C. A. Evolution of viviparous reproduction in Paleozoic and Mesozoic reptiles. *International Journal of Developmental Biology* **58**, 935-948 (2015).

8 Sander, P. M. The pachypleurosaurids (Reptilia: Nothosauria) from the Middle Triassic of Monte San Giorgio (Switzerland) with the description of a new species. *Philosophical Transactions of the Royal Society of London. B, Biological Sciences* **325**, 561-666 (1989).

9 Renesto, S., Lombardo, C., Tintori, A. & Danini, G. Nothosaurid embryos from the Middle Triassic of northern Italy: an insight into the viviparity of nothosaurs? *Journal of Vertebrate Paleontology* **23**, 957-960 (2003).

10 Cheng, Y.-n., Wu, X.-c. & Ji, Q. Triassic marine reptiles gave birth to live young. *Nature* **432**, 383-386 (2004).

11 Rothschild, B. M. & Martin, L. D. *Paleopathology: disease in the fossil record*. (CRC Press, 1993).

12 O'Keefe, F. & Chiappe, L. Viviparity and K-selected life history in a Mesozoic marine plesiosaur (Reptilia, Sauropterygia). *Science* **333**, 870-873 (2011).

13 Motani, R., Jiang, D.-y., Tintori, A., Rieppel, O. & Chen, G.-b. Terrestrial origin of viviparity in Mesozoic marine reptiles indicated by Early Triassic embryonic fossils. *PloS One* **9**, e88640 (2014).

14 Brinkmann, W. Ein Mixosaurier (Reptilia, Ichthyosauria) mit Embryonen aus der Grenzbitumenzone (Mitteltrias) des Monte San Giorgio (Schweiz, Kanton Tessin). *Eclogae Geologicae Helvetiae* **89**, 1321-1344 (1996).

15 Dal Sasso, C. & Pinna, G. *Besanosaurus leptorhynchus n. gen. n. sp., a new shastasaurid ichthyosaur from the Middle Triassic of Besano (Lombardy, N. Italy)*. (Società Italiana di scienze naturali, Museo Civico di Storia Naturale di Milano, 1996).

16 Camp, C. L. & CL, C. Large ichthyosaurs from the Upper Triassic of Nevada. *Palaeontographica A* **170**, 139–200 (1980).

17 Hartman, S. *et al.* A new paravian dinosaur from the Late Jurassic of North America supports a late acquisition of avian flight. *PeerJ* **7**, e7247 (2019).

18 Xiaofeng, W. *et al.* The Late Triassic black shales of the Guanling area, Guizhou Province, south‐west China: a unique marine reptile and pelagic crinoid fossil lagerstätte. *Palaeontology* **51**, 27-61 (2008).

19 Böttcher, R. *Neue Erkenntnisse über die Fortpflanzungsbiologie der Ichthyosaurier (Reptilia)*. (Staatliches Museum für Naturkunde, 1990).

20 Motani, R. Evolution of fish-shaped reptiles (Reptilia: Ichthyopterygia) in their physical environments and constraints. *Annu. Rev. Earth Planet. Sci.* **33**, 395-420 (2005).

21 Lomax, D. R. & Massare, J. A. The first reported Leptonectes (Reptilia: Ichthyosauria) with associated embryos, from Somerset, England. *Paludicola* **8**, 263-276 (2012).

22 Deeming, D. C., Halstead, L. B., Manabe, M. & Unwin, D. M. An ichthyosaur embryo from the Lower Lias (Jurassic: Hettingan) of Somerset, England, with comments on the reproductive biology of ichthyosaurs. *Modern Geology*, 423-442 (1993).

23 McGowan, C. A revision of the Lower Jurassic ichthyosaurs of Germany with descriptions of two new species. *Palaeontographica A* **166**, 93–135 (1979).

24 Maxwell, E. E. New metrics to differentiate apecies of *Stenopterygius* (Reptilia: Ichthyosauria) from the Lower Jurassic of Southwestern Germany. *Journal of Paleontology* **86**, 105-115 (2012).

25 Maxwell, E. E. & Caldwell, M. W. First record of live birth in Cretaceous ichthyosaurs: closing an 80 million year gap. *Proceedings of the Royal Society of London. Series B: Biological Sciences* **270**, S104-S107 (2003).

26 Maxwell, E. & Caldwell, M. A new genus of ichthyosaur from the Lower Cretaceous of Western Canada. *Palaeontology* **49**, 1043-1052 (2006).

27 Kear, B. P., Boles, W. E. & Smith, E. T. Unusual gut contents in a Cretaceous ichthyosaur. *Proceedings of the Royal Society of London. Series B: Biological Sciences* **270**, S206-S208 (2003).

28 Kear, B. P. & Zammit, M. In utero foetal remains of the Cretaceous ichthyosaurian Platypterygius: ontogenetic implications for character state efficacy. *Geological Magazine* **151**, 71-86 (2014).

29 Packard, M. J., Packard, G. C. & Boardman, T. J. Structure of eggshells and water relations of reptilian eggs. *Herpetologica*, 136-155 (1982).

30 Moffat, L. Embryonic development and aspects of reproductive biology in the Tuatara, Sphenodon punctatus. *Biology of the Reptilia* **14**, 493-521 (1985).

31 Wang, Y. & Evans, S. E. A gravid lizard from the Cretaceous of China and the early history of squamate viviparity. *Naturwissenschaften* **98**, 739-743 (2011).

32 Shine, R. Reptilian reproductive modes: the oviparity-viviparity continuum. *Herpetologica*, 1-8 (1983).

33 Deeming, D. Eggshell structure of lizards of two sub families of the Gekkonidae.

Herpetological journal **1**, 230-234 (1988).
34 Kluge, A. G. Higher taxonomic categories of gekkonid lizards and their evolution. Bulletin of the AMNH; v. 135, article 1. (1967).
35 Lucas, A. H. S. & Frost, C. *The lizards indigenous to Victoria*. (Royal Society of Victoria, 1893).
36 Schleich, H.-H. & Kästle, W. *Reptile egg-shells*. (G. Fischer, 1988).
37 Caldwell, M. W. & Lee, M. S. Live birth in Cretaceous marine lizards (mosasauroids). *Proceedings of the Royal Society of London. Series B: Biological Sciences* **268**, 2397-2401 (2001).
38 Bell, G., Sheldon, M., Lamb, J. & Martin, J. The first direct evidence of live birth in Mosasauridae (Squamata): Exceptional preservation in Cretaceous Pierre Shale of South Dakota. *Journal of Vertebrate Paleontology* **16**, 21A (1996).
39 Fernandez, V. *et al.* Evidence of egg diversity in squamate evolution from Cretaceous anguimorph embryos. *PLoS One* **10**, e0128610 (2015).
40 Barbour, T. & Ramsden, C. T. *The herpetology of Cuba*. Vol. 47 (Museum, 1919).
41 Gray, J. E. *Catalogue of the Specimens of Lizards in the Collection of the British Museum*. (order of the Trustees, 1845).
42 Packard, M. J. & DeMarco, V. G. Eggshell structure and formation in eggs of oviparous reptiles. *Egg incubation: its effects on embryonic development in birds and reptiles*, 53-69 (1991).
43 Kusuda, S. *et al.* Diversity in the matrix structure of eggshells in the Testudines (Reptilia). *Zoological science* **30**, 366-374 (2013).
44 Zelenitsky, D. K., Therrien, F., Joyce, W. G. & B. Brinkman, D. First fossil gravid turtle provides insight into the evolution of reproductive traits in turtles. *Biology Letters* **4**, 715-718 (2008).
45 Liu, J., Organ, C. L., Benton, M. J., Brandley, M. C. & Aitchison, J. C. Live birth in an archosauromorph reptile. *Nature Communications* **8**, 1-8 (2017).
46 Lü, J., Kobayashi, Y., Deeming, D. C. & Liu, Y. Post-natal parental care in a Cretaceous diapsid from northeastern China. *Geosciences Journal* **19**, 273-280 (2015).
47 Wang, X., Miao, D. & Zhang, Y. Cannibalism in a semi-aquatic reptile from the Early Cretaceous of China. *Chin Sci Bull* **50**, 281-283 (2005).
48 Chiappe, L. M., Codorniú, L., Grellet-Tinner, G. & Rivarola, D. Argentinian unhatched pterosaur fossil. *Nature* **432**, 571-572 (2004).
49 Norell, M. A. *et al.* The first dinosaur egg was soft. *Nature* **583**, 406-410 (2020).
50 Wang, X. *et al.* Sexually dimorphic tridimensionally preserved pterosaurs and their eggs from China. *Current Biology* **24**, 1323-1330 (2014).
51 Wang, X. *et al.* Egg accumulation with 3D embryos provides insight into the life history of a pterosaur. *Science* **358**, 1197-1201 (2017).
52 Ji, Q. *et al.* Pterosaur egg with a leathery shell. *Nature* **432**, 572-572 (2004).
53 Wang, X. & Zhou, Z. Pterosaur embryo from the Early Cretaceous. *Nature* **429**, 621-621 (2004).
54 Museum, R. C. o. S. o. E. *Descriptive Catalogue of the Fossil Organic Remains of Reptilia and Pisces Contained in the Museum of the Royal College of Surgeons of*

England. (Taylor & Francis, 1854).
55 Yates, A. M. & Barrett, P. M. Massospondylus carinatus Owen 1854 (Dinosauria: Sauropodomorpha) from the Lower Jurassic of South Africa: Proposed conservation of the usage by designation of a neotype. (2010).
56 Cerda, I. A., Pol, D. & Chinsamy, A. Osteohistological insight into the early stages of growth in Mussaurus patagonicus (Dinosauria, Sauropodomorpha). *Historical Biology* **26**, 110-121 (2014).
57 Wiemann, J., Yang, T.-R. & Norell, M. A. Dinosaur egg colour had a single evolutionary origin. *Nature* **563**, 555-558 (2018).
58 Simoes, T. R. *et al.* The origin of squamates revealed by a Middle Triassic lizard from the Italian Alps. *Nature* **557**, 706-709 (2018).
59 Matsumoto, R., Dong, L., Wang, Y. & Evans, S. E. The first record of a nearly complete choristodere (Reptilia: Diapsida) from the Upper Jurassic of Hebei Province, People's Republic of China. *Journal of Systematic Palaeontology* **17**, 1031-1048 (2019).
60 Ezcurra, M. D. The phylogenetic relationships of basal archosauromorphs, with an emphasis on the systematics of proterosuchian archosauriforms. *PeerJ* **4**, e1778 (2016).
61 Moon, B. C. A new phylogeny of ichthyosaurs (Reptilia: Diapsida). *Journal of Systematic Palaeontology* **17**, 129-155 (2019).
62 Andres, B., Clark, J. & Xu, X. The earliest pterodactyloid and the origin of the group. *Current Biology* **24**, 1011-1016 (2014).
63 Zhou, C.-F., Wu, S., Martin, T. & Luo, Z.-X. A Jurassic mammaliaform and the earliest mammalian evolutionary adaptations. *Nature* **500**, 163-167 (2013).
64 Otero, A. & Pol, D. Postcranial anatomy and phylogenetic relationships of Mussaurus patagonicus (Dinosauria, Sauropodomorpha). *Journal of Vertebrate Paleontology* **33**, 1138-1168 (2013).
65 Cau, A. *et al.* Synchrotron scanning reveals amphibious ecomorphology in a new clade of bird-like dinosaurs. *Nature* **552**, 395-399 (2017).
66 Han, F., Forster, C. A., Xu, X. & Clark, J. M. Postcranial anatomy of Yinlong downsi (Dinosauria: Ceratopsia) from the Upper Jurassic Shishugou Formation of China and the phylogeny of basal ornithischians. *Journal of Systematic Palaeontology* **16**, 1159-1187 (2018).
67 Pereira, A. G., Sterli, J., Moreira, F. R. & Schrago, C. G. Multilocus phylogeny and statistical biogeography clarify the evolutionary history of major lineages of turtles. *Molecular Phylogenetics and Evolution* **113**, 59-66 (2017).
68 Pyron, R. A., Burbrink, F. T. & Wiens, J. J. A phylogeny and revised classification of Squamata, including 4161 species of lizards and snakes. *BMC evolutionary biology* **13**, 1-54 (2013).
