# Supplementary material for: Extended embryo retention and viviparity in the first amniotes
Source: Nat Ecol Evol. 2023 Jun 12;7(7):1131–40. doi: 10.1038/s41559-023-02074-0 (PMC10333127; doi:10.1038/s41559-023-02074-0)
Supplement: Supplementary file 2 — Reporting Summary [file 41559_2023_2074_MOESM2_ESM.pdf]

nature portfolio

Corresponding author(s): Baoyu Jiang

Last updated by author(s): Apr 8, 2023

Reporting Summary

Nature Portfolio wishes to improve the reproducibility of the work that we publish. This form provides structure for consistency and transparency in reporting. For further information on Nature Portfolio policies, see our Editorial Policies and the Editorial Policy Checklist.

Statistics

For all statistical analyses, confirm that the following items are present in the figure legend, table legend, main text, or Methods section.

n/a	Confirmed	
☐	☒	The exact sample size (n) for each experimental group/condition, given as a discrete number and unit of measurement
☒	☐	A statement on whether measurements were taken from distinct samples or whether the same sample was measured repeatedly
☒	☐	The statistical test(s) used AND whether they are one- or two-sided<br>*Only common tests should be described solely by name; describe more complex techniques in the Methods section.*
☒	☐	A description of all covariates tested
☒	☐	A description of any assumptions or corrections, such as tests of normality and adjustment for multiple comparisons
☒	☐	A full description of the statistical parameters including central tendency (e.g. means) or other basic estimates (e.g. regression coefficient) AND variation (e.g. standard deviation) or associated estimates of uncertainty (e.g. confidence intervals)
☒	☐	For null hypothesis testing, the test statistic (e.g. F, t, r) with confidence intervals, effect sizes, degrees of freedom and P value noted<br>*Give P values as exact values whenever suitable.*
☐	☒	For Bayesian analysis, information on the choice of priors and Markov chain Monte Carlo settings
☐	☒	For hierarchical and complex designs, identification of the appropriate level for tests and full reporting of outcomes
☒	☐	Estimates of effect sizes (e.g. Cohen's d, Pearson's r), indicating how they were calculated

Our web collection on statistics for biologists contains articles on many of the points above.

Software and code

Policy information about availability of computer code

Data collection	*Provide a description of all commercial, open source and custom code used to collect the data in this study, specifying the version used OR state that no software was used.*
Data analysis	All analyses in this study were conducted using readily available, published programs and are cited in the text. Version numbers of the programs we used are as follows: R version 4.1.0; ape 5.5; castor 1.6.7; paleotree 3.3.25; phangorn 2.7.0; phytools 0.7-70; strap 1.4.

For manuscripts utilizing custom algorithms or software that are central to the research but not yet described in published literature, software must be made available to editors and reviewers. We strongly encourage code deposition in a community repository (e.g. GitHub). See the Nature Portfolio guidelines for submitting code & software for further information.

Data

Policy information about availability of data

All manuscripts must include a data availability statement. This statement should provide the following information, where applicable:

- Accession codes, unique identifiers, or web links for publicly available datasets
- A description of any restrictions on data availability
- For clinical datasets or third party data, please ensure that the statement adheres to our policy

We provide all data in Supplementary data. The phylogeny we used in the study is shown in Figure 4.

Field-specific reporting

Please select the one below that is the best fit for your research. If you are not sure, read the appropriate sections before making your selection.

☐ Life sciences ☐ Behavioural & social sciences ☒ Ecological, evolutionary & environmental sciences

For a reference copy of the document with all sections, see nature.com/documents/nr-reporting-summary-flat.pdf

Ecological, evolutionary & environmental sciences study design

All studies must disclose on these points even when the disclosure is negative.

Study description	We report an archosauromorph (choristodere) embryo of Ikechosaurus sp. inside a weakly mineralised-shelled egg, from the Lower Cretaceous of northeast China. Phylogenetic comparative analysis on extant and extinct amniotes, including the new fossil, confirm that archosauromorphs displayed EER across oviparity to viviparity, as seen in numerous extant squamates. We show that obligate oviparity evolved multiple times, and viviparity was the primitive reproductive mode, supporting the EER model for origin of the amniotic egg.
Research sample	The new specimen (MES-NJU 57004) was collected from yellowish white, thinly laminated tuffaceous mudstone of the Lower Cretaceous Jiufotang Formation (Jehol Biota, ca. 125–120 Ma) in the Lamagou locality adjacent to Chaoyang City, western Liaoning, northeastern China. The specimen is in a recognised, public institution, and available for study by anyone.
Sampling strategy	We only have one sample.
Data collection	In the phylogenetic analyses we provide all trait data as well as the parameters of the phylogenetic trees and evidence for dating those trees.
Timing and spatial scale	Not applied.
Data exclusions	No data were excluded.
Reproducibility	We provide the tree parameters, specimen dates, and trait data, as well as specification of software used (all of which is standard and available free of charge), so all our analyses can be readily repeated.
Randomization	This is applicable to the 'choice of phylogeny' question when running ancestral states analyses, and we provide full details of how thuis was carried out both in the parsimony and Bayesian analyses.
Blinding	Not applicable

Did the study involve field work? ☐ Yes ☒ No

Reporting for specific materials, systems and methods

We require information from authors about some types of materials, experimental systems and methods used in many studies. Here, indicate whether each material, system or method listed is relevant to your study. If you are not sure if a list item applies to your research, read the appropriate section before selecting a response.

Materials & experimental systems

n/a	Involved in the study	
☒	☐	Antibodies
☒	☐	Eukaryotic cell lines
☐	☒	Palaeontology and archaeology
☒	☐	Animals and other organisms
☒	☐	Human research participants
☒	☐	Clinical data
☒	☐	Dual use research of concern

Methods

n/a	Involved in the study	
☒	☐	ChIP-seq
☒	☐	Flow cytometry
☒	☐	MRI-based neuroimaging

Palaeontology and Archaeology

Specimen provenance	The specimen (MES-NJU 57004) was collected from yellowish white, thinly laminated tuffaceous mudstone of the Lower Cretaceous Jiufotang Formation in the Lamagou locality adjacent to Chaoyang City, western Liaoning, northeastern China by the Chinese researchers, according to all aspects of Chinese law
Specimen deposition	The specimen is deposited in the School of Earth Sciences and Engineering, Nanjing University to permit free access by other researchers.

Dating methods | No new dates are provided.

☒ Tick this box to confirm that the raw and calibrated dates are available in the paper or in Supplementary Information.

Ethics oversight | No ethical approval or guidance was required

Note that full information on the approval of the study protocol must also be provided in the manuscript.
